# Clinical utility and prospective of TMS–EEG: Updated review from an international expert group^☆^

**DOI:** 10.1016/j.clinph.2025.2111487

**Published:** 2026-04

**Authors:** Ulf Ziemann, Yang Bai, Fiona M. Baumer, Mikkel M. Beck, Paolo Belardinelli, Daniele Belvisi, Stephan Bender, Til Ole Bergmann, Marta Bortoletto, Silvia Casarotto, Elias Casula, Arthur R. Chaves, Daniel Ciampi de Andrade, Antonella Conte, Zafiris J. Daskalakis, Faranak Farzan, Fabio Ferrarelli, Paul B. Fitzgerald, Pedro C. Gordon, Christian Grefkes, Sylvain Harquel, Julio C. Hernandez-Pavon, Aron T. Hill, Kate E. Hoy, Friedhelm C. Hummel, Petro Julkunen, Elisa Kallioniemi, Corey J. Keller, Vasilios K. Kimiskidis, Melissa Kirkovski, Giacomo Koch, Giorgio Leodori, Pantelis Lioumis, Sara Määttä, Inbal Maidan, Marcello Massimini, Annerose Mengel, Johanna Metsomaa, Carlo Miniussi, Tuomas P. Mutanen, Yoshihiro Noda, Recep A. Ozdemir, Estelle Raffin, Lorenzo Rocchi, Nigel C. Rogasch, Mario Rosanova, Emiliano Santarnecchi, Simone Sarasso, Siobhan M. Schabrun, Mouhsin M. Shafi, Hartwig R. Siebner, Else A. Tolner, Leo Tomasevic, Sara Tremblay, Caroline Tscherpel, Domenica Veniero, Viviana Versace, Daphne Voineskos, Steve Vucic, Abraham Zangen, Christoph Zrenner, Risto J. Ilmoniemi

**Affiliations:** a Department of Neurology & Stroke, University of Tübingen, Tübingen, Germany; b Hertie Institute for Clinical Brain Research, University of Tübingen, Tübingen, Germany; c Center of Disorders of Consciousness Rehabilitation, Affiliated Rehabilitation Hospital, Jiangxi Medical College, Nanchang University, Nanchang, Jiangxi, China; d Rehabilitation Medicine Clinical Research Center of Jiangxi Province, Jiangxi, China; e Department of Neurology, Stanford University, Stanford, CA, USA; f Wu Tsai Neurosciences Institute, Stanford University, Stanford, USA; g Danish Research Centre for Magnetic Resonance, Centre for Functional and Diagnostic Imaging and Research, Copenhagen University Hospital-Amager and Hvidovre, Hvidovre, Denmark; h CiMeC, Center for Mind/Brain Sciences, University of Trento, Italy; i Department of Human Neurosciences, Sapienza University of Rome, Rome, Italy; j IRCCS Neuromed, Pozzilli, IS, Italy; k Department of Child and Adolescent Psychiatry, Medical Faculty and University Hospital, University of Cologne, Cologne, Germany; l Neuroimaging Center (NIC), Focus Program Translational Neuroscience (FTN), Johannes Gutenberg University Medical Center, Mainz, Germany; m Leibniz Institute for Resilience Research (LIR), Mainz, Germany; n Molecular Mind Laboratory, IMT School for Advanced Studies Lucca, Lucca, Italy; o Department of Biomedical and Clinical Sciences, University of Milan, Milan, Italy; p IRCCS Fondazione Don Carlo Gnocchi ONLUS, Milan, Italy; q Department of System Medicine, University of Tor Vergata, Rome, Italy; r Experimental Neuropsychophysiology Laboratory, IRCCS Santa Lucia Foundation, Rome, Italy; s The Royal’s Institute of Mental Health Research, Ottawa, Canada; t Interdisciplinary School of Health Sciences, Faculty of Health Sciences, University of Ottawa, Canada; u Center for Neuroplasticity and Pain, Department of Health Sciences and Technology, Faculty of Medicine, Aalborg University, Aalborg, Denmark; v Department of Psychiatry, University of California San Diego, La Jolla, USA; w eBrain Lab, School of Mechatronic Systems Engineering, Simon Fraser University, Surrey, British Columbia, Canada; x University of Toronto, Toronto, Ontario, Canada; y Centre for Addiction and Mental Health, Toronto, Ontario, Canada; z University of Pittsburgh School of Medicine, Pittsburgh, USA; aa School of Medicine and Psychology, The Australian National University, Canberra, Australia; ab Monarch Research Institute, Monarch Mental Health Group, Melbourne, Australia; ac Department of Neurology, Goethe University, Frankfurt am Main, Germany; ad Univ. Grenoble Alpes, Univ. Savoie Mont Blanc, CNRS, LPNC, Grenoble, France; ae Department of Psychological Sciences, Kansas State University, Manhattan, KS, USA; af Deakin University, Melbourne Burwood Campus, Australia; ag The Bionics Institute, Fitzroy, Australia; ah Defitech Chair of Clinical Neuroengineering, Neuro-X Institute (INX), École Polytechnique Fédérale de Lausanne (EPFL), Geneva, Switzerland; ai Defitech Chair of Clinical Neuroengineering, INX, EPFL Valais, Clinique Romande de Réadaptation, Sion, Switzerland; aj Clinical Neuroscience, University of Geneva Medical School, Geneva, Switzerland; ak Department of Technical Physics, University of Eastern Finland, Kuopio, Finland; al Department of Clinical Neurophysiology, Kuopio University Hospital, Kuopio, Finland; am Department of Biomedical Engineering, New Jersey Institute of Technology, Newark, NJ, USA; an Department of Psychiatry & Behavioral Sciences, Stanford University School of Medicine, Stanford, USA; ao Veterans Affairs Palo Alto Healthcare System, and the Sierra Pacific Mental Illness, Research, Education, and Clinical Center (MIRECC), Palo Alto, USA; ap First Department of Neurology, AHEPA University Hospital, Aristotle University of Thessaloniki, Thessaloniki, Greece; aq Department of Clinical and Behavioural Neurology, Santa Lucia Foundation IRCCS, Rome, Italy; ar Department of Neuroscience and Rehabilitation, University of Ferrara, and Center for Translational Neurophysiology of Speech and Communication (CTNSC), Italian Institute of Technology (IIT), Ferrara, Italy; as Department of Neuroscience and Biomedical Engineering, Aalto University, Espoo, Finland; at BioMag Laboratory, HUS Medical Imaging Center, University of Helsinki, Aalto University and Helsinki University Hospital, Helsinki, Finland; au Department of Clinical Neurophysiology, Diagnostic Imaging Center, Kuopio University Hospital, Kuopio, Finland; av Institute of Clinical Medicine, Clinical Neurophysiology, University of Eastern Finland, Kuopio, Finland; aw Laboratory of Early Markers of Neurodegeneration (LEMON), Neurological Institute, Tel Aviv Sourasky Medical Center, Tel Aviv, Israel; ax Gray Faculty of Medical and Health Sciences , Tel Aviv University, Tel Aviv, Israel; ay Sagol School of Neuroscience, Tel Aviv University, Tel Aviv, Israel; az Department of Neuropsychiatry, Keio University School of Medicine, Tokyo, Japan; ba Shinjuku-Yoyogi Mental Lab Clinic, Tokyo, Japan; bb Department of Psychiatry, International University of Health and Welfare, Mita Hospital, Tokyo, Japan; bc Berenson-Allen Center for Noninvasive Brain Stimulation, Department of Neurology, Beth Israel Deaconess Medical Center, Boston, MA, USA; bd Department of Neurology, Harvard Medical School, Boston, MA, USA; be Department of Medical Sciences and Public Health, University of Cagliari, Cagliari, Italy; bf School of Psychological Sciences and Turner Institute for Brain and Mental Health, Monash University, Melbourne, Australia; bg School of Biomedicine, University of Adelaide, Adelaide, Australia; bh Hopwood Centre for Neurobiology, Lifelong Health Theme, South Australian Health and Medical Research Institute (SAHMRI), Adelaide, Australia; bi Precision Neuroscience & Neuromodulation Program, Gordon Center for Medical Imaging, Massachusetts General Hospital, Harvard Medical School, Boston, MA, USA; bj The Gray Centre for Mobility and Activity, Parkwood Institute, St Joseph’s Healthcare, London, Ontario, Canada; bk School of Physical Therapy, University of Western Ontario, London, Ontario, Canada; bl Department of Clinical Medicine, Faculty of Health and Medical Sciences, University of Copenhagen, Copenhagen, Denmark; bm Department of Neurology, Copenhagen University Hospital Bispebjerg, Copenhagen, Denmark; bn Department of Human Genetics, Leiden University Medical Center, Leiden, the Netherlands; bo Department of Neurology, Leiden University Medical Center, Leiden, the Netherlands; bp Department of Psychiatry and Psychotherapy, University of Regensburg, Regensburg, Germany; bq Department of Human Sciences, Institute of Psychology, University of the Bundeswehr Munich, Neubiberg, Germany; br The University of Ottawa Brain and Mind Research Institute, Ottawa, Canada; bs Département de psychoéducation et de psychologie, Université du Québec en Outaouais, Gatineau, Canada; bt Department of Neuroscience, Carleton University, Ottawa, Canada; bu Department of Cellular and Molecular Medicine, University of Ottawa, Ottawa, Canada; bv School of Psychology, University of Nottingham, UK; bw Department of Neurorehabilitation, Hospital of Vipiteno (SABES-ASDAA), Vipiteno-Sterzing, Italy; bx Temerty Centre for Therapeutic Brain Intervention, Centre for Addiction and Mental Health, Toronto, Canada; by Department of Psychiatry, University of Toronto, Toronto, Canada; bz Poul Hansen Family Centre for Depression, University Health Network, Toronto, Canada; ca Brain and Nerve Research Centre, The University of Sydney, Sydney, Australia; cb Department of Life Science and the School of Brain Sciences and Cognition, Ben-Gurion University, Beer Sheba, Israel; cc Institute for Biomedical Engineering, University of Toronto, Toronto, Canada; cd Institute of Medical Science, University of Toronto, Toronto, Canada

**Keywords:** Transcranial magnetic stimulation, Electroencephalography, TMS-EEG, Cortical excitability, Effective connectivity

## Abstract

Transcranial magnetic stimulation (TMS) is a non-invasive technique to stimulate the brain, while electroencephalography (EEG) is a non-invasive technique to record its electrical activity. Their combined use (TMS–EEG) has been established only relatively recently, after successful development of TMS-compatible EEG amplifiers. TMS-EEG offers the unparalleled opportunity to directly perturb the brain with TMS and simultaneously record its response with EEG. This allows inferences on causal input–output relationships, therefore going critically beyond purely observational techniques, such as resting-state EEG or functional MRI, in the study of brain dynamics. This consensus review updates the work of Tremblay and coworkers [Clin Neurophysiol 2019; 130: 802–844]. Since then, substantial advances have been made in understanding contamination of TMS–EEG signals by physiological and non-physiological artifacts, as well as in developing strategies to avoid or control them. In parallel, new insights have emerged regarding the physiological mechanisms underlying TMS-EEG responses and their diagnostic and prognostic utility in a broad range of psychiatric and neurological disorders. As such, TMS-EEG is rapidly shaping a dynamic new field in clinical neurophysiology and neuroscience. This review provides a critical and comprehensive synthesis of current knowledge, including practical guidance for implementing TMS-EEG in the clinical setting.

## Highlights

• Updated review on transcranial magnetic stimulation – electroencephalography.
• Summarizes technology, instrumentation, artifacts, EEG analysis and physiological principles of TMS-EEG.
• Surveys TMS-EEG clinical utility and provides recommendations on TMS-EEG protocols for clinical practice.

## 1 Technique and instrumentation of TMS-EEG

### 1.1 TMS–EEG technique

Transcranial magnetic stimulation (TMS) is a non-invasive technique based on Faraday’s law of induction. In TMS, a magnetic pulse is generated by passing a strong (approximately 5 kA) and brief (a few hundred microseconds) current pulse through the windings of a coil (Ilmoniemi et al., 1999). This current creates a time-varying magnetic field that penetrates the scalp and skull, inducing an electric field (E-field) in a relatively focal area of the brain in close vicinity of the TMS coil (Ilmoniemi et al., 1997). The magnetic pulse, typically 1–3 Tesla, has a rise time of about 50–100 µs. TMS has a sub-millisecond temporal definition, which allows very precisely timed control of neuronal membrane potentials and modulation of ongoing and oscillatory brain activity (Hernandez-Pavon et al., 2023, Koponen et al., 2018b).

Electroencephalography (EEG) is a technique that measures the electrical activity of the brain with high temporal resolution from electrodes placed over the scalp, with the scalp placement and number of the electrodes generally determining the spatial resolution of the recording (Ilmoniemi and Sarvas, 2019).

When TMS is combined with EEG, brain activity can be recorded with millisecond-level temporal resolution, enabling the study of brain processes by 1) evoking neural activity with TMS and observing responses via EEG, 2) perturbing ongoing brain activity with TMS and observing its effects on EEG, or 3) synchronizing or driving TMS to the ongoing brain activity (Bergmann, 2018, Hernandez-Pavon et al., 2023, Ilmoniemi et al., 1997, Zrenner and Ziemann, 2024).

### 1.2 Instrumentation

The instrumentation required for acquiring TMS–EEG data generally includes the following components: a) TMS electronics and coil, b) TMS-compatible EEG amplifier, and c) neuronavigation (see also Fig. 5.1). In addition to these traditional elements, d) a graphical user interface (GUI) and e) a noise-masking system can be included to improve the quality of data acquisition (Casarotto et al., 2022, Fecchio et al., 2025, Hernandez-Pavon et al., 2023, Ilmoniemi and Kicic, 2010, Russo et al., 2022b). Overall, the chosen instrumentation has a significant role in ensuring the high quality and interpretability of the TMS–EEG recordings and the resulting responses. The clinical utility of TMS–EEG highly depends on the proper selection and implementation of instrumentation.

The spatial extent of the cortical area initially stimulated by TMS is influenced by factors such as coil geometry, stimulus intensity (SI), target area, and the coil-to-cortex distance (Deng et al., 2013a, Ilmoniemi et al., 1999). The spatial extension of the induced E-field is often expressed by the half-value spread, i.e., the ratio of half-value volume (defined as the volume of the brain tissue that is exposed to an electric field as strong as or stronger than half of the maximum E-field) divided by the half-value depth (defined as the radial distance from the cortical surface to the deepest point where the E-field strength is half of its maximum value on the cortical surface) (Deng et al., 2013b). The larger the half-value spread, the less focal the induced E-field is. Typical figure-of-eight coils have half-value depths of 1.2–1.4 cm and half-value spreads of 8–12 cm^2^, while non-focal round coils have half-value depths of 1.3–1.6 cm and half-value spreads of 50–70 cm^2^ (Deng et al., 2013b). Because the magnetic field diminishes rapidly with distance, and the induced E-field approaches zero towards the center of the head, TMS excites superficial cortical areas more strongly than deeper structures (Heller and van Hulsteyn, 1992, Thielscher et al., 2011). The TMS–EEG responses are also influenced by stimulation parameters, e.g., target location, E-field orientation at the target, SI, and ongoing neural activity (Casarotto et al., 2010, Fecchio et al., 2017). TMS-evoked potentials (TEPs) are among the most extensively studied responses and refer to the time-locked EEG response to TMS, reflecting both direct neuronal activation and synaptic activity. They consist of a series of detectable and analyzable waveform deflections (Casarotto et al., 2010, Lioumis et al., 2009).

When integrating TMS and EEG, ensuring technical compatibility between the two devices is crucial. Two main issues must be addressed: connectivity and interference. Connectivity refers to the communication between TMS and EEG systems and can be either one-way or two-way. In one-way connectivity, when collecting EEG responses, a reliable trigger should be available from TMS to EEG, assuming the TMS device is used to provide the trigger. Alternatively, the trigger could originate from the EEG system or a third-party device that synchronizes both TMS and EEG systems. Two-way connectivity involves bidirectional communication between the TMS and EEG systems, necessary when synchronizing EEG sampling with TMS or when triggering and receiving signals to precisely control stimulation timing and delays based on EEG.

TMS will introduce artifacts into the EEG recording, primarily due to the magnetic pulse and, to a lesser extent, inductive interference from stimulator recharge (Hernandez-Pavon et al., 2023, Ilmoniemi et al., 2015) (see Section 2). Mitigating these effects involves shielding the EEG from TMS and ensuring the TMS device is adequately shielded to prevent it from affecting other nearby devices. Rearranging the electrode’s lead wires can also help to minimize the TMS-pulse-induced artifacts (Sekiguchi et al., 2011). Addressing connectivity and interference is essential for the successful integration of TMS and EEG, and their consideration should guide the selection of the instrumentation and compatible system settings.

### 1.3 TMS electronics and coils

TMS stimulators are typically classified based on the type of pulse waveform they can generate, primarily monophasic and biphasic. Depending on the pulse waveform, they can deliver different types of patterned stimulation, from single-pulse TMS, paired-pulse, to regular repetitive TMS (rTMS), and patterned rTMS, such as theta burst stimulation (TBS).

Regardless of the stimulator used, for concurrent EEG recording, a relevant property is its ability to control the recharge delay. TMS capacitors' recharge can introduce electrical artifacts in EEG recordings (see Section 2). This can appear as a slow decay, a spike, or a signal jump, depending on the equipment, and may overlap with the signal of interest (Veniero et al., 2009). To prevent this, many modern stimulators offer a recharge delay option that allows control over the timing of capacitor recharge, thereby avoiding artifacts during the evoked response. The recharge delay should then be set outside the time window of interest.

Monophasic and biphasic waveforms are the two most common TMS pulse waveforms. They are characterized by a different amplitude ratio of the first and second phases of the E-field. A technical description of the pulse waveform is outside the scope of this paper (for more details, see, for instance, Hernandez-Pavon et al. (2023)), but when combining TMS with EEG, it might be worth considering that the stimulation threshold for monophasic stimulators is generally higher than the one obtained with biphasic pulses (Säisänen et al., 2008). Nonetheless, the final choice will depend on the experimental design.

The literature on the effects of different TMS waveforms on TEPs is sparse and often mixed with the effects of induced current directions. There is some evidence that while monophasic stimulators might increase the amplitude of some components, they might be unable to elicit early latency TEPs consistently (Casula et al., 2018b, Guidali et al., 2023). A few studies have reported that coil orientations influence TEP polarities for components peaking between 20 and 45 ms (Bonato et al., 2006, Casula et al., 2018b), with monophasic pulses unexpectedly reported to evoke a larger global mean field power (GMFP) than biphasic stimuli. However, it is worth noting that in the study by Casula et al. (2018b), the duration of the pulses was also manipulated and set to be slightly shorter compared to commercially available simulators. A recent study suggests that early latency TEPs might be affected by the current direction and pulse waveform differently compared to those described in earlier studies (Guidali et al., 2023). They investigated the effect of three current directions (posterior to anterior (PA), anterior to posterior (AP), lateral to medial (LM)) and monophasic vs. biphasic stimuli on a TEP component elicited by M1 stimulation that peaks 15 ms after pulse delivery. When comparing current directions, they found no change in polarity but a change in amplitude and latency. The responses to the monophasic stimulator critically depended on the current direction, with LM and PA stimulation unable to elicit the component of interest. Monophasic AP current direction evoked the strongest TEPs, which is in line with the results of Casula et al. (2022a). Latencies were also affected, with the AP current direction slowing down the component by several milliseconds. Granö et al. (2025) stimulated the pre-supplementary motor area (pre-SMA) in 36 different E-field directions and performed EEG source analysis. Early (20 and 40 ms) TEP components depended strongly on E-field direction. They concluded that different orientations may activate or perturb different neuronal networks. The TEP amplitude and latency modulations, which depend on the stimulation parameters, confirm the importance of coil placement and stimulator waveform and support the notion that different current directions activate different neuronal populations with different relative vigor.

Choosing a TMS coil depends primarily on the specific TMS protocol being implemented. The shape, size, and winding of the coil affect the induced E-field, which influences both the focality and depth of stimulation, ultimately determining the volume of the brain that is activated. The most used TMS coil for concurrent EEG recording is the figure-eight coil (Ueno et al., 1988). While the coil type does not seem to affect the duration of the electromagnetic artifact (Veniero et al., 2009), it will affect the number of electrode leads being directly stimulated, and it may influence the magnitude of cranial muscles stimulation, which can, in turn, affect EEG recordings (for a detailed discussion, see Hernandez-Pavon et al. (2023)). Muscle artifacts are particularly difficult to remove (Hernandez-Pavon et al., 2022, Hernandez-Pavon et al., 2012); it is therefore crucial to consider the activation of scalp, facial, and neck muscles during TMS, as certain coils—like the double-cone coil—can induce significant muscle twitches (Fernandez et al., 2021, Fong et al., 2023).

Recently, a new brain stimulation technique called multi-locus TMS (mTMS) was introduced (Koponen et al., 2018a, Nieminen et al., 2022). The mTMS allows for electronically controlled stimulation of multiple brain areas at different times and intensities without moving the coil (Koponen et al., 2018a). It will enable automated EEG-feedback-controlled stimulation, which can help reduce artifacts and enable more precise targeting of brain networks of interest (Lioumis et al., 2025, Sinisalo et al., 2024).

### 1.4 TMS-compatible EEG amplifier

One of the main methodological challenges of recording EEG during TMS is the strong E-field generated by the magnetic pulse, which can saturate the recording amplifiers for several seconds. To address this issue, a sample-and-hold circuit was introduced to stabilize the EEG signal (Virtanen et al., 1999). This circuit held the EEG signal unchanged for a few milliseconds after TMS delivery, thus preventing the recording amplifiers from saturating and allowing for the capture of the response after the hold period (Iramina et al., 2003, Virtanen et al., 1999). In recent years, a newer generation of amplifiers has gained popularity, replacing the sample-and-hold circuit approach. These amplifiers are designed to operate in high time-varying magnetic fields: they effectively avoid saturation and enable continuous EEG acquisition by combining a high dynamic range and high sampling rates. For instance, with a sampling rate of approximately 20 kHz, the artifact duration is a little over 2 ms. Since data files increase in size with the sampling rate, TMS–EEG recordings are often performed using a 5 kHz sampling rate, which keeps the pulse artifact as short as 5 ms with most amplifiers (Jamil et al., 2024). Furthermore, DC amplifiers are preferable to AC amplifiers because high-pass filters can interact with the pulse artifact, introducing artificial trends or drifts in the signal surrounding the TMS pulse. One important caveat is that, regardless of the specific acquisition parameters, proper EEG preparation is crucial to ensure high-quality data collection. TMS–EEG recordings indeed suffer from high electrode impedance values and are influenced by electrode wiring (see Section 27: Protocols to measure TMS–EEG responses).

### 1.5 Neuronavigation

Neuronavigation is strongly recommended to maintain the position, orientation, and angulation of the TMS coil consistently throughout a session and across multiple visits, especially when conducting longitudinal measurements (Lioumis and Rosanova, 2022, Sack et al., 2009). Research has demonstrated that navigated TMS (nTMS) maximizes both accuracy and precision (repeatability) in TMS positioning (Caulfield et al., 2022, Hannula and Ilmoniemi, 2017). Navigation is essential in studies involving patients with structural brain lesions, as stimulation of severely damaged areas may not elicit any EEG responses (Gosseries et al., 2015). In general, one should consider that to obtain TEPs (or TMS-related oscillations) with a high signal-to-noise ratio, it is necessary to obtain and average responses over multiple (typically > 100) trials, and to maintain throughout a constant coil configuration with respect to the participant’s head and cortical anatomy at the target site.

With nTMS, the position and orientation of the coil are monitored in real time, ensuring proper stimulation of the target area throughout the experimental session and between sessions (Danner et al., 2008, Ilmoniemi and Kicic, 2010, Ruohonen and Karhu, 2010). This real-time monitoring reduces inter-trial variability in TMS–EEG recordings caused by coil movement and enhances accuracy by minimizing the risk of stimulating slightly different areas (Bashir et al., 2011). Furthermore, since neuronavigation systems can store information about coil position and orientation, they provide consistent targeting across multiple sessions, which contributes to reproducibility (Casarotto et al., 2010, Lioumis et al., 2009). In addition, this information can be utilized in post-hoc analyses to investigate to what extent changes in coil position and orientation have affected TMS-EEG responses.

### 1.6 Graphical user interface (GUI)

An effective strategy to improve data acquisition is the use of a graphical user interface (GUI) that enables real-time evaluation of TMS-EEG responses. The idea is twofold: first, to minimize the occurrence of artifacts (e.g., scalp muscle activation, auditory potentials, decay artifacts, recharge artifacts), which otherwise would go unnoticed and must be removed offline during data analysis, with the risk of altering genuine brain responses. Second, to verify the effectiveness of a specific set of stimulation parameters (location, SI, angle) by measuring the amplitude of the early and local EEG deflections evoked by TMS.

### 1.7 Noise masking system

A loud click sound is produced by the vibration of the windings of the coil during current flow. This click sound is synchronous with the TMS pulse and evokes an auditory potential superposed on the genuine brain responses to TMS (Nikouline et al., 1999, ter Braack et al., 2015) (Section 2). This biological confounding factor can be controlled by continuously playing a masking noise through in-ear earphones during pulse delivery. This approach minimizes the occurrence of TMS-evoked auditory potentials. Successful masking at reduced loudness can be obtained by customizing the spectral characteristics of the noise based on the actual coil's click, using freely available software (Russo et al., 2022b).

## 2 Non-physiological and physiological artifacts

One of the largest challenges in recording EEG data following a TMS pulse is the low signal-to-noise ratio (SNR) resulting from the wide range of artifacts introduced by stimulation (Hernandez-Pavon et al., 2023). The signals or interest are synchronized changes in neural activity resulting from transcranial excitation of cortical neurons by the TMS pulse, resulting in a series of positive and negative deflections in the EEG signal referred to as TMS evoked potentials (TEPs). Noise is introduced by multiple sources. First, there are physiological artifacts which are defined as electrical signals generated by non-neural, biological organs like the eyes, muscles, and the heart. Second, cortical activity indirectly triggered by the TMS pulse through co-stimulation of sensory systems is another physiological artifact that contributes to noise in TMS-EEG studies. Third, non-physiological artifacts arise from the interaction of exogenous electromagnetic signals (e.g., lights, computers, laboratory equipment, the electromagnetic field generated by the TMS pulse) with the EEG recording equipment. Recognizing and minimizing artifacts, and mitigating their impact on the genuine EEG response to transcranial cortex stimulation, are crucial for the scientific and clinical utility of TMS-EEG data. The way TMS-related artifacts are addressed may contribute to the differences in TEPs recently reported between research groups (Beck et al., 2024b, Belardinelli et al., 2019, Siebner et al., 2019). Recognizing and minimizing artifacts is particularly important in clinical TMS-EEG studies, where different artifact profiles between participant groups can lead to misinterpretation of group-related differences in TEPs. In this section, we will first discuss common EEG-related artifacts, before introducing artifacts more specific to concurrent TMS-EEG recordings. For each artifact type, we will describe the features, causes, and approaches to avoid/minimize the artifact in the EEG signal.

### 2.1 Common EEG-related artifacts

EEG records the fluctuations in voltage caused by the movement of ions into and out of neurons that are detectable across the skull/scalp (Buzsaki et al., 2012). The signal recorded at any given scalp electrode represents the *difference* between the potential at the recording/active electrode and the potential at a reference electrode measured across time (Jackson and Bolger, 2014). The reference electrode is usually placed in a location less sensitive to neural activity like the mastoid bone, the ear lobe, or the forehead. In particular, it is advisable to position the reference electrode at a significant distance from the TMS coil to minimize interference and TMS-induced artifacts in all channels (Hernandez-Pavon et al., 2023). The small changes in voltage occurring in the brain are attenuated as they pass through biological tissue like the cerebrospinal fluid, bone, and skin, before reaching the scalp electrodes, resulting in an even smaller detectable signal. Furthermore, these neural signals undergo constructive and destructive interference according to the orientation and location of the underlying neural generators, which can either reduce or enhance the amplitude of the signal (Nunez and Srinivasan, 2006). To record these small fluctuations in voltage, EEG amplifiers are highly sensitive and are capable of recording voltage changes < 1 μV in amplitude.

EEG recording equipment is not only sensitive to changes in voltage resulting from neural activity (the signal of interest), but also to any other electromagnetic interference in the vicinity of the recording equipment (artifacts). These artifacts originate from both physiological and non-physiological sources and are often several orders of magnitude larger in amplitude than the signal of interest (Luck, 2014, Uriguen and Garcia-Zapirain, 2015). The following sources of artifact are common to all EEG recordings, including those with concurrent TMS.

*Line-noise:* One of the most common sources of non-physiological artifacts is line-noise. Electrical equipment including lights, computers and TMS devices are powered by alternating current (i.e., current that periodically changes direction), which fluctuates at either 50 or 60 Hz, depending on the country. The electrode lead wires which connect the scalp electrodes to the EEG (pre-)amplifier act as antennas, detecting the electromagnetic fields generated by these alternating currents sources and resulting in 50- or 60-Hz fluctuations in the EEG signal, known as line-noise. Differential amplifiers minimize line noise by utilizing common-mode rejection, which refers to the ability of a recording system to suppress signals shared by both the active and reference electrodes (e.g., line-noise) (Kappenman and Luck, 2010). This is achieved by first subtracting the signal recorded from a third ‘ground’ electrode from both the active and reference electrodes, and then calculating their difference. The ground electrode should not be confused with the earth ground, which is a physical connection of the recording equipment to the ground. Connecting participants to the earth ground is unsafe due to the risk of electrocution. Common-mode rejection is further enhanced by ensuring low and equal impedance between electrodes (Kappenman and Luck, 2010). Impedance refers to factors which oppose the current flow. In the amplifier, input impedance is determined by the physical properties of the components and is a fixed value. At the skin-electrode interface, factors affecting impedance can differ between participants, recording sites and across time, and include the material of the electrode, hair follicles, the outer layer of the skin, sweat and other oils on the skin (Shad et al., 2020). Impedance is often minimized by lightly abrading the skin under the electrode to remove the dead skin cells and oils, and introducing a conductive gel between the electrode surface and skin. In practice, obtaining equal impedance between electrodes is unachievable, resulting in some line-noise contamination. Line noise can be further reduced by isolating participants from electrical noise in a Faraday cage, although this is not common due to additional hardware requirements (Luck, 2014). Another approach is to use ‘active’ electrodes which include a preamplifier built into the electrode, thereby minimizing line-noise detection in the lead wire (Shad et al., 2020). Temporal band-stop filters centered on 50 or 60 Hz are often used either during recording or offline to further suppress line noise, although these can lead to ringing artifacts (de Cheveigné and Nelken, 2019, Leske and Dalal, 2019, Widmann et al., 2015). Regression methods can also be used to reduce the contamination of the signal by line-noise (Bigdely-Shamlo et al., 2015).

*Cephalic skin potentials:* Slow changes in impedance over an experiment (e.g., if the participant is sweating, or long experiments leading to the drying of the conductive gel) result in artifacts known as cephalic skin potentials, which manifest as low frequency (<5 Hz) voltage drifts in the EEG signal (Kappenman and Luck, 2010). Cephalic skin potentials can be minimized by keeping the recording environment cool and dry, and achieving low impedance by gently abrading the skin during electrode preparation (Kappenman and Luck, 2010). Of note, puncturing the skin with a needle (also called mini-puncture) is the most effective method for minimizing cephalic skin potentials (Burbank, 1978), but is not commonly used due to the potential for discomfort. In some instances, cephalic skin potentials are minimized off-line using high-pass filters (Kappenman and Luck, 2010); however, these can also alter the underlying neural signal, for example affecting the amplitude and latency of certain event related potentials (ERPs) (Tanner et al., 2015).

*Electrode movement:* Disruption of the skin–electrode interface caused by electrode movement (e.g., participant motion, scratching the head, or the experimenter adjusting an electrode) can produce abrupt voltage shifts due to sudden changes in impedance (Gorjan et al., 2022). Movement artifacts are minimized by ensuring the participant and experimenter do not touch the electrodes during recording. If electrode movement does occur, affected periods of signal should be removed off-line prior to analysis.

*Clipping:* Clipping (or truncation) is an artifact that occurs when the EEG signal exceeds the amplifier's input range, resulting in a flat line in the signal (Light et al., 2010). The input range is determined by the amplifier's output range (a fixed specification) and the gain (the level of signal amplification). Clipping can be minimized in real-time by ensuring an adequate input range to capture the largest signal possible and applying online high-pass filters. These filters keep the signal centered within the amplifier's range, maximizing the recordable voltage changes (AC coupling). In contrast, DC coupling, without filters, allows the signal to rest at any voltage, increasing the risk of clipping. If clipping occurs, the affected EEG segment should be removed before analysis (Light et al., 2010).

*Eye blinks and* movement*:* Eye movements produce bilateral, synchronous deflections in the EEG due to their anterior origin, with both eyes acting as a linked source. The eye generates a constant dipole independent of light, with a positive pole at the cornea and a negative source at the retina. As the eyeballs move, this dipole shifts accordingly, creating a large extracerebral surface potential that varies in polarity and amplitude depending on the direction and speed of eye movement (Tatum et al., 2011).

During spontaneous blinking, the eyelids close while the eyeballs roll upward, resulting in a relative positivity in frontopolar electrodes—recognized as the vertical eyeblink artifact on EEG. Horizontal eye movements cause artifacts in lateral electrodes, with a relative positivity in the direction of movement and a corresponding negative phase reversal in the opposite electrodes (Berg and Scherg, 1991, Lins et al., 1993). Potentials generated by eye movements are most prominent in frontopolar and frontal lateral electrodes. Additional channels placed around the eyes can improve artifact detection and characterization (Croft and Barry, 2000).

To minimize eye movement artifacts during EEG recording, subjects are typically instructed to fixate on a cross displayed on a screen. However, voluntary inhibition of blinking should be avoided, as it may introduce unintended alterations in brain activity (Berman et al., 2012). In the context of TMS-EEG, spontaneous eye movements are not time-locked to TMS, meaning they are statistically independent of TMS-evoked EEG responses and can be effectively removed using independent component analysis (ICA) or similar techniques without significant risk of overcorrection (Huang et al., 2020, Jurczak et al., 2022). However, blinks time-locked to the TMS pulse can also occur, which present additional challenges (see *TMS-evoked eye blinks*).

*Cardiac activity:* Electrocardiographic (ECG) contamination in EEG recordings occurs due to volume conduction of cardiac activity to scalp electrodes. The heart generates strong electrical potentials that propagate through surrounding tissues, including the scalp and skull (Dirlich et al., 1997). ECG artifacts typically present as rhythmic waveforms corresponding to the cardiac cycle, often resembling the QRS complex. In some cases, particularly in recordings with high electrode impedance or in individuals with strong cardiac signals, the artifact can be substantial enough to obscure underlying EEG activity (Tatum et al., 2011).

ECG contamination is most prominent in inferior and frontal scalp regions, where electrodes are positioned closer to the heart. Additionally, the trajectory of the cardiac vector generated by the ventricles can lead to pronounced QRS complexes in temporal electrodes. Certain individuals, such as those with short or stocky necks − common in overweight individuals and infants − are predisposed to ECG artifacts because the heart’s electrical dipole is positioned closer to the scalp, allowing stronger signal transmission. High electrode impedance further exacerbates ECG contamination by reducing common mode rejection, making electrodes more susceptible to picking up distant electrical sources (Gordon, 1980).

ECG artifacts can be identified by recording the ECG with electrodes placed over the left and right chest in a bipolar configuration, approximating the V2 axis of a standard ECG. The interference typically appears as a periodic waveform, recurring at approximately 1 Hz. Opposite polarities of the R-wave in the QRS complex can be observed as a negative potential on the left and a positive potential on the right in ear electrodes. The periodicity and synchrony between the EEG and ECG signals confirm the artifact’s origin (Tatum et al., 2011).

Several strategies exist to mitigate ECG contamination in EEG recordings. Preventative approaches include careful electrode placement to minimize differential pickup of ECG signals, lowering impedance, and using additional reference electrodes (e.g., mastoids or chest) to better isolate the artifact. Post-processing techniques for ECG artifact removal vary depending on the number of EEG channels available. Multichannel EEG approaches employ blind source separation methods, such as ICA and principal component analysis (PCA). Single-channel EEG corrections include correlation- or regression-based techniques that subtract ECG signals (Devuyst et al., 2008, Hamaneh et al., 2014), adaptive filtering or template subtraction (Dora and Biswal, 2020, Navarro et al., 2012).

*Spontaneous craniofacial* muscle *activity:* Craniofacial muscles are a common source of electromyographic (EMG) signals, which may appear on the EEG as continuous activity or short bursts, depending on the type of muscle contraction involved. The frontalis and temporalis muscles are primary sources of myogenic artifacts, the former particularly active during eye closure and the latter involved in chewing and jaw clenching. Activation of these muscles results in EMG predominantly recorded by frontopolar/frontal electrodes for the frontalis and lateral frontal/temporal electrodes for the temporalis. Additionally, brief bursts of EMG activity may arise from the frontalis or lateral rectus muscles during eye movements (Tatum et al., 2011). EMG activity has a higher frequency than EEG activity, typically peaking between 100–150 Hz.

To minimize EMG contamination, subjects should be instructed to avoid forced eye closure, relax their jaw, or slightly open their mouth during recording. As with eye movement artifacts, spontaneous EMG activity is not time-locked to TMS pulses, allowing for effective removal using techniques such as ICA (Fitzgibbon et al., 2016, Frolich and Dowding, 2018, Liu et al., 2019). This is particularly important when analyzing EEG frequencies in the beta and gamma range, as muscle activity significantly overlaps with these frequency bands. This EMG activity can be 10–200 times larger than EEG in the 20–100 Hz range, especially in lateral electrodes (Whitham et al., 2007).

### 2.2 EEG artifacts specific to TMS

In addition to the common artifacts in standard EEG recordings, TMS introduces additional non-physiological and physiological artifacts, potentially distorting neural signals of interest (Hernandez-Pavon et al., 2023, Ilmoniemi and Kicic, 2010, Rogasch et al., 2017).

**Fig. 2.1:**
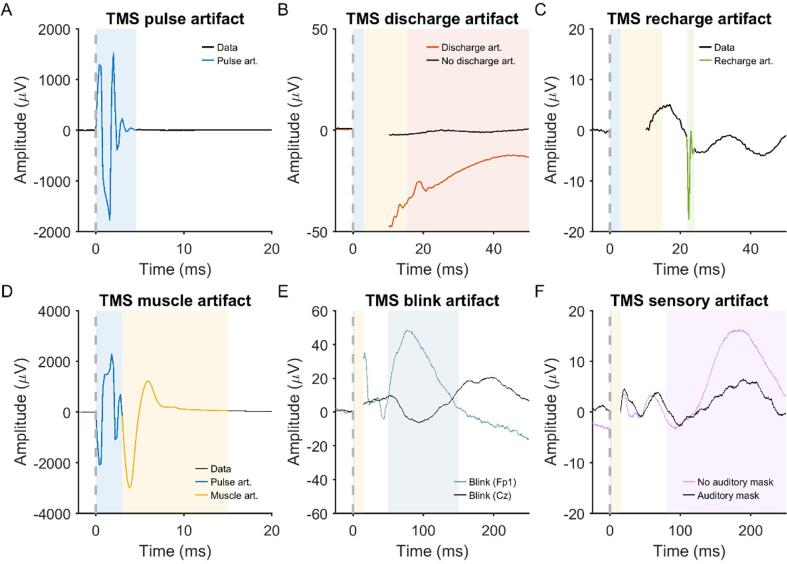
Example of non-physiological and physiological artifacts in TMS-EEG data. Data are the average of 120 trials in individual participants following epoching around the TMS pulse (time = 0 ms), baseline correction and re-referencing to the average of all electrodes. Shaded areas represent the expected time range of artifacts. **A:** TMS pulse artifact (blue). **B:** TMS discharge artifact (red). **C:** TMS recharge artifact (green). **D:** TMS muscle artifact (yellow). **E:** TMS blink artifact (teal). **F:** TMS sensory artifact (purple). Note the different x and y axis scaling between plots. Data during the TMS pulse/muscle artifact time period were removed in plots B, C, E and F. (For interpretation of the references to colour in this figure legend, the reader is referred to the web version of this article.)

*Pulse artifact:* The time-varying electromagnetic field resulting from the TMS pulse causes a large (>10 mV) and fast (<0.25 ms) non-physiological artifact referred to as TMS pulse artifact (Fig. 2.1A). In older EEG amplifiers, the TMS pulse artifact saturated (i.e., ‘clipped’) the amplifiers, with the signal taking hundreds to thousands of milliseconds to return to the operating range required for recording neural signals in electrodes near the coil, thus preventing the detection of TEPs (e.g., (Izumi et al., 1997)). One approach to minimize the TMS pulse artifact uses sample-and-hold circuits, which pins the input of the EEG amplifier for several milliseconds around the TMS pulse (i.e., keeps the signal close to 0 μV). This prevents saturation of the amplifier and allows recording of EEG signals within several milliseconds following the pulse (Ilmoniemi et al., 1997, Virtanen et al., 1999). The second and most common approach utilizes EEG amplifiers with large input ranges (> ±2.5 mV), high resolution (> 16 bit) and high sampling rates (> 5 kHz) to adequately capture the TMS pulse artifact without causing amplifier saturation (Bonato et al., 2006).

Although the improved input range of modern amplifiers minimizes saturation, the large and high-frequency voltage change caused by the TMS pulse can interact with online filters, resulting in additional ‘ripple’ or ‘ringing’ artifacts which prolong artifact recovery (Rogasch et al., 2013b, Veniero et al., 2009). High-pass filters can be avoided by using DC-coupled amplifiers. Low-pass filters (also called anti-aliasing filters) cannot be avoided and are dependent on the sampling rate of data acquisition. Higher sampling rates permit low-pass filters with higher cut-off frequencies, which have to be set to half of the sampling rate or lower to prevent aliasing artifacts. Lowering the cutoff frequency of the low-pass filter reduces the amplitude of the TMS pulse artifact but prolongs its recovery time (Freche et al., 2018, Jamil et al., 2024). Therefore, higher sampling rates and higher low-pass filter frequency cutoffs are recommended to shorten the recovery of the pulse artifact as much as possible (Hernandez-Pavon et al., 2023). For example, sampling rates of 5–10 kHz (low-pass filter = 1–2 kHz) result in pulse artifacts that recover within 5–6 ms (Rogasch et al., 2013b, Veniero et al., 2009), whereas recovery within 1–2 ms has been reported for sampling rates of 50 kHz (low-pass filter = 10.3 kHz) (Beck et al., 2024a). Some systems also synchronize the timing of the TMS pulse to the EEG sampling (Tomasevic et al., 2017). This approach results in a more consistent pulse artifact shape but has minimal impact on recovery time (Jamil et al., 2024).

*Discharge artifact:* The charges induced by the TMS pulse accumulate at the skin-gel-electrode interface and gradually dissipate, causing a discharge artifact (also known as decay artifact; Fig. 2.1B). The discharge artifact can persist for several hundred milliseconds, significantly offsetting the EEG signal (Freche et al., 2018, Rogasch et al., 2013b, Virtanen et al., 1999). The discharge artifact has been modelled as an exponential decay or the sum of two exponentials (Casula et al., 2017a, Litvak et al., 2007). However, Freche et al. (2018) found that a second order power law provided the best fit, aligning with a quantitative physical model of charge storage at the skin-electrode interface. The size and duration of the discharge artifact can be reduced by appropriate skin preparation: Reducing the skin impedance/resistance by exfoliating the skin (e.g., rubbing the skin with an abrasive paste prior to electrode placement) or puncturing the epidermis with a needle (e.g., mini-puncture) reduces the amplitude and duration of the discharge artifact (Freche et al., 2018, Julkunen et al., 2008). The material and design of the EEG electrodes also impact the discharge artifact, with sintered silver/silver-chloride pellet electrodes, and electrodes with a slit introduced to prevent the buildup of eddy currents (C-ring slit electrodes) reducing the discharge artifact compared to ring electrodes (Virtanen et al., 1999). Furthermore, orientating the recording and reference electrode lead wires in opposite directions and perpendicular to the coil can also reduce the discharge artifact, purportedly by cancelling electromotive forces (Sekiguchi et al., 2011). During data pre-processing, the discharge artifact can be minimized by removing channels with large discharge artifacts (Farzan et al., 2016), fitting and subtracting exponential or power law models from the data (Casula et al., 2017a, Freche et al., 2018), using ICA or PCA to identify and remove components representing the discharge artifact (Hernandez-Pavon et al., 2022, Rogasch et al., 2014), or using SOUND to suppress electrode noise (Mutanen et al., 2018, Rogasch et al., 2022). Of note, discharge artifacts are time-locked to the TMS pulse, which violates assumptions of ICA, increasing the risk of overcorrection with this method.

*Recharge artifact:* The TMS pulse is generated by discharging the device’s capacitors, which must then recharge before the next pulse. The recharging process can create a sharp artifact in the EEG signal (Veniero et al., 2009) (Fig. 2.1C). The timing, shape and amplitude of the recharge artifact depend on the TMS device, coil and stimulation intensity, with default recharging in some devices occurring within 100–200 ms of the TMS pulse (Veniero et al., 2009). However, most TMS devices allow users to control the recharge timing via the device interface or external software (Habibollahi Saatlou et al., 2018). Delaying capacitor recharge to a period outside the time window of interest for TEPs (e.g., 800–1000 ms post-TMS) addresses this problem. Alternatively, recharge artifacts can be minimized by removing and interpolating affected data segments, applying median filters (Rogasch et al., 2017) or via ICA.

*Electrode heating and movement:* Additional sources of artifact can arise from electrode heating during stimulation due to the buildup of eddy currents. Introducing cuts into silver/silver-chloride ring electrodes or using pellet electrodes substantially reduces the risk of electrode heating (Roth et al., 1992, Virtanen et al., 1999). Furthermore, movement of the electrode resulting from contact with the TMS coil can also introduce additional noise, particularly in low frequencies (<7 Hz). Creating a barrier between the coil and the electrode using either a thin layer of foam or a plastic spacer can reduce electrode movement-related artifacts (Ruddy et al., 2018).

*TMS-evoked craniofacial muscle activity:* Cranial muscle contractions are frequently induced by TMS, particularly at higher stimulation intensities and when lateral or posterior cortical areas are targeted (Mutanen et al., 2013a) (Fig. 2.1D). Commonly activated cranial muscles during TMS of lateral cortical regions include the frontalis, orbicularis oculi, temporalis, and masseter. These contractions result from the activation of low-threshold intramuscular motor nerve endings (Troni et al., 1983) and occur when TMS is applied to scalp regions in vicinity to scalp muscles (Fakoya et al., 2025, Rogasch et al., 2014). Additionally, TMS pulses may depolarize motor axons within a cranial nerve trunk, leading to the generation of a compound muscle action potential (CMAP) in the muscles innervated by that nerve, or MEPs when TMS activates facial representations in motor cortex (Groppa et al., 2012). In such cases, CMAPs or MEPs may arise in muscles that are not necessarily close to the stimulation site, particularly in the facial and trigeminal nerve territories. TMS-evoked muscle activity through excitation of low-threshold intramuscular motor nerve endings has very short onset latencies of <2 ms, while CMAPs evoked by excitation of the facial, trigeminal or accessorius nerves or MEPs evoked by activation of motor cortex have onset latencies in the range of 2.5–12 ms (Rösler et al., 1989, Schmid et al., 1992, Strenge and Jahns, 1998) (Fig. 2.1D). Therefore, TMS-evoked muscle activity always contaminates early TMS-EEG responses.

Facial muscle activation due to direct stimulation of the facial nerve is most commonly observed during lateral cortical stimulation, particularly with round coils (Benecke et al., 1988, Maccabee et al., 1988, Schriefer et al., 1988, Seki et al., 1990), though it can also occur with figure-of-eight coils (Rodel et al., 1999, Tokimura et al., 1993). In contrast, activation of masticatory muscles via stimulation of the motor component of the trigeminal nerve is more difficult to achieve and typically requires round coils (Macaluso et al., 1990, Schmid et al., 1995). Stimulation of the cerebellum, in particular with non-focal double-cone coils may induce strong activation of neck muscles (Fernandez et al., 2021, Fong et al., 2023). Regardless of how it is elicited, TMS-induced EMG activity appears as a time-locked, bi- or triphasic deflection with an onset latency of a few milliseconds and a duration characteristic of muscle fiber action potentials (tens of milliseconds) (Fig. 2.1D) (Korhonen et al., 2011, Rogasch et al., 2013b), and may have large amplitude, that can exceed the underlying TMS-EEG cortical responses up to several orders of magnitude. Therefore, TMS-induced EMG contamination should be avoided during data collection as much as possible, as it distorts early TMS-EEG signals.

To reduce cranial EMG activation, minor adjustments to coil position and orientation can help (Casarotto et al., 2022, Mutanen et al., 2013a, Parmigiani et al., 2025). Such modifications are not always feasible when precise targeting is required, as coil rotation itself influences TMS-EEG responses. Offline ICA is commonly employed to remove TMS-locked muscle artifacts (Rogasch et al., 2014) but, given the overlap of TMS-evoked muscle artifacts with early TMS-EEG responses and their time-locked nature, there is a considerable risk of overcorrection (Atti et al., 2024, Biabani et al., 2024). Alternative approaches, such as Signal Space Projection − Source Informed Recovery (SSP-SIR), have been proposed (Mutanen et al., 2016), and show advantages over ICA in simulated data under some circumstances (Mutanen et al., 2024). However, it remains unclear to what extent SSP-SIR offers practical advantages over ICA when applied to real data (Bertazzoli et al., 2021, Brancaccio et al., 2024, Mancuso et al., 2024, Mancuso et al., 2021, Rogasch et al., 2020). Therefore, one should avoid or minimize TMS-evoked muscle artifacts whenever possible.

*TMS-evoked eye blinks:* TMS elicits eye blinks time-locked to the TMS pulse following stimulation over frontal, motor and parieto-occipital locations (Corthout et al., 2011, Sohn et al., 2004). The genesis of these eye movements is likely multifactorial and dependent on the location of stimulation and the type of coil (circular vs figure-of-eight). Likely mechanisms include stimulation of the trigeminal nerve resulting in a reflex blink, or stimulation of the motor cortical region responsible for eye movements (Sohn et al., 2004). TMS-induced blinks can occur as early as 10–12 ms post stimulation and often peaks ∼60–140 ms post stimulation (Fig. 2.1**E**) (Corthout et al., 2011). While TMS-related blinks are well documented, other types of eye movement like saccades have not been reported following TMS.

Blinking engages periocular muscles, generating high-frequency electromyographic activity, introducing broad-spectrum noise into EEG recordings, in addition to the blink artifact. Since eye blinks are produced by frontalis and orbicularis oculi muscles close to frontal EEG electrodes, the EMG signal enhances the signal in these electrodes. Rapid eyelid movements can also cause minor shifts in scalp electrodes or changes in electrode impedance. Finally, blinking momentarily reduces light input, potentially altering neuronal activity in the visual cortex.

Time-locked blinks in the EEG signal have been reported following stimulation of the dorsolateral prefrontal cortex in adults (Rogasch et al., 2014) and motor cortex in children (Bruckmann et al., 2012). There are no systematic methods for avoiding TMS-induced eyeblinks; however, adjusting the coil position and angle have anecdotally been reported to minimize them. Offline approaches like ICA have been used to suppress TMS-locked eyeblinks (Rogasch et al., 2014) but their time-locked nature may weaken the assumptions of ICA, which impact the accuracy of correction (Atti et al., 2024).

*TMS-evoked sensory potentials:* The activation of the TMS coil generates multimodal sensory inputs that elicit neural responses, which overlap with the EEG responses to direct cortical activation by TMS (Nikouline et al., 1999, Paus et al., 2001). The TMS coil activation produces a high-pitched “click” sound that is clearly perceived by participants. Additionally, the electric field induced by TMS can excite free cutaneous nerve endings in the scalp, and cause muscle twitching by different mechanisms (see above) (Mancuso et al., 2023). The resulting EEG response is an event-related potential (ERP) (Ilmoniemi and Kicic, 2010), often termed a peripheral-evoked potential (PEP) in TMS-EEG studies (Fig. 2.1F). These are typically characterized by a negative deflection peaking at around 100–120 ms, predominantly observed in the fronto-central midline channels, followed by a positive deflection at approximately 180–200 ms (Ahn and Fröhlich, 2021, Biabani et al., 2019, Ilmoniemi and Kicic, 2010). This response, commonly referred to as the N100–P200 complex, can be elicited by various sensory stimuli, suggesting that it is largely independent of sensory modality, reflecting the processing of perceptual inputs and salient events (Downar et al., 2002, Kenemans, 2015, Mouraux and Iannetti, 2009, Singhal et al., 2002, Wessel and Aron, 2017). However, modality-specific potentials can also be observed as early as 20 ms after the TMS pulse, and these should be taken into account when designing TMS-EEG measurements (Holt and Ozdamar, 2016, Malcharek et al., 2011).

Handling PEPs is critical for the accurate interpretation of TMS-EEG signals. This is especially challenging because, like the signal of interest (TEPs), PEPs are time-locked to the TMS pulse and have a neuronal origin. Several offline procedures have been proposed to minimize the contribution of PEPs in TMS-EEG data, such as ICA, SSP-SIR, linear regression, and cosine similarity-based analyses (Belardinelli et al., 2019, Biabani et al., 2019, Freedberg et al., 2020, Raffin et al., 2020, Ross et al., 2022). However, approaches like ICA have been criticized for assuming 1) statistical independence and 2) linear interactions between TEPs and PEPs, assumptions which may not hold (Chowdhury et al., 2022).

An alternative approach to address auditory input involves masking the TMS click sound using ear defenders and noise, and preventing bone-conduction of the click by placing a layer of foam between the coil and scalp. This combination may prevent participants from perceiving the click, thereby preventing the consequent auditory ERP, at least within a certain range of stimulation intensities (Leodori et al., 2022, Massimini et al., 2005, Rocchi et al., 2021). Specifically, masking noise derived from the spectral characteristics of the TMS coil “click” has been shown to be particularly effective to prevent auditory evoked potentials in TMS-EEG measurements (Russo et al., 2022b, Trapp et al., 2024). Nevertheless, some studies have reported that the masking noise approach does not fully block the TMS click sound for all participants (Conde et al., 2019, Gordon et al., 2021), especially when the coil is placed close to the ear or over lateral scalp locations (Biabani et al., 2024, Fernandez et al., 2021, Fong et al., 2023). Furthermore, approaches which combine noise masking, foam, and a more predictable interstimulus interval result in a larger suppression of the N100/P200 complex than any approach applied individually, suggesting a combination of approaches is required for optimal PEP suppression (Ross et al., 2022). Foam padding increases the coil-to-cortex distance, and this may require the need for a disadvantageous increase in stimulation intensity (Ross et al., 2022). In addition, care should be taken that the foam does not lead to absorption of conductive gel from adjacent EEG electrodes and a consecutive increase in impedance (Hernandez-Pavon et al., 2023).

Despite the results highlighted above, no successful attempts to fully occlude the sensory input have been reported. A proposed solution is to use a control condition employing sham TMS, which mimics the sensory inputs of real TMS without directly stimulating the cortex (Ruohonen et al., 2000). A sham TMS condition that also generates a click sound can simulate the auditory input of real TMS, addressing the potential limitations of the masking noise approach. Similarly, applying electric stimulation with electrodes placed on the same scalp region as the TMS target can simulate the somatosensory input from the real TMS pulse (Conde et al., 2019, Mennemeier et al., 2009, Rocchi et al., 2021, Rossi et al., 2007), especially at supra-threshold stimulation intensities (Gordon et al., 2021, Raffin et al., 2020). However, it is uncertain whether PEPs elicited by electrical stimulation of the scalp are equivalent to those generated following somatosensory input by TMS. It has been proposed that it is possible to isolate the “true” TEPs by comparing the sham TMS response to the real TMS response (Gordon et al., 2021, Takano et al., 2021). This would require that the somatosensory and auditory responses evoked by the sham procedure closely match those of real TMS, as any differences in PEP amplitude could lead to misinterpretations of TEP components (Gordon et al., 2021, Siebner et al., 2019). Further, this proposition assumes that the peripheral evoked contribution to the TEP is stable and can be handled by a simple subtraction approach. This assumption is most likely incorrect, as the peripherally induced N100–P200 component of the TEP is strongly modulated by the predictability of the TMS. The N100–P200 component can be attenuated by making the timing of the TMS pulse predictable, presumably reflecting an active gating effect at the central level.

Another potential source of PEPs is the motor response observed when stimulating the primary motor cortex (M1) at suprathreshold intensities. The sensory input from muscle twitching involved in the TMS motor-evoked potential (MEP), known as motor reafference, may overlap with TEPs and generate cortical responses. It has been suggested that a potential observed around 60 ms after the TMS pulse may be attributed to motor reafference (Biabani et al., 2021, Fecchio et al., 2017, Paus et al., 2001, Petrichella et al., 2017). However, no procedure has yet been proposed to control for this potential confounder, aside from stimulating M1 at subthreshold intensities, which do not evoke an MEP.

Together, this body of work demonstrates the challenge of uncovering neural responses in EEG signals following TMS due to the wide range of potential artifacts. Ensuring high quality data collection methods and robust cleaning pipelines (see Section 3) are essential for maximizing the SNR of TMS-EEG data and developing reliable and valid TMS-EEG biomarkers for clinical applications.

## 3 TMS–EEG analysis

### 3.1 TMS-EEG metrics

The effect of TMS on the concurrent EEG can be evaluated in different ways (see also Section 5), depending on whether the interest lies in a single-pulse or a repetitive-stimulation protocol (Bergmann et al., 2021). In single-pulse studies, the typical measures of brain response to TMS are analogous to those used in other EEG studies that employ external stimuli. For example, just as a visual stimulus is evaluated by the visual evoked potential (VEP) or an auditory stimulus by the auditory evoked potential (AEP), the brain response to TMS is evaluated via the transcranial evoked potential (TEP) (Ilmoniemi et al., 1997).

An evoked potential is calculated by averaging trials that are time-locked to the stimulus. The number of trials should be sufficient to minimize random fluctuations in the EEG signal while preserving all activity that is time-locked to the stimulus, representing the activity evoked by the pulse (Dawson, 1954). In TMS–EEG studies, the evoked potentials can consist of a superimposition of both wanted and unwanted brain responses. These include the cortical responses to direct transcranial stimulation as well as those due to the concurrent activation of the brain caused by peripheral evoked potentials (PEPs) elicited by TMS, such as the loud click or somatosensory input (Conde et al., 2019). The averaged trials will show fluctuations in each channel with varying amplitudes at different time points following the pulse. These fluctuations are composed of a series of peaks which may represent the activation of one or more cortical sources. Since the voltage values in each channel vary over time, each of the peaks can be represented as a topographical map, which illustrates the time-specific spatial distribution of the cortical activity at the electrode level.

In the TMS–EEG literature, the overall EEG activity is often quantified in terms of the global mean field amplitude (GMFA) (Kähkönen et al., 2003, Lehmann and Skrandies, 1980) and the GMFP (e.g., (Bertazzoli et al., 2021)). These correspond to the standard deviation or variance across the channels, respectively, at each time point. They represent the global activation of the entire brain over time and can be used to identify peaks, as well as to highlight time points where two or more conditions differ. There are also derived indices used mainly in TMS–EEG studies, such as the local mean field amplitude or power (LMFA and LMFP, respectively) (Pellicciari et al., 2013), where a subgroup of adjacent electrodes is combined to represent the activity of a region of interest. When interpreting LMFA/LMFP values, special caution is required due to the bipolar distribution of EEG sources and the influence of volume conduction.

Another metric used in TMS–EEG field that is common to standard evoked protocols with EEG is the time–frequency analysis (T/F analysis). This method evaluates the changes over time in the frequency components of the signal for each channel (Rosanova et al., 2009). For this metric, the proper preprocessing steps are even more important, as single-trial artifacts have a stronger impact on the global measure.

If the T/F analysis is performed on the evoked part of the signal, it will show the decomposition of the TEP into its frequency components. If the evoked T/F signal is subtracted from the total estimation, it will reveal the induced T/F signal, which represents the power changes in different frequencies that are not phase-locked across trials (Pellicciari et al., 2017). T/F power results are dominated by low frequencies because of the typical 1/f characteristic of power in EEG recordings. Appropriate baseline normalization (e. g., expressing post-stimulus power as relative change to baseline) can improve identification and visualization of stimulus-induced effects in high frequencies bands. However, there is little consensus on when and how to perform baseline correction. Typically, this baseline correction is done after averaging the single trial power T/F results. However, this may lead to an overestimation of post-stimulus power, in particular in the case of noisy trials. This may be solved by single-trial normalization and by using the full trial, instead of only the pre-stimulus baseline (Grandchamp and Delorme, 2011). Another way to address this problem is normalization based on subtracting mean baseline power per frequency (Hu et al., 2014).

Moreover, T/F responses can be exploited in the phase domain to obtain several other indices, for instance, inter-trial coherence, phase locking value, and phase-lag index, which measure phase consistency (or synchronization) across trials at single channels (Pellicciari et al., 2017).

When evaluating the effects of a plasticity inducing protocol (Bergmann et al., 2021), the main interest lies in identifying EEG differences between the brain states before and after stimulation. This can be achieved by analyzing resting state data and/or the response to single TMS pulses. The latter follows the repeated analyses of TEPs, as described above. Resting-state data analysis is mainly based on changes in power across different frequency bands and in different EEG channels, applying the power spectral density estimation.

In some cases, the state of the brain can also be estimated during the rTMS intervention (Leodori et al., 2021). To this end, the measures listed above can be evaluated during the interstimulus or interburst intervals. This can be useful not only to monitor the efficacy of the protocol during the administration, but also for safety reasons. With the development of new stimulation devices and approaches, the latter point is becoming increasingly important (Jadidi et al., 2025, Sinisalo et al., 2024).

### 3.2 EEG preprocessing

Preprocessing is commonly a multi-step data cleaning procedure, where the EEG data is prepared for the actual analysis. The aim is to remove and correct erroneous parts of the signal, which would lead to biased analysis outcome, while preserving the essential brain-derived signals as intact as possible. Since the TMS–EEG data are affected by TMS-related artifacts that are not present in other EEG measurements, post-hoc preprocessing steps must take them into account. TMS-related artifacts can be divided into instrumental (non-physiological) and physiological artifacts (see Section 2).

After interpolating the TMS-pulse artifacts, TMS–EEG preprocessing typically follows several standard EEG-analysis steps, including Fourier filtering, channel/trial rejection, downsampling, epoching and baseline correction. Evoked EEG is epoched within desired time windows, such that time stamps in all epochs are uniform. Epochs can be averaged or studied at single trial level for using statistical metrics. Baseline signal, meaning the time window before the stimulus, is often set such that the mean signal amplitude before the stimulus is set to zero, corresponding to the assumption that deflections at rest are random. EEG data typically have slow drifts and fast-changing noise patterns from extra-cranial origins. High- and low-pass filters in time(−frequency)-domain are often used to remove the slow drifting patterns and the fast ripples, respectively. However, since TMS-induced artifacts span a wide range of frequencies, removing a specific frequency band may leave residual oscillations around the artifacts, creating artificially generated signals. This ‘ringing’ phenomenon can sometimes be mitigated by using less aggressive filters with longer transition bands, but it may not be entirely eliminated. Overall, one should carefully consider the need for low-pass filters and only apply them after the sporadic high-amplitude artifacts have been removed with interpolation or methods such as spatial filtering. For drift removal, an effective approach is to use robust detrending techniques that fit a trendline extending beyond the artifact time window (De Cheveigné and Arzounian, 2018).

The most popular methods to clean some of the TMS-related artifacts are ICA (Hernandez-Pavon et al., 2012, Korhonen et al., 2011, Rogasch et al., 2014) and signal-space-projection (SSP) (Mäki and Ilmoniemi, 2011), the latter of which is often combined with source-informed correction (SSP-SIR) (Mutanen et al., 2016). As opposed to temporal filters, these tools work by combining multichannel information across all channels at a single point of time. Such methods are referred to as spatial filters (Hernandez-Pavon et al., 2022). Importantly, to use spatial filters, we assume that EEG data at any instant of time are generated by superimposing a set of weighted spatial patterns (topographies) and that these patterns stay fixed (constant) over time. The time-varying change of the weights of the topographies describes the activations as a function of time. To use spatial filtering, one needs to define the topography of the artifact or a set of artifact patterns, if one topography is not enough to describe the TMS artifact signals. The topographies can be estimated in various ways: ICA, principal component analysis (PCA), or setting the topography based on a-priori hypothesis or knowledge. After defining the artifact topographies, to estimate the final spatial filter, one may use different approaches. Orthogonally projecting out all EEG within the artifact subspace is referred to as the signal-space projection (SSP) method, and it inevitably removes neural data simultaneously. To avoid the excessive signal removal resulting from out-projection one can use different ways to preserve the EEG data that correlates with the clean EEG and can thus be considered neural data that should be preserved. This neural EEG can be estimated using the physical modeling of cortical activity (lead-field matrix) or the data itself. The first approach is utilized by SSP-SIR and SOUND (Mutanen et al., 2018) methods, and the latter approach is used by ICA, data-driven Wiener filtering (Mutanen et al., 2018) and beamforming-based artifact removal (Metsomaa et al., 2024).

As compared to time-domain filters, the spatial filters do not provoke ringing artifacts, but some important pitfalls may remain. The application of ICA to TMS-evoked EEG may result in removing some neural signals simultaneously (Mutanen et al., 2024). It is also possible to dampen the EEG amplitudes by SSP, as it completely eliminates some data dimensions by definition, and by SSP-SIR or SOUND depending on the degree of chosen regularization, which leads to spatial smoothening of all remaining topographies, thus decreasing the spatial resolution (Mutanen et al., 2022).

Since all of the spatial filters rely on statistics, e.g., uncorrelatedness or independence of the artifacts, they require enough representative samples to produce robust cleaning outcomes. A word of warning: ICA works best for randomly occurring artifacts that are independent from TMS-evoked neural data but may excessively clean data in time windows where overlapping stimulus-locked artifacts exist (Atti et al., 2024, Metsomaa et al., 2014, Metsomaa et al., 2016, Mutanen et al., 2024). Epoched EEG responses may have parts where the data quality is too low for any data cleaning procedures, in which case one may choose to completely delete such signals. One can reject one or more channels, or all channels within epochs where outlier data are identified. Interpolation methods can be applied to reconstruct the missing data if needed. It is noteworthy that interpolation cannot introduce any additional information that is not already present in the remaining data.

TMS-provoked PEPs, including the somatosensory and auditory ones, are most often undesired elements in EEG signals. To measure PEP signals separately, the measurement protocol may include sham stimulation, with the final aim of removing the sham-evoked responses from TEPs. For the removal, a number of different methods have been proposed. Simple subtraction of PEPs from TEPs at each instant of time separately can be justified if one can argue that the magnitude of the PEP amplitudes is equal in both sham and TMS conditions (Song et al., 2024). Alternatively, linear regression has been proposed, such that best-fitted topographies from PEP to TEP condition are removed for each time instant from TEP data (Biabani et al., 2019). The above-discussed spatial filtering methods have also been used in various versions of ICA, SSP-SIR (Biabani et al., 2019) and beamforming (Metsomaa et al., 2024). In all these approaches, the PEP-derived topographies were regarded as representing artifacts, and were thus estimated from sham-evoked data with the aid of ICA or PCA, either by using entire time intervals of the responses or shorter moving time windows matched across the conditions. The main limitation is the assumption that the sham protocols can entirely mimic the real PEPs, with the danger of distorting the genuine TEPs.

When the study involves multiple conditions which are compared in the final analysis, one should preferably align the measured epochs across all conditions such that the entire preprocessing is performed simultaneously over all data if possible. This handling guarantees that preprocessing is not artificially introducing systematic differences between conditions, which could be later erroneously interpreted resulting from neurophysiological origins. For example, if spatial filters are estimated and applied separately for different conditions, this may unintentionally bring about a filter-derived deviation between the conditions.

Real-time EEG cleaning is becoming more relevant in practice as personalized closed-loop-driven TMS measurement and therapy protocols are emerging (Makkonen et al., 2021, Vetter et al., 2023, Zrenner et al., 2018). The requirement for real-time processing is to produce the outcome at such a speed that it still allows time for further analysis in a required time frame for delivering the stimuli within targeted time windows. The cleaning quality must be at a level that the decision making is accurate enough for the given application.

In practice, the spatial cleaning techniques are more appealing as opposed to temporal cleaning due to their lower computational cost, which only takes place over the number of channels as opposed to channels x time instants in the case of temporal filtering. All the above-mentioned filtering techniques are fitted to real-time EEG cleaning but it is noteworthy that the implementation via beamforming has proven highly efficient (Metsomaa et al., 2024). The slowest initial step for filter computation is to identify the artifact topographies. If the artifact topographies are well-defined, one may only redefine the spatial filter according to changing statistical properties in the data, as defined by the data or noise covariance matrices. Such updating of the filter can take place at a slower pace in a parallel process, as the information of the data statistics is increasing and varying (non-stationarity) during the measurement, whereas the filter application itself is a very fast process that exceeds the high sampling frequency of EEG (Makkonen et al., 2021).

Several open-source toolboxes have been developed specifically for TMS-EEG preprocessing which include many of the abovementioned methods (Hernandez-Pavon et al., 2023). Examples include TESA (Rogasch et al., 2017), TMSEEG (Atluri et al., 2016), ARTIST (Wu et al., 2018), and an extension to the FieldTrip toolbox (Herring et al., 2015). The development of standardized methods is important to enable the creation of reproducible preprocessing pipelines for clinical TMS-EEG applications (a performance comparison of different toolboxes on real TMS-EEG data can be found in (Brancaccio et al., 2024).

### 3.3 Statistical analysis of TMS–EEG data

In general, the ultimate goal of TMS–EEG data analysis is to identify and demonstrate differences between various conditions or cohorts. It is also common to search for patterns that explain relationships between TMS-elicited EEG responses and different functional processes. To this end, solid statistical approaches and appropriate analytical tools are essential. As in any scientific discipline, the statistical analysis of TMS–EEG data can be divided into exploratory and confirmatory approaches (Tukey, 1980): exploratory approaches aim to identify patterns or effects without strong prior hypotheses, when little is known about the data or when the goal is to generate new hypotheses. Confirmatory approaches are hypothesis-driven and designed to test specific, predefined questions.

TMS–EEG data is inherently multidimensional—typically involving dozens of channels, hundreds of time points, and, in the case of T/F analysis, multiple frequency bins. The confirmatory approach can be particularly useful for narrowing down this parameter space by focusing on specific regions of interest (ROIs), time windows, or frequency bands, when strong prior information is available (e.g., (Huber et al., 2013)). Once specific physiological readouts of interest, such as N15-P30 peak-to-peak amplitude over the stimulated channel region, are derived from the TMS–EEG data, standard parametric tests—such as t-tests for pairwise comparisons or ANOVA for multi-group comparisons—can be used to assess statistical differences between conditions. Note that when applying these types of parametric tests to TMS–EEG data, it is crucial to assess whether the key statistical assumptions, such as normality and homoscedasticity, are met. In many cases, transformations (e.g., log or Box-Cox) or normalization may be required (Osborne, 2002). To determine the need for such adjustments, data should be inspected using visualizations (e.g., histograms, quantile plots) and statistical tests for normality. If several metrics are tested simultaneously, common correction methods such as false discovery rate (FDR) and family-wise error rate (FWER) adjustments are used to control the risk of false positives (the so-called multiple comparison problem) (Ludbrook, 1998).

The advantage of confirmatory testing is that when significant effects are observed, they allow for strong inferences about the neurophysiological origin of these effects. However, the validity of these inferences depends on the specificity of the prior information and the legitimacy of the assumptions. Furthermore, the less specific our prior assumptions are, the more the FDR and FWER methods compromise the overall statistical power.

In cases where little or no prior information is available, exploratory cluster-based statistics are often used to identify potential differences across broad time, frequency, and spatial domains. Cluster-based permutation testing is a widely used method that addresses the multiple comparisons problem by identifying contiguous clusters of significant data points rather than evaluating each point independently (Maris and Oostenveld, 2007). Cluster-based approaches increase sensitivity to distributed effects, but they only allow researchers to conclude that a difference exists between groups or conditions (Sassenhagen and Draschkow, 2019). I.e., they do not permit precise inferences about the spatial or temporal location of the effect. To make more detailed claims, exploratory results should guide follow-up confirmatory tests conducted on independent datasets.

In addition to testing effects between groups or conditions, TMS–EEG researchers are often interested in correlations between TMS-elicited EEG responses and other functionally relevant metrics, such as motor-evoked potential (MEP) amplitude when targeting the primary motor cortex (M1) (Mäki and Ilmoniemi, 2010). The conventional approach involves isolating a simple readout from the multidimensional TMS–EEG data—for example, measuring the peak-to-peak values of early TEPs in a channel ROI—and performing standard correlation or regression analyses to identify statistically significant relationships between TEPs and the functional measure of interest (e.g., (Farzan et al., 2013, Rogasch et al., 2013a)). While less common, correlation analysis can also be combined with exploratory cluster statistics (Bracco et al., 2023), when strong priors on the location or timing of potential correlations are lacking. Another interesting approach, which is gaining attention in the TMS–EEG field, is the use of mixed-effects models (Desforges et al., 2022, Kaarre et al., 2018, Passera et al., 2022). These models can account for well-known individual and intertrial variability by treating them as random effects, thereby increasing the power to detect meaningful population-level relationships between TMS-elicited responses and the functional metrics of interest.

### 3.4 Machine learning / AI applied to EEG and TEPs

Machine learning (ML) is an emerging field of methodology that can benefit EEG analysis in conjunction with TMS in two-fold ways: (1) One may use them as exploratory methods to uncover relevant signal features, such as separation of pure TEPs from contamination by sensory input (Cristofari et al., 2023), or neurophysiological mechanisms that are related to a research question at hand. In this way, they may be considered as an alternative method for some statistical methods, like cluster-based statistics. (2) ML can be used as part of a closed-loop TMS-EEG approach to individually optimize TMS protocols using somewhat similar ideas as with brain-computer interfaces. In the latter application, one would train and update the ML model online, and after reaching an acceptable accuracy, the model would directly be applied in a real-time setting to adjust the TMS parameters (timing, site, frequency, intensity, etc.).

To date, few studies have employed ML with the purpose of predicting cortical excitability based on pre-stimulus EEG signals (Ermolova et al., 2024, Haxel et al., 2024, Metsomaa et al., 2021). MEP amplitude has been used as a measure of corticospinal excitability when stimulating M1, enabling the use of supervised learning algorithms. For binary classification of the single-pulse MEPs into classes ‘high’ or ‘low’, the employed ML methods include logistic regression, support-vector machine, random forest, and linear discriminant analysis. The EEG features used for predicting excitability have been phases of oscillations, spatio-temporal patterns, power and/or location of spatially travelling or fixed oscillatory patterns at various frequencies. Data-driven compression methods to extract the essential features, such as generalized eigenvalue decomposition, are often combined with ML methods to avoid overfitting. The risk of overfitting is also diminished by cross-validation, where trained models are tested against another set of data. Having defined an optimal EEG-based brain state, in real-time applications, it is important to be able to forecast EEG for delivering the TMS pulses at the optimal brain state. To that end, it has been demonstrated that neural networks, in the form of WaveNet, can outperform the linear auto-regression-based prediction of amplitude and phase when investigating theta and alpha band signals (Pankka et al., 2025).

To estimate a regression model linking the pre-stimulus EEG oscillation phase to MEP amplitude, the use of Bayesian optimization in non-parametric and parametric forms has also been proposed (Kirchhoff et al., 2024). Furthermore, Bayesian framework in the form of Gaussian process regression (GPR) has been deployed to set the optimal orientation of the TMS-induced E-field based on TEP deflection amplitude at a predefined latency and channel (Tervo et al., 2022). GPR is a flexible, non-parametric, and robust tool to optimize parameters that define an unknown function, which is known to change smoothly with varying parameter values. In the case of TMS–EEG, the unknown function could be any relevant EEG-derived measure, e.g., the power of defined induced oscillations.

ML-based models are optimally suited and often applied at the single-subject and −measurement level, which makes them ideal for studying (patho)physiological features that vary across individuals. These methods make use of single-trial-derived features, having low SNR and, therefore, they require a high number of trials for training and carefully designed preprocessing, as outlier trials are prone to cause overfitting. Statistical testing of the ML-based results, e.g., accuracy, of the obtained model is often straightforward to perform, but one should take care to test final accuracy on a separate never-seen-before test dataset, that has not been used in training or decision-making of the estimated ML model.

### 3.5 Source reconstruction of EEG sensor signals

Source reconstruction of EEG signals in the cortical source space can provide optimal spatial information about the cortical origin of the EEG signal pre- and post-TMS.

To estimate the subject’s head conductivity properties, a ‘forward’ solution is needed. This is a physical description of the way through which the modeled source dipoles, representing the neuronal cortical activity, contribute to the signal at the scalp EEG sensors, in a linear summation-defined “leadfield” (Fuchs et al., 2002). Most used solutions for the forward model are the Boundary Element Method (BEM) and the Finite Element Method (FEM).

An individual MRI scan is not strictly needed to successfully perform source localization. However, for the purpose of neurophysiological research, it is considered as best-practice to use individual head-models as segmented from MRI scans to source-extract the signal of interest. When a source solution is properly implemented to reconstruct EEG activity at the cortical level, a precise individual headmodel allows for a theoretical spatial precision of a few millimeters (Neugebauer et al., 2017, Troebinger et al., 2014). Furthermore, when available, the neuronavigation system can also be used to record the exact 3D positions of EEG electrodes relative to the individual MRI, thereby improving the accuracy of the forward model (Homölle and Oostenveld, 2019, Nielsen et al., 2023).

In the ‘inverse’ solution, an EEG deflection evoked by TMS or a specific oscillatory brain signal of interest can be extracted from sensor signals, by linearly combining the voltages recorded at each of the EEG sensors to reconstruct the neuronal cortical activity which generated the sensor signal (Mosher et al., 1999). Among the several inverse recipes, two have been routinely used with TMS–EEG: Minimum Norm Estimation (MNE) (Hämäläinen and Ilmoniemi, 1994) and Linear Constrained Minimum Variance (LCMV) Beamforming (Van Veen et al., 1997). Whereas the first is a proper inverse solution providing for an estimate of source activity for each time sample and each source location, the latter is a technique derived from radar science, scanning the source space voxel by voxel (or point by point) serially. This last property of the LCMV beamformer allows reconstructing activity exclusively from regions of interest, an advantage if computation time is an issue, as in real- time EEG-TMS.

## 4 Effects contributing to TMS-EEG variability /test–retest reliability

### 4.1 Variability and test–retest reliability: Why they are important in the clinical context

The development of diagnostic and prognostic markers in psychiatric and neurological brain disorders (Farzan, 2024) is one of the most widespread applications of TMS-EEG (see 10, 10.1, 10.2, 10.3, 11, 11.1, 11.2, 11.3, 12, 12.1, 12.2, 12.3, 12.4, 13, 13.1, 13.2, 13.3, 14, 14.1, 14.2, 14.3, 15, 15.1, 15.2, 15.3, 16, 16.1, 16.2, 16.3, 17, 17.1, 17.2, 17.3, 18, 18.1, 18.2, 18.3, 18.4, 18.5, 19, 19.1, 19.2, 19.3, 20, 20.1, 20.2, 20.3, 21, 21.1, 21.2, 21.3, 22, 22.1, 22.2, 22.3, 23, 23.1, 23.2, 23.3, 24, 24.1, 24.2, 24.3, 25, 25.1, 25.2, 25.3, 26). In the motor system, TMS-evoked myographic potentials have an established clinical diagnostic use, where the amplitude and latency of the muscle twitch serve as a reliable and repeatable marker of central signal propagation and corticospinal tract integrity (Groppa et al., 2012). TMS-evoked EEG potentials carry analogous information about the functional and structural integrity of cortical networks and circuits, beyond the motor system. However, it is much more difficult to extract similarly reliable diagnostic markers from the evoked EEG signal, in part because: (1) The post-stimulus EEG signal under the coil that could in principle be used analogously to the MEP for deriving hotspot and threshold information is contaminated by artifacts (Section 2); (2) EEG has a much higher dimensionality (e.g., 64 channels x 250 samples = 16,000 dimensions) than the EMG signal, and it is not clear how to best derive a scalar value from this frequently heavily pre-preprocessed data (Altman and Krzywinski, 2018); (3) The TMS-evoked response is superimposed on high-amplitude fluctuating ongoing dynamics that is captured by the EEG signal (Arieli et al., 1996), leading to a high degree of inter-trial variability even in the absence of artifacts and with standardized and optimized measurement (Parmigiani et al., 2025) and analysis procedures (Hernandez-Pavon et al., 2023).

The prospect of employing measures derived from TMS-EEG in clinical contexts as a diagnostic biomarker or for monitoring purposes calls for understanding their reliability and their potential sources of variability (Julkunen et al., 2022). Indeed, reliability is a critical component in any biomarker development as it indicates the consistency of a measure across time, conditions, and raters. A measure that is not reliable cannot be used to diagnose or monitor disease progression, as the measurement is inconsistent.

The concept of reliability is strictly linked to the concept of variability. While it might seem intuitive to assume that greater variability simply reduces reliability, the relationship between the two is more complex and may depend on the variability that is being considered (Miniussi and Bortoletto, 2025). Therefore, in this section we will review inter-area, inter-individual, and methodological variability, and highlight how each of them relates to reliability. It is important to understand if there are factors or procedures that reduce reliability and make TMS-EEG measures changeable in unchanging conditions. By systematically identifying and quantifying these factors, researchers can refine protocols, standardize procedures, or statistically adjust for confounders, ultimately improving the reliability of the measure.

### 4.2 Test-retest reliability of TMS-EEG

Test-retest reliability is a crucial feature in developing TMS–EEG-derived biomarkers. It has been a focus of investigation since early studies (Casarotto et al., 2010, Lioumis et al., 2009) and continues to be of great interest (Gogulski et al., 2024, Kerwin et al., 2018). Our current understanding of the two subtypes of test–retest reliability, i.e., the degree to which individuals in a sample maintain their position relative to each other with repeated measurements (relative reliability) and the degree to which repeated measurements vary within a sample of stable individuals (absolute reliability) (Beaulieu et al., 2017, Mokkink et al., 2010) was recently reported for TMS-EEG in a systematic review (Bertazzoli et al., 2025): while most efforts have been directed at examining the relative test–retest reliability of TEPs, several equally important dimensions remain largely unexplored. These include the absolute reliability of TEPs, and both relative and absolute test–retest reliability of TMS–EEG-derived measures beyond TEPs, including TMS-induced oscillations.

The relative reliability of TEPs has been measured mainly with the intraclass correlation coefficient (ICC) or with the concordance correlation coefficient (CCC; (Lin, 1989) on several cortical targets, including the primary motor cortex, prefrontal, and parietal regions. These studies show that later components occurring from 80 ms onwards have high relative reliability, while early components have shown lower reliability than late components.

When CCC and ICC were calculated, studies showed variable test–retest reliability for peaks and latencies of TEPs, with higher values for late components than for early components (Bertazzoli et al., 2021, Kerwin et al., 2018). Kerwin et al. (2018) found that late components—particularly N100 and P200—were consistently reliable, especially in central and centroparietal regions. Early components, including the N40 (or N45) and P60, however, produced more variable results. Bertazzoli et al. (2021) also used CCC to test the reliability of TEPs in both the DLPFC and inferior parietal lobule. Their findings echoed those of Kerwin et al. (2018), showing a clear trend: reliability improves from early to late components, with N100 and P200 again standing out for their robustness. These results align with early findings on TEP peaks based on correlations (Lioumis et al., 2009). Other research focused on the spatial and temporal correlations of TEPs between sessions. These analyses compared either the distribution of activity across electrodes at given time points (spatial) or the signal's time course at each electrode (temporal). Across the board, late components consistently demonstrated stronger and more stable correlations than early ones (Bertazzoli et al., 2021, Kerwin et al., 2018, Ozdemir et al., 2021b).

The reason why early components are less reliable than late components is unclear. As described below, a possible explanation is related to the presence of different artifacts throughout the time window of TEPs. Specifically, early components are prone to muscle and decay artifacts, which may be more variable (Gogulski et al., 2024, Lioumis and Rosanova, 2022). Late components are prone to sensory artifacts, which may be more consistent. The study by Song et al. assessed the test–retest reliability of TEPs in spatial and temporal domains across repeated sessions for TMS targets (angular gyrus – AG, supplementary motor area – SMA, and medial prefrontal cortex – mPFC) (Song et al., 2024). High spatial CCCs (*>*0.8) were observed from 90 ms onward in both active and sham conditions. After removing peripheral evoked potentials (PEPs), i.e., auditory-evoked potentials (AEPs) and the somatosensory-evoked potentials (SEPs) generated by the TMS, the reliability of “cleaned” TEPs decreased. For AG, spatial CCCs remained fair to moderate (0.4–0.67) until 190 ms, with significant CCCs up to 150 ms. SMA showed reliable spatial CCCs until 80 ms (0.2–0.6), while mPFC had lower CCCs, with spatial reliability until 80 ms and generally low temporal reliability.

In summary, these studies suggest that relative TEP reliability is influenced by the reliability of concurrent artifacts, and the presence of sensory artifacts in late components of the TEP may undermine their validity (Nikouline et al., 1999).

### 4.3 Variability: what is it and what affects it

#### 4.3.1 Inter-area variability

Cortical regions exhibit distinct TEPs (Freedberg et al., 2020, Harquel et al., 2016, Noda et al., 2021, Song et al., 2024). For instance, TMS over M1 generates a fast, robust TEP with early peaks (∼15 and 30 ms, see (Bortoletto et al., 2021, Zazio et al., 2022) and even earlier peaks, see (Beck et al., 2024a, Stango et al., 2025) and well-defined later components such as the N100 (∼100 ms) (Bonato et al., 2006, Komssi et al., 2002, Lucarelli et al., 2025, Nikulin et al., 2003, Ozdemir et al., 2021b, Paus et al., 2001, Rogasch et al., 2014). Responses are relatively consistent across subjects, making M1 a suitable benchmark for TMS-EEG studies. However, the direct translation of knowledge acquired from M1 TEPs to remote or “silent” brain regions, regions without measurable behavioral output, appeared to decrease the external reliability and validity of these TMS-EEG markers (Parmigiani et al., 2023). For instance, another well-explored region in the TMS-EEG field is the dorsolateral prefrontal cortex (DLPFC), in which TMS produces more variable TEPs (Gogulski et al., 2024, Kähkönen et al., 2005b, Niu et al., 2024, Song et al., 2024), with multiple peaks (∼50 ms, ∼100 ms, ∼200 ms) reflecting complex connectivity and subject to various methodological confounds (e.g., large and inconsistent TMS-induced artifact (Gogulski et al., 2024)). Importantly, despite its ambiguous origin, the amplitude of the N100 over the DLPFC is increasingly used in research as a predictive biomarker in major depression (Sheen et al., 2024, Sun et al., 2016, Sun et al., 2024, Voineskos et al., 2021, Voineskos et al., 2019) (Sheen et al., 2024, Sun et al., 2016, Voineskos et al., 2021, Voineskos et al., 2019).

More generally, previous studies have reported distinct and complex shapes of biological input–output functions between stimulation sites: EEG components display various mixtures of linear and nonlinear relationships with increasing stimulation intensities at different cortical targets (Komssi et al., 2004, Raffin et al., 2020, Zazio et al., 2019). These results suggest different dynamical properties of neuronal responses reflecting regional differences in cortical microcircuits. This highlights the need for more systematic evaluation and comparison of the TMS-EEG features throughout the brain (Harquel et al., 2016). In particular, stimulating more lateral brain regions elicits strong early muscle artifacts that can interfere with TEP interpretation (Gosseries et al., 2015, Mutanen et al., 2013b, Passera et al., 2023). Additionally, the peripheral auditory click and somatosensory perception produced by a TMS pulse evoke non-specific sensory responses (Biabani et al., 2019, Conde et al., 2019, Ross et al., 2022) that can substantially vary from one stimulation site to another (Gogulski et al., 2024). Furthermore, in a preprint study, Sayalı and colleagues compared the test–retest reliability of TEPs across two cortical regions (DLPFC and angular gyrus) in comparison to M1. The authors reported that TEPs evoked from M1 showed the highest reliability while DLPFC and angular gyrus showed lower and comparable reliability (Sayalı et al., 2024) (but see (Ozdemir et al., 2021b)).

Moving away from evoked potentials, spectral features have been shown to follow a rostro‐caudal gradient in their main oscillatory frequency (i.e., natural frequencies) (Rosanova et al., 2009). While each separate cortical region appears to have its own dynamical signature, cortical areas encompass a specific mixture of oscillatory signals (Fecchio et al., 2017, Raffin et al., 2020).

These regional differences in both TEPs and oscillatory responses can be attributed to multiple biological factors. Cytoarchitecture seems to strongly affect how neuronal populations respond to TMS (Lioumis et al., 2025). More precisely, layer composition and neuronal density will act on the reliability of TEPs. For instance, M1 has a thick layer V, densely packed with large pyramidal neurons (Betz cells) (Nolan et al., 2024), which may contribute to strong and reliable TEPs. In contrast, the prefrontal cortex has a more complex granular structure, with fewer large pyramidal neurons and denser local inhibitory circuits (Carlén, 2017), that may lead to more variable TEPs.

Other important factors are myelination and conduction properties, which significantly differ across the cortex. More myelinated regions (e.g., motor cortex, primary sensory areas) exhibit faster and more synchronized responses, while less myelinated areas, such as the association cortices, may show prolonged or dispersed potentials due to slower conduction and integration delays (Nunez et al., 2015, Schmidt and Knösche, 2019, Tomasevic et al., 2022).

The excitability of a given cortical region also depends on the unique balance between excitatory and inhibitory neurotransmission. Again, contrasting M1 versus the prefrontal cortex, variability may be partly explained by the difference in glutamatergic activity. Regions with high excitatory drive (e.g., M1, but also the primary visual cortex) generate strong, consistent TEPs. By opposition, higher inhibitory tone, where GABAergic inhibition is dominant or in regions in which additional types of interneurons might be enriched in the local microcircuits, that inhibit or excite other interneurons, can result in delayed TEPs or in their suppression. Regional structural and functional connectivity patterns may drive some of the variability observed between stimulated sites. Highly interconnected areas (e.g., M1, somatosensory cortex) show strong local excitatory loops but also connections with recurrent large-scale networks, reinforcing consistent TMS-evoked activity, while less interconnected regions (e.g., higher-order association cortices) may exhibit less synchronized responses (Momi et al., 2023). Furthermore, it is thought that regions with strong reciprocal connections to subcortical structures (e.g., thalamus, basal ganglia) exhibit complex, delayed, or polyphasic TEPs. In summary, regional differences in TMS-evoked potentials arise from complex interactions between cytoarchitecture, myelination patterns, excitatory-inhibitory balance, and connectivity profiles, with highly interconnected regions producing more robust responses and regions with strong reciprocal subcortical connections exhibiting more complex waveforms.

#### 4.3.2 Interindividual variability

The vast majority of studies to date have characterized the TMS-EEG responses by averaging measurements from groups of subjects, therefore providing population-level inferences. However, it has been shown that differences in TEP topography between individuals at a specific time point highlight variability in the spatial organization of activated neural sources (Michel and Murray, 2012). Meanwhile, individual differences in the temporal progression of these source-localized TEPs demonstrate distinct propagation patterns across various brain regions. High inter-individual variability is a well-recognized and widely reported issue for measures obtained with EEG. Consequently, inferences made at the group level may not necessarily hold at the level of individual trials or subjects (Bridwell et al., 2018).

Some studies have quantified how much individual TEPs differed from the group-average TEP (e.g., (Ozdemir et al., 2021b)). The conclusions are that TMS-evoked responses are highly reproducible at the group level but, in most cases, individual responses are clearly different from the group response and highly heterogeneous across subjects (Casarotto et al., 2010, Menardi et al., 2022, Momi et al., 2023, Ozdemir et al., 2021b, Raffin et al., 2020).

Some TMS-EEG features are more sensitive than others to interindividual variability (Harita et al., 2022). In the time domain, some group-level inferred components resulting from TMS of M1 (especially early peaks like N15/P15, P30, related to direct cortical excitation) are thought to be more stable, whereas later components (e.g., P60, N100, P200, thought to be linked to inhibition and cognitive processing) are supposed to be more variable across individuals (Casarotto et al., 2010, Kerwin et al., 2018, Komssi and Kähkönen, 2006, Lioumis et al., 2009). Indeed, early responses can be detected at individual level (Guidali et al., 2023, Zazio et al., 2022), and i-TEPs are visible both at individual and at single trial level (Stango et al., 2025). This may partly explain the lower relative reliability of early components and the higher relative reliability of late components (see above). Indeed, inter-subject variability, as a factor in the formula to calculate the ICC, modulates the relative reliability so that the higher the inter-subject variability, the higher the relative reliability.

Several studies have used multimodal brain imaging, such as fMRI, DWI/DTI, MR spectroscopy, and structural MRI, to explain inter-individual variability in TMS-EEG features. These studies aim to relate TMS-EEG responses to underlying structural and functional brain characteristics, helping to interpret why individuals differ in their responses (Momi et al., 2021a, Paparella et al., 2025). This also applies to clinical populations in which GABAergic system activity may predict EEG responses to TMS throughout recovery in post-stroke patients (Harquel et al., 2024, Paparella et al., 2023, Tscherpel et al., 2024) (cf. Section 20). Of note, a recent study suggests that the multimodal results gathered from structural MRI, resting-state fMRI and TMS-EEG might capture distinct neuroarchitecture profiles, indicating that TMS-EEG mapping reveals unique information about signal propagation (Sun et al., 2024).

Biological factors, including neuroanatomical and neurophysiological factors mentioned above, as well as age (Guerra et al., 2021, Kallioniemi et al., 2022) (Section 8), genetic, state-dependent, and methodological factors (Janssens and Sack, 2021) also affect inter-individual differences in TMS-EEG responses. Although there is not much literature examining the role of these factors specifically for TMS-EEG, it is likely that many factors known to drive inter-individual variability in response to non-invasive brain stimulation (Guerra et al., 2020, Lopez-Alonso et al., 2014, Ridding and Ziemann, 2010, Siebner et al., 2022) also apply to TMS-EEG.

Thus, there is an obvious need to account for inter-individual variability in TMS-EEG to avoid misinterpretation. While adopting standard procedures for TMS-EEG data acquisition (Hernandez-Pavon et al., 2023) or data analyses (Brancaccio et al., 2024) (Section 3) might reduce such variability, more complex analytic tools can also be implemented to account for the large inter-individual variability in evoked neural responses (e.g., Raffin et al., 2020), e.g. by applying an adaptation of Group Task-Related Component Analysis (gTRCA), a novel multivariate signal decomposition method (Couto et al., 2025). As this inter-subject variability cannot always be controlled or eliminated, a rigorous approach is to model it explicitly, treating it as factor, allowing interactions among individual characteristics, and identifying the features that influence or predict responses (Miniussi and Bortoletto, 2025). Alternatively, as highlighted above, differences in TEPs across individuals may also reflect genuine neurophysiological or cognitive traits (e.g., working memory capacity, attention, neurotransmitters’ activity) that are important to capture. Reporting individual TEPs and investigating their inter-individual variability will be important in the development of biomarkers for psychiatric and neurological disorders or to better understand cognitive functions.

#### 4.3.3 Methodological variability

One of the major challenges in evaluating TMS-EEG responses lies in the high variability in methodology across studies. Variability in methodological choices is a well-known issue in complex techniques that generate multidimensional data, such as fMRI, MEG, and EEG (Botvinik-Nezer et al., 2020), and TMS-EEG is no exception. Differences in both data acquisition and processing pipelines contribute to inconsistencies across studies. For instance, the choice of stimulation parameters, such as pulse waveform and coil orientation, impacts the amplitude and latency of TEP components as shown in M1 (Bonato et al., 2006, Casula et al., 2018b, Guidali et al., 2023) in pre-SMA (Casula et al., 2022a, Tervo et al., 2022) and in DLPFC (Gogulski et al., 2024). It also impacts the spatial distribution of activated sources (Lucarelli et al., 2025). Similarly, the choice of the signal processing pipeline can markedly alter the shape and amplitude of TEPs (Bertazzoli et al., 2021), even by changing a single step in the pipeline (Rogasch et al., 2022) (Section 3). In contrast, few reports show consistent TEPs regardless of the preprocessing pipeline used (Mancuso et al., 2024, Mancuso et al., 2021). The procedure of systematic mapping may help avoiding or reducing artifacts in the recording phase and checking the amplitude of early TEP responses before recording (Casarotto et al., 2022, Lioumis and Rosanova, 2022, Parmigiani et al., 2025). This variability affects the spatio-temporal features of TMS-EEG responses and the comparability of findings across research groups.

Beck and colleagues recently investigated the impact of methodology on TEPs measured after M1 stimulation (Beck et al., 2024b). This study provides a first estimation of the methodological variability across studies. Moreover, it highlights some of the issues related to methodological variability. Indeed, while the typical TEP components can be retrieved across studies, several variables, such as TMS intensity, the use of noise masking, and ICA, introduce systematic differences in the amplitude of TEP components. This poses a substantial obstacle to progress in the field, adding methodological noise that compromises data interpretation and hampers advancement.

The lack of ground truth to establish which methodological approach may result in “true TEPs”, i.e., an artifact-free cortical activation induced by the TMS electromagnetic field (Farzan and Bortoletto, 2022), increases the difficulty in interpreting differences across studies. On this premise, a recent study (Brancaccio et al., 2024) reported the comparison of TEPs obtained by processing a known synthetic TMS-EEG signal with different pipelines and showed their effects on the spatio-temporal features of TEPs and trial-by-trial variability. Nevertheless, a more systematic investigation, as proposed by the Team for TMS-EEG (T4TE) initiative (Bortoletto et al., 2023) is needed.

### 4.4 Effect of methodological variability on test–retest reliability

The impact of methodological variability across laboratories and studies on the reliability of TMS-EEG-derived measures remains largely understudied. However, understanding this variability is crucial for the development of new biomarkers, given the lack of standardization in TMS-EEG protocols and the significant differences in methodological parameters across cortical targets and research groups (Beck et al., 2024b). A few studies have started to reveal how these choices affect the reliability of the TEPs. For example, Gogulski and coworkers stimulated different DLPFC targets commonly used for rTMS and TMS–EEG studies and found that more medial and posterior DLPFC locations produced more reliable responses (Gogulski et al., 2024). Kerwin and colleagues demonstrated that at least 60 pulses should be delivered to achieve minimum reliability standards (Kerwin et al., 2018). Bertazzoli et al. applied four published pipelines on the same dataset and showed that the test–retest reliability of TEPs obtained in two separate sessions depended on the preprocessing pipeline (Bertazzoli et al., 2021). Therefore, both the quality of measurements as well as the efficacy of preprocessing procedures can affect reliability.

Methodological choices impact on the signal-to-noise ratio (SNR), which in turn is a crucial factor for the reliability of TMS-EEG measures. As previously explained, methodological choices that are highly efficient in reducing all artifacts present in TMS-EEG recording not only allow to obtain valid measures of cortical excitability and connectivity but also allow to reveal the reliability of these measures. On the contrary, methodological procedures that leave a substantial artifactual component in the signal will result in unprecise estimates of reliability that are affected by the reliability of artifact themselves. This may lead to the paradoxical situation that artifactual contaminated TMS-EEG measures may present higher reliability than clean data. The future development of shared and efficient methodologies for dealing with artifacts will be important both to obtain valid measures and also to understand their reliability.

## 5 TMS-EEG metrics as physiological readouts

### 5.1 Introduction

When applied over M1, a single TMS pulse induces local depolarization of axonal membranes, triggering synchronous action potentials. This neural volley is rapidly conducted along established anatomical pathways, such as the corticospinal tract, resulting in the generation of D- and I-waves (Di Lazzaro et al., 2004). The functional readout of this activation is the MEP, which can be recorded by combining TMS with electromyography (TMS-EMG). Key features of the MEP, such as its amplitude and latency, are considered physiological markers of corticospinal excitability. For instance, changes in MEP amplitude or latency can reflect pathway-specific plastic modifications or the presence of pathological conditions (Vucic et al., 2023).

A similar principle applies to the transsynaptic activation of local neuronal populations and interconnected circuits, including cortico-cortical and cortico-subcortical pathways, also when TMS is delivered over non-motor cortical areas. The activity evoked by TMS and its reverberation across these networks can be captured by TMS-EEG. This approach enables the direct, non-invasive probing of cortical circuits *in vivo*. Over the past decade, several TMS-evoked EEG metrics have emerged as candidate physiological readouts of cortical and cortico-subcortical excitability, connectivity, and response complexity. These include TEPs and their components, as well as derived measures such as TMS-related oscillations, connectivity metrics, and complexity estimates like the Perturbational Complexity Index (PCI).

This section provides systematic definitions of these key metrics, outlines the methodological principles underlying their extraction, discusses their neurophysiological correlates, and presents clinical examples illustrating their diagnostic value.

### 5.2 TMS-evoked potentials (TEPs) and TEPs components

#### 5.2.1 Definition and measurement

**Fig. 5.1:**
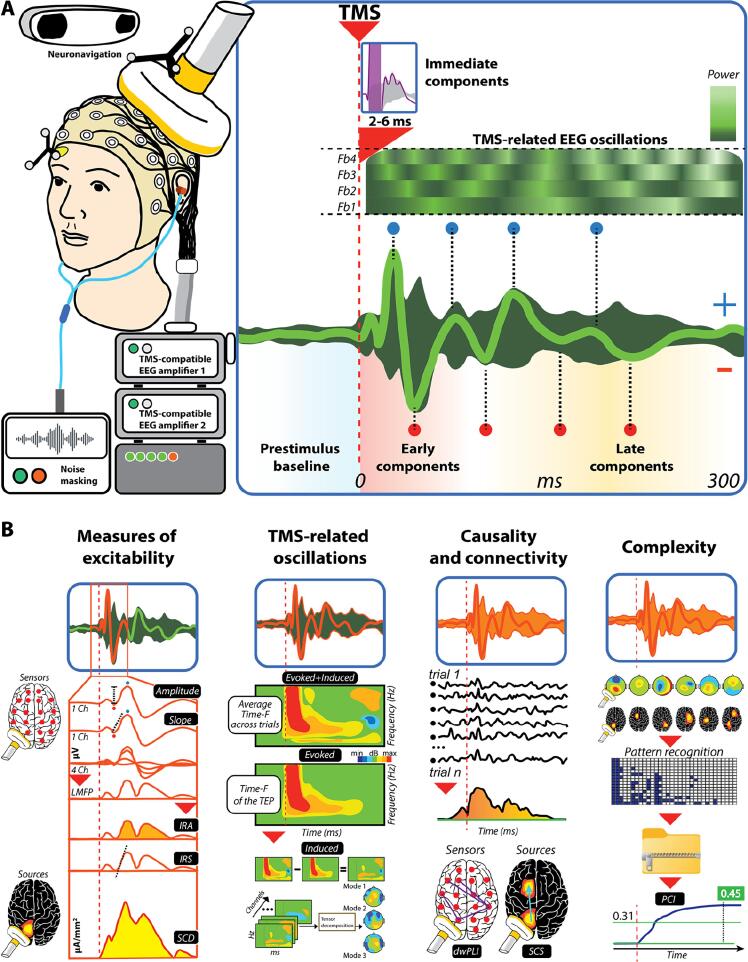
TMS metrics as physiological readouts. A (left) shows a schematic representation of the main elements used to acquire TMS–EEG signals: a neuronavigated TMS coil, an EEG cap with TMS-compatible electrodes designed to reduce eddy currents, and TMS-compatible ergonomic earplugs delivering noise masking. A (right) displays an example of TEPs recorded from the posterior parietal cortex (modified from (Rosanova et al., 2009), shown for one channel (thick light-green trace) and across all channels as a butterfly plot (dark-green shaded area). Positive (blue circles) and negative (red circles) peaks corresponding to TEP components are marked. Background colors indicate the prestimulus baseline (light blue), early components (light red), and late components (light yellow). A symbolic representation of oscillatory activity in four frequency bands (Fb1–Fb4, increasing in frequency) is shown as rectangles with alternating light and dark green shading. The inset illustrates immediate TEP components recorded over M1 (modified from (Beck et al., 2024a)). B (left to right) illustrates: Measures of excitability—examples include the peak-to-peak amplitude and slope of the first TEP component at the single-channel level. Similar measures can be derived from the Local Mean Field Power (LMFP) within the first 50 ms, such as the Immediate Response Area (IRA) and Immediate Response Slope (IRS) (modified from (Casarotto et al., 2019)). Cortical excitability can also be estimated at the source level using indices such as the Source Current Density (SCD; modified from (Casali et al., 2010)). TMS-related oscillations—schematic representation of methods used to extract evoked and induced TMS-related oscillations (time–frequency plots modified from (Rosanova et al., 2009); pipeline modified from (Harquel et al., 2025)). Causality and connectivity—examples of local causality measures (Phase Locking Factor, PLF) and connectivity indices computed at the sensor level (directed Weighted Phase Lag Index, dWPLI) and at the source level (Source Current Scattering, SCS; modified from (Casali et al., 2010)). Complexity—schematic representation of the main steps to compute the perturbational complexity index (PCI) (modified from (Casali et al., 2013) and (Rosanova et al., 2018)). (For interpretation of the references to colour in this figure legend, the reader is referred to the web version of this article.)

TEPs are sequences of positive and negative voltage deflections in the EEG signal, time-locked to the TMS pulse, and can extend over several hundred milliseconds. These deflections—referred to as TEP waves or components—are typically labeled according to their polarity (N for negative, P for positive) and latency in milliseconds (Fig. 5.1A). TEPs represent a special class of EEG-evoked potentials, and, as with classical sensory evoked potentials, they typically require the delivery of tens to hundreds of TMS pulses for averaging. The EEG signal surrounding each pulse is segmented into epochs and averaged across trials. This averaging attenuates EEG activity that is not time-locked to the stimulus, allowing stimulus-locked activity to emerge in the form of TEP components, which significantly deviate from noise and pre-stimulus ongoing activity (Ilmoniemi et al., 2024), ultimately resulting in a high signal-to-noise ratio. However, unlike sensory evoked potentials, TMS can directly activate the cerebral cortex using a wide range of stimulation parameters, including coil position, orientation, and the intensity of the induced electric field.

When targeting M1, stimulation effectiveness is first guided by relevant anatomical landmarks and then fine-tuned until a MEP is reliably recorded from the selected target muscle. In contrast, when targeting cortical areas outside M1, anatomical landmarks may be less apparent, and immediate physiological readouts such as the MEP are not available. As a result, it becomes more challenging to verify whether TMS is effectively activating the target cortical circuits.

To address this issue, researchers have developed freeware tools to search for optimal stimulation parameters—such as intensity and coil orientation—in real time, with the aim of ensuring a high signal-to-noise ratio of early TEP components (<50 ms) under the TMS coil (Casarotto et al., 2022, Hassan et al., 2022, Parmigiani et al., 2025, Tervo et al., 2022). Notably, only by increasing stimulation intensity to result in an induced electric field strength above ∼100 V/m it is possible to obtain a local activation that results in TEPs with high signal-to-noise ratio (Casarotto et al., 2022, Raffin et al., 2020). Finally, employing a neuronavigation system can further increase the accuracy in targeting the cortical area of interest and improve the selection and stability of stimulation parameters across trials and TMS–EEG sessions (Lioumis and Rosanova, 2022) (Section 27: Protocols how to measure TMS-EEG responses).

The components elicited by stimulating M1 are among the best characterized, and include the N15, P30, N45, P60, N100, and P180 (Beck et al., 2024b, Farzan and Bortoletto, 2022, Lioumis et al., 2009). Another frequently targeted region is the dorsolateral prefrontal cortex (DLPFC). TEPs evoked by single-pulse TMS of this area display components labeled P25, N40, P60, N100, and P180 (Farzan and Bortoletto, 2022, Lioumis et al., 2009), with early components peaking at different latencies and expressing different scalp topographies compared to those observed following M1 stimulation. This suggests that, particularly at early latencies, TEP components—and consequently TEP waveforms—may reflect target-specific cortical dynamics.

Late components may also show some degree of target specificity, provided that effective procedures for minimizing biological confounds are applied (Belardinelli et al., 2019, D'Ambrosio et al., 2022, De Martino et al., 2023, Fecchio et al., 2017, Ferrarelli et al., 2012, Rosanova et al., 2009) (Section 2).

Recent evidence suggests that TEPs also contain the immediate cortical response to TMS (Beck et al., 2024a). These so-called immediate TEPs (i-TEPs) can be recorded as early as 2 ms after TMS of the M1, when EEG amplifiers with very high sampling rates are used and scalp muscle activation is avoided by real-time EEG visualization. The M1 i-TEPs contain high frequency peaks approx. 1.1–1.5 ms apart (600–800 Hz) that resemble the frequency of the peaks of the indirect waves (I-waves) that can be observed in epidural recordings after stimulation of the M1. Moreover, this fast component consistently correlates with the amplitude of MEPs (Stango et al., 2025). Finally, i-TEPs are elicited when stimulating over pericentral areas and become smaller when linearly mapping the cortex across the rostro-caudal axis (Nuyts et al., 2025). While this may suggest that i-TEPs are specific for sensorimotor regions, it remains to be determined whether responses at comparably short latencies can be generated from other cortical areas.

#### 5.2.2 Neural underpinnings

Although the precise neural underpinnings of TEP components remain unclear, growing evidence in recent years has begun to shed light on their nature, which will be briefly reviewed below.

i-TEPs are affected by changes in the direction of the induced biphasic currents, similar to I-waves evoked by TMS, suggesting that i-TEPs are underpinned by the local activation of intracortical circuits underneath the TMS coil (Ziemann, 2020). Indeed, intracellular recordings in the primary somatosensory cortex of rats, and single- and multi-unit recordings in M1 of non-human primates and rodents have shown that neurons can fire action potentials within the first 5  ms after a TMS pulse (Li et al., 2017, Mueller et al., 2014), providing the substrate for immediate local synaptic activation and hence I-waves, and i-TEPs.

Early TEP components are also likely to reflect the activity of local cortical circuits directly activated by the TMS pulse, as supported by animal and modeling studies. Studies conducted in rats using extracellular multiunit recordings showed that single-pulse TMS applied over rodent’s equivalent to the primate M1 evoked action potentials underneath the TMS coil in the first 50 ms after the pulse with a peak around 20 ms (Li et al., 2017). Similar observations were made in non-human primates: targeting M1 (Mueller et al., 2014) and the posterior parietal cortex (Romero et al., 2019) led to sustained firing of cortical neurons under the coil between 20 and 40  ms after the TMS pulse. An earlier study in anesthetized cats using single-unit recordings during TMS of the primary visual cortex showed that neurons underneath the coil exhibited a significant increase in firing rate within the first 100  ms after the pulse (Moliadze et al., 2003). At the mesoscale level, a recent study combining TMS with intracranial recordings in non-human primates showed that the early components of TMS-evoked local field potentials are highly target-specific. Systematic changes in the TMS target location led to corresponding changes in the pattern of early responses recorded by the intracranial electrodes (Perera et al., 2023). Overall, these observations suggest that early TEP components are underpinned by the firing of local cortical neurons in the targeted region. It is also conceivable that this early neuronal firing engages local synaptic circuits, contributing to early TEP components through the synchronous spatial and temporal summation of excitatory and inhibitory postsynaptic potentials (EPSPs and IPSPs). In this regard, little is known about the action of TMS at the cellular level and, consequently, about the contribution of different intracellular events to TEPs. An intriguing possibility is that early TEPs may reflect dendritic synaptic and suprathreshold events—such as Ca^2+^ spikes—which can be detected by surface electrodes (Suzuki and Larkum, 2017). Notably, TMS may also indirectly activate inhibitory interneurons that target apical dendrites, leading to the suppression of dendritic Ca^2+^ spikes (Murphy et al., 2016).

Cortical neurons located underneath the TMS coil exhibit a facilitation of firing activity that can persist for up to 500 ms (Moliadze et al., 2003), suggesting that late TEP components, like early ones, are at least partially shaped by local circuit dynamics. However, a recent multi-scale study of premotor TMS responses—integrating TMS-EEG, intracranial recordings from both humans and rodents, and computational modeling—indicates that these late components may also reflect the engagement of subcortical circuits (Russo et al., 2025a). The study shows that thalamic rebound activity provides a major contribution to TEP components centered around 200  ms following TMS over premotor areas, and that their amplitude is strongly modulated by motor tasks that influence the state of thalamocortical neurons.

#### 5.2.3 Physiological and clinical relevance

Overall, the evidence indicates that early TEP components mainly reflect local reverberant activity within the targeted cortical region and can therefore serve as reliable readouts of local neural excitability. However, given the typical latencies of even the early TEPs, cortico-cortical, cortico-thalamo-cortical, cortico-cerebellar, and, in the case of M1, even cortico-muscular loops may also contribute to the local expression of early TEP components at the stimulation site. These components, typically occurring within 10–50 ms after the TMS pulse, can be quantified using their slope, amplitude, or the area under the curve of local power within the relevant time window at sensors level or synthetic indices such as the Source Current Density (SCD) at sources level (Fig. 5.1B). This approach provides a robust measure of cortical excitability and plasticity, closely mirroring methods used in animal studies of cortical plasticity (Bliss and Lomo, 1973, Vyazovskiy et al., 2008)) and has enabled the detection of plastic changes in human cortical circuits. For example, changes in the slope and amplitude of early TEP components reveal a progressive increase in excitability with time spent awake in healthy subjects, along with strong modulation by circadian rhythms (Huber et al., 2013, Ly et al., 2016). Moreover, modulation of early TEP components can indicate the effects of non-invasive brain stimulation techniques known to alter neuronal excitability, such as repetitive TMS (rTMS) (Esser et al., 2006), transcranial direct current stimulation (tDCS) (Pellicciari et al., 2013, Romero Lauro et al., 2014), and transcranial static magnetic field stimulation (Shibata et al., 2024), or reveal neural changes produced by attention (Herring et al., 2015). Finally, early TEP components can be used to compare the effects of single and paired-pulse TMS (Ferreri et al., 2011), or to reveal neural changes produced by drugs acting on GABAergic and glutamatergic receptors (Darmani and Ziemann, 2019) (Section 6).

Source modeling and pharmacological studies have shed light on the physiological meaning of late TEPs components especially when M1 is targeted. The N100 component shows a broader scalp distribution compared to early TEPs components, likely reflecting the activation of multiple cortical sources, as well a partial overlap with the topography of those related to peripheral co-stimulation such as auditory and somatosensory evoked potentials (Biabani et al., 2019, Conde et al., 2019). Its amplitude correlates with the cortical silent period—a brief suppression of voluntary muscle activity following suprathreshold TMS over M1 during contraction (Farzan et al., 2013, Nikulin et al., 2003, Rogasch et al., 2013a)—and is sensitive to GABAB receptor activity, as shown by pharmacological studies (Darmani and Ziemann, 2019) (Section 6). Later components such as the P180 and N280 are thought to reflect large-scale network propagation, although their origins remain poorly understood and may include contributions from peripherally-evoked potentials (Biabani et al., 2019, Rocchi et al., 2021) when not effectively controlled for (Conde et al., 2019, Fecchio et al., 2025).

The clinical relevance of TEP components lies in their potential to serve as biomarkers for neurological and psychiatric disorders (cf. 10, 10.1, 10.2, 10.3, 11, 11.1, 11.2, 11.3, 12, 12.1, 12.2, 12.3, 12.4, 13, 13.1, 13.2, 13.3, 14, 14.1, 14.2, 14.3, 15, 15.1, 15.2, 15.3, 16, 16.1, 16.2, 16.3, 17, 17.1, 17.2, 17.3, 18, 18.1, 18.2, 18.3, 18.4, 18.5, 19, 19.1, 19.2, 19.3, 20, 20.1, 20.2, 20.3, 21, 21.1, 21.2, 21.3, 22, 22.1, 22.2, 22.3, 23, 23.1, 23.2, 23.3, 24, 24.1, 24.2, 24.3, 25, 25.1, 25.2, 25.3, 26).

### 5.3 Tms-related EEG oscillations

#### 5.3.1 Definition and measurement

TMS-EEG responses can be analyzed not only in the time domain—leading to the characterization of TEPs and their components—but also in the frequency and time–frequency domain, which enables the detection of oscillatory activity following the TMS pulse (Wang et al., 2024).

In this framework, a distinction is typically made between phase-locked and non-phase-locked activity (Pellicciari et al., 2017). Phase-locked activity—also referred to as evoked—is consistently aligned in phase with the onset of the TMS pulse. Thus, it is detectable in time-domain after averaging (i.e., TEPs), also by filtering the EEG response to TMS in a band of interest, and time–frequency analysis. In contrast, non-phase-locked (or induced) activity is time-locked to the TMS event but varies in phase across trials. Consequently, it does not appear in the time domain but can be observed using time–frequency analysis (Fig. 5.1A).

The most widely used tool for time–frequency decomposition of EEG signals is the event-related spectral perturbation (ERSP). This wavelet-based method averages changes in the frequency power spectrum of EEG data time-locked to TMS pulses, producing a two-dimensional (time-by-frequency) representation of the mean change in spectral power (in dB) relative to prestimulus baseline (Ferrarelli et al., 2012, Grandchamp and Delorme, 2011, Makeig et al., 2004, Rosanova et al., 2009). ERSP can also be performed at higher temporal resolution using the Hilbert–Huang transform instead of wavelets (Pigorini et al., 2011). From the ERSP, one can also derive the natural frequency, calculated as the peak in the time–frequency power spectrum during the first 200–300 ms following the TMS pulse. This natural frequency corresponds to the dominant frequency of the sustained oscillations elicited by TMS of the targeted cortical area (Ferrarelli et al., 2012, Rosanova et al., 2009), and can be defined, as in physics, as the intrinsic frequency at which a system tends to oscillate after a single perturbation.

Notably, when applied to TMS-EEG responses, ERSP reflects total power and therefore represents a mixture of both phase-locked and non-phase-locked TMS-related oscillatory activity. Specific methods are available to separate these two oscillatory components (Fig. 5.1B). For instance, isolating induced oscillations can rely on subtracting the evoked response from each individual trial before applying the time–frequency decomposition (Premoli et al., 2017a). Subsequent studies have introduced data-driven approaches, such as parallel factor analysis (PARAFAC), to address the complexity of multidimensional (≥4 dimensions, including electrode, time, frequency, subject, and other experimental factors) TMS-induced oscillation datasets (Belardinelli et al., 2021, Harquel et al., 2025, Tangwiriyasakul et al., 2019). This method simplifies the data into a concise set of distinct and parsimonious components, or oscillatory modes.

#### 5.3.2 Neural underpinnings

In addition to demonstrating that TMS over the cat’s primary visual cortex can drive cortical neurons to fire action potentials for up to 500  ms after the pulse, above a certain stimulation intensity, peaks of facilitation recur at ∼ 100  ms intervals (corresponding to a frequency to ∼ 10 Hz), resembling oscillations in the alpha band (Moliadze et al., 2003). Similar results were obtained in non-human primates by targeting the parietal cortex. Romero and colleagues observed that, although TMS did not evoke significant spiking responses in neurons located 2  mm or more from the stimulation center, it did induce strong oscillations—primarily in a frequency band below 10 Hz—in their firing rates, which may also contribute to the TMS-EEG response (Romero et al., 2019). Finally, combining intracranial recordings with TMS applied to different cortical sites revealed widespread power increases lasting ∼ 500  ms. These responses reflected a mixture of evoked and induced oscillations and appeared to be region-specific (Solomon et al., 2024).

#### 5.3.3 Physiological and clinical relevance

Alongside studies examining the effects of different drugs on TEPs (Darmani and Ziemann, 2019) (Section 6), the analysis of TMS-related EEG oscillations can help elucidate the physiological mechanisms underlying TMS–cortex interactions and the functional properties of brain circuits. For instance, TEPs recorded in patients with unilateral thalamectomy of the ventrolateral nuclei showed a marked reduction in beta-band (15–30  Hz) oscillations following stimulation of the ipsilesional M1 (Van Der Werf et al., 2006), suggesting that cortico-thalamo-cortical reverberations contribute to TMS-related EEG oscillations. Several studies have demonstrated that different cortical targets exhibit distinct natural frequencies in their TEPs (D'Ambrosio et al., 2022, De Martino et al., 2024, Ferrarelli et al., 2012, Garcia et al., 2011, Rosanova et al., 2009, Vallesi et al., 2021). Anterior cortical regions tend to oscillate at higher frequencies (beta and gamma bands) following TMS, whereas posterior regions preferentially oscillate in the beta and alpha bands. This anterior–posterior frequency gradient was also reported in a study that systematically mapped 18 cortical targets using a robotic arm (Harquel et al., 2016). These oscillatory differences are likely shaped by local cytoarchitectonics and cortico–cortical interactions, as suggested by modeling studies (Cona et al., 2011). Moreover, stimulating nearby targets within the sensorimotor cortex, or across the premotor cortex and M1, has revealed distinct early dynamics between cortical sites on short distances (Lioumis et al., 2025, Passera et al., 2022), highlighting the fine-grained spatial differentiation in the input–output properties of cortical circuits.

The differentiation of TEPs—and their associated natural frequencies—has functional significance in both healthy and pathological conditions. Natural frequencies are reduced in the frontal cortex of individuals with schizophrenia (Donati et al., 2025, Ferrarelli et al., 2012) and delirium (Bai Y et al., 2023a) and are replaced by stereotypical slow waves in perilesional areas after stroke (Bai Z et al., 2023a, Sarasso et al., 2020, Tscherpel et al., 2020). Notably, this regional differentiation is also completely lost during NREM sleep (Massimini et al., 2007) and in the vegetative state (Rosanova et al., 2018).

The distinction between evoked and induced oscillatory responses remains incompletely understood (David et al., 2006). Ongoing brain rhythms likely result from complex interactions between local and remote neural oscillators (Wang et al., 2024); thus, a TMS pulse may elicit a combination of both evoked and induced oscillations (Pellicciari et al., 2017, Solomon et al., 2024). Disentangling these components could provide valuable mechanistic insights. In a landmark study, Premoli and colleagues investigated TMS-induced oscillations over the primary motor cortex (M1) up to 400  ms after stimulation using the subtraction method described above (Premoli et al., 2017a). They observed initial synchronization in the alpha and beta bands (30–200  ms) over the stimulated sensorimotor cortex and adjacent lateral frontal cortex, followed by bilateral desynchronization (200–400  ms) over the sensorimotor cortices in the same frequency bands. Notably, administration of GABAergic drugs significantly modulated these alpha- and beta-band oscillations, suggesting that inhibitory cortical networks strongly contribute to these induced responses.

Analyzing TMS-induced oscillations can also reveal distinct and long-lasting oscillatory modes that would otherwise remain undetected. For instance, applying PARAFAC decomposition to TMS-EEG datasets over an extended post-stimulus window (up to 900  ms) identified three to four physiologically meaningful oscillatory modes induced by M1 stimulation (Harquel et al., 2025, Tangwiriyasakul et al., 2019). These modes were temporally and spectrally distinct: each was dominated by a specific frequency band (low-frequency, alpha, or beta) and associated with a unique spatiotemporal pattern.

The most reproducible pattern consisted of a late (>200  ms) and prolonged (up to 850  ms) alpha mode, maximal over the parieto-occipital regions (Harquel et al., 2025, Tangwiriyasakul et al., 2019) and the stimulated M1 (Belardinelli et al., 2021). A second mode corresponded to sensorimotor beta-band activity, originating in the sensorimotor cortex, characterized by an early burst (<50–100  ms) followed by a later rebound (>400  ms). Importantly, pharmaco-TMS-EEG studies showed that these induced alpha and beta patterns are directly modulated by glutamatergic and GABAergic inhibitory systems (Belardinelli et al., 2021, Premoli et al., 2017a).

Overall, these findings highlight the value of studying TMS-induced oscillations to understand the excitation / inhibition (E/I) balance in cortical circuits, particularly in neurological and psychiatric disorders (Ghatak et al., 2021).

### 5.4 Local and global measures of brain response to TMS and state-dependency

In wakefulness, a single TMS pulse initiates a cascade of neural events, starting with volleys of action potentials in the targeted cortical area. These signals propagate through interconnected axonal pathways to engage distant cortical and subcortical structures, which in turn send feedback activity back to the cortex initially stimulated by TMS. This recurrent activation is most likely reflected in the sequential components of TEPs and in TMS-related oscillatory activity as demonstrated by modelling studies (Momi et al., 2023). These large-scale events depend on the ability of the TMS pulse to elicit changes not only in the cortical region underneath the coil but also in distant areas through effective connectivity—defined as the capacity of one cortical area to causally influence the activity of another connected region (Friston, 2002). Massimini and colleagues showed that the fast recurrent waves and long-range activations typical of TEPs recorded in wakefulness disappear at sleep onset (Massimini et al., 2005). During light sleep (N1/N2), the early TEP component (<50  ms) becomes more prominent while later waves are markedly reduced. In deep sleep (N3), the initial positive deflection nearly doubles in amplitude, is delayed, and is followed by a large negative slow wave peaking at ∼ 150  ms. Depending on the stimulation site, this positive–negative sequence remains localized near the stimulation site (Massimini et al., 2007, Massimini et al., 2005) or spreads over the large portions of the cortex (Bergmann et al., 2012, Massimini et al., 2007) and illustrates how complex network interactions sustained by effective connectivity, hence causal interactions, can collapse within minutes despite an intact structural connectome. Since then, several indices have been developed to quantify local and global causal interactions underlying TEP dynamics at both sensors and sources level (Casali et al., 2010, De Martino et al., 2025) (Fig. 5.1B).

The enhanced early positive TEP component observed during sleep (Sachdev et al., 2004, Wörgötter et al., 1998)is followed by a prominent negative wave and a marked suppression of high-frequency (>20 Hz) activity, consistent with sustained neuronal hyperpolarization and silence (Rosanova et al., 2018). These dynamics resemble cortical OFF-periods—phases of neuronal silence underlying sleep delta waves —possibly driven by intrinsic hyperpolarizing mechanisms (Massimini et al., 2024). Importantly, OFF-periods disrupt also local causality and effective connectivity(Rosanova et al., 2018). This collapse of local and global interactions impairs the brain’s capacity for integrated processing and is captured by a drop in PCI (Fig. 5.1B), a global marker of TEP complexity (Casali et al., 2013). A similar cascade of events has been documented under general anesthesia (Sarasso et al., 2015) (Section 6), in patients with disorders of consciousness following severe brain injury (Casarotto et al., 2016, Massimini et al., 2024) (Section 14), and locally in perilesional areas after stroke (Sarasso et al., 2020, Tscherpel et al., 2020) (Section 20). Importantly, these alterations can be reversible: the resurgence of complex, causal dynamics has been shown to accompany the recovery of consciousness (Rosanova et al., 2012) and functional improvement post-stroke (Sarasso et al., 2025). Understanding how TEPs change across these states may improve their diagnostic and prognostic relevance.

Building on the above evidence linking cortical reactivity in TMS-EEG responses to prestimulus brain states, recent work emphasizes the role of E/I balance in shaping cortical responses. Since E/I imbalance is a hallmark of many neurological and psychiatric disorders (Ghatak et al., 2021), capturing it non-invasively is crucial (Ferrarelli and Phillips, 2021).

The aperiodic component of the EEG power spectrum, particularly the 1/f slope, has emerged as a promising marker of E/I balance (Donoghue et al., 2020; Gao et al., 2017). This slope reflects how EEG power declines with frequency, with steeper slopes indicating increased inhibition, and flatter ones suggesting greater excitation.

Recent TMS-EEG studies have leveraged this metric: Thong and colleagues found that during motor learning, reduced inhibition (lower N45) was accompanied by a steeper 1/f slope, correlating with learning success (Thong et al., 2025). Pellegrino and colleagues reported similar slope changes after intermittent theta-burst stimulation (iTBS) (Pellegrino et al., 2024), highlighting its sensitivity to excitability shifts (Salvatore et al., 2024).

Incorporating 1/f slope estimates—either at rest or trial-wise—alongside TEPs (e.g., N45, N100), oscillations, and clinical outcomes may enrich our understanding of ongoing brain state and improve the sensitivity of TMS-EEG to both trait- and state-related alterations. This is especially relevant for disorders with altered E/I dynamics (Gao and Penzes, 2015, Hu et al., 2023).

## 6 Pharmacology of TMS-EEG

### 6.1 Pharmacology of TMS-EEG with stimulation of motor cortex (M1)

All reported drug effects were obtained with a single oral dose in healthy subjects, in a double-blind randomized placebo-controlled crossover design. Only TMS-EEG studies are reported here that have used TMS intensities of 90–100 % of resting motor threshold (RMT) to avoid significant contamination of TMS-EEG responses by re-entry somatosensory input caused by MEPs (Fecchio et al., 2017), a focal figure-of-eight coil, and an orientation of the induced electrical field from lateral-posterior to medial-anterior and a monophasic current waveform, to prevent variation of TMS-EEG responses with changes in electrical field orientation and current waveform (Bonato et al., 2006, Casula et al., 2018b, Tervo et al., 2022).

#### 6.1.1 Effects of drugs on TEPs

The early TEPs with peak latencies < 70 ms are responses that reflect specifically direct brain responses to TMS. In contrast to later TEPs they are not contaminated by non-specific responses generated by auditory and somatosensory input associated with the TMS pulse (Belardinelli et al., 2019, Conde et al., 2019, Gordon et al., 2023a, Hernandez-Pavon et al., 2023). The early TEPs to motor cortex stimulation can be reliably divided into the following components, according to their polarity and peak latency (in milliseconds): N15 (or N18), P25 (or P30), N45, and P60 (or P55, or P70) (Beck et al., 2024b, Bonato et al., 2006, Ilmoniemi and Kicic, 2010, Rogasch et al., 2014).

Table 6.1 summarizes the drug effects from the currently available studies on these early TEP components. Only drugs with specific singular modes of action were considered. Drugs with several modes of action, such as levetiracetam that is a ligand at the presynaptic vesicle protein SV2A but has also shows direct inhibitory action on glutamatergic neurotransmission through AMPA and NMDA receptors, were disregarded.

**Table 6.1:** Effects of drugs on amplitude of TEP components.

**Drug**	**N**	**Dose**	**N15**	**P25**	**N45**	**P60**	**Reference**
*VGSC blocker*							
Carbamazepine*	15	600 mg	n.t.	↓	●	●	(Darmani et al., 2019)
Lamotrigine*	15	300 mg	n.t.	●	●	●	(Premoli et al., 2017b)
*PAM of KCNQ2/3 (potassium channel opener)*							
XEN1101^	20	20 mg	↓		↓	n.t.	(Premoli et al., 2019)
*PAMs of the GABAARs*							
Diazepam	19	20 mg	n.t.	●	↑	●	(Premoli et al., 2014a)
Diazepam^#^	20	20 mg	n.t.	●	↑	↓	(Gordon et al., 2023b)
Alprazolam	22	1 mg	n.t.	●	↑	●	(Premoli et al., 2014a)
Zolpidem	22	10 mg	n.t.	●	↑	●	(Premoli et al., 2014a)
*GABA re-uptake inhibitors*							
Tiagabine	15	15 mg	n.t.	●	●	●	(Darmani et al., 2019)
*α5-GABAAR antagonist*							
S44819	18	100 mg	n.t.	●	↓	●	(Darmani et al., 2016)
*GABABR agonists*							
Baclofen	19	50 mg	n.t.	●	●	●	(Premoli et al., 2014a)
*Specific ligands to the presynaptic vesicle protein SV2A*							
Brivaracetam	15	100 mg	n.t.	●	●	●	(Darmani et al., 2019)
*AMPAR antagonists*							
Perampanel	18	12/6mg	n.t.	●	●	↓	(Belardinelli et al., 2021)
*NMDAR antagonists*							
Dextromethorphan	18	120 mg	n.t.	●	↑	●	(Belardinelli et al., 2021)
*L-type voltage-gated calcium channel blocker*							
Nimodipine	18	30 mg	n.t.	●	●	●	(Belardinelli et al., 2021)

*Effects were demonstrated with and without TMS intensity correction to adapt for drug-induced RMT increase;^Effects were not controlled by TMS intensity correction of drug-induced RMT increase; ^#^ Effects were controlled by a realistic TMS sham condition. Abbreviations: AMPAR, α-amino-3-hydroxy-5-methyl-4-isoxazolepropionic acid receptor; GABA, gamma-aminobutyric acid; GABAAR, GABA-A receptor; GABABR, GABA-B receptor; NMDAR, N-methyl-D-aspartate receptor; PAM, positive allosteric modulator; VGSC, voltage-gated sodium channel; n.t., not tested; ●, no effect; ↑, increase of TEP amplitude; ↓, decrease of TEP amplitude.

This survey indicates that the pharmacological characterization of TEPs is still preliminary, as compared to pharmaco-TMS-EMG studies (Ziemann et al., 2015), with only few studies that have been conducted in accord with the above outlined standards. It appears that the N15-P25 complex is regulated by drugs acting on ion channel conductivity, while the N45 is modulated by drugs operating on GABAARs and NMDARs, with shifting the excitation/inhibition (E/I) balance towards more inhibition resulting in an increase of N45 amplitude. Finally, the P60 appears to be depressed by AMPAR inhibition. Clearly, more work is needed to replicate and extend these findings. Only one study so far has applied a realistic sham control condition (Gordon et al., 2023b). It largely replicated the effects of diazepam on the N45-P60 complex from earlier studies. More importantly, it demonstrated that drug effects described in earlier non-sham-controlled studies on late TEPs (>70 ms) were largely caused by drug effects on PEPs rather than TEPs, justifying the limitation of the current survey on the pharmacological characterization of TMS-EEG responses to early TEPs.

In addition to single-pulse pharmaco-TMS-EEG studies in M1, one paired-pulse TMS-EEG study tested long-interval intracortical inhibition (LICI), using an interstimulus interval of 100 ms, and demonstrated that the amplitudes of the P25 and N45 responses were reduced and the amplitude of the P60 was increased by the conditioning pulse (Premoli et al., 2014b). As expected, baclofen resulted in a trend to increase LICI of the N45 response, while diazepam had no effect on LICI of the P30 and N45 responses (Premoli et al., 2014b).

#### 6.1.2 Effects of drugs on TMS-related spectral perturbations (TRSPs)

Drug effects on TRSPs have been studied even less. The time window of interest was typically set to 30–200 ms after the TMS pulse, showing synchronization in the alpha- and beta-bands over the stimulated sensorimotor cortex and adjacent frontal cortex. The VGSC blocker lamotrigine decreased delta power around the site of stimulation of the left M1 in one study (Biondi et al., 2022) but suppressed oscillations in the alpha range in the occipital region when applying a specific tensor decomposition approach for data analysis (Tangwiriyasakul et al., 2019). The potassium channel opener XEN1101 decreased delta TRSP-power in a broad area including the site of stimulation of the left M1 (Biondi et al., 2022). The PAMs of GABAARs, diazepam and zolpidem enhanced, and alprazolam tended to enhance TRSP-power in the alpha band around the site of stimulation of the left M1 (Premoli et al., 2017a). In addition, alprazolam and the GABABR agonist baclofen decreased power in the beta band (Premoli et al., 2017a). These effects were not trivially explained by drug-induced changes on MEP amplitude, or resting-state EEG power. In another study, diazepam resulted in a decrease in TRSP-power in the low-beta frequency band around the site of stimulation of the left M1 (Gordon et al., 2023b). Perampanel increased power in the beta-frequency band in the stimulated M1, and in the alpha-frequency band in midline parietal channels, while dextromethorphan and nimodipine had no effect on TRSPs (Belardinelli et al., 2021).

A later time window of interest 200–400 ms is associated with a desynchronization in the alpha and beta frequency bands. In this time window, lamotrigine resulted in a decrease of theta TRSP-power (Biondi et al., 2022), and XEN1101 decreased theta, alpha and beta TRSP-power (i.e., an enhancement of alpha and beta desynchronization) in a broad centro-parietal area (Biondi et al., 2022). Alprazolam and diazepam also resulted in an enhancement of beta desynchronization (Premoli et al., 2017a), and baclofen enhanced alpha and beta desynchronization (Premoli et al., 2017a).

These data are too preliminary to draw any strong conclusions on a systematic pattern of pharmacological effects on TRSPs. However, the data support the view that the alpha and beta desynchronization in the time window 200–400 ms after the TMS pulse reflect E/I balance, exhibiting an increase of desynchronization with shifts of E/I balance towards less excitation (potentiation the open state of potassium channels) or more inhibition (PAMs of GABAARs, agonist at GABABR).

Most of the tested drugs also significantly affect power of different frequency bands in the resting-state EEG. Application of a realistic sham control condition enables disentangling these effects from the drug effects on TRSPs (Gordon et al., 2023b).

#### 6.1.3 Effects of drugs on TMS-EEG response complexity

**Fig. 6.1:**
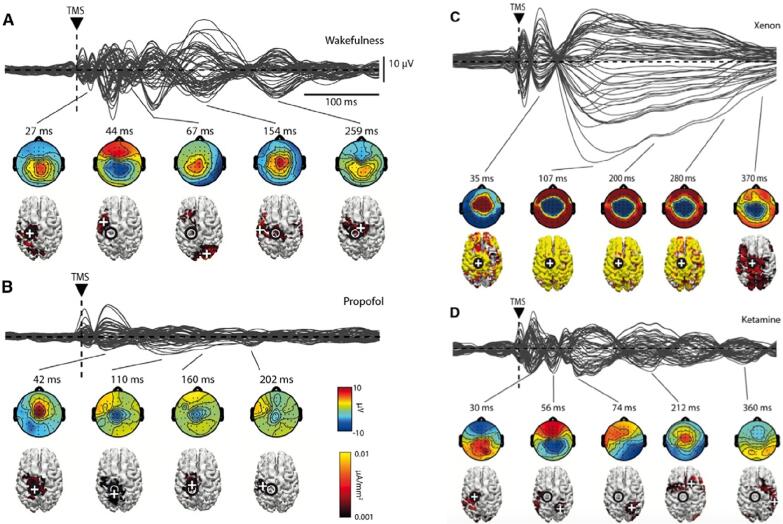
Different spatiotemporal dynamics induced by propofol, xenon, and ketamine. (A–D) Representative averaged TMS-evoked potentials at all electrodes, superimposed in butterfly plots together with voltage topographies and absolute cortical current density reconstructions estimated with L2 norm in periods of significant TMS-evoked activation during wakefulness (A), propofol (B), xenon (C), and ketamine (D). Black circle superimposed to the cortical surface represents TMS target; the current density distribution is thresholded to highlight the location of maximum current sources (white cross) (modified from (Sarasso et al., 2015), with permission).

TMS-EEG response integration and differentiation can be indexed by PCI (Casali et al., 2013, Comolatti et al., 2019). PCI values and drug effects on PCI are largely independent of the site of stimulation (Casali et al., 2013). Therefore, this survey includes also studies that have applied TMS outside of M1. Ferrarelli and colleagues demonstrated that loss of consciousness (LOC) induced by midazolam, a PAM of GABAARs, resulted in a short-lived TMS-EEG response that did not propagate into cortical areas away from the site of stimulation over premotor cortex (Ferrarelli et al., 2010). In addition, an index of significant current scattering (SCS), reflecting effective connectivity, was used to distinguish between consciousness and LOC, with SCS being significantly lower during LOC compared to wakefulness (Ferrarelli et al., 2010). Collectively, this was interpreted as a breakdown of effective cortico-cortical connectivity, similar to the TMS-EEG responses observed during non-REM slow-wave sleep (Massimini et al., 2005). The range of PCI values during wakefulness was between 0.44 and 0.67, with a mean ± SD of 0.55 ± 0.05 (Casali et al., 2013). During midazolam-induced LOC, PCI decreased to 0.28 ± 0.03 (range, 0.23 to 0.31) (Casali et al., 2013). Under general anesthesia with propofol, a strong positive modulator of GABAARs, PCI decreased to 0.23 ± 0.04 (range, 0.13 to 0.30), and under general anesthesia with xenon, a strong potentiator of conductivity through two-pore domain potassium (2PK + ) channels, PCI decreased to 0.23 ± 0.06 (range, 0.12–0.31) (Casali et al., 2013). These observations were confirmed and further refined in a subsequent investigation (Sarasso et al., 2015) (Fig. 6.1). General anesthesia and LOC induced by propofol resulted in a drop of PCI (mean: 0.24, range: 0.20 to 0.30), and the TMS-EEG responses consisted of a low-amplitude, local positive–negative wave that rapidly faded without propagating from the site of stimulation (Fig. 6.1B). Similarly, under general anesthesia and LOC induced by xenon, PCI dropped (mean: 0.17, range: 0.08 to 0.24), but the TMS-EEG response consisted of an initial high-amplitude positive component followed by a high-amplitude, stereotypical negative wave that spread gradually to the rest of the cortex, a pattern closely resembling the TMS-EEG responses during non-REM sleep (Fig. 6.1C) (Massimini et al., 2007, Massimini et al., 2005). In contrast, during LOC induced by ketamine, typically associated with vivid dreaming activity, PCI values (mean: 0.44, range: 0.35 to 0.55) remained comparable to those during wakefulness, and the TMS-EEG response was complex, characterized by long-range spatiotemporal dynamics closely resembling that evoked during wakefulness (Sarasso et al., 2015) (Fig. 6.1D). Ketamine is a NMDAR antagonist, but unlike propofol does not potentiate GABAAR activity, and unlike xenon does not potentiate potassium currents, which may explain why it is not as effective in disrupting the complexity of cortico-cortical interactions. Similarly, exposure to psilocybin, a serotonergic psychedelic drug that produces increased sensory-emotional awareness and arousal states, did not alter PCI (Ort et al., 2023). In summary, drug-induced LOC is directly linked to low PCI values (typically ≤ 0.31), i.e., a significant degradation of the TMS-EEG response integration and differentiation. Reductions of PCI values intermediate between wakefulness (PCI ≥ 0.44) and unconsciousness (PCI ≤ 0.31) were demonstrated during low-dose propofol anesthesia (Casali et al., 2013), and by increasing the availability of GABA in the synaptic cleft by the GABA re-uptake inhibitor tiagabine, in this case even without overt signs of altered consciousness (Darmani et al., 2021). In contrast, other excitability-decreasing drugs, but without enhancing action on GABAAR activity (carbamazepine, brivaracetam) did not alter PCI (Darmani et al., 2021). While the pharmacological characterization of TMS-EEG response complexity also requires further systematic investigation, the available data suggest that PCI is modifiable by GABAAR activity and conductivity through potassium channels, and can be used as an objective measure of consciousness.

### 6.2 Pharmacology of TMS-EEG with stimulation of non-M1 areas

Two studies (Ferrarelli et al., 2010, Sarasso et al., 2015) have been surveyed in the previous section. One other study assessed the effects of dextromethorphan (an NMDA-receptor antagonist) on TEPs following TMS of the prefrontal and premotor cortex (Rogasch et al., 2020). In contrast to findings from M1, prefrontal and parietal TEPs were unchanged following dextromethorphan administration, suggesting different mechanisms may underlie TEPs following stimulation of different sites. Another study investigated the effects of scopolamine (a non-selective muscarinic acetylcholine receptor antagonist) and biperiden (a non-selective and selective muscarinic acetylcholine receptor antagonist, respectively) on TEPs following TMS of the medial prefrontal cortex (mPFC), supplementary motor area (SMA), and angular gyrus (AG) (Song et al., 2025). The N45 was increased following scopolamine and to a lesser extent biperiden for SMA TEPs, but not mPFC or AG, suggesting SMA TEPs are sensitive to cholinergic inhibition. The other two studies evaluated the roles of cholinergic, dopaminergic, GABAergic, and glutamatergic neurotransmission on LICI (Salavati et al., 2018b) and plasticity induction by paired associative stimulation (PAS) (Salavati et al., 2018a) with TMS of the dorsolateral prefrontal cortex (DLPFC), assessing the effects of baclofen, rivastigmine, dextromethorphan, and L-DOPA administration in placebo-controlled randomized crossover study designs. Therefore, the number of pharmaco-TMS-EEG studies in non-M1 areas is still very limited compared to those in M1, and further research and validation are needed. The last two studies will be reviewed in more detail in the next paragraphs.

#### 6.2.1 Effects of drugs on GABABR-mediated inhibition as indexed by LICI of the DLPFC

LICI was assessed according to an established protocol comparing TMS-EMG responses (cortical evoked activity) to paired-pulse TMS (interstimulus interval, 100 ms) with those to single-pulse TMS (Daskalakis et al., 2008), and the effects of each drug were compared to the placebo condition. As expected, the selective GABABR agonist baclofen resulted in a significant increase in LICI. In addition, the acetylcholine-esterase inhibitor rivastigmine led in a significant decrease in LICI, while dextromethorphan and L-DOPA did not produce significant changes in LICI compared to placebo (Salavati et al., 2018b). The baclofen results replicated and extended the results of the TMS-EEG study that tested the effects of baclofen on LICI in M1 (Premoli et al., 2014b). The study confirmed that LICI from the DLPFC is primarily mediated by inhibitory neurotransmission via GABAB receptors and suggested that enhancement of cholinergic activity reduces LICI in the DLPFC.

#### 6.2.2 Effects of drugs on the neuroplasticity as indexed by PAS from the DLPFC

Salavati et al. also evaluated the effects of baclofen, rivastigmine, dextromethorphan, and L-DOPA on PAS-induced plasticity in the DLPFC (Salavati et al., 2018a), following a previously established PAS protocol using a TMS-EEG readout (Rajji et al., 2013). The results showed in accord with their hypotheses that L-DOPA and rivastigmine enhanced PAS-induced long-term potentiation (LTP)-like plasticity in the DLPFC, while dextromethorphan suppressed it (Salavati et al., 2018a). The findings on L-DOPA in the DLPFC replicated and expanded previous findings that evaluated dopaminergic modulation of PAS-induced LTP-like plasticity in M1 through MEPs (Nitsche et al., 2009). In contrast, baclofen did not significantly suppress the PAS-induced neuroplasticity compared to placebo. This is the first and up to now only TMS-EEG study that has tested pharmacological modulation of neural plasticity induced by non-invasive brain stimulation.

### 6.3 Summary and prospectives

Altogether, the pharmacology of TMS-EEG responses is still limited. Testing the neurophysiological mechanisms underlying readouts of TMS-EEG measurements by CNS-acting drugs with a specific mode of action is a very elegant, if not the best way of mechanism-anchored TMS-EEG biomarker development. This strategy was already very successful in furthering our mechanistic understanding of TMS-EMG readouts (Ziemann et al., 2015), to the extent that TMS-EMG readouts are now broadly used in evidence-based clinical applications as biomarkers of altered E/I balance in a large variety of pathologies (Vucic et al., 2023). The same development is possible for the development of TMS-EEG biomarkers, but this will require more extensive and systematic investigation of drug effects on TMS-EEG measures.

## 7 Roadmap to clinical translation

The field of TMS-EEG research is rapidly evolving. For use in clinical applications, the outcomes of TMS-EEG need to be both reliable (i.e., reproducible within individuals and across research groups) and valid (i.e., represent a true measure of the underlying neural signal or pathophysiology of interest) (Farzan, 2024, Kallioniemi and Daskalakis, 2022, Parmigiani et al., 2023). In the previous review on the clinical utility of TMS-EEG (Tremblay et al., 2019), we identified several key challenges, which required improvement to allow translation of TMS-EEG as a clinical technique. In this section, we review progress in these areas over the last 6 years and highlight areas requiring further improvement. We finish by proposing a checklist to assess the progress of a given TMS-EEG biomarker on the road to clinical translation.

### 7.1 Standardized methods for data collection, analysis, and quantification

A major finding of the last several years is that TEPs are highly sensitive to the way in which TMS-EEG data are collected and analyzed. The signal-to-noise ratio of TEPs (i.e., the size of the neural response triggered by TMS of the cortex versus the size of artifacts, see also Section 2) is crucially dependent on a range of factors during data collection, including coil position and angle, the use of neuronavigation, stimulation intensity, pulse shape, interpulse interval, amplifier settings, and the methods used to minimize sensory input (Hernandez-Pavon et al., 2023). Furthermore, using different cleaning pipelines (i.e., the processing steps used to minimize artifacts in the signal after data collection) can also alter TEP properties (Bertazzoli et al., 2021, Rogasch et al., 2022) (see also Section 3). While TEPs tend to be highly reliable within a research group using the same methods (e.g., (Casarotto et al., 2010)), different methodological practices across the field limit the external comparability of findings between research groups (Beck et al., 2024b, Belardinelli et al., 2019, Siebner et al., 2019). Methodological variability is therefore a major challenge in clinical TMS-EEG studies seeking to uncover reliable and valid biomarkers of pathophysiology in brain disorders.

Given the sensitivity of TEPs to methodological decisions, clinical translation of TMS-EEG research protocols requires careful standardization of methods for data collection and analysis, as well as proper training of technicians and clinicians (Fried et al., 2021). As an additional issue, the several components of navigated TMS-EEG are often provided by different manufacturers, each of which is specialized in the development and production of either the neuronavigation software or the TMS unit or the EEG amplifier (see also Section 1). However, combining navigated TMS and EEG entails specific requirements as compared to simply applying simultaneously the two techniques. For a proper translation of TMS-EEG potentiality in the clinics, it would be advisable to promote the development and commercialization of TMS-EEG devices by the same manufacturer, who should be able to specifically test the entire equipment for compatibility, handiness and reliability. Experienced TMS-EEG researchers should represent the primary source of information for companies interested in pursuing this business.

The effects of TMS on cortical neurons cannot be precisely controlled a priori, because they crucially depend on anatomical and functional parameters that are difficult to measure (e.g., axon orientation, fine cortical geometry and neuronal excitability). Thus, recording TMS-evoked brain responses unconfounded by major artifacts and with a high signal-to-noise ratio implies the necessity to carefully control for EEG signal quality already during data collection. This approach, together with a freely available software tool, has been recently described by Casarotto and colleagues (Casarotto et al., 2022). In addition, software for developing more effective masking noise for suppressing perception of the TMS click sound was also recently released (Russo et al., 2022b). The availability of open-source methods to improve signal quality during TMS-EEG data collection represents a major step forward in the development of standardized clinical TMS-EEG protocols.

Avoiding artifacts during data collection remains the gold standard in collecting high quality TEPs, but this approach is challenging in lateral brain regions. For example, many clinical TMS-EEG studies target the DLPFC due to its involvement in the pathophysiology of various brain disorders. However, stimulation of the DLPFC results in large scalp muscle artifacts (Hernandez-Pavon et al., 2022, Korhonen et al., 2011, Mutanen et al., 2013a, Rogasch et al., 2013b) and larger somatosensory-evoked potentials than stimulation of other regions, which are hard to avoid (Biabani et al., 2024). As a result, offline cleaning protocols have been developed to try and uncover the underlying TEPs from the noise (Rogasch et al., 2014). Several open-source toolboxes dedicated to TMS-EEG analysis have been released (Atluri et al., 2016, Cline et al., 2021, Rogasch et al., 2017, Wu et al., 2018) and subsequently updated with state-of-the-art cleaning protocols as they become available (Mutanen et al., 2020). ICA-based methods have proved particularly popular for attempting to suppress artifacts in TMS-EEG recordings. However, recent theoretical (Atti et al., 2024, Mutanen et al., 2024) and experimental (Biabani et al., 2024) findings have challenged the effectiveness of ICA for cleaning artifacts such as TMS-evoked muscle activity from TMS-EEG data due to violation of several key assumptions, calling into question the validity of these cleaning protocols. The implication is that outcomes from TMS-EEG data with more severe artifact profiles may not be as reliable as data where artifacts are avoided during data collection. Approaches are currently being developed for assessing the effectiveness of TMS-EEG cleaning pipelines by comparing their ability to uncover ‘ground-truth’ TEPs (i.e., TEPs of known shape and amplitude that are subsequently hidden under noise introduced in experimental or simulated data) (Brancaccio et al., 2024, Metsomaa et al., 2024, Mutanen et al., 2024). Furthermore, standardized ways of sharing existing brain stimulation data are currently under development (e.g., an extension of the brain imaging data structure for non-invasive brain stimulation data, NIBS-BIDS, https://bids.neuroimaging.io/extensions/beps/bep_037.html). The availability of data from different research groups is essential for thoroughly testing and validating TMS-EEG cleaning pipelines, thereby ensuring generalizability of existing and newly developed methods. The continued development of cleaning pipelines of TMS-EEG data is of paramount importance for informing and validating clinical TMS-EEG protocols targeting lateral brain regions.

TEPs are highly sensitive to the intensity of stimulation. When stimulating M1, stimulation intensity is often titrated to RMT, i.e., the minimum intensity required to evoke a measurable MEP in ∼ 50 % of trials. However, another challenge is defining how to set equivalent stimulation intensities in regions outside of M1 that do not have easily measurable outcomes like an MEP. Such targets are common in clinical TMS-EEG studies. Currently, a range of approaches are used to set stimulation intensity for non-motor sites including using the RMT, adjusting the RMT based on scalp/coil-to-cortex distance, using the estimated E-field strength, or using a ratio of the E-field strength relative to E-field achieved at RMT (Numssen et al., 2024). Each of these approaches results in a different absolute stimulation intensity, which has potential to bias TEPs between groups (e.g., if there is a systematic difference in coil-to-cortex distance or gyrification between groups). There is currently no consensus on how best to set stimulation intensity in TMS-EEG studies, an issue which needs to be addressed for clinical applications.

TEPs are also sensitive to brain-state. The N100 and P200 components are the most consistently reproducible (Kerwin et al., 2018, Moffa et al., 2022) and remain stable across different analytical approaches (Bertazzoli et al., 2021, Farzan and Bortoletto, 2022). However, caution should be exercised interpreting this high reliability due to contamination of these later components with somatosensory and auditory evoked potentials (Belardinelli et al., 2019, Conde et al., 2019). In contrast, earlier TEP components (approximately < 50 ms) tend to exhibit lower reliability, which could reflect genuine fluctuations in cortical excitability. These variations might be mitigated by accounting for the brain’s current state—for instance, using EEG-guided TMS targeting. Supporting this idea, recent work (Kabir et al., 2024) has highlighted how microstate dynamics immediately preceding TMS can influence the amplitude of early TEPs (<80 ms).

### 7.2 Adequate sensory masking and control conditions

Another major finding of the last several years is that commonly used methods for suppressing sensory-evoked potentials in TMS-EEG are not always as effective as expected. Despite the use of noise masking and foam padding, several studies have reported sensory-evoked potentials under certain experimental conditions (Biabani et al., 2019, Conde et al., 2019), which are larger following stimulation of regions, such as the prefrontal cortex for somatosensory input (Biabani et al., 2024). This finding has important implications for interpreting clinical TMS-EEG studies, as between-group differences in certain TEPs, particularly frontocentral N100/P200 peaks, could reflect differences in sensory-evoked potentials as opposed to activity from the targeted cortical site or network. From an experimental design perspective, these findings suggest that the success of sensory masking needs to be confirmed on an individual participant level, with experimental parameters such as the position of the coil and the volume and frequency content of the masking noise optimized to prevent sensory experiences such as scalp muscle activity and perception of the click noise (Casarotto et al., 2022, Russo et al., 2022b). Furthermore, making the timing of the TMS pulses more predictable might also help reduce sensory-evoked potentials, presumably through an active gating mechanism (Ross et al., 2022). Of note, effective sensory masking has been demonstrated for some TMS-EEG protocols and stimulation sites (Massimini et al., 2005, Rocchi et al., 2021), but may not be achievable for all stimulation sites and conditions. Control conditions, which mimic the sensory experience of TMS without providing transcranial stimulation of the cortex are important to: 1) confirm the success of a sensory masking method; and 2) assess whether a group difference in a TEP feature can be explained by differences in sensory processing. Currently, control conditions include stimulating away from the scalp (Poorganji et al., 2021), stimulating a non-cephalic site like the shoulder (Biabani et al., 2019, Herring et al., 2015), or using electrical stimulation of the scalp with the coil held off the scalp to mimic the somatosensory and auditory experience of TMS (Conde et al., 2019, Gordon et al., 2021, Raffin et al., 2020). However, none of these methods perfectly capture the sensory experiences of TMS and their systematic application may considerably prolong the duration of the experiments. Nonetheless, adequate control conditions are essential for confirming the mechanistic specificity of TEP biomarkers in clinical studies. Many clinical TMS-EEG findings, particularly those including N100/P200 peaks (Tremblay et al., 2019), may need to be re-evaluated in light of potential sensory involvement.

### 7.3 Influence of medications

Individuals with brain disorders are often receiving a range of medications to treat their medical conditions, many of which are neuroactive and have the potential to confound measures of brain function (see also Section 6). For example, in case/control studies a between-group difference in a given TEP feature could reflect a pathophysiological mechanism, or a difference caused by medication. In contrast, the lack of a between-group difference in a TEP feature could reflect intact neural function, or the correction of a pathophysiological mechanism by medication. Perhaps the best method for disentangling the possible effects of medication on TEPs in clinical studies is to test participants on and off medication (where this is ethically possible). For example, Casarotto and colleagues applied TMS-EEG to the left and right supplementary motor area of individuals with Parkinson’s disease both on and off levodopa (a precursor of dopamine). Off drug, TEPs were reduced in amplitude following TMS of the hemisphere with greater subcortical dopamine loss, a result which was reversed following administration of levodopa (Casarotto et al., 2019). This finding highlights how medication commonly used in people with Parkinson’s disease reversed a pathophysiological biomarker, which would not have been evident if participants were all tested on medication. If medication cannot be withheld, an alternative approach is to correlate overall medication dose (e.g., using a measure of dose equivalence between different medications) with the TEP feature of interest (e.g., (Farzan et al., 2010, Ferrarelli et al., 2012)). While this approach is useful to rule-in a possible medication-TEP relationship if a positive result is found, a null finding is more difficult to interpret as typical frequentist statistical approaches do not test evidence for the null hypothesis. Bayesian statistics, which test evidence for and against the null hypothesis may provide a more appropriate method for ruling out a relationship between medications and TEPs. Furthermore, these statistical approaches generally assume a linear relationship between drug dose and the TEP feature of interest, which may not always be the case. Determining whether a between-group difference/similarity in a TEP feature reflects the underlying pathophysiology of the brain disorder or is a byproduct of medication is a key step in establishing the validity of that measure as a biomarker and requires careful consideration.

Another important recent finding is that TEPs from different cortical regions may be sensitive to different underlying neural mechanisms. A key experimental approach to investigate the neural mechanisms underlying TEPs is to give healthy participants a drug of known molecular action and then assess how the TEP is altered (Darmani and Ziemann, 2019). Pharmaco-TMS-EEG studies have been invaluable in interpreting clinical TMS-EEG outcomes in terms of potential pathophysiological mechanisms (cf. Section 6). To date, pharmacological studies have largely focused on TEPs following stimulation of M1 in healthy individuals. The N45 following prefrontal or parietal cortex stimulation is unaffected by dextromethorphan (Rogasch et al., 2020) while the N45 following M1 stimulation is enhanced (Belardinelli et al., 2021). Furthermore, scopolamine, a cholinergic antagonist, increased N45 amplitude following stimulation of the supplementary motor area, but not following stimulation of prefrontal or parietal cortex (Song et al., 2025). Another study found that baclofen, a GABABR agonist, reduced a purported measure of interhemispheric signal propagation following stimulation of M1, but not prefrontal cortex (Hui et al., 2020). These findings have important implications for interpreting clinical TMS-EEG outcomes and call into question whether TEP components like the N45 are universally sensitive to the E/I balance or may reflect different neural mechanisms following stimulation of different sites. As such, care is required when using pharmaco-TMS-EEG studies from M1 to interpret findings from other cortical sites in clinical TMS-EEG studies. More comparative studies assessing how drugs impact TEPs following stimulation of regions outside of M1 are urgently required.

### 7.4 Roadmap to clinical translation

Given the sensitivity of TEPs to methodological choices, clinical translation of TMS-EEG is going to require careful validation of end-to-end protocols describing the equipment, data collection methods, data analysis pipelines, and quantification metrics for a given TEP biomarker. Furthermore, interpretation of the neurophysiological mechanisms underlying TEP biomarkers require validation for specific stimulation sites and in relation to potential sensory and medication confounds. Although consensus on the optimal experimental protocol for obtaining TMS-EEG biomarkers in a clinical setting is still debated and may require specific equipment/protocols for certain biomarkers, some general principles have emerged:

• Use DC-coupled amplifiers with a sampling rate > 5 kHz (low-pass filter > 1 kHz) to minimize duration of pulse and other artifacts, and high-density, 64-channel EEG recordings.
• Ensure a high TEP signal-to-noise ratio (SNR) by:
  o 1) using online feedback to minimize artifacts/optimize TEP amplitude by adjusting coil position and stimulation intensity;
  o 2) using adequate sensory masking such as noise masking and foam padding to minimize TMS-related sensory potentials (and check success against a sensory control condition);
  o 3) collecting a sufficient number of trials (at least 100 clean trials is a heuristic rule); and
  o 4) monitoring EEG quality throughout recording and adjusting the experimental arrangement as required.
• Where possible, use neuronavigation to ensure accurate and consistent coil positioning.
• Share code used for data analysis and biomarker quantification to ensure reproducibility by other groups.

An important consideration for biomarker development is the practicality of applying the protocol in a real-world clinical setting. Protocols need to have sufficient sensitivity and specificity but also need to be completed within a reasonable time frame and without unnecessary burden on the patient. Assessment of the minimum number of channels, trials, interstimulus intervals, stimulation sites etc. is an important consideration in TMS-EEG biomarker development. While most TMS-EEG research has used high-density EEG and a large number of trials, more recent research has begun to explore whether these values can be lowered, which may confer considerable practical benefits in clinical application.

**Fig. 7.1:**
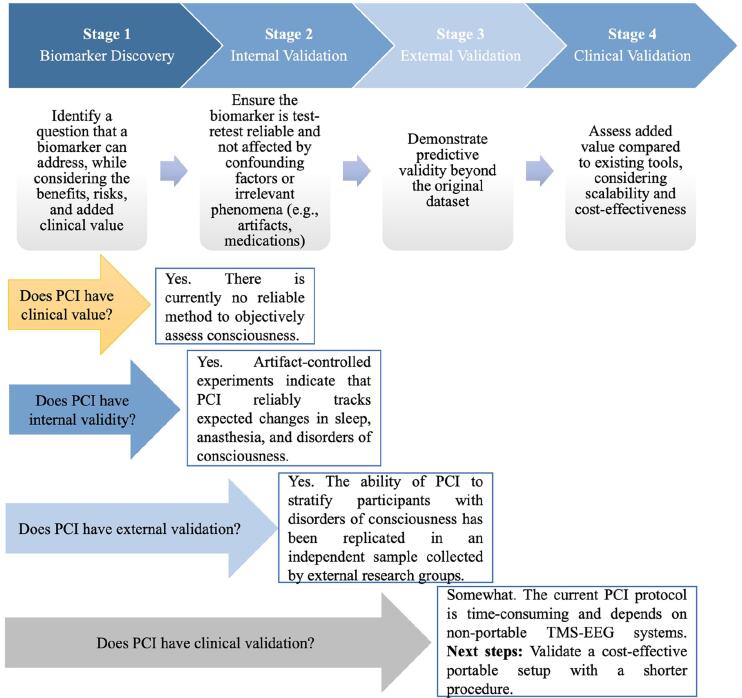
Schematic representation of the four stages of development for biomarkers. The bottom part of the figure shows the work out of TMS-EEG biomarker development for perturbational complexity index (PCI) in the diagnosis of disorders of consciousness (cf. also Section 14). Adapted with permission from (Farzan, 2024).

Given the above considerations, we propose the following series of questions to assess the progress of a given TMS-EEG biomarker on the road to clinical translation (Fig. 7.1) (Farzan, 2024):

Stage 1 (biomarker discovery):

• Is the TMS-EEG biomarker clinically relevant?
  - Does the biomarker answer a relevant clinical question considering the cost/benefit ratio?
  - What are the current clinical procedures?

Stage 2 (internal validation):

● Is the TMS-EEG biomarker a valid measure of the proposed neurophysiological mechanism?
  - Has the potential involvement of sensory activity been assessed and quantified?
  - Have artifacts successfully been avoided during data collection or minimized during data cleaning without impacting the underlying neural signal?
  - Has the neurophysiological mechanism been confirmed for the site of stimulation (i.e., is not extrapolated from another region)?
  - Has the potential for a medication confound been assessed?
● Does the TMS-EEG biomarker have good internal reliability?
  - Is test–retest reliability good-to-high within an individual?

Stage 3 (external validation):

● Does the TMS-EEG biomarker have good external reliability?
  - Are the methods for data collection, analysis and quantification easily and fully reproducible by another research group?
● Is the TMS-EEG biomarker generalizable?
  - Have the findings been confirmed by the same or another research group in an independent data set?

Stage 4 (clinical validation):

● Is the methodological set-up to record the TMS-EEG biomarker achievable in a clinical setting?
  - May require collaboration between researchers and commercial partners to develop integrated TMS-EEG-neuronavigation systems.
  - May require the development of advanced second-order analysis methods to obtain valuable clinical information from reduced channel montage or reduced number of trials, which could crucially simplify and shorten the measurement sessions and as such making TMS-EEG more applicable in a clinical setting.

As an example, we use the above checklist on a proposed TMS-EEG biomarker (PCI for disorders of consciousness as a diagnostic biomarker) to assess progress towards translation (Fig. 7.1).

Stage 1 (biomarker discovery):

• Is the TMS-EEG biomarker clinically relevant?
  - Yes. Clinical evaluation of consciousness following severe brain injuries relies on the observation of appropriate behavioural responses to sensory stimulation; however, this approach may underestimate the state of consciousness in patients who may be conscious but disconnected from the external environment because their sensory, motor, or executive functions are impaired. There is currently no reliable method to objectively assess consciousness. PCI provides an objective metric designed to assess consciousness in participants who cannot communicate.

Stage 2 (internal validation):

• Is the TMS-EEG biomarker a valid measure of the proposed neurophysiological mechanism?
- Yes. Experimental arrangements which target medial brain regions, use adequate noise masking, and use online feedback to optimize coil position, stimulation intensity and masking parameters have demonstrated minimal contribution from muscle activity and sensory potentials to the TMS-EEG signal. Studies in healthy individuals with altered consciousness due to sleep or anaesthesia show reduced PCI as compared to wakefulness and to conscious brain-injured patients (locked-in syndrome, stroke, emergence from minimally conscious state). PCI was able to detect with high sensitivity (94.7 %) in patients in a minimally conscious state (Casarotto et al., 2016).
• Does the TMS-EEG biomarker have good internal reliability?
  - Yes. PCI shows good-to-high test–retest reliability (>0.7) within conscious individuals following stimulation of premotor, motor, and parietal cortex (Caulfield et al., 2020).

Stage 3 (external validation):

• Does the TMS-EEG biomarker have good external reliability?
  - Mostly. Open-source software exists to replicate coil optimization and auditory masking procedures required for high SNR TEPs and to calculate one version of the PCI (state-transition PCI; PCI^ST^), although code for calculating the original PCI which requires source reconstruction and permutation statistics is not currently publicly available. There is currently no consensus on an end-to-end analysis pipeline for PCI measures. However, comparable PCI values have been achieved by other research groups (Sinitsyn et al., 2020).
• Is the TMS-EEG biomarker generalizable?
  - Preliminary evidence suggests yes. The ability of PCI to stratify participants with disorders of consciousness has been replicated in a larger sample by the same research group (Casarotto et al., 2016, Casarotto et al., 2024), and in independent samples collected by external research groups (Bodart et al., 2017). Larger, multicentre clinical trials are now required.

Stage 4 (clinical validation):

• Is the methodological set-up to record the TMS-EEG biomarker achievable in a clinical setting?
  - In some instances, yes. The current protocol for assessing PCI requires 64-channel EEG and stimulation of multiple cortical targets to find the maximum PCI value, which can take several hours in total. Feasibility of this approach has been demonstrated in subacute and chronic care settings and has endorsement for inclusion in clinical application from several clinical associations; however clinical uptake remains minimal. For use in intensive care settings, more portable TMS-EEG equipment is required with a shorter overall protocol (Bai Y et al., 2023a). PCI^ST^ has been demonstrated on 19-channel EEG recordings (reduced from 64-channel recording) but requires validation as a stand-alone protocol.

Based on the above assessment, PCI for disorders of consciousness is in the late stage 3/early stage 4 phase of development, representing an advanced diagnostic biomarker approaching clinical translation. Next steps include clinically validating a portable TMS-EEG setup and a shorter procedure duration to improve clinical viability and larger-scale adoption.

### 7.5 Summary

Over the last several years, the TMS-EEG field has continued to mature. The continuing refinement and availability of methods and the identification of potential confounds has led to a clear pathway for clinical translation. The following sections will review the progress of various TMS-EEG biomarkers on the road to clinical translation and their application to specific brain disorders.

## 8 Developmental and aging characteristics

The brain undergoes profound anatomical and functional changes throughout the lifespan. The first two decades of life are characterized by structural modifications, including region-specific changes in grey matter (initial increase followed by decline) and linear increases in white matter (Giedd, 2008), accompanied by significant functional and structural connectivity changes (Betzel et al., 2014). These developmental processes underlie the acquisition of sensory, motor, and cognitive capacities (Casey et al., 2005).

In contrast, aging is typically associated with grey and white matter volume loss (Markov et al., 2022, Walhovd et al., 2005) and a gradual decline in cognitive (Brito et al., 2023) and motor (Clark, 2019) abilities. Despite these changes, the aging brain maintains significant plasticity, with compensatory processes and neural network adaptations in response to external inputs (Rossini et al., 2013).

This section examines TMS-EEG studies characterizing age-related changes, comparing children/adolescents and older adults to young adults. We also review studies in pediatric patients with various neurological disorders. The results are summarized in Table 8.1, Table 8.2.

**Table 8.1:** TMS-EEG studies of pediatric neurologic and psychiatric disorders.

**Authors**	**Sample**	**Site of Stimulation**	**EEG** **Recordings**	**TMS parameters**	**Measurements/Interventions**	**Key findings**
**Attention Deficit Hyperactivity Disorder (ADHD)**						
*Neurophysiology*						
(Bruckmann et al., 2012)*	20 ADHD 19 HS	Left M1	64 channels SR: 5,000 Hz	Single pulse (monophasic, 105 % RMT)	N100 amplitude and latency at C3, measured at rest, during movement, and during a reaction task time	(1) N100 decreased in ADHD(2) Tendency for decreased latency of N100 in ADHD(3) ADHD: No reduction of N100 during movement preparation and less reduction during movement execution
(D'Agati et al., 2014)*	18 ADHD 19 HS	Left M1	22 channels SR: 5,000 Hz	Single pulse (monophasic, 1 mV)	N100 amplitude and latency at P3, measured at rest & during go/NoGo task	(1) No difference in amplitude of the N100 at rest in ADHD (2) Smaller N100 modulation during a go/NoGo task
*Intervention*						
(Helfrich et al., 2012)*	25 ADHD	Left M1	64 channels SR: 5,000 Hz	Single pulse (biphasic, 110 % RMT) rTMS: 1 Hz (active vs. sham)	N100 amplitude at M1, measured pre, during and post rTMS	(1) N100 reduction during (0–500 pulses) and after active rTMS. No difference with sham rTMS
**Autism Spectrum Disorder (ASD)**						
*Neurophysiology*						
(Jarczok et al., 2016)*	22 ASD 22 HS	Left M1	64 channels SR: 5,000 Hz	Single pulse (monophasic, intensity yielding MEPs of 1 mV)	Connectivity measured as Interhemispheric signal propagation (ISP)	(1) No difference in ISP between groups(2) TMS-evoked activity correlated negatively with age(3) ISP correlated positively with age
*Intervention*						
(Yang et al., 2024)	12 ASD	Left parietal lobe (P3)	32 channels SR: 1,024 Hz	Single pulse (monophasic, 50 % MSO as rMT difficult to assess) rTMS: five 5 s trains at 15 Hz, with 10 min interval between trains for 15 consecutive weekdays	Connectivity measured using adaptive directed transfer function (ADTF) Change in ASD symptoms	Early (60–83 ms):(1) Increased information transmission from bilateral occipital to frontal regions(2) Decrease connections between bilateral frontal and centrotemporal regionsLate (83–1400 ms):(1) Increased frontal to parietal and right posterior temporal connectivity(2) Decreased posterior brain connectivity
**Tourette Syndrome (TS)**						
*Neurophysiology*						
(Schmidgen et al., 2023)	18 MDD 15 HS	Left M1	64 channels SR: 5,000 Hz	Single pulse (biphasic, 110 % rMT and stimulation-intensity slope at 40/60/80 % MSO)	N100 measured during rest, motor preparation, and motor executionN100 response curve (40/60/80 % MSO)	(1) N100 amplitude at 110 % rMT same between groups(2) Age-dependent decrease in both groups(3) TS group had less N100 modulation with stimulation intensity-slope
**Epilepsy**						
*Neurophysiology*						
(She et al., 2024)	18 SeLECTS	Left and right M1	64 channels SR: 25,000 Hz	Single pulse (biphasic; 120 % rMT)	TEP stability: # of pulses required to obtain a TEP similar to one from 100 trials. Considered local (M1) and global (GMFP), in early (<80 ms) and late (>80 ms) time periods.	(1) Early (15–80 ms): Local TEPs need 22 ± 19 pulses. GMFP requires 35 ± 22 pulses(2) Late (80–350 ms): Local TEP needs 17 ± 11 pulses; GMFP 24 ± 17 pulses
*Intervention*						
(Baumer et al., 2020)	14 SeLECTS	Left M1	48 channels SR: 1,000 Hz	Single pulse (biphasic; 120 % rMT)rTMS (1 Hz, 1000 pulses; 85 % rMT)	N100 at baseline and again after 1-Hz rTMS Language & motor learning skills	(1) High-amplitude TEPs at 100 ms that decrease with age(2) No group change in N100 amplitude after 1 Hz rTMS, but individual N100 change associated with language learning scores
(She et al., 2025)	19 SeLECTS	M1 (side with most epileptiform activity)	64 channels SR: 25,000 Hz	Single pulse (biphasic; 120 % rMT)rTMS (1 Hz; 1000 pulses; 90 % rMT vs. 1000 pulses sham rTMS)	M1 P60 and N100; GMFP.Connectivity measured using the weighted phase lag index (wPLI) Epileptiform discharges	(1) TEPs (P60, N100, GMFP) did not change after rTMS(2) Connectivity decrease after active but not sham rTMS(3) Epileptiform discharges decrease after active but not sham TMS.
**Major Depressive Disorder (MDD)**						
*Neurophysiology*						
(Dhami et al., 2020)	45 MDD 20 HS	Bilateral: M1, DLPFC, & IPL	64 channels SR: 20,000 Hz	Single pulse (monophasic, intensity yielding MEPs of 1 mV)	TEPs (all channels; 10–250 ms)Coherence (channel x frequency x time)	(1) Right DLPFC N100 greater in MDD(2) Left DLPFC P200 greater in MDD(3) Right DLPFC stimulation increased connectivity in theta and alpha bands
(Biermann et al., 2022)	36 MDD	Bilateral DLPFC	64 channels SR: 5,000 Hz	Single pulse (biphasic; 120 % rMT)	N100 at DLPFC measured before and after 6-week therapy	(1) DLPFC N100 amplitude decreased after therapy(2) N100 same between hemispheres
*Intervention*						
(Dhami et al., 2021)	16 MDD 16 HS	Bilateral: M1, DLPFC, & IPL	64 channels SR: 20,000 Hz	Single pulse (monophasic, intensity yielding MEPs of 1 mV)TBS (10 bilateral DLPFC TBS sessions daily; iTBS to left and cTBS to right DLPFC)	TEPs (P30, N45, P60, N100, P200) comparison	(1) Left DLPFC: group differences in P30 and P200(2) Right IPL: group difference for P60 and reduction in N45 in MDD group following TBS(3) Left DLPFC: Baseline N45 associated with clinical symptoms
(Dhami et al., 2023)	20 MDD (Trial 1)30 MDD (Trial 2)	Bilateral: DLPFC & IPL	64 channels SR: 20,000 Hz	Single pulse (monophasic, intensity yielding MEPs of 1 mV)TBS (10–20 bilateral DLPFC TBS sessions daily; iTBS to left & cTBS to right DLPFC)	TEP (P30, N45, P60, N100, P200) at 4 sites	(1) Baseline left DLPFC TEPs predict clinical response(2) Larger N45 and smaller P60 amplitudes predict better response

Abbreviations: ADHD, attention deficit hyperactivity disorder; ADTF, adaptive directed transfer function; ASD, autism spectrum disorder; cTBS, continuous theta burst stimulation; DLPFC, dorsolateral prefrontal cortex; EEG, electroencephalography; HS, healthy subjects; iTBS, intermittent theta burst stimulation IPL, inferior parietal lobule; ISI, inter-stimulus interval; ISP, interhemispheric signal propagation; M1, primary motor cortex; MDD, major depressive disorder; MEP, motor evoked potential; MSO, maximum stimulator output; RMT, resting motor threshold; rTMS, repetitive transcranial magnetic stimulation; SeLECTS, self-limited epilepsy with centrotemporal spikes; spTMS, single-pulse transcranial magnetic stimulation; SR, sampling rate; TBS, theta burst stimulation; TEP, transcranial magnetic stimulation-evoked potential; TMS, transcranial magnetic stimulation; TS: Tourette syndrome. * Studies previously included in (Tremblay et al., 2019) retain the same information and format as published in that paper.

**Table 8.2:** TMS-EEG studies of physiological aging.

**Authors**	**Sample**	**Site of stimulation**	**EEG recordings**	**TMS parameters**	**Measurements/ Interventions**	**Key findings**
(Casarotto et al., 2011)	9 young subjects (2F, 7 M) aged 31 ± 4.5 years 9 elderly subjects aged 72 ± 8.4 years (5F, 4 M) (+9 patients with Alzheimer’s disease)	Left superior frontal cortex	60 channels SR: 1,450 Hz	∼ 200 biphasic single pulses SI: 110 V/m ITI 1.5–––1.8 s Neuronavigation	Analyses in the 10–45 ms post stimulus period Grand average TEPs at FC1 Time course of the grand average SCD averaged over the stimulated cortical area	TEP waveform at FCI consisted of 2 peaks within the first 45 ms in both groups The local mean SCD integrated over 10–45 ms did not differ between the young and older subjects
(Ferreri et al., 2017)	12 older subjects (5F, 7 M) mean age 67.6 years 12 young subjects (7F, 5 M) mean age 24.5 years	Left M1	19 channels SR: 2,500 Hz	80 monophasic single pulses SI: 120 rMT ITI 4–6 s Neuronavigation	GMFP (20–350 ms) TEP amplitudes and latencies Current density analysis (MNE)	P30, N45, P60, N100, P180 and N280 peaks were identified in both groups. GMFP reduced in the elderly subjects No latency differences P30 globally larger in the older subjects, but larger in young subjects near the site of stimulation (C3, P3) N45 smaller in the older subjects at C3, P3 and P7 N100 larger at FP1 and F3 in the younger groups, and smaller at Cz compared to the elderly P180 globally larger in the younger subjects N280larger at C3 and smaller at Fp1 and Fp2 in the younger compared to older subjects The current density maps differed between the groups
(Noda et al., 2017b)	12 older subjects (6F, 6 M), mean age 72 ± 9 years 12 younger subjects (6F, 6 M) aged 29 ± 12 years	Left DLPFC	64 channels SR: 20,000 Hz	Monophasic single pulses The number of stimuli not stated ITI not stated SICI and ICF protocols SI: The CS intensity at 80 % of rMT and TS individual 1 mV MEP intensity	TEP amplitude in the baseline and after SICI and ICF at 5 ROIs TEP amplitude change: TEPS evoked by CS & TS / TS.	SICI paradigm: 1) N100 larger in older adults and smaller in younger adults 2) Age correlated negatively with a modulation of P180 and positively with a modulation of N45 ICF paradigm: 1) N45 larger and N100 smaller in older adults and N45 larger in younger adults 2) Age correlated negatively with modulation of N100 TEP
(Noda et al., 2017a)	12 older subjects (6F, 6 M) mean age 72 ± 9 years, 12 younger subjects (6F, 6 M), mean age 39 ± 12 years	Left M1 and left DLPFC (at F5 electrode site)	64 channels SR: 20,000 Hz	100 monophasic single pulses/condition (200 pulses/site) SAI protocol ITI 5 s SI: individual 1 mV MEP intensity	TEP amplitude in the baseline and after SAI TEP amplitude change Other: cognitive tests	P30, N45, P60, N100 and P180 TEPs were identified in both groups M1 stimulation: N45 and N100 components were less modulated by SAI in older subjects DLPFC stimulation: N100 was less modulated by SAI in the older subjects DLPFC-SAI in older subjects correlated with executive function
(Opie et al., 2018)	17 older subjects, mean age 71.4 ± 1.4 years 17 younger subjects mean age 24.2 ± 1.1 years	Left M1	62 channels SR: 2,048 Hz	Monophasic single and paired pulses. 84 stimuli/condition LICI protocol SI: Conditioning and test stimuli 120 % rMT ITI: 5 s with a 10 % variance between trials.	GMFA (area under the GMFA curve for the first 300 ms) TEP amplitudes and latencies LICI: The change in TEP amplitude as the % of the test alone TEP size. Time-frequency analysis	P30, N45, N100 and P180 were identified in both groups GMFA did not differ between the groups After the test stimulus, in the GMFP, latency of P30 decreased and P180 increased, and the amplitude of N45 TEP increased in the older subjects. The spatial distribution of the N100 and P180 differed between the groups LICI of N100 and P180 was increased in older subjects No group differences in the oscillatory activity
(Opie et al., 2020)	17 older subjects (7F, 10 M), mean age 68.3 + -5.6 years 23 young subjects (11F, 12 M) aged 22.3 ± 2.2 years	Left M1	62 channels SR: 2,048 Hz	90 monophasic single pulses/condition SI: 120 % rMT ITI: 5 s with a 10 % variance between trials	TEP amplitude before, during and after fatiguing exercise Other: Fatiguing exercise	P30, N45, N100 and P180 were identified in both groups In older adults, N45 decreased during and after fatigue, and N100 was modified In younger adults, P30 increased after fatigue, N100 decreased during fatigue, and P180 decreased post-fatigue compared with during-fatigue condition
(Goldsworthy et al., 2020)	33 older subjects, mean age 68 ± 7 years 33 young subjects, mean age 22 ± 3 years	Left DLPFC (between F3 and F5 electrodes sites)	57 channels SR: 2,048 Hz	80 monophasic single pulses/condition SI: 120 % rMT ITI: 5 s with a 10 % variance between trials	TEP amplitude and latency at baseline and at 5 and 20 min following rTMS. Other: iTBS, cognitive testing	3 peaks were considered: N40, P60, and N100 No difference in N40 and P60 amplitudes or latencies in younger vs adults N100 latency increased in older participants P60 amplitude increased following iTBS in younger, but not older individualsThe change in P60 was related to performance on a paired associative learning task
(Noda et al., 2021)	12 older subjects (6F, 6 M), mean age 72 ± 9 years 12 young subjects (6F, 6 M), mean age 39 ± 12 years (+ 12 patients with schizophrenia)	Left M1 and left DLPFC	64 channels SR: 20,000 Hz	100 monophasic single pulses/site ITI: not stated SI: individual 1 mV MEP-threshold	TEP amplitudes and latencies at 5 ROIS	P30, N45, P60, N100 and P180 TEPS were elicited in both groups to M1 and DLPFC stimulation M1 stimulation: older subjects had smaller N45 and P180 amplitudes and longer P60 latency at the left central ROI DLPFC stimulation: older subjects’ N45 amplitude decreased at the left frontal ROI and N45 and P60 latencies increased in the right central ROI
(Cespón et al., 2022)	20 older adults (10F, 10 M), mean age 67.0 ± 3.11 years 21 healthy young subjects (16F, 5 M), mean age 22.6 ± 2.59 years	Left parietal cortex (over CP5, angular gyrus/supramarginal gyrus)	57 channels SR: 5,000 Hz	80 single-pulses ITI: 4–5 s SI: 80 % rMT Neuronavigation Simon task before TMS	GMFP (20–100 ms, 101–200 ms, 201–300 ms) TEP amplitudes and latencies over the stimulated area and midline Other: Simon task before TMS (ERP correlates of attentional and inhibitory control processes)	GMFP between 191–200 ms increased in young adults. Older adults’ N45 amplitude decreased and P180 latency increased In older adults, GMFP with N2pc latency recorded in the Simon task
(Bai Z et al., 2023b)	21 older healthy adults (6F, 15 M), mean age 62.8 ± 4.2 years 21 young adults (7F, 14 M), mean age 28.1 ± 3.2 years	Left and right M1	64 channels SR: 5,000 Hz	90 biphasic single pulses/hemisphere SI: 110 % rMT ITI 4–5 s	TEP amplitudes and latencies	N100 amplitude decreased N100 and P180 latencies increased in older subjects
(Perellón-Alfonso et al., 2024)	47 subjects (16F, 31 M), mean age 54.8 ± 7.1 years	Left DLPFC and left IPL	64 channels SR: 1,000 Hz	120 biphasic single pulses/site SI: 120 % rMT ITI: 4–6 s Neuronavigation	Perturbation-based cortical excitability (from 15 to 35 ms and from 160 to 240 ms) Other: Spontaneous EEG Plasma concentrations of p-tau181 and NfL	TMS-EEG markers are associated with blood p-tau181, particularly in older individuals

Abbreviations: DLPFC, dorsolateral prefrontal cortex; ERP, event-related potential; F, female; GMFA, global mean field amplitude; GMFP, global mean field power; ICF, intracortical facilitation; IPL, inferior parietal lobule; iTBS, intermittent theta burst stimulation; ITI, intertrial interval; LICI, long latency intracortical inhibition; M, male; M1, primary motor cortex; MEP, motor evoked potential; MNE, minimum norm estimate; NfL, neurofilament light chain; rMT, resting motor threshold; ROI, region of interest; SAI, short-latency afferent inhibition; SI, stimulus intensity; SICI, short interval intracortical inhibition; SR, sampling rate; TEP, TMS-evoked potential.

### 8.1 TMS-EEG in healthy children

**Fig. 8.1:**
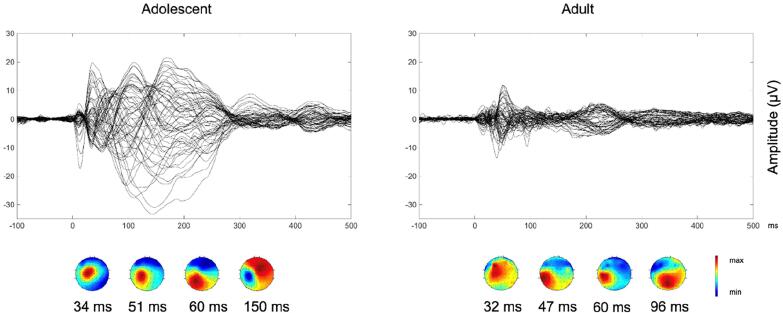
Age-typical TEPs and topographical maps to suprathreshold left primary motor cortex stimulation in a representative individual in two age groups (16-year-old adolescent and 30-year-old adult). The top row depicts butterfly plots, and the bottom row shows topographical maps of the main TEPs for the 60 recorded channels. A suprathreshold (110 % MT) TMS pulse was targeted to the left primary motor cortex and given at time = 0 ms.

TEP components show pronounced maturation during childhood and adolescence, with amplitudes inversely correlating with age. Children have a remarkably prominent N100 component in response to M1 stimulation, reaching > 50 µV at around 7 years (Bender et al., 2005, Määttä et al., 2017). This prominent local N100 response possibly reflects immature connectivity patterns and/or immature GABAergic circuits (Määttä et al., 2017). The differences between developing and adult TMS-EEG responses are presented in Fig. 8.1.

Since RMT also inversely correlates with age, children younger than 6 years often have an RMT exceeding maximum stimulator output (MSO), typically allowing only subthreshold TEPs to be measured. Unlike adult MEPs, which show steep amplitude increases around RMT, pediatric TEPs show linear increases at subthreshold intensities (Bender et al., 2005). When adults and children are stimulated at the same intensity, children still demonstrate significantly higher N100 amplitudes, suggesting that higher TEP amplitudes cannot be attributed solely to stronger stimulation due to higher RMT (Baumer et al., 2020, Bender et al., 2005, Määttä et al., 2017).

Higher amplitude TEPs in children lead to better signal-to-noise ratios, requiring fewer stimuli than in adults to evoke reliable TEPs. For local late TEP responses (e.g., TEPs at C3, C5, CP5 for left M1 stimulation > 80 ms), even 20 trials can be sufficient for many research questions (Bender et al., 2005, She et al., 2024), while 40–50 stimuli may suffice for overall TEP topography and early components (She et al., 2024). N100 amplitude is reduced during tasks requiring motor preparation (Bender et al., 2005, Schmidgen et al., 2023) or movement execution (Schmidgen et al., 2023), so task-based paradigms may require more stimuli.

Most pediatric studies have focused on M1 stimulation (Baumer et al., 2020, Bender et al., 2005, Bruckmann et al., 2012, D'Agati et al., 2014, Jarczok et al., 2016, Määttä et al., 2017, Moliadze et al., 2018, Schmidgen et al., 2023, She et al., 2025). However, TEPs can also be evoked in other brain regions, such as the DLPFC (Biermann et al., 2022) or dorsal premotor cortex (Määttä et al., 2019). While functional localization of the TMS hot spot works well in children, neuronavigation presents additional challenges. Auditory masking is feasible (Määttä et al., 2017, She et al., 2024), but some children tolerate it poorly, so alternative approaches include correction of auditory evoked potentials by ICA or dipole analysis (Bruckmann et al., 2012). While repeated single-pulse stimulation (>5 s interstimulus interval) may produce slight potentiation (Bender et al., 2005), 1 Hz inhibitory rTMS of M1 leads to decreased N100 in children with attention deficit hyperactivity disorder (ADHD), reaching a plateau after approximately 400–500 stimuli (Helfrich et al., 2012), suggesting TEPs may be used to monitor rTMS effects in children.

### 8.2 TMS-EEG in pediatric disorders

TMS-EEG has been used to investigate psychiatric and neurologic disorders with onset in childhood or adolescence. Several studies have also used single-pulse TMS to assess treatment-related changes associated with both rTMS interventions and other therapies.

#### 8.2.1 Attention deficit hyperactivity disorder (ADHD)

ADHD is characterized by reduced inhibition, leading to difficulties with attention, impulsivity, and hyperactivity. TMS-EEG studies of M1 have found decreased N100 amplitudes and latencies across ages (Bruckmann et al., 2012), though this finding was not replicated with different methods (D'Agati et al., 2014). Children with ADHD show impaired modulation of M1 inhibition: during both response preparation and overt movement, neurotypical children demonstrate larger reductions in N100 amplitude, whereas those with ADHD show only non-significant changes (Bruckmann et al., 2012). Children with ADHD also exhibit decreased N100 modulation in go/no-go tasks (D'Agati et al., 2014). A sham-controlled trial of 1 Hz rTMS to M1 showed a steady decrease in N100 amplitude through about 500 pulses. Single-pulse TMS applied before versus after rTMS, but not after sham rTMS, confirmed stimulation-induced changes in N100 amplitude, with source analysis indicating changes at the stimulated M1 (Helfrich et al., 2012).

#### 8.2.2 Tourette syndrome (TS)

TS is a pediatric-onset disorder characterized by motor and vocal tics involving the basal ganglia and M1. Prior TMS-EMG investigations found alterations in GABAAR-mediated inhibitory circuits. To test whether GABABR-mediated inhibitory circuits were also disrupted, a study of young adolescents with and without TS assessed N100 differences during external modulation (comparing different TMS intensities) and internal modulation (comparing different behavioral tasks: rest, movement preparation, and execution) (Schmidgen et al., 2023). Both baseline N100 and age-related reduction in N100 amplitudes were similar between groups. However, N100 in adolescents with TS showed reduced responsiveness to both internal and external modulation compared to controls. Specifically, neither the expected increase in N100 amplitude with higher stimulation intensities nor the reduction during movement preparation/execution was as large in children with TS, suggesting reduced inhibitory “reserve” (Schmidgen et al., 2023).

#### 8.2.3 Autism spectrum disorder (ASD)

ASD is characterized by impaired social communication and interaction, with studies suggesting reduced callosal connectivity. A TMS-EEG study testing the hypothesis that callosal connectivity (measured via interhemispheric signal propagation) is reduced in ASD found no difference between individuals with ASD and typically developing controls aged 10–21 years (Jarczok et al., 2016). An open-label trial evaluating left parietal rTMS found both improved clinical symptoms and altered brain connectivity, measured by applying an adaptive directed transfer function (ADTF) to EEG following single TMS pulses, with strengthened long-range connections and weakened short-range connections (Yang et al., 2024).

#### 8.2.4 Pediatric epilepsy

Epilepsy, a disorder of spontaneous seizures, involves disrupted excitation-inhibition balance and connectivity. Studies of children and young adolescents with self-limited epilepsy with centrotemporal spikes (SeLECTS) – an epilepsy that arises from M1 – found very high RMT, often exceeding MSO, even in medication-naive children, potentially reflecting the known M1 pathophysiology. As with healthy children, the M1 N100 amplitude decreases with age (Baumer et al., 2020). Studies measuring the impact of 1 Hz rTMS to the M1 found that TEPs did not change at the group level (Baumer et al., 2020, She et al., 2025). However, individual N100 amplitude changes induced by rTMS were associated with language and motor learning performance, suggesting that response to rTMS may be a potential marker of brain plasticity required for rapid learning (Baumer et al., 2020).

#### 8.2.5 Major depressive disorder (MDD)

Studies suggest TEPs may be important biomarkers of treatment response in adolescents with MDD. A study of hospitalized adolescents undergoing whole-body vibration strength training found that after therapy, both depressive symptoms and DLPFC N100 amplitude decreased; these two changes were correlated at a trend level, suggesting therapeutic effects were mediated by reduced inhibitory tone (Biermann et al., 2022). A series of three studies focused on youth enrolled in TBS trials to the bilateral DLPFC (iTBS to the left and cTBS to the right DLPFC) who, along with age-matched controls, underwent single-pulse TMS-EEG to the bilateral M1, DLPFC, and inferior parietal lobule (IPL) before and after TBS therapy (or separated by 2 weeks for controls). At baseline, right DLPFC stimulation evoked larger N100 and greater connectivity (measured by coherence) while left DLPFC stimulation evoked larger P200 amplitudes and P30 amplitudes (Dhami et al., 2021, Dhami et al., 2020) in the MDD group. Left DLPFC TEPs were particularly informative: larger baseline N45 TEPs and smaller P60 TEPs indicated better treatment outcomes, suggesting that greater baseline cortical inhibition is associated with better response to TBS therapy (Dhami et al., 2023). Thus, changes in the E/I balance may be important for treatment response in MDD.

### 8.3 TMS-EEG in healthy aging

TMS-EEG is well-suited for evaluating functional and connectivity changes with aging. TMS-EEG responses may be affected by potentially higher motor thresholds and a larger coil-to-cortex distance in older adults. While higher TMS intensities (that are typically set in relation to motor threshold rather than TEP threshold) cause larger TEP amplitudes (Komssi et al., 2004, Lioumis et al., 2009, Saari et al., 2018), a larger coil-to-cortex distance may cause higher motor threshold and lower E-field strength (Danner et al., 2012), but the combined impact of TMS intensity and coil-to-cortex distance on TMS-EEG responses has not yet been systematically explored. TMS-EEG studies in geriatrics remain relatively few, with mixed results.

The differences in TMS-evoked EEG responses between young and older adults are subtle. Both age groups show similar TEP waveforms with the typical P30-N45-P60-N100-P180 sequence after motor (Bai Z et al., 2023b, Ferreri et al., 2017, Noda et al., 2021, Noda et al., 2017a, Opie et al., 2020, Opie et al., 2018), frontal (Noda et al., 2021, Noda et al., 2017a, Noda et al., 2017b), and parietal cortex stimulation (Cespón et al., 2022). However, peak amplitudes, topographies, and latencies vary between age groups, as does the responsiveness to experimental paradigms.

#### 8.3.1 Motor cortex stimulation

TMS-EEG studies of M1 suggest age-related alterations in cortical excitability, though findings are inconsistent. (Ferreri et al., 2017) found decreased GMFP, higher P30 amplitude, and smaller P180 amplitude in older adults, with regional differences in P30 and N45 amplitudes but no latency differences. Noda and colleagues reported that in older adults, the N45 and P180 peaks in the left central region were significantly smaller than in young adults (Noda et al., 2021). Another study found reduced N100 amplitude and prolonged N100 and P180 latencies in older subjects (Bai Z et al., 2023b). These results were interpreted as reflecting age-related alterations in cortical excitability and possibly compensatory activation. Contrary to these findings, Opie and colleagues reported no difference in GMFP between groups, but with larger N45 amplitude in older subjects and latency differences (shorter P30 and longer P180 latency) (Opie et al., 2018). The authors interpreted this as increased M1 excitability and potentiated GABAAR-mediated inhibition (Opie et al., 2018).

#### 8.3.2 Frontal cortex stimulation

Three studies using different methodologies assessed frontal cortex TEPs in aging. (Casarotto et al., 2011) found that early TEPs (10–45 ms) to left superior frontal cortex stimulation did not differ between younger and older adults when using standardized stimulation intensity. Goldsworthy and colleagues, also found no difference in N40 and P60 amplitudes or latencies in younger vs adults when stimulation left DLPFC, although N100 latency was longer in older participants (Goldsworthy et al., 2020). Interestingly, P60 TEP amplitude was increased following iTBS in younger, but not older individuals, suggesting an age-related decline in prefrontal plasticity. The change in P60 was related to performance on a paired associative learning task, indicating a reduction in plasticity may relate to age-related cognitive decline. In contrast, Noda and colleagues found that in older participants, DLPFC stimulation elicited smaller N45 peaks in the left frontal region and longer N45 and P60 latencies in the right central region, potentially reflecting age-related neurophysiological changes (Noda et al., 2021).

#### 8.3.3 Experimental paradigms

Four studies assessed TEP modulation in experimental paradigms. Studies examining paired-pulse TMS-EEG paradigms found age-related differences in SICI and intracortical facilitation (ICF) protocols (Noda et al., 2017b) as well as in LICI (Opie et al., 2018). Additionally, fatiguing exercise affected TEP peaks differently in young versus older participants (Opie et al., 2020). These findings suggest that there are cortical excitability differences between young and older individuals, probably due to differences in GABA-receptor mediated inhibitory activity. (Noda et al., 2017a) reported that in older subjects the M1 N45 and N100, and the DLPFC N100 were less modulated than in younger subjects by short-latency afferent inhibition (SAI), a TMS-EMG marker of central cholinergic tone (Di Lazzaro et al., 2000). DLPFC SAI modulation of P60 and N100 in older individuals was directly correlated with executive function. These results suggest that modulation of TEPs by SAI in DLPFC may be a marker of central cholinergic tone.

Research has also investigated how age-related TMS-EEG alterations after parietal cortex stimulation correlate with cognitive functions (Cespón et al., 2022). The results demonstrated that GMFP values were decreased in older adults and correlated with altered cognitive event-related potentials, suggesting that age-related changes in cortical excitability may be dysfunctional (Cespón et al., 2022). Finally, a correlational study showed that TMS-EEG-based excitability markers were associated with blood p-tau181, a neuropathological hallmark of tauopathies, in healthy middle-aged subjects (Perellón-Alfonso et al., 2024). According to this research, cortical excitability might be a potential biomarker for the early or preclinical detection of Alzheimer’s disease (Perellón-Alfonso et al., 2024).

### 8.4 Summary and future directions

TMS-EEG offers a valuable tool for investigating neurophysiological changes across the lifespan. This review highlights several key findings in both typical and atypical development. Children show distinctive TEPs, with prominent N100 components in response to motor cortex stimulation that diminish with age, potentially reflecting the maturation of inhibitory circuits and connectivity. TMS-EEG investigations of various pediatric disorders have uncovered disturbances in inhibitory dynamics. While subtle, aging-related changes in TEPs reflect alterations in E/I balance and connectivity, and some of the findings are suggestive of compensatory mechanisms. TMS-EEG measures show promise as biomarkers for both diagnostic purposes and treatment response prediction, particularly in pediatric populations and potentially in aging. Future research would benefit from longitudinal studies tracking TMS-EEG measures across development and aging to better understand trajectories of change. Additionally, standardization of methodologies across research groups and increased application of TMS-EEG techniques would help resolve inconsistencies in the literature, particularly in aging studies, which remain relatively sparse. A unique consideration in pediatric TMS studies is that historically, some institutions have restricted TMS studies in children due to theoretical risks. However, substantial evidence now supports the safety of TMS, rTMS, and TBS in children (Zewdie et al., 2020), highlighting the importance of utilizing these powerful neurophysiologic and therapeutic techniques to better understand typical development and neuropsychiatric disorders in this population.

## 9 Depression

### 9.1 Introduction

Studies investigating the translational utility of TMS-EEG in Major Depressive Disorder (MDD) and Treatment-Resistant Depression (TRD) have grown substantially since the previous TMS-EEG review in 2019 (Tremblay et al., 2019). Most studies focused on the DLPFC. While TEPs were originally characterized in M1, similar deflections are observed in prefrontal stimulation with peaks around 25, 40, 60, 100 and 180 ms (Kähkönen et al., 2005a, Lioumis et al., 2009). However, latency, amplitude, and neurophysiological interpretation may vary depending on the specific cortical target and underlying circuitry (e.g., (Rosanova et al., 2009). In the following section, TEPs have been quantified in the DLPFC unless otherwise specified. As a general comment, the available studies show a substantial degree of heterogeneity of findings, in particular with regard to the early TEPs, while findings on later TEPs are more consistent across studies. The reasons for these disparities are not clear but may be related to differences in the stimulation parameters (cf. Table 9.1).

**Table 9.1:** TMS-EEG findings in major depressive disorder.

**Authors**	**Sample**	**Region(s) of interest**	**EEG recordings**	**TMS parameters**	**Measurements / Intervention**	**Key findings**
**Neurophysiology**						
(Li et al., 2025)	59 MDD58 HC	Left DLPFC	64 channels SR: 20,000 Hz	Single pulse (biphasic, 100 % RMT)	TEP(P30, N45, P60, N100, P180, N280)	1) N100 and N280 increased in MDD (trend-level)2) Smaller P60 and larger N100 and P280 were associated with more severe depressive symptoms
(Noda et al., 2024)	60 MDD60 HC	Left DLPFC	64 channels SR: 3,000 Hz	Single pulse (monophasic, 120 % RMT)	Power spectrumPhase synchronization (wPLI) Phase-amplitude couplingMachine Learning (analyses over 17 frontal electrodes)	1) Alpha phase synchronization decreased in left DLPFC (F5-AF3; AFz-F5) 2) Theta phase synchronization decreased in left and right DLPFC (AF3, F8) 3) AI model successfully discriminated MDD from HC when combining rest-EEG with TMS-EEG metrics with AUC = 0.922
(Niu et al., 2024)	34 MDD36 HC	Left DLPFC	64 channels SR: 2,500 Hz	Single pulse (biphasic, 110 % RMT)	TEP (P30, N45, P60, N100, P200, N280, P380) Trial-by-Trial Variability (TTV)	1) TTV in the gamma band (32–64 Hz) decreased, that negatively correlated with depression severity 2) TTV in the delta band (1–2 Hz) increased 3) P200 amplitude increased in MDD
(Li et al., 2024)	133 MDD76 HC	L-DLPFC, F3 electrode site	64 channels SR: 25,000 Hz	Single pulse (biphasic, 110 % RMT)	TEP (P30, N45, P60, N100, P180 − all TEPs assessed in whole brain with GMFA) GMFA-AUC	1) P180 amplitude decreased 2) GMFA-AUC decreased 3) P180 amplitude decrease correlated with increased severity of anxious and depressive symptoms 4) P30 amplitude decrease correlated with increased cognition
(Kaneko et al., 2024)	60 TRD30 HC	Left DLPFC	64 channels SR: 3,000 Hz	Single pulse (monophasic, 120 % RMT)	TEP (early vs. late) ERSP Pre and post DLPFC PAS	1) Both groups exhibited increases in early TEPs after PAS, but TEP changes at 30–40 ms were smaller in TRD 2) Gamma power increased after PAS in the HC group but decreased in the TRD group.
(Pantazatos et al., 2023)	17 MDD11 HC	Left DLPFC	36 channels (MR-compatible bipolar EEG system) SR: 488 Hz	Single-pulse TMS (biphasic, 120 % RMT)	TMS-Evoked Functional Connectivity (FC): fMRI-derived FC between DLPFC and ACC subregionsDynamic Causal Modeling (DCM) : Analyzed top-down vs. bottom-up connectivity TMS-Evoked BOLD Activation	1) DLPFC-SCG functional connectivity increased during the rising phase of alpha waves in both HC and MDD groups 2) DLPFC-SCG functional connectivity decreased during the falling phase of alpha waves in both HC and MDD groups 3) MDD group lacked phase-dependent top-down modulation of DLPFC-SCG 4) SCG/ACC hyperactivation, positive only feedback loop 5) Increased rACC BOLD activation in MDD during specific alpha phases (not seen in HC)
(Li X et al., 2023)	41 MDD 42 HC	Left DLPFC	64 channels SR: 25,000 Hz	Single pulse (monophasic, 100 % RMT)	TEP (P30, N45, P60, N100, P180)	1) P60 amplitude decreased compared with HC, negatively correlated with depression severity2) Regression analysis showed that P60 significantly predicted depressive symptoms
(Han et al., 2023)	53 MDD (28 active, 25 sham) 64 HC	Left DLPFC	64 channelsSR: 25,000 Hz	Single pulse (monophasic, 100 %RMT)	TEP (P30, P60, N100, P180) LMFA-AUCEffective Connectivity (SCD, SCS, SOE) *10 Hz-rTMS for 2 weeks*	1) P60/N100 decrease in MDD vs. HC 2) LMFA-AUC (164–215 ms) decrease in MDD vs. HC 3) Hypoactivity (SCD) in DLPFC, OFC and hippocampus, and decreased low frequencies (SOE) 4) Decreased connectivity (SCS) between DLPFC and OFC in MDD
(Wada et al., 2022)	60 TRD30 HC	Left DLPFC	64 channelsSR: 3,000 Hz	Single pulse (monophasic, 120 %RMT)	GMFP LMFP ERSPdSPM (current density) Connectivity in 7 networks	1) Decreased theta power in TRD vs HC 2) Decreased signal propagation from left DLPFC to the salience network in the theta and alpha bands (100–500 ms) 3) Decreased signal propagation positively correlated with cell-specific gene expression of oligodendrocytes 4) Decreased signal propagation negatively correlated with cell-specific gene expression of cornu ammonis 1 pyramidal neurons, somatosensory cortex pyramidal neurons and interneurons
(Dhami et al., 2021)	16 MDD youth (16–24 yrs)16 HC	Bilateral DLPFC Bilateral M1 Bilateral IPL	64 channels SR: 20,000 Hz	Single pulse (monophasic, 1 mV)	TEP (P30, N45, P60, N100, P180) *2-week bilateral TBS*	1) P30 decrease and P200 increase in left DLPFC in MDD vs HC 2) P60 increase in right IPL in MDD vs HC
(Hill et al., 2021)	38 MDD22 HC	Left DLPFCLeft M1	64 channels SR: 20,000 Hz	Single pulse (monophasic, 1 mV)	Oscillatory power *Acute course of ECT (n = 14) or MST (n = 24)*	1) Oscillatory power increase within delta, theta, and alpha bands in left DLPFC, while no difference in M1
(Dhami et al., 2020)	45 youth TRD20 youth HC	Bilateral DLPFC Bilateral M1Bilateral IPL	64 channels SR: 20,000 Hz	Single pulse (monophasic, 1 mV)	TEP (P30, N45, P60, N100, P200) Mean coherence connectivity *4 weeks daily left iTBS*	1) N100 increase right DLPFC; classified TRD with 79 % accuracy (82 % sensitivity, 78 % specificity) 2) P200 increase left DLPFC; classified TRD with 69 % accuracy (70 % sensitivity, 83 % specificity) 3) P200 positively correlated with anhedonia symptoms 4) Increase of left DLPFC connectivity in the alpha and theta range with multiple regions including left rostral anterior cingulate cortex, left angular gyrus, and right precuneus; classified TRD with 83 % accuracy (83 % sensitivity, 82 % specificity)
(Voineskos et al., 2019)	30 TRD30 HC	Left DLPFCLeft M1	64 channels SR: 20,000 Hz	Single pulse (monophasic, 1 mV)	TEP(N45, P60, N100) GMFA-AUC	1) N45, P60 and N100 increase in DLPFC in MDD vs HC 2) N45 increase in DLPFC predicted illness state with 76.6 % accuracy (80 % sensitivity, 73.3 % specificity) 3) GMFA-AUC increase in MDD
(Miyauchi et al., 2019)	10 MDD	M1Occipital	27 channels SR: 1,000 Hz	Single pulse (biphasic, 95 % RMT)	PLV, PLF	1) PLV between visuo-motor areas in alpha band negatively correlated with depression severity
(Hadas et al., 2019)	30 TRD 30 HC	Left DLPFC	64 channels SR: 20,000 Hz	Single pulse (monophasic, 1 mV)	SCS, SCD(DLPFC-SGC connectivity)	1) SCS (N100, P200) increase in MDD vs HC 2) SCD in ACC (P30, N100, P200) increase in MDD vs HC 3) SCD in ACC differentiated MDD vs controls with 77 % accuracy (70 % sensitivity, 83 % specificity)
(Noda et al., 2018)*	29 MDD28 HC	Left DLPFC	64 channels SR: 20,000 Hz	Single pulse (monophasic, 1 mV)PAS: median nerve (3x threshold) and DLPFC TMS (1 mV); ISI of 25 ms	TEP power ERSP Plasticity measured with PAS	1) Decreased PAS-induced plasticity in MDD vs HC as measured with TEP power 2) Decreased PAS-induced plasticity in MDD vs HC as indexed with gamma, theta and delta power, and theta-gamma coupling
(Canali et al., 2015)*	12 MDD 12 BPD 12 SCZ12 HC	Premotor cortex	60 channels SR: 1,450 Hz	Single pulse (> 90 V/m) Neuro-navigation	ERSPNatural frequency	1) Decreased main frequency in patients with BPD, MDD and SCZ (11–27 Hz) in comparison with HC (beta-gamma: 21–50 Hz)
**Intervention**						
*Non-convulsive brain stimulation*						
(Sheen et al., 2024)	23 MDD	Right DLPFC	16 channels SR: 512 Hz	1 Hz pulses (biphasic, 120 %RMT)	TEP (N100) 1 Hz-rTMS, accelerated protocol (8 daily sessions for 5 days)	1) Increased baseline N100 amplitude was strongly associated with greater symptom improvement 2) N100 amplitude decreased from baseline to follow-up, but change was not significant 3) Responders had larger N100 baseline amplitude compared to non-responders 4) Decrease in baseline N100 amplitude correlated with smaller post-treatment improvement
(Zangen et al., 2023)	169 MDD(multicenter study)	Lateral or Medial PFC	32 or 64 channels SR: 2,048 Hz	18 Hz trains (biphasic pulses, 120 % RMT)	Power spectrum measured during inter-train intervals in the first treatment session. 24 treatment sessions over 6 weeks, 18 Hz DTMS directed to either lateral PFC (H1 coil group) or medial PFC (H7 coil group)	1) Within treatment activity in the alpha band, low-gamma band, and low-gamma/alpha ratio over the medial PFC were significantly correlated with the clinical improvement following medial PFC (H7 coil group) treatment.2) Within treatment, asymmetric PFC activity in the alpha band significantly correlated with the clinical improvement following both lateral (H1 coil group) and medial (H7 coil group) treatment.
(Han et al., 2023)	74 MDD (28 active, 25 sham) 64 HC	Left DLPFC	64 channelsSR: 25,000 Hz	Single pulse (monophasic, 100 %RMT)	TEP (P30, P60, N100, P180) LMFA-AUCEffective Connectivity (SCD, SCS, SOE) 10 Hz-rTMS for 2 weeks	1) Increase in LMFA-AUC (150–185 ms) post active rTMS (vs sham), which was associated with relief of depressive symptoms 2) Increased activation (SCD) in DLPFC, OFC and hippocampus post active rTMS
(Strafella et al., 2023)	114 TRD	Left DLPFC	64 channels SR: 20,000 Hz	Single pulse (monophasic, 120 %RMT)	TEP (GMFA) (N45, N100) GMFA-AUCTwo groups (30 sessions over 6 weeks):A) Separated iTBS schedule (54-minute interval between two 600-pulse iTBS sessions)B) Continuous iTBS schedule (0-minute interval between two 600-pulse iTBS sessions)	1) Decrease in N100 amplitude from baseline to posttreatment across both iTBS schedules 2) Decrease in N100 amplitude in responders post-iTBS vs non-responders 3) Increase in post-treatment N45 amplitude in responders vs. non-responders 4) Baseline N100 amplitude predicted post-iTBS depression scores 5) Increased baseline GMFA-AUC predicted posttreatment depression scores
(Li et al., 2022)	40 MDD	Left DLPFC	32 channels SR: 20,000 Hz	Single pulse (monophasic, 120 %RMT); LICI (ISI: 100 ms)	TEP (P30, N45, P60, N100)LICI-AUC (50–150 ms) FDG-PET Group A: iTBS Group B: 10-Hz rTMSGroup C: Sham iTBS or sham rTMS	1) P30 correlated with glucose metabolism in limbic regions (amygdala, hippocampus, parahippocampus) 2) LICI correlated with metabolism in limbic regions, occipital cortex, and temporal cortex 3) Decrease in P60 post 10-Hz rTMS 4) Decrease in LICI post-iTBS
(Dhami et al., 2023)	50 MDD (20 MDD in RCT1 and 30 MDD in RCT2)	Bilateral DLPFC Bilateral inferior parietal lobule (IPL) (used as control sites)	64 channels SR: 20,000 Hz	Single pulse (monophasic, 1 mV)	TEP (P30, N45, P60, N100, P200) RCT1: 2-week bilateral TBSRCT 2: 4-week bilateral TBS combined with cognitive exercise	1) Baseline left DLPFC N45 and P60 predicted treatment response 2) Increased N45 and decreased P60 at baseline were associated with better TBS response
(Dhami et al., 2021)	16 MDD youth(16–24 years) 16 HC	Bilateral DLPFC Bilateral M1Bilateral IPL	64 channels SR: 20,000 Hz	Single pulse (monophasic, 1 mV)	TEP (P30, N45, P60, N100, P180) 2-week bilateral TBS	1) Baseline N45 in DLPFC associated with decrease in symptoms post TBS 2) Decrease in N45 post-TBS in right IPL 3) Right IPL – right DPLFC connectivity at baseline correlated with magnitude of N45 change in right IPL
(Voineskos et al., 2021)	30 TRD (21 active, 9 sham)	Left DLPFCLeft M1	64 channels SR: 20,000 Hz	Single pulse (monophasic, 1 mV)	TEP (N45, P60, N100) GMFA-AUC 6 weeks of active unilateral or bilateral rTMS (10-Hz left, 1-Hz right), or sham rTMS	1) Decrease in N45 and N100 after active rTMS2) N100 change correlated with symptom improvements
(Eshel et al., 2020)	16 TRD active12 TRD sham	Right and left DLPFCLeft V1	64 channels SR: 2,048 Hz	Single pulse (120 %RMT)	TEP (P30, P60, N100, P200) 4 weeks of daily 10 Hz active or sham rTMS in DLPFC	1) Decrease in P30 in left frontal and parietal regions after active vs. sham2) Positive correlation between P30 change and symptom reduction in active group
(Hadas et al., 2019)	43 TRD (26 active, 17 sham)	Left DLPFC	64 channels SR: 20,000 Hz	Single pulse (monophasic, 1 mV)	SCS, SCD (DLPFC-SGC connectivity)Pre and post 30 sessions of rTMS (active vs sham)	1) Decrease in SCS (P200) and SCD (P200) following active vs. sham rTMS
(Pellicciari et al., 2017)*	1 TRD	Bilateral DLPFC	62 channels SR not reported	Single pulse (90 % RMT)	Prefrontal oscillatory activity Pre/Post: 10 sessions of left iTBS/right cTBS	1) Pre-treatment: asymmetry of DLPFC cortical activity (left alpha, right beta and gamma) 2) Post-treatment: decrease in left-hemispheric theta and alpha, increase in beta/gamma; increase in right-hemispheric alpha
*Convulsive brain stimulation*						
(Hill et al., 2021)	38 MDD	Left DLPFCLeft M1	64 channels SR: 20,000 Hz	Single pulse (monophasic, 1 mV)	Oscillatory powerAcute course of ECT (n = 14) or MST (n = 24)	1) Increase in power in delta and theta bands in DLPFC post-MST 2) Decrease in delta, theta, and alpha power in DLPFC post-ECT 3) Decrease in delta and theta power in M1 post-ECT 4) Correlation between change in alpha power and symptom improvements
(Hadas et al., 2020)	31 TRD	Left DLPFC	64 channels SR: 20,000 Hz	Single pulse (monophasic, 1 mV); LICI (ISI: 100 ms)	SGC, SCS24 sessions of MST or until remission achieved	1) Decrease in SCS between DLPFC and SGC; correlated with depressive symptom improvements 2) Decrease in SCD in hippocampus after treatment; correlated with memory changes post-MST
(Miyauchi et al., 2019)	10 MDD	M1 Occipital	27 channels SR: 1,000 Hz	Single pulse (biphasic, 95 % RMT)	PLV, PLFPre and post ECT (bilateral, 2-3x weekly)	1) Negative correlation between changes in PLV in the alpha band and symptom improvement2) Post-treatment latency in differences in PLF peak between visual-motor areas were correlated with symptom severity
(Sun et al., 2018)*	23 TRD	Left DLPFC, Left M1	64 channels SR: 20,000 Hz	Single pulse (monophasic, 1 mV); LICI (ISI: 100 ms)	LICI-AUC CEA24 sessions of MST or until remission achieved	1) Increase in CEA post MST, no correlation with change in SSI score 2) Decrease in LICI correlated with decrease in SSI score 3) LICI change identified resolution of suicidal ideation with a 90 % sensitivity and 88 % specificity
(Sun et al., 2016)*	27 TRD	Left DLPFC, Left M1	64 channels SR: 20,000 Hz	Single pulse (monophasic, 1 mV); LICI (ISI: 100 ms)	TEP (N100) LICI −AUC 24 sessions of MST or until remission achieved	1) Increased LICI and N100 in DLPFC at baseline associated with increase in SSI score change post-MST 2) N100 predicted remission of suicidal ideation post-MST with 84 % accuracy (80 % sensitivity, 89 % specificity) 3) Combined N100 and LICI predicted remission of suicidal ideation post MST with 89 % accuracy (90 % sensitivity, 89 % specificity)
(Casarotto et al., 2013)*	8 TRD	Left or right superior frontal gyrus	60 channels SR: 1,450 Hz	Single pulse (biphasic, 90–130 V/m)	TEPs (IRA, IRS)Pre and postECT: twice a week with bilateral electrode placement (from 3 to 9 sessions)	1) Increase in IRA in frontal regions after ECT in all patients 2) Increase in IRS of early TEPs after ECT (5 patients)
*Other interventions*						
(Hao et al., 2024)	5 MDD 5 HC	Left DLPFC	23 channels SR: 1,024 Hz	Single pulses (monophasic, 90 % RMT)	Connectivity (ADTF) 14 days of transcranial photobiomodulation (tPBM; 820 nm) to the left frontal pole	1) Increase in connectivity between fronto-central cortex and left posterior temporal cortex
(Biermann et al., 2022)	36 MDD adolescents (12–18yrs)	Left and right DLPFC	64 channels SR: 5,000 Hz	Single pulses (biphasic, 120 % RMT)	TEP (N100)Six weeks of inpatient treatment with additional sports therapy	1) Decrease in N100 post-treatment 2) Decrease in depression severity correlated with decrease in N100 in the left DLPFC
(Canali et al., 2017)*	18 MDE with BPD type I	Middle caudal portion of the superior frontal gyrus (BA6)	60 channels SR: 1,450 Hz	Single pulse (biphasic, 90 V/m)	ERSPPre and post one week of chronotherapy (light therapy + sleep deprivation)	1) No effect of chronotherapy on ERSP
(Canali et al., 2014)*	21 MDE with BPD type I	Bilateral PFC	60 channels SR: 1,450 Hz	Single pulse (biphasic, 90–130 V/m)	TEPs (IRA, IRS, GMFP)Cortical oscillationsPre and during one week of chronotherapy (6 time-points including morning, evening, sleep, sleep deprivation)	1) Increase in TEPs slope values during treatment 2) Higher baseline cortical excitability (GMFP and IRA) in responders than non-responders 3) Responders showed lower increase in EEG theta power after sleep deprivation

Abbreviations, ACC, anterior cingulate cortex; ADTF, adaptive directed transfer function; AUC, area under the curve; Brodmann area; BDP, bipolar disorder; CEA, cortical evoked activity; cTBS, continuous theta burst stimulation; DLPFC, dorsolateral prefrontal cortex; dSPM, dynamic statistical parametric maps; ECT, electroconvulsive therapy; ERSP, event-related spectral power; GMFA, global mean field amplitude; IRA, immediate response area; IRS, immediate response slope; ISI, interstimulus interval; iTBS, intermittent theta bust stimulation; HC, healthy controls; LICI, long-interval intracortical inhibition; M1, primary motor cortex; MDD, major depression disorder; MDE, major depressive episode; MST, magnetic seizure therapy; PAS, paired associative stimulation; PLV, phase locking value; PLF, phase locking factor; RMT, resting motor threshold; SCD, significant current density; SCG, subgenual cingulate cortex, SCS, significant current scattering; SOE, significant oscillatory envelope; SR, sampling rate; TEP, TMS-evoked potential; TRD, treatment resistant depression; wPLI, weighted phase lag index. * indicate articles that were included in the previous review by Tremblay et al. 2019.

### 9.2 TEP findings

*P30 component:* Six studies (Dhami et al., 2020, Han et al., 2023, Li et al., 2024, Li et al., 2025, Li X et al., 2023, Niu et al., 2024) examined the early P30 component in MDD, but only one reported a significant alteration. One study observed reduced P30 amplitude in the left DLPFC of youths with MDD (Dhami et al., 2020), suggesting a possible left-lateralized sodium channel–mediated dysfunction (Darmani et al., 2019, Darmani and Ziemann, 2019, Premoli et al., 2014a) (cf. Section 6). No alterations were found in the right DLPFC, M1, or inferior parietal lobule (IPL). These findings may reflect a youth-specific vulnerability given developmental ion channel differences. In contrast, one other study found no group differences in P30 amplitude in a larger adult sample, but lower whole-brain P30 amplitudes were associated with better cognition and visuospatial skills – suggesting a potential role of P30 in cognitive reserve (Li et al., 2024). Two of five studies (Dhami et al., 2021, Dhami et al., 2023, Eshel et al., 2020, Han et al., 2023, Li et al., 2022) reported significant P30 findings related to treatments. Eshel et al. (2020) showed reduced P30 amplitude following HF-rTMS of the left DLPFC versus sham, with degree of change correlating with symptom improvement. While Li et al. (2022) observed no post-treatment change, they found a positive correlation between P30 amplitude and glucose metabolism in the amygdala, hippocampus, and parahippocampal gyrus (via PET), suggesting P30 may reflect limbic circuit activity.

*N45 component:* Of seven studies (Dhami et al., 2021, Dhami et al., 2020, Li et al., 2024, Li et al., 2025, Li X et al., 2023, Niu et al., 2024, Voineskos et al., 2019), only one reported significant findings. Voineskos et al. (2019) found increased N45 amplitude in the left DLPFC, which classified TRD diagnostic status with 76.6 % accuracy. Although this may indicate altered prefrontal GABA_A_-mediated inhibition (Premoli et al., 2014a, Salavati et al., 2018b) and NMDA receptor function (Belardinelli et al., 2021), five other studies reported null findings, highlighting the need for replication and protocol standardization. Findings linking N45 to treatment response have been more consistent. Of five studies (Dhami et al., 2021, Dhami et al., 2023, Li et al., 2022, Strafella et al., 2023, Voineskos et al., 2021), four reported significant findings. Dhami et al. (2023) found that higher baseline N45 amplitude predicted greater clinical improvement following bilateral sequential TBS. In a younger cohort (16–24 years), the same team found TBS-induced N45 changes in the right IPL, with modulation correlating with baseline IPL-DLPFC fMRI connectivity (Dhami et al., 2021). Strafella et al. (2023) found higher N45 amplitude post-iTBS in clinical responders (vs. non-responders), while Voineskos et al. (2021) showed N45 amplitude reductions following left unilateral or bilateral sequential rTMS. Together, these studies suggest N45 is modifiable by diverse NIBS protocols.

*P60 Component:* Alterations in P60 were reported in four of eight studies (Dhami et al., 2021, Dhami et al., 2020, Han et al., 2023, Li et al., 2024, Li et al., 2025, Li X et al., 2023, Niu et al., 2024, Voineskos et al., 2019). Voineskos et al. (2019) reported increased P60 amplitude in individuals with MDD, whereas Li et al. (2023) observed reduced P60, with lower amplitude correlating with greater symptom severity. In a subsequent study, Li et al. (2025) found no group-level differences but again noted a negative correlation with symptom severity. Han et al. (2023) reported a lower P60/N100 ratio, suggesting altered E/I balance. Although inconsistent, these findings suggest prefrontal excitability dysfunctions in MDD, linked to glutamatergic AMPA receptors activity (Belardinelli et al., 2021). Out of six studies examining P60 in the context of treatments (Dhami et al., 2021, Dhami et al., 2023, Eshel et al., 2020, Han et al., 2023, Li et al., 2022, Voineskos et al., 2021), two found significant results. Dhami et al. (2023) found that a lower baseline P60 predicted better response to bilateral TBS. Li et al. (2022) found that P60 amplitude was significantly reduced following HF-rTMS, but not after iTBS or sham, suggesting P60 may be sensitive to excitatory mechanisms elicited by distinct rTMS protocols.

*N100 component:* Three of nine studies reported increased N100 amplitude in MDD (Bailey et al., 2023c, Dhami et al., 2021, Dhami et al., 2020, Han et al., 2023, Li et al., 2024, Li et al., 2025, Li X et al., 2023, Niu et al., 2024, Voineskos et al., 2019). Li et al. (2025) found greater N100 linked with symptom severity. Voineskos et al. (2019) and Dhami et al. (2020) reported similar increases in left and right DLPFC, respectively. Given its association with GABA_B_ receptor-mediated inhibition (Premoli et al., 2014a, Premoli et al., 2018) (cf. Section 6), elevated N100 may reflect altered or compensatory inhibitory activity across bilateral prefrontal regions in MDD. Alternatively, the changes may reflect differences in sensory processing given the contribution of peripherally-evoked potentials to the N100. Of nine studies examining the N100 component following interventions (Dhami et al., 2021, Dhami et al., 2023, Eshel et al., 2020, Han et al., 2023, Li et al., 2022, Sheen et al., 2024, Strafella et al., 2023, Sun et al., 2016, Voineskos et al., 2021), four reported significant findings. Sheen et al. (2024) found that higher baseline N100 predicted better outcomes with accelerated 1 Hz-rTMS over the right DLPFC. Strafella et al. (2023) found that responders to iTBS had higher baseline N100 and showed significant reductions post-treatment. Similarly, Voineskos et al. (2021) reported N100 decreases after high-frequency or bilateral rTMS, with changes correlating with clinical improvement. Sun et al. (2016) extended this to suicidality, showing baseline N100 predicted symptom reductions following magnetic seizure therapy (MST). Combining N100 with LICI improved prediction accuracy.

*P200 component:* Four of eight studies (Dhami et al., 2021, Dhami et al., 2020, Han et al., 2023, Li et al., 2024, Li et al., 2025, Li X et al., 2023, Niu et al., 2024, Voineskos et al., 2019) reported P200 alterations. Dhami et al., 2020, Dhami et al., 2021 and Niu et al. (2024) found increased P200 amplitude in the left DLPFC, while Li et al. (2024) observed reduced whole-brain P200 (GMFA) amplitude, negatively associated with depressive and anxious symptoms. This suggests that local versus whole-brain markers may reflect distinct mechanisms. Out of the four studies that examined the P200 component in the context treatments (Dhami et al., 2021, Dhami et al., 2023, Eshel et al., 2020, Han et al., 2023), none reported significant modulations or link with clinical outcomes.

*N280 and P380 components:* Of two studies to date (Li et al., 2025, Niu et al., 2024), only Li et al. (2025) found reduced N280 in MDD, with lower amplitudes correlating with greater symptom severity. However, the functional significance of N280, particularly in the prefrontal cortex, remains unclear. Niu et al. (2024) is the only study that examined the P380 and found no alterations in MDD. Neither component has been studied in relation to treatment outcomes.

*Other TEP features:* One study assessed LMFA and reported reduced amplitude (164–215 ms) in the left DLPFC (Han et al., 2023), whereas another study found no LMFP alteration (Wada et al., 2022). Two studies examined GMFA following left DLPFC stimulation, with conflicting results: Li et al. (2024) observed reduced GMFA, whereas Voineskos et al. (2019) found increased GMFA, along with altered coupling between GMFA and N45 amplitude suggesting possible E/I imbalance. No significant alteration of GMFP was found by Wada et al (2022). Prefrontal paired associative stimulation (PAS), paring median nerve stimulation with TMS to the DLPFC (Rajji et al., 2013), has provided a proxy of prefrontal neuroplasticity in MDD. Another study reported reduced early TEPs modulation (30–40 ms) following PAS in MDD (Kaneko et al., 2024). One study quantified LMFA and found a significant increase post 10-Hz rTMS, associated with symptom improvement (Han et al., 2023). Similarly, Sun et al. (2018) and Casarotto and colleagues found increased local cortical evoked activity after MST and electroconvulsive therapy (ECT) (Casarotto et al., 2013). Regarding global effects, Strafella et al. (2023) showed that higher baseline GMFA predicted better mood improvement with iTBS, whereas Voineskos et al. (2021) found no GMFA changes following unilateral or bilateral rTMS. Lastly, three studies investigated changes in LICI. Li et al. (2022) reported reduced LICI after iTBS—but not after 10-Hz rTMS or sham—while Sun et al. (2016, 2018) found reduced LICI post-MST and that baseline LICI predicted remission of suicidal ideation. These findings suggest overlapping effects of MST and iTBS and underscore the predictive potential of LICI. Finally, studies by Canali and colleagues (Canali et al., 2017, Canali et al., 2014) on chronotherapy showed local TEP slope changes during treatment and higher baseline cortical activity in responders (Tremblay et al., 2019).

### 9.3 Power spectrum and ERSP findings

TMS-evoked oscillatory dynamics provide insights into network-level dysfunction in MDD. Five studies (Dhami et al., 2020, Hill et al., 2021, Noda et al., 2024, Noda et al., 2018, Wada et al., 2024) reported frequency-specific abnormalities. In the alpha and theta bands, Noda et al. (2024) reported reduced phase synchronization using the weighted phase lag index (wPLI) in bilateral DLPFC, suggesting disrupted network coherence. Wada et al. (2022) found reduced whole-brain and left DLPFC theta power. In contrast, Hill et al. (2021) found increased theta power in the left DLPFC, highlighting inconsistency in findings. In the beta range, no alterations were found, though Noda et al. (2024) observed increased fronto-central beta power in resting EEG. For gamma, Noda et al. (2024) observed increased right DLPFC phase synchronization, while Niu et al. (2024) found reduced trial-by-trial variability (TTV) in the 32–64 Hz range, that negatively correlated with depression severity. Niu et al. (2024) also reported reduced TTV in delta, whereas Hill et al. (2021) reported increased delta power, pointing to potential divergence between amplitude and variability metrics. Finally, Canali et al. (2015) showed lower frontal dominant frequencies in MDD (∼19 Hz) relative to healthy controls (∼27 Hz).

Assessing prefrontal plasticity with PAS, Noda et al. (2018) found attenuated PAS-induced potentiation in MDD (smaller reductions in gamma, theta, delta power, and weaker theta-gamma coupling), while Kaneko et al. (2024) reported reduced gamma-band modulation post-PAS.

One multicenter study assessed the predictive value of rTMS-related oscillations on treatment outcomes. Zangen et al. (2023) examined spectral power during inter-train intervals of the first session of deep rTMS (n = 169). In the medial PFC group (H7 coil), greater low-gamma power, reduced alpha power, and a higher low-gamma/alpha ratio predicted better outcomes following six weeks of treatment. Alpha-band evoked asymmetric activity across the PFC also predicted clinical improvement in both medial (H7 coil) and lateral (H1 coil) rTMS groups, supporting its proposed role in affective regulation, and potential as a marker of neuromodulation efficacy. A previous case report showed iTBS treatment restored prefrontal asymmetry in TMS-evoked gamma and alpha power (Pellicciari et al., 2017), but replication in larger samples is needed.

Two studies explored the effects of ECT and MST on TMS-related oscillations. Hill et al. (2021) reported reduced delta and theta power after MST and broader reductions (delta, theta, alpha) after ECT, with alpha decreases linked to symptom improvement. Miyauchi et al. (2019) reported that decreased alpha-band phase locking value (PLV) following ECT correlated with greater clinical improvement. Changes in phase locking factor (PLF) latency between visual and motor regions correlated with symptom severity, suggesting ECT may restore inter-regional temporal coordination.

### 9.4 Connectivity and source density findings

Source-level TMS-EEG has revealed both local and network-level dysfunction in MDD, particularly within prefrontal-limbic circuits. Wada et al. (2024) observed decreased current density in the left DLPFC and disrupted signal propagation (i.e., dynamic statistical parametric maps, dSPM) during intracortical facilitation (ICF), correlating with reduced astrocyte-specific gene expression —suggesting glial contributions to excitability deficits in MDD. However, Wada et al. (2022) previously found no prefrontal alteration using dSPM during single-pulse TMS. Han et al. (2023) reported reduced source current density (SCD) across the DLPFC, orbitofrontal cortex (OFC), and hippocampus, alongside decreased low-frequency significant oscillation envelope (SOE; in delta, theta, alpha), indicating impaired slow-wave synchronization. Hadas et al. (2019) found elevated SCD in the subgenual cingulate cortex (SGC) during P30, N100, and P200, differencing MDD from controls with 77 % accuracy.

Functional connectivity findings similarly point to fronto-limbic disruptions. Han et al. (2023) found decreased signal propagation (significant current scattering, SCS) between the DLPFC and OFC, while Hadas et al. (2019) reported increased DLPFC-SGC SCS. Pantazatos et al. (2023) showed that DLPFC–SGC connectivity is normally modulated by alpha phase in the left DLPFC; this modulation was absent in MDD, suggesting impaired top-down inhibitory control and persistent SGC hyperactivity.

In the alpha and theta bands, Dhami et al. (2020) found reduced mean coherence connectivity between the left DLPFC and several cortical regions (e.g., left rostral ACC, left angular gyrus, right precuneus), achieving 83 % classification accuracy of youth with TRD versus controls. Similarly, Miyauchi et al. (2019) found lower alpha PLV between visuo-motor areas was linked to higher depression severity, indicating impaired inter-regional synchrony may track clinical symptoms. Finally, Wada et al. (2022) found reduced DLPFC-salience network signal propagation in the theta/alpha bands, positively correlated with oligodendrocyte gene expression.

Although SCD and connectivity metrics are increasingly used to characterize depression-related neurophysiology, only four studies have applied them to assess treatment effects. Han et al. (2023) reported normalized SCD in the DLPFC, OFC and hippocampus post HF-rTMS. Hadas et al. (2019, 2020) found reduced DLPFC-SGC connectivity (SCS) after both HF-rTMS and MST. MST also reduced hippocampal SCD, correlating with memory changes, whereas rTMS selectively reduced SGC SCD in the P200 time window. Together, this suggests both treatments target the same circuit but differ in their downstream effects.

Finally, Hao et al. (2024) examined transcranial photobiomodulation (tPBM) of the frontal pole and observed increased functional connectivity between fronto-central and left posterior temporal regions (using Causality Analysis of Directed Transfer Function), indicating that tPBM may enhance long-range cortical communication in networks implicated in emotion and language processing.

### 9.5 Summary and prospectives

Recent TMS-EEG studies (cf. Table 9.1) consistently report alterations in mid- to late-latency TEP components (P60, N100, and P200) in MDD/TRD, with earlier components (P30, N45) showing less reliable effects. These mid/late components, linked to glutamatergic and/or GABAergic neurotransmission, correlate with symptom severity and may serve as biomarkers of E/I dysfunction in the DLPFC (Farzan, 2024). Frequency-domain analyses also reveal disruptions in spectral power and phase synchrony across alpha, theta, gamma, and delta bands. Altered prefrontal plasticity and disrupted connectivity with the SGC, hippocampus and OFC suggest broader network dysfunction in MDD.

Emerging metrics such as SCS, SCD, dSPM and gene-informed connectivity provide promising avenues to link TMS-EEG features with circuit-level dysfunction. Recent work leveraging multimodal and machine learning approaches have demonstrated high classification accuracy for MDD, e.g., AUC > 90 % (Noda et al., 2024), suggesting strong potential for clinical translation.

In terms of treatment outcomes, TMS-EEG effectively tracks neurophysiological changes across a wide range of treatment modalities in depression, including TBS, rTMS, MST and ECT. Improvements in E/I balance, normalization of oscillatory rhythms and restoration of network coherence have been linked to treatment response. Components like the N100 and N45, along with alpha and low-gamma activity evoked by rTMS trains, show promise as markers of therapeutic outcome. Less commonly used metrics (e.g., source density, connectivity, synchrony) offer further insights into both local and network-level effects of treatments in MDD.

Together, these findings support a mechanistic model where effective treatment recalibrates disrupted prefrontal-limbic pathways in depression. However, heterogeneity in stimulation protocols, preprocessing, analysis methods and sample characteristics currently limits generalizability. With greater methodological harmonization and replication/validation in larger, stratified samples, TMS-EEG holds promise for developing relevant neurophysiology biomarkers for diagnosis, symptom monitoring and treatment selection in depression.

## 10 Schizophrenia

### 10.1 Introduction

Schizophrenia (SCZ) is a major psychiatric disorder characterized by positive symptoms (e.g., delusions, hallucinations), negative symptoms (e.g., reduced emotional range, social withdrawal), and cognitive impairments, with a lifetime prevalence of 1 % (Moreno-Kustner et al., 2018). Although many brain regions (e.g., hippocampus, striatum) and molecular mechanisms (e.g., dopaminergic pathway) have been implicated in the neurobiology of the disorder, increasing electrophysiological (EEG) evidence points toward alterations in frontal-prefrontal cortical areas and in disrupted glutamatergic and GABA-ergic neurotransmission (Gonzalez-Burgos et al., 2023, Mathalon and Sohal, 2015, Uhlhaas and Singer, 2015). Specifically, aberrant fast beta and gamma EEG oscillations have been reported in numerous studies of patients with schizophrenia (Hirano and Uhlhaas, 2021). TMS-EEG recordings can be collected without requiring any conscious effort or engagement from the participant, which allows to mitigate several of the confounds, including lack of motivation and fluctuation in the level of attention, commonly observed in patients with SCZ during wakefulness EEG recordings. In this section, we will review TMS-EEG studies in SCZ that have been published since 2019, the year the previous review was published (Tremblay et al., 2019).

### 10.2 TMS-EEG findings

Recent work has emphasized the possibility of using TMS-EEG as a powerful tool to investigate abnormalities in brain activity and connectivity in SCZ by directly assessing cortical neuronal responses to controlled perturbations, thus offering novel insights into the pathophysiology of the disorder. For example, one study by Molina et al. examined the PCI in individuals with SCZ and found that PCI values were significantly lower in both chronic and first-episode patients compared to healthy control (HC) subjects (Molina et al., 2025). This reduction indicates that the spatiotemporal patterns of the cortical responses evoked by TMS in SCZ are less complex, suggesting an impaired capacity to dynamically coordinate neural activity across distributed networks in these patients. Furthermore, several recent TMS-EEG studies have specifically targeted frontal cortical regions, from motor to prefrontal areas, to examine the induced oscillatory activity, including the natural frequency in first episode psychosis (FEP) and early-course (EC)-SCZ patients vs. HC. Two initial studies found that FEP patients had reduced TMS-evoked EEG activity in the beta frequency band in the motor region that correlated with worse positive psychotic symptoms at baseline and predicted positive symptoms severity at six-month follow-up assessments (Donati et al., 2021, Ferrarelli et al., 2019). Based on these findings the authors concluded that reduced TMS-evoked fast oscillatory activity in the motor cortex is an early neurophysiological abnormality that: 1) is present at illness onset; 2) may represent a state marker of psychosis; and 3) could facilitate the development of new tools of outcome prediction in psychotic patients. Another TMS-EEG work found that the natural frequency of the premotor cortex was significantly reduced in EC-SCZ compared to HC subjects (Donati et al., 2023), while a recently published study established that, compared with HC, EC-SCZ patients had reduced prefrontal natural frequency and higher prefrontal slower, beta-range relative spectral power (Donati et al., 2025). Furthermore, in EC-SCZ patients, the DLPFC natural frequency was inversely associated with negative symptoms, whereas the beta band relative spectral power negatively correlated with AX-Continuous Performance Task performance, a measure of sustained and selective attention, thus suggesting that prefrontal oscillatory slowing is an early pathophysiological biomarker of SCZ that is associated with its symptom severity and cognitive impairments. Another TMS-EEG study has recently shown that, compared to HC, individuals with SCZ had reduced theta oscillations and trend level decreases in task-related theta and cortical reactivity along with trend level associations between task-related oscillations and impaired cognition, thus providing further experimental support for neurophysiological markers of cognitive deficits in SCZ (Hoy et al., 2021).

TMS-EEG can also be used to assess the effects of repetitive TMS protocols, including TBS, in patients with SCZ, including treatment resistant individuals. For example, one pilot study demonstrated that iTBS delivered to the left prefrontal cortex led to significant improvements in visual-spatial working memory (Wang L et al., 2022). These improvements were associated with changes in local brain activity (e.g., fractional amplitude of low frequency fluctuations), indicating that targeted neuromodulation can positively affect both neurophysiological markers and clinical symptoms. Moreover, systematic reviews of TMS-EEG studies in SCZ have highlighted consistent abnormalities in cortical excitability, connectivity, and plasticity, further supporting the role of TMS-based interventions in these patients (di Hou et al., 2021, Ferrarelli and Phillips, 2021).

### 10.3 Summary and prospectives

TMS–EEG has been used in several studies to better understand the neurophysiological characteristics of SCZ in recent years. They provided converging evidence for altered cortical excitability, in particular for abnormal generation and modulation of frontal beta and gamma oscillations and a link of these abnormalities to the clinical severity of SCZ. In a next step it would be of much interest to test the utility of induced frontal beta or gamma oscillations as a monitoring and/or predictive biomarker of treatment effects in large-scale pharmacological or brain stimulation randomized controlled treatment trials.

## 11 Autism spectrum disorder

### 11.1 Introduction

Autism spectrum disorder (ASD; hereafter, autism) is a persistent neurodevelopmental condition estimated to affect 78 million people worldwide, or approximately 1 % of the global population (Lord et al., 2022, Zeidan et al., 2022). Autism is characterized by challenges in social interaction and communication, as well as the presence of restricted interests and repetitive behaviors (RRBI; APA, 2022). While autism is typically first diagnosed during early childhood, many autistic adolescents and adults continue to face ongoing social, cognitive, and communicative difficulties, which place significant barriers to employment, community engagement, and interpersonal relationships (Howlin et al., 2015). The neurobiology of autism is complex and remains incompletely understood, although it is commonly linked to an altered neurodevelopmental trajectory, particularly atypical patterns of cell proliferation and synaptic pruning (Dawson et al., 2023, Rafiee et al., 2022). Alterations in structural and functional connectivity as well as disruptions in excitation-inhibition balance have also been widely observed (Holiga et al., 2019, Rubenstein and Merzenich, 2003).

### 11.2 TMS-EEG findings

To date, five studies have utilized TMS-EEG to examine neural activity and connectivity in autism (Table 11.1). Four studies failed to show differential effects in TMS-induced electrophysiological responses. An initial 2016 study by (Jarczok et al., 2016) used TMS-EEG to measure interhemispheric signal propagation (ISP) between the left and right primary motor cortices between autistic individuals and neurotypical controls. This approach assesses connectivity between these regions by measuring transmission of cortical evoked activity as it travels through the corpus collosum. No differences in ISP were observed between groups. Similarly, aimed at informing the E/I balance hypothesis, Kirkovski and colleagues (2016) reported no difference between autistic and typically developing adults in high-frequency (gamma and beta) oscillatory power or phase synchrony, as measured by the weighted phase lag index (wPLI), in response to single-pulse TMS applied to the right dorsolateral prefrontal cortex (DLPFC), primary motor cortex (M1), or temporoparietal junction (TPJ). In a related study with an overlapping sample, the same group (Kirkovski et al., 2022) also reported no difference in the N100 TEP following single-pulse TMS, nor in the long-interval intracortical inhibition (LICI_EEG_) response following paired-pulse TMS at these sites. This outcome is further corroborated by a lack of difference in electromyographical (EMG) −related LICI outcomes between the autistic and typically developing groups (Kirkovski et al., 2022). Most recently, Mimura and colleagues also investigated LICI_EEG_ in the DLPFC using both sensor- and source-based analyses (Mimura et al., 2025). While the expected suppression of the neural response was observed following the LICI protocol, no differences were found between the autism and neurotypical control groups at either the sensor or source level. Source based time–frequency analyses also failed to show any differences between groups in terms of either event-related spectral perturbation (ERSP) or inter-trial phase clustering (ITPC) across the theta and alpha bands (Mimura et al., 2025).

**Table 11.1:** Synopsis of TMS-EEG studies in autism.

**Authors**	**Sample**	**Age (yrs)**	**Stimulation** **Site**	**EEG** **Recordings**	**TMS** **Parameters**	**Measurements/** **Intervention**	**Key Findings**
(Jarczok et al., 2016)	22 male autism, 22 male TDcontrols	autism: 10–21, TD: 9–19	Left M1	64 channels SR: 5,000 Hz	SPF-8 coil	ISP (left to right M1)	No difference in ISPbetween autism and TD. Increase in ISP with age (in both groups)
(Kirkovski et al., 2016)	22 autism (10 male), 20 TD (11 male)	autism: 21–55, TD: 19–56	Right DLPFC, right M1,right TPJ	20 channels SR:10,000 Hz	SP (75 pulses at each site;monophasic) , 70 mm F-8 coil	TMS-inducedoscillation power, wPLI	No differencesbetween autism and TD for power or wPLI
(Kirkovski et al., 2022)	23 autism (11 male), 22 TD (11 male)	Mean (SD);autism: 30.3 (9.2) TD: 29.8 (9.8)	Right DLPFC, right M1,right TPJ	20 channels SR:10,000 Hz	SP and PP 70 mm F-8 coil 75 SP and PPmonophasic pulses at each site	SP: amplitude/ latency of N100 TEP. PP: LICI_EEG_	No between-group differences in N100 amplitude/latency or LICI_EEG_
(Mimura et al., 2025)	32 autism (16 male), 34 TD controls (18 male)	Mean (SD) autism: 28.8 (8.32), TD: 29.2 (8.10)	Left DLPFC	64 channels SR: 3,000 Hz	80 SP and 80 PP	LICI_EEG_; TFA (ERSP, ITPC)	No between-group differences in LICI_EEG_, ERSP, or ITPC
(Yang et al., 2024)	12 children with autism (9 male)	Mean (SD)7.2 (3.1)	Left parietallobe (P3electrode)	32 channels SR: 1,024 Hz	SPF-8 coil	Network connectivity (directed) pre-and post-rTMS (15 rTMS sessions over left parietal cortex)	Strengthened long- range and reduced short-range connectivityfollowing rTMS

Abbreviations: DLPFC, dorsolateral prefrontal cortex; ERSP, event-related spectral perturbation; F-8, figure-of-eight; ISP, interhemispheric signal propagation; ITPC, inter-trial phase clustering; LICI, long-interval intracortical inhibition; M1, primary motor cortex; PP, paired-pulse; rTMS, repetitive transcranial magnetic stimulation; SP, single-pulse; SR, sampling rate; TD, typically developing; TEP, TMS-evoked potential; TFA, time–frequency analysis; TPJ, temporoparietal junction; wPLI, weighted phase-lag index.

With respect to the clinical features of autism, in contrast to the findings of (Jarczok et al., 2016), who report no relationship between ISP and autism severity scores measured using the Social Responsiveness Scale, Kirkovski et al. (2016) provide correlational evidence to suggest that elevated autistic features may be associated with the response to TMS, by way of reduced functional connectivity, as measured using TMS-evoked phase synchrony.

Sample demographics are also a critical consideration with respect to the reviewed studies. Specifically, age (O’Hearn and Lynn, 2023) and biological sex (Floris et al., 2023, Kirkovski et al., 2013, Napolitano et al., 2022) are factors known to contribute to the heterogeneity observed in autism, and indeed, both have been implicated here. For example, (Jarczok et al., 2016) report greater ISP values in older participants, likely reflecting maturational changes in signal propagation across childhood to early adulthood. With respect to biological sex, while not reaching statistical significance, trends indicative of reduced beta-band phase synchrony in autism following TMS to the right M1 and TPJ were reported by Kirkovski et al. (2016), an effect that appeared to be driven by female sex.

Over the past decade, there has been increased interest in the therapeutic application of plasticity-inducing TMS protocols to target the core symptomatology of autism (Chen et al., 2024, Cole et al., 2019, Enticott et al., 2021, Enticott et al., 2014, Oberman et al., 2016, Smith et al., 2023). Presently, only one study has utilized TMS-EEG to examine the impact of such protocols in autism. In a recent study, Yang et al. (2024) examined effective connectivity alterations following high-frequency repetitive TMS (rTMS) in autistic children (age range: 4 – 13 years). The authors collected TMS-EEG recordings before and after a three-week course (total of 15 sessions) of rTMS administered over the left parietal cortex. The rTMS procedure consisted of a series of five five-second 15 Hz TMS trains, with each train spaced 10 min apart. TMS-EEG recordings were obtained before and after rTMS over the left parietal cortex. A time-varying directed network analysis approach was used to measure effective connectivity. Following the rTMS intervention, a strengthening in long-range connections between brain regions was observed, while short-range connections in posterior regions tended to be diminished. Given long-range underconnectivity is likely to be an underlying feature in ASD (O'Reilly et al., 2017), these preliminary findings provide some initial evidence to suggest that rTMS might be capable of modifying long-range connections in ASD. It should be noted, however, that this study did not include a sham condition for comparison, though a published protocol will do so in a single-session plasticity study using paired associative stimulation-EEG (Kariminezhad et al., 2024).

### 11.3 Summary and prospectives

While limited in number, these studies provide preliminary evidence supporting the utility of TMS-EEG for investigating brain function in autism, particularly regarding assessing the clinical application of plasticity-inducing rTMS protocols. Future research could expand on these findings by incorporating larger sample sizes, a broader age range, and using TMS-EEG to explore the effects of a wider range of plasticity-inducing protocols in autism.

## 12 Attention-Deficit hyperactivity disorder (ADHD)

### 12.1 Introduction

The pathophysiology of ADHD is poorly understood in terms of the electrophysiological basis for the behavioral impairments, and no widely accepted biomarker or objective diagnostic test currently exists. As a result, diagnosis typically relies on behavioral rating scales filled by parents and teachers, alongside the clinical judgment of a physician based on an interview and sometimes supported by computerized cognitive tasks (Jannati et al., 2022). Although still in its early stages, TMS-EEG has shown some promise as an objective method for assessing neurophysiological function in individuals with ADHD. By providing a quantitative framework, TMS-EEG may enhance diagnostic precision and support treatment monitoring, thereby reducing clinical variability and advancing personalized care (Cao et al., 2021, Hadas et al., 2021, Tremblay et al., 2019).

### 12.2 TEP findings

*N100 component.* Bruckmann et al. used a circular coil to administer TMS over the vertex, oriented tangentially to the skull over the left hemisphere (Bruckmann et al., 2012). The TMS-evoked N100, defined as the most prominent negative peak at electrode C3, ipsilateral to the stimulation site, was compared between children with or without ADHD. The authors reported a significantly reduced N100 amplitude at rest, a trend toward shorter N100 latency, and a smaller reduction in N100 amplitude during movement execution in children with ADHD. Given that a reduction in N100 was found across all age groups in children with ADHD, the authors concluded that it reflects a qualitative alteration in cortical inhibition, rather than a simple developmental delay. D’Agati et al. employed a figure-of-eight coil to deliver TMS over the left M1, measuring the TMS-evoked N100 at the ipsilateral P3 electrode (D'Agati et al., 2014). While no group differences were observed in N100 amplitude at rest, children with ADHD showed reduced modulation of the N100 during both go and no-go trials. The authors suggested that these results partially support the role of TMS-evoked N100 as a motor cortical marker of abnormal inhibitory processes in children with ADHD. Avnit et al. utilized a figure-of-eight coil to apply TMS over the right prefrontal cortex (PFC) and found a reduced area under the rectified curve alongside diminished right-to-left inter-hemispheric signal propagation of the TEP response within 50–150 ms following the pulse in adults with ADHD (Avnit et al., 2023). This reduction was found to correlate with right-frontal asymmetry of the stop-signal N200 ERP component (a marker of response inhibition). The authors concluded that abnormal frontal asymmetry is linked to a core cognitive deficit in ADHD and suggested that this asymmetry may be partially driven by reduced right-frontal cortical excitability and connectivity, as indicated by the abnormal TEP responses.

*P30 component.* Hadas et al. used a figure-of-eight coil to apply TMS over the right PFC of young adults with or without ADHD, and reported a significantly reduced LMFP of the P30 component over right frontal electrodes in the ADHD group, which correlated with symptom severity (Hadas et al., 2021). The authors proposed that the attenuated P30 response may reflect reduced cortical excitability of the right PFC in individuals with ADHD, albeit LMFP may also be influenced by the spatial characteristic of the cortical response, leading to alternative interpretations, e.g., the distribution of the signal rather than the overall magnitude of the response to the pulse.

### 12.3 Predicting and monitoring treatment response

Helfrich et al. administered either 1 Hz rTMS using a figure-8 coil or sham stimulation to the left M1 of children with ADHD for 15 min (900 pulses) while recording EEG (Helfrich et al., 2012). They observed a progressive decrease in the TMS-evoked N100 amplitude during active 1 Hz-rTMS, which reached a plateau after approximately 500 pulses at the group level. Post-rTMS assessments using supra-threshold single pulses confirmed a sustained reduction in N100 amplitude compared to pre-rTMS baseline, whereas sham stimulation had no effect. The authors suggested that changes in the TMS-evoked N100 amplitude during rTMS sessions may serve as a real-time physiological marker of stimulation effects on cortical excitability and perhaps on clinical outcomes, although this study did not determine whether these changes in N100 are predictive of clinical improvement. Alyagon et al. examined the relationship between treatment and TMS-evoked EEG activity in adults with ADHD (Alyagon et al., 2020). The intervention involved a three-week course of daily high-frequency (18 Hz) deep rTMS targeting the right PFC using the H6-coil, with TEPs recorded before and after the first and last treatment sessions using a figure-of-eight coil. Notably, the authors found that alpha and low-gamma EEG activity recorded during the intertrain interval of the first rTMS session strongly predicted treatment outcomes, accounting for close to 90 % of the variance in clinical response. They proposed that low-gamma activity may reflect an immediate cortical response to rTMS, whereas alpha activity represents a more stable, trait-like neural characteristic of the cortical inhibition level. Additionally, treatment-related modulation of the N75 component was observed, with significant changes detected in both frontal and parietal-occipital electrode clusters. The authors concluded that these persistent neurophysiological changes likely reflect neuroplastic adaptations induced by multiple rTMS sessions, indicating an effect on the E/I balance within the stimulated neural networks. However, the significant modulation of this TEP component did not predict clinical outcomes.

### 12.4 Summary and prospectives

The available TMS-EEG evidence points to abnormal inhibitory processes and their deficient modulation by voluntary action and response inhibition in frontal cortex of ADHD. It would be of much interest to test these TMS-EEG markers, in particular the N100 TEP in future clinical trials for their utility as monitoring or predictive biomarkers of the treatment response.

## 13 Substance use disorders

### 13.1 Introduction

Substance use disorders (SUDs) are chronic and relapsing conditions characterized by compulsive alcohol, tobacco, or drug consumption despite negative consequences (Volkow and Blanco, 2023). They are linked with substantial cognitive, emotional, and neurobiological impairment, while their pathophysiology is poorly known (Volkow and Blanco, 2023). Among the most affected systems are the GABAergic and glutamatergic neurotransmitter networks (Volkow and Blanco, 2023), and addiction is increasingly understood as a disorder of neural plasticity (O'Brien, 2009).

A key mechanism in the development of SUDs is the mesocorticolimbic circuit, which mediates the neuroplastic transition from voluntary drug use to compulsive substance-seeking. This shift is driven by drug-induced alterations in reward sensitivity, executive control, and emotional regulation (Di Chiara and Imperato, 1988, Koob and Volkow, 2010, Lüscher et al., 2020, Volkow et al., 2016). These maladaptations are core to the pathophysiology of SUDs. SUDs are highly prevalent worldwide, with rates differing by country and substance type, and typically, males are impacted more than females (Vasilenko et al., 2017).

### 13.2 TMS-EEG findings

So far, TMS-EEG has been applied in heroin and methamphetamine use (Liu et al., 2024), alcohol dependence (Naim-Feil et al., 2016, Naim-Feil et al., 2022), heavy long-term alcohol use (Juntunen et al., 2023, Kaarre et al., 2018) and in healthy individuals following acute ethanol exposure (Kähkönen et al., 2001, Kähkönen and Wilenius, 2007, Kähkönen et al., 2003, Loheswaran et al., 2017) (Table 13.1).

**Table 13.1:** Synopsis of TMS-EEG studies in SUDs.

**Authors**	**Sample**	**Region(s) of interest**		**EEG recordings**		**TMS** **parameters**		**Measurements/** **Intervention**	**Key findings**
*Heroin and methamphetamine use*									
(Liu et al., 2024)	72 heroin, 69 meth-amphetamine, 35 HS	F3 (left frontal), F4 (right frontal), and P3 (left parietal) EEG electrodes		64 channels SR: not reported		Single-pulse, 10 Hz rTMS, 100 % rMT intensity, neuronavigation		10 Hz rTMS left DLPFC, ERSP pre- and post-rTMS	At baseline ERSP did not differ between groups, post-rTMS HS showed ERPS alpha decrease at P3 and ERPS beta increase at F3
*Alcohol use*									
(Naim-Feil et al., 2016)	12 ALD post-detoxification, 14 HS	Left and right DLPFC		24 channels SR: 20,000 Hz		Single-pulse, paired-pulse (LICI with ISI 100 ms), intensity corresponding to 1 mV MEP		TEP area under rectified curve	In ALD, less LICI in both DLPFCs relative to HS, no difference in single pulse area under curve
(Kaarre et al., 2018)	27 heavy drinkers since adolescence and 25 HS	Left M1		64 channels SR: 5,000 Hz		Single-pulse, 90 % of rMT intensity, biphasic, neuronavigation		TEP amplitude, GMFP, topography	Heavy alcohol use during adolescence showed GMFP increase, N45 TEP increase, altered P60 and N100 TEP topographies
(Naim-Feil et al., 2022)	11 ALD early recovery, 16 HC	Left and right DLPFC		24 channels SR: 20,000 Hz		Single-pulse, paired-pulse (LICI with ISI 100 ms), intensity corresponding to 1 mV MEP		Graph theory	No differences between ALD and HS after single-pulse, after LICI, the network topology was significantly different in ALD compared to HS, some network metrics linked with clinical ratings of severity of alcohol use
(Juntunen et al., 2023)	26 heavy drinkers since adolescence and 21 HS	Left M1		64 channels SR: 5,000 Hz		Single-pulse, 90 % of rMT intensity, biphasic, neuronavigation		N45 TEP, cortical thickness	Thinner cortex correlated with larger N45 amplitude
*Acute alcohol exposure in healthy individuals*									
(Kähkönen et al., 2001)	10 HS	Left M1	60 channels SR: 1,450 Hz		Single-pulse, sham, 100 % of rMT intensity, biphasic		Pre- and pos-ethanol exposure, GMFA		Altered GMFA over right frontal and left parietal areas
(Kähkönen et al., 2003)	9 HS	Left DLPFC	60 channels SR: 1,450 Hz		Single-pulse, sham, 100 % of rMT intensity, biphasic		Pre- and pos-ethanol exposure, GMFA		GMFA decrease at 30–270 ms post-TMS
(Kähkönen and Wilenius, 2007)	10 HS	Left M1	60 channels SR: 1,450 Hz		Single-pulse, sham, 100 % of rMT intensity, biphasic		Pre- and pos-ethanol exposure, N100 TEP		N100 decrease
(Loheswaran et al., 2017)	15 HS	Left DLPFC	64 channels SR: 20,000 Hz		Single-pulse, intensity corresponding to 1 mV MEP, monophasic		Pre- and pos-ethanol exposure, pre-post paired associative stimulation (PAS), TEP power, theta-gamma coupling		TEP power decrease after ethanol exposure, theta-gamma coupling decrease after PAS and ethanol exposure

Abbreviations: ALD, alcohol-dependent; DLPFC, dorsolateral prefrontal cortex; EEG, electroencephalography; ERSP, event-related spectral perturbation; GMFA, global mean field amplitude; GMFP, global mean field power; HS, healthy subjects; ISI, inter-stimulus interval; LICI, long-interval cortical inhibition; MEP, motor evoked potential; M1, primary motor cortex; PAS, paired associative stimulation; rMT, resting motor threshold; SR, sampling rate; TEP, TMS-evoked potential; TMS, transcranial magnetic stimulation

Liu and colleagues (Liu et al., 2024) investigated cortical excitability and plasticity in response to a single session of 10 Hz rTMS targeting the left DLPFC in individuals with heroin and methamphetamine use disorders. At baseline, cortical excitability, as assessed by TMS-induced ERSP, did not differ between SUD groups and healthy controls. In healthy controls, post-rTMS measurement showed diffuse alpha and beta power decreases and increases, depending on the evaluated site, whereas SUD groups showed no changes. These findings suggest impaired cortical plasticity in heroin and methamphetamine use disorders, supporting the theory that addiction is a disorder of neuroplasticity.

In the context of alcohol dependence, Naim-Feil et al. applied paired-pulse TMS-EEG to evaluate LICI in the left and right DLPFC in post-detoxification (Naim-Feil et al., 2016). They found that in both DLPFCs, individuals with alcohol dependence presented reduced LICI compared to healthy individuals. As the DLPFC is a key structure in the mesocorticolimbic circuitry, the authors speculated that the findings reflect reduced GABAergic activity in this circuitry, which has been shown to have a critical role in the development of addiction (Koob and Bloom, 1988).

Expanding on this work, Naim-Feil et al. further examined network-level changes in early recovery using LICI and single-pulse TMS-EEG (Naim-Feil et al., 2022). Following single-pulse stimulation, there were no differences between alcohol dependence and healthy controls in network topology assessed with measures from graph theory. However, after paired-pulse TMS-EEG for LICI evaluation, the network topology was significantly different in alcohol dependence compared to healthy controls in the left DLPFC. Importantly, some of the network metrics were linked with clinical ratings of the severity of alcohol use. This highlights the potential of TMS-EEG for the detection of clinically relevant network impairments in addiction.

The effects of alcohol have also been studied in individuals with heavy alcohol use since adolescence, but who did not yet fulfill alcohol use disorder criteria (Kaarre et al., 2018). Heavy-drinking adolescents were matched with adolescents with little or no alcohol use when they were 13–18 years old The participants underwent a TMS-EEG study targeting M1 when they were 23–29 years old. Heavy alcohol use during adolescence was found to be linked to increased GMFP, enhanced N45 TEP amplitude, as well as altered P60 and N100 TEP topographies. Thus, while their alcohol use did not reach diagnostic levels, consumption during adolescence led to widespread changes in cortical excitability and functional connectivity. Juntunen et al. continued to assess the same study sample by evaluating how the N45 TEP results in heavy drinking during adolescence were associated with cortical thickness (Juntunen et al., 2023). They found that in several brain regions, such as the left superior frontal gyrus, the left supramarginal gyrus, and both superior parietal lobes, thinner cortex correlated with larger N45 amplitude. These brain regions develop late in adolescence (Gogtay et al., 2004) which underscores the vulnerability of the adolescent brain to alcohol.

Although these studies evaluated the long-term impact of alcohol use, similar widespread effects have been observed after acute alcohol exposure in healthy subjects. For instance, Kähkönen et al. reported abnormal TEPs in the right prefrontal and left parietal regions (Kähkönen et al., 2001). In a subsequent study, they also found that alcohol significantly reduced cortical excitability, as indicated by a decreased GMFA (Kähkönen et al., 2003). These effects were most pronounced at anterior electrodes, reinforcing the role of prefrontal inhibition. Considering the N100 TEP, Kähkönen and Wilenius showed that alcohol nearly abolished the N100 response across ipsilateral, contralateral, and frontal sites (Kähkönen and Wilenius, 2007). Loheswaran et al. extended this work by demonstrating that alcohol impairs LTP-like plasticity in the DLPFC induced by paired associative stimulation (Loheswaran et al., 2017). Alcohol consumption significantly reduced both mean and peak potentiation of cortical evoked activity, and disrupted theta-gamma coupling, which is thought to be critical for working memory and cognitive function (Rajji et al., 2013).

### 13.3 Summary and prospectives

Current evidence from TMS-EEG studies (summarized in Table 13.1) shows alterations in cortical excitability, inhibition, and neuroplasticity across various stages and types of SUDs. Many of these findings converge on the mesocorticolimbic system, especially the DLPFC, which has been identified as a key hub affected by chronic substance use. Findings of both acute and longitudinal studies also support the sensitivity of TMS-EEG to short-term as well as long-term changes in cortical function for substances. Future studies should expand the SUDs studied with TMS-EEG and apply TMS-EEG to monitor how the responses change over time, in response to interventions and how they could be used to predict outcomes.

## 14 Disorders of consciousness

### 14.1 Introduction

At its core, consciousness is the ability to experience what it feels like to be something (Nagel, 1974). From a first-person perspective, this intrinsic sense of existence is undeniable, directly given without requiring external validation. However, because experience is inherently private, determining whether others are conscious is always an inference. The clinical gold standard for inferring consciousness is behavioral. In this context consciousness is operationalized as the capacity to maintain integrated awareness of both environmental stimuli and internal physiological states, reflected in coherent behavioral responses to multimodal inputs. The clinical spectrum of consciousness disorders emerges when this adaptive responsiveness becomes quantitatively diminished or qualitatively altered, according to standard behavioral scales, such as the Coma Recovery Scale – Revised (CRS-R). These pathological states primarily result from severe acquired brain injuries (e.g., traumatic, hypoxic-ischemic) or progressive neurological conditions, characterized by impairments in fundamental brainstem arousal mechanisms and/or by global impairment of thalamocortical functions. The main types of disorders of consciousness (DOC) include coma, vegetative state/unresponsive wakefulness syndrome (VS/UWS), and minimally conscious state (MCS) (Giacino et al., 2018, Kondziella et al., 2020). Among these, coma is the most severe form of consciousness disorder, characterized by the complete abolition of sleep-wake cycles and conscious awareness. In VS/UWS patients, despite the presence of eye opening, sleep-wake cycles and primitive responses to external stimuli, such as pain, purposeful behavior is absent (Laureys et al., 2010). MCS patients, on the other hand, can make limited, purposeful responses to external stimuli, but they still cannot engage in functional communication (Giacino et al., 2002). MCS can be further categorized into MCS- and MCS+, with the distinction based on whether the patient retains behaviors indicative of language comprehension and expression. The presence of even one of the following behaviors—such as following commands, producing understandable speech, or engaging in intentional communication—allows for a diagnosis of MCS+ (Bruno et al., 2011).

A fundamental challenge to this behavioral stratification is that patients recovering from severe brain injuries or neurological disorders may retain some level of consciousness yet lack essential abilities like motor function, executive control, language, or the capacity to perceive and process external information. The extent of these impairments varies widely, their combinations are unpredictable, and advances in life support continue to expand the spectrum of such conditions (Lissak and Young, 2024).

In recent years, scientists have utilized neuroimaging and neurophysiological techniques to detect the presence of consciousness beyond overt motor responses. In a pivotal study by Owen et al., fMRI analysis of a VS/UWS patient revealed preserved neural responses to linguistic stimuli, with activation patterns mirroring those of healthy controls (Owen et al., 2006). Notably, the patient exhibited task-specific cortical activation during mental imagery tasks following verbal commands. These findings provide empirical evidence for the existence of covert consciousness in certain VS/UWS cases (Curley et al., 2018, Kondziella et al., 2016). In a clinically misdiagnosed VS/UWS patient exhibiting fMRI-confirmed covert consciousness, diffusion tensor imaging (DTI) analysis demonstrated disrupted thalamocortical connectivity to motor regions, manifesting as preserved motor imagery capacity alongside complete skeletal muscle paralysis (Schiff, 2015). To describe this condition, researchers proposed the definition of cognitive-motor dissociation (CMD) (Bodien et al., 2024). This refers to a state where bedside behavioral assessments are consistent with a VS/UWS or MCS diagnosis, but laboratory tests reveal preservation of higher-order cognitive abilities. A neuroimaging investigation found that the VS/UWS patients who retained bilateral superior temporal gyrus activation to music stimuli predicted the potential for consciousness recovery (Okumura et al., 2014). Edlow et al. defined this retained stimulus-evoked cortical activation as covert cortical processing (CCP) (Edlow et al., 2023). The evolving conceptual frameworks of covert consciousness, CMD, and CCP reflect an ongoing effort to detect consciousness by analyzing brain responses to verbal commands and sensory stimuli in the absence of overt motor responses (Young et al., 2025).

### 14.2 TMS-EEG findings

Since patients with severe brain injuries may experience significant disconnection on both the sensory (input) and motor (output) levels, parallel efforts have focused on detecting consciousness in the brain independently of sensory processing, executive function, and motor behavior. At this level, signs of consciousness can only be inferred by examining their brain's internal physiological properties. In practice, this requires probing directly cortico-thalamic networks to assess their internal capacity for communication, while bypassing sensory and motor blockages and without requiring the subject’s engagement in a task. Direct cortical perturbations and recordings with TMS-EEG have thus emerged as a neurophysiological technique to probe the recovery of such internal brain interactions. In the landmark investigations (Ragazzoni et al., 2013, Rosanova et al., 2012), TMS-EEG was applied to assess cortical effective connectivity in DOC patients, demonstrating distinct effective connectivity patterns between VS/UWS and MCS patients. Specifically, MCS showed TEPs characterized by widespread and complex spatiotemporal patterns, whereas only simple local responses were observed in VS/UWS patients. To explicitly assess the spatiotemporal complexity of these interactions and based on theoretical premises (Massimini et al., 2009, Sarasso et al., 2021, Tononi and Edelman, 1998), PCI was subsequently developed.

**Fig. 14.1:**
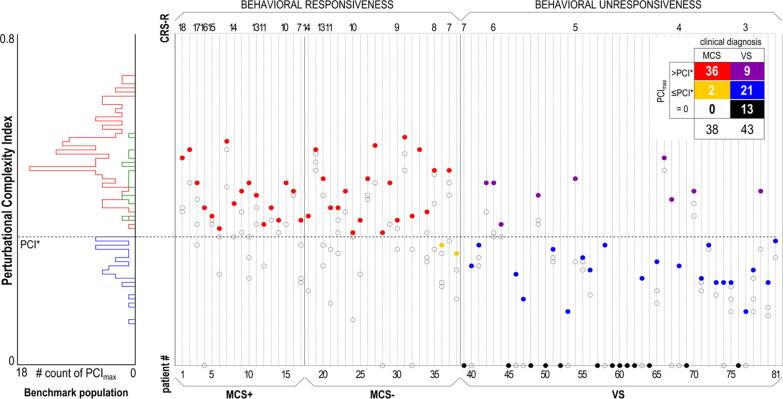
The histogram (left) summarizes the distribution of maximum Perturbational Complexity Index values (PCImax) in the benchmark population, specifically obtained in the absence of subjective report (blue) and in the presence of subjective report (delayed, green; immediate, red) conditions. The dashed horizontal line highlights the optimal cutoff (PCI*) computed from receiver operating characteristic curve analysis on the benchmark population. The scatter plot (right) shows all PCI values obtained in minimally conscious state (MCS+/MCS-) and vegetative state (VS) patients. The PCI values computed in each patient (2–4 values) are aligned along vertical columns. Within each diagnostic group, patients are sorted by the Coma Recovery Scale-Revised (CRS-R) total score in decreasing order. For each patient, PCImax is represented by a color-filled circle, whereas lower PCI values are represented by empty circles. The contingency table (right upper corner) is obtained by slicing through the PCImax values with PCI* and shows that 36 MCS patients resulted in PCImax > PCI* (red), whereas in 2 MCS- patients PCImax was lower than PCI* (yellow). In addition, VS patients could be divided into 3 subgroups according to PCImax: 9 patients with PCImax > PCI* (purple), 21 patients with PCImax ≤ PCI* (blue), and 13 patients with PCImax = 0 (black) (reproduced from (Casarotto et al., 2016)). (For interpretation of the references to colour in this figure legend, the reader is referred to the web version of this article.)

PCI gauges the compressibility (Lempel-Ziv) of the deterministic cortical pattern of activation evoked by TMS. The method relies on high-density EEG recordings and MRI guided stimulation of pre-defined brain regions. Based on a large body of evidence in healthy volunteers recorded during awake states and different stages of sleep, patients undergoing general anesthesia, and patients with chronic DOC the performance, characteristics of PCI have been carefully developed, yielding very high sensitivity and specificity measures for consciousness (Fig. 14.1) (Casali et al., 2013, Casarotto et al., 2016).

PCI measurements show relationships with the residual brain metabolism after injury (Bodart et al., 2017) and allow a reliable stratification of DOC patients (Casali et al., 2013, Casarotto et al., 2016). A cut-off of 0.31 (PCI*), identified in benchmarks conditions (Fig. 14.1), results in a detection of MCS patient with high (94 %) sensitivity (Casarotto et al., 2016). Notably, the sensitivity of TMS-EEG was retained also in MCS patients showing moderately and severely abnormal patterns in the spontaneous EEG (Casarotto et al., 2024). Most importantly, patients classified as VS/UWS based on behavioral assessments exhibit significant heterogeneity in PCI. The functional integrity of cortical networks in behaviorally unresponsive patients can vary across individuals and brain regions (Li C et al., 2023), underscoring the need for more refined stratification. In this respect PCI measures allow a three-tier classification of VS/UWS patients based on their cortical responses to TMS: (1) Non-responsive (PCImax = 0), characterized by a complete absence of response to TMS; (2) Low complexity (PCImax ≤ PCI*), showing only simple, localized responses; and (3) High complexity (PCImax > PCI*), demonstrating complex brain activity similar to that observed in conscious states (Casarotto et al., 2016). This stratification highlights the possibility that a significant number (about 20 %) of VS/UWS patients may harbor covert consciousness—a residual capacity for consciousness that remains undetectable through assessments based on sensory, motor and executive functions. Subsequent studies confirmed this finding (Sinitsyn et al., 2020) and suggested that TMS-EEG based measures can detect recovery of consciousness before this is behaviorally evident (Comanducci et al., 2024). Hence, it has been proposed that TMS-EEG may play a role in assessing and predicting recovery of consciousness in the acute phase of the neurointensive care environment (Bai Y et al., 2023a, Bai Y et al., 2023b, Edlow et al., 2023).

An important caveat is that computing PCI requires high spatial sampling (at least 60 EEG channels), source modeling and complex statistics potentially limiting its application in the routine clinical setting beyond academic centers. Hence, alternative approaches and algorithms shall be developed and tested to overcome these limitations. One such example is the Perturbational Complexity Index based on State Transitions (PCI^ST^), enabling rapid estimation of the brain’s complexity response to TMS while using fewer EEG channels and significantly reducing computational loads (Comolatti et al., 2019). The comparative study demonstrated that PCI^ST^ retained a sensitivity that was only slightly below (91.9 %) of that of conventional PCI while still identifying high-complexity VS/UWS patients. Subsequent clinical studies further explored PCI^ST^ parameter selection and its potential for predicting prognosis in DOC patients (Wang Y et al., 2022). Findings confirmed that PCI^ST^ demonstrates high accuracy in diagnosing consciousness levels in MCS and VS/UWS patients, as well as in predicting consciousness recovery outcomes, with its optimal performance observed in the 9–12 Hz frequency band. Further explorations have demonstrated that PCI^ST^ might also be valuable for predicting and tracking consciousness recovery during DOC treatment. For example, baseline PCI^ST^ values showed a significant positive correlation with responsiveness to rTMS treatment, indicating that responders had significantly higher baseline PCI^ST^ values compared to non-responders (Xu et al., 2024). Additionally, during spinal cord stimulation protocols, MCS patients exhibited significant increases in TEP amplitude, natural frequency, GMFP, and PCI^ST^ as their consciousness levels improved. These changes reflect enhanced cortical excitability and oscillatory activity, with strong correlations observed between changes in baseline CRS-R scores and cortical activity (Wang et al., 2023).

The prospect of computing PCI with a reduced set of EEG channels and simplified algorithms is certainly appealing. However, achieving accurate performance and clinically meaningful results requires stringent criteria during data acquisition and preprocessing. First, magnetic and auditory-related artifacts must be minimized both during acquisition and subsequent ICA component rejection. While this can be effectively achieved with 60-channel recordings, it remains uncertain whether the same level of artifact reduction is possible with reduced montages. Second, a reliable PCI assessment depends on the initial TMS input to the cortex being highly effective, as indicated by a high-amplitude (approximately 10 µV, peak-to-peak) early (0–50 ms) component (Casarotto et al., 2022). Real-time TEP visualization software is now available to monitor and adjust stimulation parameters to ensure optimal input (Casarotto et al., 2022). Third, extracting the deterministic EEG response to TMS requires collecting a sufficient number of trials (around 150), which takes several minutes. This means that short-term fluctuations between different states of consciousness—such as wakefulness, sleep, or dreaming—will be averaged out and become undetectable. Finally, it is crucial to keep patients aroused during the measurement by applying the Coma Recovery Scale arousal protocol when necessary. Drowsiness or falling asleep during the procedure can lead to artificially low PCI values in healthy subjects and false-negative results in patients.

Besides PCI, spectral and phase threshold analyses of TEPs have revealed that VS/UWS patients exhibit a phenomenon akin to the “OFF-period” observed during sleep, characterized by a significant suppression of high-frequency brain activity (>20 Hz) (Rosanova et al., 2018). This OFF-period leads to a rapid disruption of the causal effects of TMS on local cortical activity and is significantly correlated with decreased PCI values in VS/UWS patients. Furthermore, researchers investigated the TMS induced oscillatory effect networks and discovered significant functional suppression in both the temporal and spatial dimensions of these networks in DOC patients (Bai Y et al., 2023b). Compared to healthy controls, DOC patients exhibited shorter durations of oscillatory reactivity and more restricted propagation ranges within the theta, low-beta, and high-beta frequency bands. Notably, cortical theta reactivity was positively correlated with CRS-R scores in DOC patients. A combination of the time-varying multivariate adaptive autoregressive (TV-MVAAR) model and the adaptive directed transfer function (DTF) was utilized to capture effective information flow in the TMS-EEG of DOC patients (Bai et al., 2024). The results showed that TMS-induced effective information flow in DOC patients, especially those in the VS/UWS state, was significantly lower than that in healthy individuals. Additionally, the degree of interaction in information flow across different brain regions correlated with the level of consciousness in DOC patients. Specifically, the rate of information interaction was significantly lower in VS/UWS patients compared to MCS patients. Collectively, these findings shed light on the mechanisms contributing to the loss of brain complexity in VS/UWS patients, providing deeper insights into the neurophysiological basis of consciousness disorders.

### 14.3 Summary and prospectives

In conclusion, TMS-EEG, by directly assessing the effective connectivity and information integration capabilities within thalamocortical networks, provides an objective tool for inferring covert consciousness in subjects who are disconnected from the external environment. However, its clinical application still faces challenges. For instance, the data recording setup and analysis are relatively complex, requiring specialized training and technical support, which limits its widespread use in primary healthcare settings. Additionally, the substantial individual heterogeneity among DOC patients means that interpreting TMS-EEG results requires a comprehensive analysis in conjunction with multimodal data (such as MRI and fMRI). With advancements in data processing technology, big data models, and machine learning, significant breakthroughs are expected in the clinical application of TMS-EEG as a tool to detect consciousness in unresponsive patients.

## 15 TMS-EEG in delirium

### 15.1 Introduction

Delirium is a complex neuropsychiatric syndrome that is characterized by an acute, fluctuating disturbance in attention, level of consciousness, and cognition. Delirium is a common complication, particularly in older adults after surgery or neurologic injury such as stroke, and is associated with prolonged intensive care unit and hospital stay, post-discharge institutionalization, persistent cognitive decline, loss of functional independence, and increased mortality (Fong et al., 2015, Oh et al., 2017, Yan et al., 2023). Delirium is also more common in individuals with dementia, where it accelerates cognitive decline (Fong and Inouye, 2022). Thus, better tools to identify individuals at risk for developing delirium in response to stressors are sorely needed. One fundamental limitation of current approaches to delirium is that the brain dysfunction that results in delirium in particular individuals is unclear. In 2017, Shafi et al. presented a conceptual model (Shafi et al., 2017) that delirium results from the breakdown in normal brain network dynamics by insults or stressors, and that this was more likely to occur in individuals with baseline decreased brain resilience due to abnormal connectivity and/or neuroplasticity. The authors noted that TMS-EEG can be used to determine the effective (causal) connectivity of different brain regions in individual subjects (Momi et al., 2021b, Ozdemir et al., 2020, Vink et al., 2020), and has been utilized to characterize the changes in cortical physiology in response to repetitive TMS protocols in both motor and nonmotor regions (Chung et al., 2017, Ozdemir et al., 2021a), which could serve as an index of neuroplasticity. Consequently, they suggested that TMS-EEG could be an ideal tool to test the model that delirium is a consequence of the breakdown in brain network dynamics in patients with abnormal connectivity or neuroplasticity.

### 15.2 TMS-EEG findings

Subsequently, these authors utilized TMS-EEG to evaluate cortical plasticity and connectivity in a cohort of elderly patients undergoing major elective surgery, as part of the Successful Aging after Elective Surgery renewal (SAGES II) renewal study (Hshieh et al., 2023). One of the major goals of this study is to identify neurophysiological features that identify patients at high risk for developing post-operative delirium, and thereby test the conceptual model that patients with post-operative delirium have baseline abnormal connectivity and plasticity. Prior to undergoing elective surgery, patients in this study underwent resting-state EEG; single-pulse TMS-EEG to multiple brain regions (left DLPFC node of the frontoparietal control network; left M1 motor hotspot; left angular gyrus node of the default-mode network; and left superior parietal node of the dorsal attention network) to assess effective connectivity; and intermittent theta-burst stimulation to M1 to assess the mechanisms of plasticity. An initial proof-of-concept study focused on resting-state EEG spectral power and TMS-EEG plasticity measures in the first 23 patients undergoing the TMS-EEG evaluation, 6 of whom developed post-operative delirium (Ross et al., 2023). The authors reported that 5/6 patients with delirium had baseline (pre-operative) EEG spectral features and 4/6 had baseline TMS-EEG plasticity metrics outside of the range observed in patients without post-operative delirium, providing conceptual support for their prior model. A final total of 92 participants eventually participated in the study, 12 (13 %) of whom developed post-operative delirium. In a preliminary analysis of the single-pulse TMS-EEG data (Tseng et al., 2025), the delirium group exhibited significantly increased baseline TMS-evoked activity (relative to the group that did not develop delirium) with stimulation of both the inferior parietal (default-mode network) and superior parietal (dorsal attention network) targets, but no significant differences with DLFPC stimulation, suggesting that baseline parietal hyperexcitability may be increasing the risk of post-operative delirium. Notably, some recent TMS-EEG studies have also highlighted parietal default-mode network hyperexcitability in Alzheimer’s disease (Casula et al., 2023, Maiella et al., 2024, Ozdemir et al., 2024), suggesting a potentially shared cerebral mechanism for the markedly increased delirium risk noted in patients with dementia.

**Fig. 15.1:**
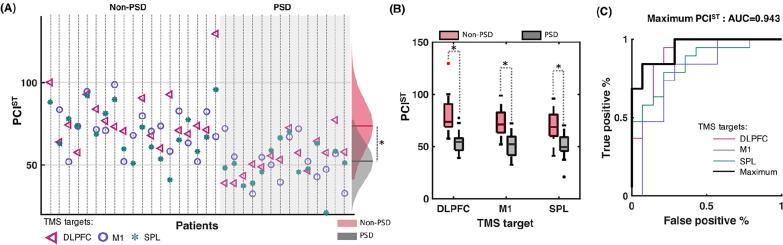
Perturbational complexity index (PCI^ST^) values in 33 S patients obtained with transcranial magnetic stimulation with electroencephalography (TMS-EEG) of three brain areas. (A) Scatter plot of individual PCI^ST^ values obtained with TMS of dorsolateral prefrontal cortex (DLPFC, pink triangles), primary motor cortex (M1, purple circles) and superior parietal lobule (SPL, green asterisks). Each vertical dotted line represents values coming from one patient. Gray area indicates patients who developed post-stroke delirium (PSD) while white area indicates patients who did not develop delirium (non-PSD). Right part of the panel displays Gaussian distributions and statistics (independent two-sample *t*-test, * p < 0.001) of PCI^ST^ values in the non-PSD (red) and PSD (grey) groups, where horizontal lines represent group averages. (B) Boxplots of PCI^ST^ values obtained with TMS of DLPFC, M1 and SPL divided into non-PSD (red) and PSD (grey) group (* indicates significance after Bonferroni correction, all *p* < 0.001; two-way rmANOVA with post-hoc independent two-sample *t*-tests). (C) Receiver operating characteristic curves of PCI^ST^ values obtained with TMS of DLPFC, M1 and SPL as well as maximum PCI^ST^ across the three TMS sites in classification of non-PSD vs. PSD groups. Area under the curve (AUC) of the maximum PCI^ST^ is indicated. Figure reproduced from (Bai Y et al., 2023a). (For interpretation of the references to colour in this figure legend, the reader is referred to the web version of this article.)

Building on the conceptual model proposed by Shafi et al. (2017) two TMS-EEG studies were conducted in acute stroke patients to test if TMS-EEG measures can predict PSD. In the first study, 33 patients (14 with PSD, 19 without) underwent resting-state EEG and single-pulse TMS-EEG targeting the right DLPFC, M1, and superior parietal lobule (SPL). Patients who developed PSD showed increased delta and decreased beta spectral power, significantly reduced TMS-evoked responses, and lower perturbational complexity index (PCI^ST^) at source level across all stimulation sites. PCI^ST^ distinguished PSD from non-PSD patients with high accuracy (lower PCI^ST^ in the PSD group, ROC-AUC = 0.943, Fig. 15.1) and was inversely correlated with delirium duration (Bai Y et al., 2023a).

To validate these findings and assess hemispheric effects, a second study (unpublished) was conducted in an independent cohort of 34 patients (13 with PSD, 21 without), this time applying TMS-EEG bilaterally to the DLPFC and SPL. Irrespective of whether stimulation was applied to the ipsilesional or contralesional hemisphere, patients with PSD consistently showed significantly lower PCI^st^ values (all p < 0.001). A two-way ANOVA confirmed the stability of this group difference across stimulation sites (no significant interaction), and the effects were independent of stroke localization. Logistic regression demonstrated that PCI^st^ was a significant independent predictor of PSD, outperforming conventional clinical and EEG-based predictors such as lesion size, NIHSS score, and resting-state theta power. The highest predictive accuracy was achieved using PCI^st^ from ipsilesional SPL stimulation.

### 15.3 Summary and prospectives

Taken together, these findings support the hypothesis that PSD is preceded by a breakdown in cortical complexity and connectivity, detectable with TMS-EEG shortly after stroke. Consistent with the conceptual framework proposed by Shafi et al. (2017), these findings demonstrate that early alterations in frontoparietal network dynamics are central to the pathogenesis of PSD. These studies highlight the potential of TMS-EEG as a non-invasive tool to identify patients at high risk for delirium—both in the postoperative and post-stroke setting—by capturing early dysfunction in large-scale brain networks.

Beyond risk stratification, TMS-EEG may also pave the way for targeted therapeutic interventions aimed at restoring network integrity in vulnerable individuals. As an example, one study demonstrated that one session of 10 Hz rTMS applied in the post-anesthesia care unit can significantly decrease the incidence of post-operative delirium in elderly patients undergoing abdominal surgery (Zhou et al., 2025). TMS-EEG could be used to identify patients at high risk for post-operative delirium, to efficiently guide preventive rTMS delivery. TMS-EEG could thus help shift delirium care from reactive symptom management toward precision-based prevention and modulation of brain network dysfunction.

## 16 Post-COVID syndrome

### 16.1 Introduction

People with corona virus disease 2019 (COVID-19) may experience post-infection sequelae including loss of smell and taste, fatigue, mental confusion (“brain fog”), dyspnea, chest and joint pain, palpitations, gastrointestinal problems, insomnia, anxiety, depression and more than 150 other possible associated symptoms (Nasserie et al., 2021). This condition, known by various names but more commonly referred to as 'post-COVID syndrome' or 'long COVID', has recently been included in the 11th revision of the International Classification of Diseases (Soriano and Ancochea, 2021). The pathogenesis of the disease may be due to several factors, among them the potential persistence of SARS-CoV-2 traces in tissues, ongoing inflammation, impact of SARS-CoV-2 on the microbiome, emergence of autoimmunity, microvascular blood clotting with endothelial dysfunction, and dysfunctional signaling in the brainstem and/or vagus nerve (Davis et al., 2023). One of the most common complaints of post-COVID syndrome is persistent fatigue (Carfì et al., 2020). Fatigue is defined as the inability to sustain or even begin physical activity, accompanied by an overwhelming feeling of exhaustion that does not improve with rest. In neurological disorders, fatigue may result from metabolic or structural brain lesions that disrupt the normal activation process of interconnected pathways among the basal ganglia, thalamus, limbic system, and higher motor and pre-motor centers (Chaudhuri and Behan, 2004).

TMS investigation based on MEPs showed the presence of alterations in excitability and neurotransmission within M1 in people with fatigue and 'brain fog' due to post-COVID syndrome (Baker et al., 2023, Ortelli et al., 2021, Ortelli et al., 2022, Versace et al., 2023, Versace et al., 2021). By means of paired-pulse TMS protocols, these studies demonstrated impairment of intracortical GABAergic activity, as evidenced by disruption of SICI and LICI mechanisms, and underactivity of intracortical glutamatergic transmission, as measured by intracortical facilitation ICF (Baker et al., 2023, Ortelli et al., 2022, Versace et al., 2023, Versace et al., 2021). Other observed alterations included increased RMT and decreased MEP amplitude in the absence of spinal or peripheral neuromuscular disease, thus indicating M1 hypoexcitability (Ortelli et al., 2022).

These observations were restricted to M1, but it is important to consider that the reduced motor excitability in post-COVID syndrome could also be related to underactivity of cortical areas upstream of M1 involved in motor planning and preparation, such as premotor cortex, supplementary motor area (SMA), cingulate motor area and basal ganglia (Inglese et al., 2004, Roelcke et al., 1997). SARS-COV-2 infection can cause (by direct entrance of the virus in the frontal lobe via olfactory pathways or by indirect effects on the brain) a frontal lobe or whole brain inflammation via cytokines and chemokines as well as microglial reactivity, leading to neuronal and glial dysregulation and resulting in neural circuit dysfunction and reduction in cortical excitability. The clinical effects of this pathological cascade could be fatigue, brain fog and other neurocognitive symptoms (Davis et al., 2023, Greene et al., 2024, Lee et al., 2022, Libby, 2024, Spudich and Nath, 2022). These aspects underline the importance of investigating cortical activity outside M1 by combining EEG recordings throughout the scalp during TMS.

### 16.2 TMS-EEG findings

So far, only one recent study directly assessed cortical activity outside M1 using a TMS-EEG approach in patients with post-COVID syndrome, compared with healthy controls (HCs) matched for age, gender, and education level (Casula et al., 2024). Here the authors explored the cortical activity throughout the scalp after stimulation of SMA and M1, two key areas involved in central fatigue. Cortical activity was assessed in the time domain, by means of TEPs, and in the time/frequency domain, by means of TMS-related spectral perturbation (TRSP). Compared to HCs, patients showed a remarkable reduction of TMS-induced oscillatory activity in the beta frequency range after stimulation of both SMA and M1, and in the gamma frequency range after stimulation of M1. Differences in beta-TRSP after SMA stimulation seemed to be particularly relevant since they were inversely correlated with the individual level of perceived fatigue.

It is well-known that beta oscillations are established in primary sensorimotor cortex during stable postures and are decreased during active states, such as movement planning and execution (Engel and Fries, 2010, Kilavik et al., 2013). Despite their robust nature and high potential value, these movement-related oscillatory responses remain poorly understood, and only few studies reported that beta-oscillations are reduced during fatiguing activities (Liu et al., 2005) and in patients with chronic fatigue syndrome (Zinn et al., 2018).

According to this perspective, the observed decrease in beta TRSP over frontal regions (Casula et al., 2024) could contribute to the generation of fatigue perception. This hypothesis is supported from the results of correlation analysis showing that higher levels of perceived fatigue were associated with lower beta TRSP when stimulating the SMA. No correlation was found between fatigue scores and beta activity in M1. This result could be explained by the higher variability shown in the TRSP when tested in M1 (Casula et al., 2018a, Koch et al., 2019) or by a more important role of the SMA in fatigue mechanisms, as suggested by several neuroimaging (Hou et al., 2016, Puri et al., 2010, van Duinen et al., 2007) and TMS investigations (Sharples et al., 2016). Reduced SMA activity can determine reduced tonic activation of M1, hypoexcitability of M1 and suboptimal neural drive to spinal alpha motor neurons, thus contributing to central fatigue.

The authors also observed reduction of gamma oscillatory activity after TMS of M1 (Casula et al., 2024). Considerable evidence from animal studies indicates that the initiation and maintenance of gamma band oscillations are closely associated with fast-spiking parvalbumin-positive inhibitory GABAergic cortical interneurons (Cardin et al., 2009, Whittington et al., 1995). Impaired activity of GABA-ergic intracortical circuits demonstrated in post-COVID patients with fatigue (Baker et al., 2023, Ortelli et al., 2022, Versace et al., 2023, Versace et al., 2021) could account for these findings. Mouse experiments showed that gamma oscillations may also originate from neuronal activity in cortico-thalamo-cortical loops (Hu et al., 2014, Russo et al., 2025b) or hippocampal neurons (Butler et al., 2016). As post-COVID patients with cognitive impairment or fatigue demonstrate thalamic (Dadsena et al., 2025, Leitner et al., 2024, Rau et al., 2025) and hippocampal (Díez-Cirarda et al., 2023) inflammation, atrophy and altered connectivity, these pathologies could also account for the observation of their reduced gamma activity after TMS. No significant between-group differences were observed in TEPs (Casula et al., 2024), a result that seems to indicate a similar cortical reactivity when assessed by phase-locked signals.

The same cohort of patients with post-COVID syndrome from the aforementioned trial was assessed for deficits in GABA-mediated intracortical inhibition in the DLPFC, a crucial region for numerous cognitive functions, particularly executive functions. The relationship between the level of cortical inhibition and cognitive abilities was also investigated. GABA_B_-mediated intracortical inhibition, as measured by LICI, was found to be disrupted in the DLPFC of patients compared to healthy controls (Versace, unpublished data). Patients demonstrated poorer cognitive performance than healthy controls, albeit still at a subclinical level, and a linear relationship was observed between LICI levels and cognitive performance.

### 16.3 Summary and prospectives

Post-infective processes may compromise the integrity of cortical inhibition, thereby diminishing the brain’s ability to generate and regulate fast task-relevant oscillations crucial for executive cognition. Deficient inhibition in the DLPFC may ultimately increase background noise, raising the energy cost of maintaining stable task representations and amplifying “brain fog” in post-COVID patients. These data highlight TMS-EEG paradigms as potentially sensitive biomarkers in post-COVID syndrome. More studies are needed to confirm the TMS-EEG findings that are currently based on only one study (Casula et al., 2024).

## 17 Alzheimer’s disease

### 17.1 Introduction

Alzheimer's disease (AD) is one of the most devastating conditions affecting elderly people in the Western world. Relatively well-defined criteria have been identified for the diagnosis of early AD, based on patients’ clinical presentation and biomarkers allowing the presence of beta-amyloid (Aβ) and tau pathology to be detected either by cerebrospinal fluid (CSF) examination or Positron Emission Tomography (PET) imaging (Dubois et al., 2014).

Despite this recent diagnostic improvement, the clinical course of AD remains heterogeneous and unpredictable. This is mainly due to a poor comprehension of the underlying pathophysiological mechanisms determining the severity of disease progression. Filling this gap of knowledge is particularly relevant in the context of upcoming clinical trials, since the development of novel biomarkers is necessary not only to increase the accuracy of early diagnosis but also and especially to track disease progression. Although several AD biomarkers are widely applied and considered useful for diagnosis, sufficient accuracy is still lacking in evaluating disease severity and predicting disease progression both considering CSF (i.e., Aβ42, t-tau, and p-tau) and neuroimaging parameters such as hippocampal atrophy/whole brain volume. Use of single biomarker provides insufficient information to capture the underlying severity of disease across its entire spectrum, from preclinical to clinical stages of AD (Jack and Holtzman, 2013). Moreover, AD biomarkers evaluation is routinely assessed by means of invasive and/or high-cost procedures, limiting their use in clinical practice. Thus, several efforts are underway to combine multiple biomarkers to predict the severity of AD with the major difficulty in tracking the temporally different evolution of each biomarker throughout the disease course (Jack and Holtzman, 2013). In recent years, several evidence supported the concept that loss of synaptic density could be an early event and precede neuronal degeneration, suggesting that the impairment of synaptic plasticity mechanisms should play a key role in the pathogenesis of AD (Selkoe, 2002). Notably, in various efforts to find semi-quantitative correlations between the progressive cognitive impairment and brain pathological alterations (e.g., the burden of cortical amyloid plaques or neurofibrillary tangles), the strongest relationship has been found between the loss of synaptic density and the degree of cognitive impairment. Thus, the impairment of synaptic transmission due to toxic oligomeric species (Selkoe, 2002), when significantly pronounced, could predict disease severity more precisely than neuronal loss, a more tardive event. Taken together, this evidence suggest that synaptic dysfunction could represent a key driver of AD-related cognitive decline rather than merely the ongoing neurodegeneration.

Within this framework, novel neurophysiological techniques could be useful to predict and track AD disease progression by providing a well-characterized estimate of cortical functioning at a certain time. Cortical plasticity, such as long-term potentiation (LTP), the main neurophysiological substrate for learning and memory, can be assessed reliably and safely in humans by means of rTMS (Huang et al., 2005). In the past years, a series of neurophysiological studies using rTMS to investigate the alteration of cortical synaptic transmission and plasticity in AD. LTP-like cortical plasticity is consistently impaired in AD patients as assessed with iTBS applied over M1 (Koch et al., 2012). Moreover, this impairment of LTP-like cortical plasticity is independent from age of disease onset and is associated with a more aggressive clinical course (Di Lorenzo et al., 2020, Di Lorenzo et al., 2016, Motta et al., 2018). The magnitude of impairment LTP-like plasticity appears to be influenced by CSF tau rather than by Aβ levels (Koch et al., 2011). Notably, the link between impaired cortical plasticity and altered CSF biomarkers levels is clinically relevant when considering that AD patients with very high CSF tau and p-tau levels exhibit a faster disease progression and higher mortality (Wallin et al., 2010). This evidence finds support on experimental studies showing that Aβ peptides and tau proteins can interfere with physiological mechanisms of neuronal synaptic plasticity in AD animal models. It has been demonstrated that these molecules influence hippocampal LTP (Lasagna-Reeves et al., 2011). These altered mechanisms have been related to spine shrinkage, neuronal network disarrangement, and cell death (Palop and Mucke, 2010).

### 17.2 TMS-EEG findings

More recently, the opportunity to assess synaptic dysfunction in the AD spectrum has been enriched by TMS-EEG (Koch et al., 2018, Koch et al., 2022). By applying a series of magnetic pulses over a target area, it is possible to map how neural signal propagates from the site of stimulation within the network in each patient (Koch et al., 2018, Koch et al., 2022). Functional and structural brain MRI can be used to integrate these neurophysiological signals within the individual anatomical and functional network (Mencarelli et al., 2024). Hence, in the context of AD, TMS-EEG is providing novel important evidence that shapes and characterizes the neurophysiological abnormalities that accompany the onset and development of cognitive dysfunction, providing the neural bases for synaptic dysfunction.

**Fig. 17.1:**
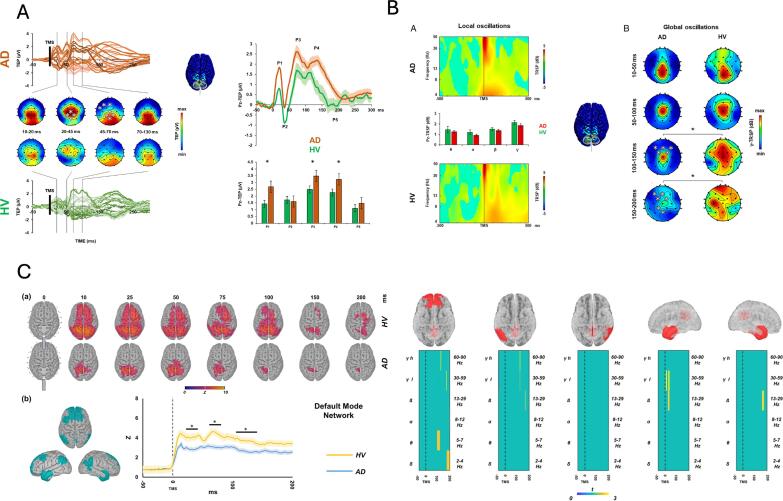
TMS-EEG recordings after precuneus stimulation in AD patients. A: Cortical excitability analysis after precuneus (PC) TMS. Left: TEPs recorded over all scalp EEG sensors after PC-TMS in the Alzheimer's disease (AD) group (upper panel) and in the healthy volunteers (HVs) group (lower panel). Reproduced from Casula et al. (2023). B: Oscillatory activity analysis after PC-TMS. Right: TEPs recorded over the PC in the AD group and in the HV group. Error bars and shaded lines indicate standard error. * Indicates p < 0.05. Left: TMS-related spectral perturbations (TRSP) recorded over the PC in the AD group (upper plot, and red bars in the middle plot) and in HV group (lower plot, green bars). Right: TRSP with scalp maps of gamma activity recorded over all scalp EEG sensors after PC-TMS in the AD group (left maps) and in the HV group (right maps). White asterisks indicate electrodes with significant group differences. *p < 0.05. Reproduced from Casula et al. (2022). C: Cortical source activations after PC-TMS for both HV and AD. Panel (a) shows the global activation for different time points (indicated above the topoplots, in ms) for both HV (upper row) and AD (lower row). Cortical source activation tracks a posterior-anterior propagation only for HV, while AD activations remain localized to the area of the stimulated PC. Panel (b) shows the time-series of the default mode network cowrtical source activations for the two groups (AD in blue, HV in yellow). The panel on the right shows coherence analyses after PC-TMS between PC and 5 ROIs for both HV and AD patients, i.e., from left to the right: PC/Frontal, PC/Parietal left, PC/ Parietal right, PC/Temporal left, PC/Temporal right. Figure shows a plot for every couple of ROIs from 50 ms before until 200 ms after the TMS pulse, with the six frequency bands (δ, θ, α, β, γ −low, γ-high) analyzed for every time point. Yellow bars indicate significantly stronger synchronized oscillations in HV than AD at this particular frequency band and time period. Reproduced from Maiella et al. (2024). (For interpretation of the references to colour in this figure legend, the reader is referred to the web version of this article.)

A first line of evidence concerns the development of neuronal hyperexcitability which occurs trough different mechanisms including the impairment of inhibitory neurons, the loss of inhibitory receptors and synapses and block of glutamatergic reuptake. Such neuronal E/I imbalance is considered a cause of neuronal network malfunctioning in AD, contributing to cognitive dysfunction (Maestú et al., 2021). In AD patients, electrophysiological measures have been used to probe network hyperexcitability, mainly for diagnostic purposes. Indeed, several neurophysiological investigations have reported that M1 excitability is abnormally increased in AD patients (Di Lazzaro et al., 2021). However, these observations are confined to M1 and therefore do not allow conclusions on hyperexcitability in associative cortical areas, which are predominantly affected during the disease progression. Recently, the TMS-EEG approach has been used to investigate the E/I balance across different brain areas related to AD, such as the DLPFC (Bagattini et al., 2019, Casarotto et al., 2011, Casula et al., 2023, Ferreri et al., 2016), the superior frontal cortex (Casarotto et al., 2011), M1 (Ferreri et al., 2016) the posterior parietal cortex (PPC) (Casula et al., 2023) and the precuneus (PC) (Casula et al., 2023). Overall, the analysis of TEPs locally to the stimulated areas showed clear hyperexcitability of the PC (Fig. 17.1A) and to some extent over the DLPFC (Bagattini et al., 2019, Casarotto et al., 2011, Ferreri et al., 2016), but not over the PPC (Casula et al., 2023). Specific to the results of PC stimulation, AD patients showed a higher TEP amplitude after the TMS pulse that likely reflects a profound disruption of synaptic activity involving different interneuronal populations mediating TEPs in the first 130 ms after TMS. Moreover, the TEP hyperexcitability measured over the PC strongly correlated with Aβ CSF levels but not with tau. Hence, although tau pathology seems to modulate cortical excitability, it does not necessarily lead to E/I imbalance (Casula et al., 2023). Further studies considering metabolic, tau and Aβ imaging with TMS-EEG are needed to better understand the possible correlation between regional cortical hyperexcitability and the underlying pathological accumulation of tau and Aβ, as compared to regional hypometabolism.

A second relevant aspect concerns the AD‐related synaptic dysfunction that has recently been linked to a disorder of high‐frequency neuronal activity. Specifically, local changes in the activation of excitatory and fast‐spiking inhibitory neurons (FSNs) that resonate in the gamma frequency modulate the activity of multiple brain areas critical for learning and memory, such as the hippocampus and the prefrontal cortex (Iaccarino et al., 2016). Along the same lines, findings showed the key role of gamma activity in ruling synaptic plasticity (Bartos et al., 2007). It was recently hypothesized that TMS‐EEG could reveal altered local gamma oscillatory activity in AD patients by measuring TRSPs over 3 different hubs of the frontoparietal network: the DLPFC, the PPC and the PC (Casula et al., 2022b). AD patients exhibit a marked reduction in frontal gamma oscillatory activity. This was observed through stimulation of various frontoparietal network hubs and direct recording of evoked cortical oscillations in a large cohort of individuals with mild-to-moderate AD. Notably, the reduction in gamma activity was evident both with direct stimulation of the DLPFC and indirect stimulation via PC (Fig. 17.1B), but not with stimulation of the left PPC, highlighting the site-specific nature of the effect (Casula et al., 2022b). Moreover, both left DLPFC gamma activity and the predominant TMS-evoked frequency emerged as reliable predictors of future cognitive decline: patients showing greater reductions in these measures experienced more pronounced deterioration over a 24-week follow-up. Also, a specific linear relationship between TMS-evoked gamma activity and other AD-related physiological markers was found. Higher frontal gamma power was associated with greater iTBS-induced LTP-like cortical plasticity and with lower levels of tau and p-tau, underscoring the potential of gamma activity as an additional biomarker of AD pathology (Casula et al., 2022b).

Notably TMS-EEG metrics have shown promise in classifying AD patients. Statistical and feature importance analyses identified the most discriminative markers between AD patients and healthy controls as the maximum amplitude of the post-TMS signal and the amplitude of the TEP within the 45–80 ms post-stimulus window. Importantly, neurophysiological alterations are not limited to patients with clinically diagnosed AD, but can also be observed in individuals with amnestic mild cognitive impairment (MCI), a condition that may precede AD. A longitudinal study investigated neurophysiological changes in the sensorimotor cortex among individuals with amnestic MCI, aiming to identify early predictors of conversion to AD (Ferreri et al., 2021). The study revealed reduced excitability in M1 and abnormal local EEG synchronization in the beta and gamma frequency bands in subjects who later progressed to AD. Moreover, a specific feature of the TEP waveform, indicative of time-sensitive cortical activity changes, demonstrated high predictive accuracy for AD conversion. Hence, these TMS-EEG metrics offer a non-invasive means to deepen our understanding of AD pathophysiology and may support both patient classification and longitudinal monitoring of disease progression (Tăuƫan et al., 2023).

A third important potential of TMS-EEG in AD relies in the study of long-range connectivity matrices. Investigating brain connectivity in AD represents one of the most promising strategies for understanding the neurophysiological and cognitive impairments associated with the condition. AD-related connectivity deficits often affect specific resting-state networks (RSNs), particularly the Default Mode Network (DMN), as demonstrated by findings from FDG-PET and functional MRI studies (Buckner et al., 2008). Core AD symptoms, such as memory impairment, have been closely linked to dysfunction within the DMN (Koch et al., 2018, Mevel et al., 2011, Veldsman et al., 2017). However, direct *in vivo* quantification of synaptic function within these networks in AD patients has remained elusive. Although techniques like FDG-PET, fMRI and EEG have been employed to study the relationship between synaptic dysfunction and connectivity in AD, they offer only indirect insights (Ding et al., 2019). Their limited temporal resolution restricts the ability to capture synaptic activity at the millisecond scale, where neuronal communication occurs. For instance, BOLD-fMRI reflects slow hemodynamic changes linked to neuronal activity and provides relative—not absolute—measures over seconds, rather than real-time synaptic events. To address this limitation, TMS-EEG has been advanced as a novel approach to track neural signal propagation in real time within the DMN of AD patients, aiming to better characterize disease pathophysiology. TMS-EEG was applied to the PC, a central hub of the DMN, and the resulting signal propagation within the network was examined in each patient. These neurophysiological data were then integrated with individual functional and structural MRI to contextualize the findings within each patient's anatomical and functional brain architecture (Maiella et al., 2024). In AD patients, the TMS pulse elicited a robust local response over the PC, revealing underlying cortical hyperexcitability (Casula et al., 2022b). However, despite this heightened local activation, the evoked signal failed to propagate efficiently to other core DMN regions (Fig. 17.1C). This impaired propagation was mapped using fMRI and tractography, which allowed to relate the disruption in signal transmission to structural and functional connectivity matrices—particularly those involving the cingulum, a major white matter tract supporting DMN integrity. This breakdown in signal propagation appeared to be specific to the DMN. When assessing the frontoparietal network (FPN) using the same TMS-EEG methodology, no significant differences in signal propagation were observed between AD patients and age-matched healthy controls (Maiella et al., 2024). Within this perspective this approach has been used to personalize stimulation and increase the precision of targeting in rTMS clinical trials, in which we developed a novel method based on the analyses of neuronavigated TMS evoked EEG activity (Koch et al., 2024, Koch et al., 2018, Koch et al., 2025, Koch et al., 2022). TMS-EEG has the unique property to directly probe local and widespread cortical dynamics, through the recording of TMS-evoked potentials. By applying a series of magnetic pulses over the precuneus, the central hub of the DMN, it is possible to determine accurately the spot from which neural signal propagates more efficiently from the site of stimulation within the network in each patient.

Finally, it is important to consider that TMS-EEG metrics, based on the above described broad neurophysiological characteristics, can be potentially used as biomarkers of response to novel therapies, especially of those acting on synaptic transmission (Koch et al., 2020).

### 17.3 Summary and prospectives

Exaggerated TEP amplitudes provide evidence for cortical hyperexcitability in AD. Reduced frontal TMS-induced activity in the gamma frequency range in AD is a possible marker for reduced capacity for plasticity induction. Furthermore, TMS-EEG revealed a breakdown of effective connectivity specifically in the DMN. This information has been utilized for rTMS clinical trials, in particular for targeting the precuneus, a central hub of the DMN. Future studies are needed to gain more information to what extent the TMS-EEG findings are already expressed in prodromal or mild cognitive impairment stages of AD.

## 18 Parkinson’s disease

### 18.1 Introduction

While Parkinson's disease (PD) is traditionally viewed as a basal ganglia (BG)-centric disorder, growing evidence highlights the involvement of cortical networks in both motor and non-motor symptoms. Non-motor symptoms such as sleep disturbances, hyposmia, mood disorders and cognitive impairments often precede motor symptoms and significantly impact quality of life (Pletcher et al., 2023, Wiesman et al., 2023, Wiesman et al., 2024). Furthermore, pathological electrophysiological changes in the BG, such as beta bursts, have been implicated in cortical areas, such as M1 (Tinkhauser et al., 2018), emphasizing that BG-cortical pathways are relevant in PD leading to cortical dysfunction throughout the disease's progression. TMS-EEG offers unique insights into cortical excitability, connectivity, and oscillatory activity, providing a window into these cortical contributions. In this section, we explore the clinical applications of TMS-EEG in PD across three key aspects: its potential role in diagnosis, its ability to characterize and decode PD clinical heterogeneity, and its utility in assessing the effects of treatments.

### 18.2 TMS-EEG in PD diagnosis

Cross-sectional studies utilizing TMS-EEG have revealed significant differences between healthy controls (HC) and PD patients, even at relatively early disease stages (Formaggio et al., 2023, Leodori et al., 2022, Leodori et al., 2024a, Maidan et al., 2021, Zifman et al., 2024). Four studies that included time-domain TEP measures reported significant differences between HC and PD (Formaggio et al., 2023, Leodori et al., 2022, Leodori et al., 2024a, Maidan et al., 2021, Zifman et al., 2024). Two studies including a total of 94 early-stage PD patients and 97 HC revealed group differences in several TMS-EEG measures recorded from M1 and DLPFC stimulation at 80 % RMT (Leodori et al., 2022, Leodori et al., 2024a, Maidan et al., 2021, Zifman et al., 2024). PD patients showed lower waveform adherence (WFA), early phase deflection (EPD), inter-trial adherence (ITA), and reduced connectivity between homologous areas compared to HCs, with these alterations observed in both investigated regions. No differences were found in late phase deflection (LPD), short term plasticity (STP), and cortical excitability, the latter measured as the area under the curve of the entire TEP, a measure similar to the GMFP (Maidan et al., 2021, Zifman et al., 2024). One study that compared PD patients at advanced stages of the disease with HCs demonstrated reduced M1 cortical excitability in the patient group as measured by GMFP, with stimulation delivered at 90 % RMT (Casula et al., 2017b). The absence of differences in cortical excitability at early stages may suggest compensatory mechanisms that deteriorate with disease progression. Another study examining 20 moderate-to-advanced PD patients in ON and OFF medication states and 19 HCs found that OFF-state PD patients exhibited smaller M1 P30 TEPs and increased pre-SMA N40 compared to HCs. These differences were not observed when patients were in the ON state, suggesting potential sensitivity of TMS-EEG measures to medication effects (Leodori et al., 2024a). In addition, a study of 28 de novo PD patients showed a reduced M1 P30 TEP amplitude compared to HCs, while pre-SMA N40 TEP amplitude remained similar between groups (Leodori et al., 2022). It is important to note that, in the latter two studies, the stimulation was delivered at 110 % RMT. Altogether, these findings suggest that TMS-EEG might be sensitive to changes in M1 excitability already at the onset of motor symptoms in PD, as well as to excitability alterations in other areas of the motor network associated with disease progression. However, it is crucial to note that these studies demonstrate group-level differences and do not yet provide evidence for individual-level diagnosis. In contrast, one study that examined frequency domain measures in 15 early-stage PD patients and 10 HCs found no specific differences in time-resolved spectral perturbation (TRSP) frequency bands in response to TMS of M1 at 110 % RMT (Formaggio et al., 2023). Conversely, an earlier study applying TMS to M1 at 120 % RMT in 7 PD patients and 11 HCs reported higher beta TRSP in PD patients compared to HCs (Van Der Werf et al., 2006). Therefore, current findings on frequency domain measures for discriminating between PD patients and HCs are conflicting and require further investigation, particularly given the potential relevance of probing beta oscillatory activity at the cortical level. The mixed results underscore the need for larger sample sizes, standardized protocols, and longitudinal investigations to better understand the specificity and sensitivity of TMS-EEG measures in PD before considering its potential as a diagnostic tool.

### 18.3 TMS-EEG to characterize clinical PD heterogeneity

Only one study investigated the potential of TMS-EEG measures by stimulating M1, DLPFC, and visual cortex (V1) to differentiate between distinct PD clinical subtypes, specifically comparing patients with tremor-dominant (TD) (n = 21), non-tremor-dominant (NTD) (n = 27), and rapid disease progression (RDP) (n = 14) subtypes (Zifman et al., 2024). Significant differences between the three subtypes were observed only for V1 stimulation, where the RDP and NTD groups showed lower WFA than TD. Moreover, TEP response to V1 stimulation was most successful in discriminating RDP from all other PD subgroups, with a ROC curve AUC of 0.85. Additionally, lower V1 interhemispheric connectivity correlated with advanced disease stage, suggesting its potential as a biomarker for disease progression. This study demonstrates the potential of TMS-EEG of non-motor areas in investigating clinical PD heterogeneity. Another study used TMS-EEG measures to better understand the pathophysiology of resting and re-emergent tremors in 10 PD patients (Leodori et al., 2020). Specific modulation in M1 P60 TEP amplitude was associated with tremor onset and suppression. These results demonstrated that M1 plays a critical role in PD tremor generation and that M1 TMS-EEG responses are sensitive to subcortical circuit dynamics. Moreover, these findings suggest that TMS-EEG can be used to characterize specific motor symptoms associated with PD, providing deeper insight into the underlying pathophysiology. It is important to note that both studies utilized stimulation intensities below motor threshold, indicating that, even at these lower intensities, TMS-EEG can effectively probe circuit dynamics that are pathophysiologically relevant in PD. Another study conducted on 26 patients with Lewy body disease, including 20 PD patients, found that those experiencing visual hallucinations showed reduced TMS-evoked cortical activation following intraparietal sulcus stimulation compared to patients without hallucinations (Leodori et al., 2023a). This further supports the utility of TMS-EEG in probing non-motor areas relevant to complex symptoms in PD. By demonstrating sensitivity to subtype-specific cortical dynamics, disease progression, and symptoms such as tremor and hallucinations, TMS-EEG emerges as a promising tool to unravel the complex heterogeneity of PD and support the development of personalized therapeutic strategies.

### 18.4 TMS-EEG to assess the effects of treatment

TMS-EEG has been utilized to assess and better understand the effects of common treatments for PD, including dopaminergic medications (Casarotto et al., 2019, Casula et al., 2017b, Leodori et al., 2024a), thalamotomy (Van Der Werf et al., 2006), deep brain stimulation (DBS) (Casula et al., 2017b, Passera et al., 2023), and rehabilitation (Pei et al., 2022). The effects of dopaminergic medications on TMS-EEG measures in PD patients have been investigated in three studies, revealing complex and localized changes in cortical excitability and neural activity (Casarotto et al., 2019, Casula et al., 2017b, Leodori et al., 2024a). The medication state appears to modulate cortical excitability in motor and premotor areas as measured by TEPs (Casarotto et al., 2019, Leodori et al., 2024a). Specifically, cortical excitability near the SMA was significantly increased by dopaminergic medication ipsilateral to the more affected brain side, with this increase being more pronounced than in the less affected hemisphere (Casarotto et al., 2019). Based on the stimulation coordinates reported in the study, the response likely also reflects coactivation of lateral premotor areas. In another study, dopaminergic medications resulted in increased M1 P30 and decreased pre-SMA N40 TEP amplitudes as compared to the OFF state, making it similar to the response of HCs (Leodori et al., 2024a). Furthermore, levodopa administration was found to increase the late P3 component of the GMFP (corresponding to the N100 TEP), which could be attributed to levodopa’s action not only on classical BG-thalamo-cortical pathways but also more directly on cortical GABABergic neurons involved in the generation of the N100 component (Casula et al., 2017b) (cf. Section 6). In addition to these localized effects, dopaminergic medications have been observed to induce broader changes in neural activity. One study has reported an increase in beta activity following levodopa administration (Casula et al., 2017b). Moreover, levodopa intake produced a further increase in late TMS-evoked activity (approximately 80–130 ms after the TMS pulse) and beta TMS-evoked oscillations (13–30 Hz) compared to OFF conditions, effectively normalizing reactivity to the range of values seen in HCs. Altogether, these findings suggest that TMS-EEG responses, particularly from motor and premotor areas, are sensitive to changes in BG activity induced by dopaminergic medication. The observed increases in excitability of premotor areas in the hemisphere with worse putaminal dopaminergic denervation further underscore the potential of TMS-EEG measures to reflect dopaminergic deficits and their modulation by dopaminergic medications in PD patients.

One study investigated the effect of thalamotomy on cortical oscillatory activity, as measured by TRSP elicited by M1 stimulation at 120 % RMT, in 7 PD patients by comparing the treated and untreated sides (Van Der Werf et al., 2006). Thalamotomy reduced beta TRSP amplitude without affecting beta phase-locking values, suggesting a key role of the thalamus in generating cortical beta oscillations through subcortical loops in PD. Two studies have investigated the effects of DBS on cortical excitability in advanced PD patients using TMS-EEG measures (Casula et al., 2017b, Passera et al., 2023). The first study examined 6 PD patients with bilateral STN-DBS, applying TMS to the left M1 at 90 % RMT (Casula et al., 2017b). They assessed GMFP of P1, P2, P3, and P4 components, as well as TRSP in high-alpha and beta bands. Results showed lower GMFP in PD patients across all components, with DBS increasing P2 GMFP. Notably, DBS and dopaminergic medications demonstrated synergistic facilitatory effects on cortical excitability. The second study, also involving 6 PD patients with bilateral STN-DBS, stimulated M1, pre-SMA, and inferior frontal gyrus (IFG) on the more affected side at 120 % RMT (Passera et al., 2023). Using Local Mean Field Power (LMFP) measures in different time windows, they found lower M1 early (10–30 ms) LMFP and higher IFG late (60–100 ms) LMFP in DBS OFF compared to ON states, with no differences observed for pre-SMA. A paired-pulse DBS-M1 paradigm revealed higher M1 early (20–60 ms) LMFP at 4 ms inter-stimulus interval. Collectively, these findings suggest that DBS facilitates M1 excitability while inhibiting IFG excitability, providing insights into the cortical effects of DBS in PD patients.

Only one study has explored the impact of multidisciplinary intensive rehabilitation treatment (MIRT) on cortical activity in PD patients using TMS-EEG measures (Pei et al., 2022). The study involved 48 PD patients who underwent a two-week MIRT program. TMS was applied to the left M1 at 90 % RMT, and GMFP and TRSP in theta, alpha, beta, and gamma bands before and after the intervention were examined. Notably, only patients who responded to the treatment showed significant changes in cortical activity. These responders exhibited a reduction in GMFP (significant at 98–104 ms) and decreased theta, beta, and gamma TRSP over right parietal and central areas. Importantly, the decrease in central beta TRSP correlated with motor improvement. These findings suggest that rehabilitation can induce cortical plasticity changes linked to both motor and non-motor improvements in PD, with beta TRSP modulation specifically relating to motor recovery. The study demonstrates that TMS-EEG can be effectively used to assess plastic changes induced by non-pharmacological interventions in PD patients, providing valuable insights into the neurophysiological effects of rehabilitation strategies.

### 18.5 Summary and prospectives

In conclusion, TMS-EEG has emerged as a promising tool for investigating cortical motor and non-motor network dynamics in PD, moving beyond the traditional focus on BG dysfunction and M1 intracortical excitability changes. The findings are summarized in Table 18.1. Cross-sectional studies have demonstrated the potential of TMS-EEG to identify group-level differences between PD patients and HC, particularly in time-domain TEP measures. While findings suggest sensitivity to cortical changes even in early-stage PD, especially with M1 and DLPFC stimulation, it is important to acknowledge the current lack of evidence for individual-level diagnosis. Studies exploring frequency domain measures have yielded less consistent results, underscoring the need for further investigation and standardized protocols. Furthermore, TMS-EEG shows promise in characterizing distinct clinical subtypes of PD, as evidenced by the identification of neurophysiological signatures associated with tremor-dominant, non-tremor-dominant, and rapid disease progression subtypes through occipital stimulation, and by its ability to detect cortical alterations underlying symptoms particularly important for disease subtyping such as tremor and visual hallucinations.

**Table 18.1:** Synopsis of TMS-EEG studies in PD.

**Authors**	**Sample**	**Region(s) of interest**	**EEG recordings**	**TMS parameters**	**Measurements/ Intervention**	**Key findings**
(Maidan et al., 2021)	32 PD 21 HC	Bilateral M1, bilateral DLPFC	32 channels SR: 5,000 Hz	SP, 0.1 Hz and 1 Hz 80 % RMT	WFA, EPD/LPD, Inter-trial adherence, M1-DLPFC Coherence / PD (ON med.) vs HCs	Lower WFA, inter-trial adherence, and coherence, and higher EPD in PD (all areas); lower DLPFC inter-trial adherence correlates with disease duration
(Leodori et al., 2022)	20 PD 19 HC	Bilateral M1, Pre-SMA	32 channels SR:10,000 Hz	SP 110 % RMT	TEP amplitude (current source density) / PD vs HCs, ON vs OFF med.	Lower M1 P30 and higher pre-SMA N40 in PD; Normalization in ON; M1 P30 correlates with bradykinesia.
(Leodori et al., 2024a)	28 de novo PD 28 HC	Most affected M1, Pre-SMA	32 channels SR:10,000 Hz	SP 110 % RMT	TEP amplitude (current source density): M1 P30, pre-SMA N40 / PD (OFF med.) vs HCs	Lower M1 P30 in de novo PD. No sig. differences in pre-SMA N40. No correlations between TEPs and bradykinesia.
(Formaggio et al., 2023)	15 mild PD, 10 HC	Left M1	32 channels SR: 5,000 Hz	Biphasic SP 110 % RMT	TRSP delta, theta, alpha, and beta bands / PD (ON med.) vs HC	No significant differences in TRSP between PD and HC
(Zifman et al., 2024)	62 PD (27 TD, 21 NTD, 14 RDP) 76 HC	Bilateral M1, bilateral DLPFC, bilateral V1	32 channels HR: 5,000 Hz	SP 85 % RMT	wWFA, IHCCONN, CEx, LPL / PD (ON med.) vs HCs; subtypes comparisons	Lower wWFA and IHCCONN in PD (all areas); lower V1 wWFA in NTD and RPD vs TD; lower V1 IHCCONN correlated with advanced disease stage.
(Casula et al., 2017b)	6 advanced PD (bilateral STN-DBS), 8 HC	Left M1	19 channels SR: 5,000 Hz	SP 90 % RMT	GMFP P1 (31 ms), P2 (66 ms), P3 (112 ms), and P4 (190 ms); TRSP high-alpha, and beta bands / PD vs HC; OFF vs. ON DBS and OFF vs. ON med.	Lower GMFP in PD (all components); DBS increased P2; medications increased P3 and high-alpha and beta.
(Van Der Werf et al., 2006)	7 PD (ventrolateral thalamotomy) 11 HC	Bilateral M1, control area outside M1	60 channels SR: 1,450 Hz	Monophasic SP 120 % RMT	TRSP and beta phase locking in the beta band, EOR delta, theta, alpha, beta, and gamma bands. / PD thalamotomy side vs non-thalamotomy side (ON med.), vs HCs.	Higher beta TRSP, EOR in PD; thalamotomy reduces both without affecting beta phase locking.
(Leodori et al., 2020)	10 PD with tremor	Most affected M1	60 channels SR:80,000 Hz	Biphasic SP 80 % AMT	TEP amplitude (limited to electrodes around M1) / Tremor conditions (rest tremor, posture holding, re-emergent tremor)	M1 P60 amplitude modulated during tremor onset and suppression.
(Leodori et al., 2023a)	12 PD (3 with VHs), 8 PDD (2 with VHs), 6 DLB (all with VHs)	Right FEF, right IPS, right V1/V2	32 channels SR:10,000 Hz	SP 160 % RMT	TMS-evoked source activation within the DAN and VIS / Patients with VHs vs without VHs	Lower TMS-evoked source activation within the DAN in patients with VHs
(Casarotto et al., 2019)	13 PD	Bilateral SMA*, bilateral SPL	60 channels SR: 1,450 Hz	Biphasic SP 120 V/m emEF	IRA: LMFP of the first two early positive and negative TEPs / ON vs OFF med.	Dopaminergic medication increased SMA IRA in the most affected side.
(Passera et al., 2023)	6 PD (bilateral STN-DBS)	Most affected M1, pre-SMA, and IFG	128 channels SR: 5,000 Hz	Biphasic SP 120 % RMT adj. to scalp-to-cortex distance	LMFP / OFF vs. ON DBS (OFF med.); Paired-pulse DBS-M1 TMS (4, 20, control 180 ms)	(All Non sig. Trends) Lower early M1 and higher late IFG LMFP in DBS OFF; no pre-SMA differences. Higher early M1 LMFP at 4 ms ISI.
(Pei et al., 2022)	48 PD	Left M1	64 channels SR: not reported	Biphasic SP 90 % RMT	GMFP; TRSP: theta, alpha, beta, gamma bands / Pre vs. Post two-week multidisciplinary intensive rehabilitation treatment	Responders show reduction in late GMFP, reduced theta, beta, and gamma TRSP. Beta TRSP correlates with motor improvement.

Abbreviations: CEx, cortical excitability; DAN, dorsal attention network; DLB, dementia with Lewy bodies; emEF, estimated maximum electric field; EOR, TMS-evoked oscillatory response; EPD/LPD, early/late phase deflection; FEF, frontal eye fields; GMFP, global mean field power; HCs, healthy controls; IFG, inferior frontal gyrus; IHCCONN, interhemispheric connectivity; IPS, intraparietal sulcus; IRA, immediate response area; LMFP, local mean field power; LPL, late phase latency; NTD, non-tremor dominant; PD, Parkinson’s disease; PDD, Parkinson's disease dementia; RDP, rapid disease progression; SP, single-pulse TMS; SPL, superior parietal lobule; SR, sampling rate; STN-DBS, subthalamic nucleus deep brain stimulation; TD, tremor dominant; TRSP, TMS-related spectral perturbation; VHs, visual hallucinations; VIS, visual network; WFA, waveform adherence; wWFA, wide-waveform adherence. *Possible co-stimulation/contribution of dorsal premotor cortex (PMd) (ed. note).

Beyond diagnosis and clinical heterogeneity, TMS-EEG has proven valuable in assessing the effects of various therapeutic interventions in PD. Dopaminergic medications, a mainstay of PD treatment, have been shown to modulate motor and premotor cortical excitability as measured by TEPs, reflecting the drug's impact on BG-cortical pathways. Additionally, TMS-EEG has been used to evaluate the effects of thalamotomy and DBS on cortical excitability and the impact of multidisciplinary intensive rehabilitation treatment on cortical plasticity and motor recovery. To fully realize the potential of TMS-EEG in PD, future research should prioritize longitudinal studies to track disease progression, larger sample sizes to enhance statistical power, and standardized protocols to improve data comparability. By addressing these limitations, TMS-EEG can further contribute to our understanding of PD pathophysiology and inform the development of more targeted and effective treatments.

## 19 Amyotrophic lateral sclerosis (ALS)

### 19.1 Introduction

Amyotrophic lateral sclerosis (ALS) is a progressive neurodegenerative disorder of the human motor system characterized by concomitant upper and lower motor dysfunction (Eisen et al., 2017, Kiernan et al., 2011, Kiernan et al., 2021, Shefner et al., 2020). Cortical dysfunction has been identified as an important feature in ALS affecting cortical regions beyond the motor areas (Dharmadasa et al., 2024, Dukic et al., 2022, Geevasinga et al., 2017, Menke et al., 2018, Petri et al., 2006). At a pathophysiological level, ALS has been postulated to be mediated by a multi-step process (Al-Chalabi et al., 2014, Chio et al., 2018, Vucic et al., 2020) with cortical hyperexcitability potentially representing an important step (Geevasinga et al., 2016). The “dying-forward” hypothesis proposes that cortical hyperexcitability mediates motor neuron degeneration via an anterograde excitonic mechanism (Eisen et al., 2017, Eisen et al., 1992, Eisen et al., 2024). The processes underlying the development of cortical hyperexcitability in ALS are complex, mediated by loss of cortical inhibitory circuits and overactivity of facilitatory neuronal populations (Eisen et al., 2024). Dysfunction of transcallosal neuronal function result in further cortical disinhibition, thereby contributing to development of cortical hyperexcitability (van den Bos et al., 2021). Understanding the nature of cortical hyperexcitability is key to unravelling ALS pathophysiology in the search for a cure.

Conventional TMS techniques, utilizing single and paired-pulse paradigms, have established dysfunction of facilitatory and inhibitory interneuronal circuits as contributory mechanisms to development of cortical hyperexcitability (Dharmadasa et al., 2024, Menon et al., 2015, Menon et al., 2016, Menon et al., 2020, van den Bos et al., 2019, Vucic and Kiernan, 2006, Vucic et al., 2023). At a neurotransmitter level, dysfunction of inhibitory GABAA-ergic inhibitory circuits (as assessed by short-interval intracortical inhibition, SICI) as well as overactivity of short latency facilitatory circuits (probed by short-interval intracortical facilitation, SICF) are evident in ALS leading to cortical dysfunction (Dharmadasa et al., 2024, van den Bos et al., 2018, Vucic and Kiernan, 2006, Vucic et al., 2023). Of relevance, cortical hyperexcitability correlates with greater functional disability, a faster rate of disease progression and is associated with reduced survival (Shibuya et al., 2016, van den Bos et al., 2018). It should be stressed that the abnormalities demonstrated in ALS patients with conventional TMS techniques provide indirect evidence for cortical dysfunction, and a contribution from lower motor neurons and spinal pathways cannot be entirely discounted (Calma et al., 2024).

### 19.2 TMS-EEG findings

In ALS, TMS-EEG has revealed abnormalities of TEPs, that are in keeping with dysfunction of GABA-ergic circuits and supporting the notion that cortical hyperexcitability is mediated by cortical disinhibition (van den Bos et al., 2024). Specifically, single-pulse TMS-EEG disclosed a significant reduction in the N100 TEP component along with marked variability of the N45 component (van den Bos et al., 2024). Given that previous pharmacological TMS-EEG studies have established that inhibitory GABAergic circuits acting via both GABAA and GABAB receptors contribute to the N45 and N100 potentials (cf. Section 6) (Premoli et al., 2014a), these abnormalities are likely mediated by dysfunction of cortical GABAergic neurotransmission. Moreover, there was reduced paired-pulse inhibition of the P60 and N100 TEP components in ALS patients, consistent with dysfunction of cortical interneuronal GABAA-ergic circuits. Further, the reduction in paired-pulse inhibition of the N100 component, was associated with longer disease duration in ALS patients (van den Bos et al., 2024).

Separately, paired-pulse TMS disclosed a significant increase in the P30 and P180 TEP components in ALS, while these components were not abnormal with single-pulse TMS-EEG studies. The precise mechanisms underlying the increase in P30 and P180 TEP components remains to be fully resolved, albeit a dysfunction across both facilitatory and inhibitory circuits has been suggested (van den Bos et al., 2018, van den Bos et al., 2024). This notion needs to be confirmed in future studies.

At a pathophysiological level, the findings of TMS-EEG studies provide support for dysfunction of GABAergic circuits and thereby the importance of cortical disinhibition in the development of hyperexcitability in ALS. Cortical disinhibition appears to be mediated by dysfunction of GABA-ergic interneuronal circuits acting via both GABAA and GABAB receptors, although a concomitant dysfunction of facilitatory circuits seems likely. Importantly, TMS-EEG findings demonstrating a correlation of N45 potential reduction with increasing muscle weakness and the loss of N100 paired pulse modulation with increasing disease duration confirm pathophysiological significance (van den Bos et al., 2024).

Of relevance, TME-EEG abnormalities corroborate the findings of multiple studies utilizing the threshold tracking TMS technique whereby a reduction of SICI reduction (representing cortical disinhibition) was established to be a specific feature of ALS (van den Bos et al., 2019, van den Bos et al., 2018, Vucic and Kiernan, 2006), correlating with motor neuron degeneration (Vucic and Kiernan, 2006), and associated with an adverse prognosis (Shibuya et al., 2016), patterns of disease progression (Dharmadasa et al., 2020, Menon et al., 2018, Menon et al., 2019) and clinical features such as the split hand and phenomenon (Menon et al., 2014).

There is emerging genetic and molecular evidence for dysfunction of inhibitory GABAergic inhibitory circuits (Maekawa et al., 2004, Nihei et al., 1993, Zhang et al., 2016), which could account for the TME-EEG abnormalities. Degeneration of parvalbumin-positive interneurons (Maekawa et al., 2004, Nihei et al., 1993, Zhang et al., 2016) and reduced expression of mRNA GABAA receptor subunits in the primary motor cortex in the ALS (Petri et al., 2003) have been reported. Additionally, brain PET studies (using 11C-flumazenil) have established widespread loss of GABAA receptor binding in ALS patients (Turner et al., 2005, Wicks et al., 2008, Yabe et al., 2012), with the genomic risk for developing ALS consistently mapping to GABAergic interneurons and oligodendrocytes (Saez-Atienzar et al., 2021). Transgenic ALS mouse model studies have reported that functional inhibition of parvalbumin-positive inhibitory interneurons leads to development of hyperexcitability (Zhang et al., 2016), and modulation of the dysfunctional parvalbumin-positive interneurons resulted in a delay of disease onset and prolongation of lifespan (Khademullah et al., 2020). Consequently, mechanisms that restore cortical inhibition could prove therapeutically useful in ALS.

### 19.3 Summary and prospectives

The potential advantage of TMS-EEG compared to conventional TMS-EMG measures of impaired cortical inhibitory neuronal function relates to direct assessment of cortical components with the bypassing of a contribution from brain stem and spinal cord. TMS-EEG also enables assessment of cortical function in the setting of marked muscle wasting that precludes reliable recording of MEPs. Although TMS-EEG is technically more challenging than TMS-EMG, advances in technology that enable a more rapid and precise recording of TEP signals, automated online processing and analysis will enable translation into a clinical setting to serve as biomarkers of target engagement and therapeutic efficacy in future therapeutic trials (Kiernan et al., 2021).

## 20 Stroke

### 20.1 Introduction

Stroke constitutes a major cause of disability worldwide, especially due to motor, language and cognitive deficits impacting on functional independence and the activities of daily living (Feigin and Owolabi, 2023, Grefkes and Fink, 2020). All along, the field has been aiming at understanding plasticity mechanisms following stroke in order to promote recovery of function (Hensel et al., 2019, Micera et al., 2020, Raffin and Hummel, 2018, Volz et al., 2016). Despite compelling evidence that stroke as a focal lesion leading to confined clinical symptoms reflects widespread network-related abnormalities, it is still largely comprehended as a local dysfunction in the clinical context.

Brain stimulation techniques, such as rTMS and tDCS targeted to ipsi- or contralesional areas, are increasingly used as adjuvant therapies to promote plasticity and recovery of function. Although promising, these approaches are limited in their therapeutic effects by numerous factors, comprising lack of knowledge about the underlying circuitries and their relevance to symptoms; a strategy for mapping circuit abnormalities in single patients; and, thus most importantly, an individualized understanding of where and how to stimulate the brain.

### 20.2 TMS-EEG findings

Recently, TMS–EEG has greatly contributed to the understanding of the neurophysiological aftermath of stroke, offering crucial insights into the pathophysiology of stroke-induced network alterations (Massimini et al., 2024). Furthermore, TMS-EEG allows a standardized read-out of the integrity of specific neuronal networks of stroke patients, and this information may provide targets for guided brain stimulation approaches used for rehabilitation.

TMS-EEG in patients recovering from acute stroke has revealed the presence of substantial functional alterations in the cortical tissue surrounding ischemic brain lesions. Specifically, TMS applied to both motor and non-motor areas revealed the presence of full-fledged sleep-like responses to TMS in awake stroke patients, marked by a prominent high-amplitude slow wave and by a suppression of high-frequency activity in response to TMS to perilesional brain regions (Bai Y et al., 2023a, Brancaccio et al., 2025, Harquel et al., 2024, Sarasso et al., 2020, Sarasso et al., 2025, Tscherpel et al., 2020, Tscherpel et al., 2024). Such neurophysiological fingerprints are the characteristic hallmarks of physiological sleep and anesthesia where EEG slow waves are underpinned by neuronal silent OFF-periods (see Section 6). Notably, these cortical OFF-periods are associated with the disruption of causal interactions at the level of network nodes (Sarasso et al., 2020, Sarasso et al., 2025, Tscherpel et al., 2020, Tscherpel et al., 2024).

Importantly, and in contrast to the conditions of sleep, anesthesia or disorders of consciousness, the sleep-like slow wave in stroke is a local phenomenon of the ipsilesional functional network (Tscherpel et al., 2020, Tscherpel et al., 2024) or even the perilesional cortical tissue (Sarasso et al., 2020).

Moreover, these signal alterations have been associated with the individual patients’ initial motor deficit. Of note, TMS-EEG even captures the individual trajectories of motor recovery several months after stroke onset (Harquel et al., 2024, Sarasso et al., 2025, Tscherpel et al., 2020). This implies that slow waves and cortical OFF-periods might represent the electrophysiological expression of a pathophysiological condition hindering functional reorganization (Tscherpel et al., 2024) and, thus, highlighting the utility of TMS-EEG as a non-invasive readout of the potential for functional recovery.

Supporting this relevant possibility is that the reversibility of perilesional sleep-like cortical dynamics (Sarasso et al., 2025, Tscherpel et al., 2024) was associated with the recovery of local cortical interactions as measured by local indices of PCI (Comolatti et al., 2019). Their link to recovery has been, however, inconsistent across studies: While the reversibility of PCI was not related to an improvement in motor recovery in one study (Tscherpel et al., 2024), another study found a relationship between the increase in PCI and the overall clinical improvement as measured by NIHSS (Sarasso et al., 2025). Such difference may be explained by the structural damage to the corticospinal tract (CST) which typically characterizes patients affected by poor motor recovery. As the CST constitutes the principal output pathway linking cortical motor activity to spinal motor neurons, its structural integrity is a necessary condition for translating cortical reorganization into functional motor gains.

Notably, TMS-EEG measurements revealed the presence of such sleep-like cortical dynamics even in those patients where slow waves were not immediately observable in the ongoing EEG, thus highlighting the usefulness of probing the input–output properties of cortical neurons by means of TMS-EEG to assess the presence of relevant circuit alterations above and beyond spontaneous EEG activity (Sarasso et al., 2025).

Although slow waves have been shown to be present for subcortical and cortical stroke lesions, at least very early after stroke (Tscherpel et al., 2020, Tscherpel et al., 2024), subcortical lesions may reflect a more nuanced slowing of TEPs. As an example, in a study by Pellicciari and colleagues ipsilesional TEPs featured a sequence of positive and negative polarity deflections with a slowing of EEG activity and a reduced amplitude of fast frequency oscillatory components in the subacute phase of subcortical stroke (Pellicciari et al., 2018). One other study also found an overall slowing of TMS-induced oscillations, i.e., the natural frequencies in ipsilesional and contralesional cortex of subacute and chronic subcortical stroke compared to healthy controls (Sarasso et al., 2020).

Supporting the concept that focal structural lesions impact whole brain network properties, a number of recent TMS-EEG studies corroborates the long existing concept of diaschisis (Carrera and Tononi, 2014, von Monakow, 1914), i.e., functional alterations of structurally unaffected brain regions connected to the site of the lesion (Grefkes and Fink, 2020). In this vein, a recent report combined TMS-EEG with magnetic resonance imaging (MRI)-derived quantitative estimates of structural disconnection in a single case study of an acute unilateral subcortical stroke (D'Ambrosio et al., 2023). This allowed the identification of remote cortical TMS targets with the highest disconnection probability. TEPs recorded from these targets showed the occurrence of a pathological, high-amplitude EEG slow wave, similar to those described for responses obtained from perilesional cortex. Notably, these abnormal TMS-EEG responses were site-specific, and normalized when TMS was applied outside of the disconnected target site. Corroborating their sleep-like nature, such slow wave responses to TMS were found associated to lesions resulting in a disconnection from the ipsilesional pedunculopontine tegmental nucleus, a component of the brainstem ascending activating systems located in the pontomesencephalic reticular formation (Tscherpel et al., 2024).

Altogether, these studies offer a reappraisal of the long-standing finding of EEG slow waves after brain injury (Walter, 1937). According to a recent perspective, such intrusion of postlesional sleep-like cortical dynamics in the awake brain represents the key mechanistic element for the network and behavioral consequences of focal brain injuries which are best revealed through direct cortical perturbations (Massimini et al., 2024).(Rolle et al., 2021, Tecchio et al., 2023)Further corroborating this view, in a recent study TMS-EEG derived connectivity and graph theory were used to generate dynamic connectivity analyses to longitudinally explore cortical connectivity alterations and reorganization after stroke, extending previous fMRI findings (Tscherpel et al., 2024). Here, a stroke-induced enhancement of slow activity between frontocentral and parietal regions was demonstrated, which not only fits with sleep-like slow waves after stroke but also provides further evidence to put the findings of stroke-induced slow wave activity in the context of diaschisis. Notably, the increase of slow oscillations was accompanied by a loss of the physiological network architecture, and this disruption early after stroke was predictive of motor outcomes at 3 months follow-up: recovery of motor function and cortical reorganization were associated with the normalization of increased low-frequency coupling and the restoration of complex network structures.

In addition, TMS-EEG derived indices of functional motor network connectivity (i.e., the weighted phase lag index from TMS of the contralesional M1) showed a significant relation to motor recovery in chronic stroke patients (Rolle et al., 2021). In line with that, Tecchio and colleagues used TMS-EEG measures of bilateral M1 in subacute stroke patients and showed increased excitability, i.e., larger GMFPs, in the ipsilesional hemisphere compared to the contralesional hemisphere and healthy controls (Tecchio et al., 2023).

Another example of how alterations of TEPs directly reflect diaschisis stems from a case report of cerebellar TMS in combination with EEG allowing to assess the connectivity between the cerebellum and cerebral cortex (Gassmann et al., 2023). TMS applied to the cerebellum in a chronic stroke patient revealed an absence of an early frontal cerebellum-TMS-EEG response, likely as a consequence of the secondary degeneration of the ipsilateral dentato-thalamo-cortical tract.

Abnormal cortical reactivity was also found in a single case of focal brain hemorrhage (Russo et al., 2022a). Here, TMS was targeted over the hemorrhagic region as well as over the homologous contralateral area. At odds with the absence of a TMS-EEG response when targeting necrotic brain tissue in three earlier cases (Gosseries et al., 2015), it was found that TMS of the hemorrhagic core triggered a pathological TEP characterized by reduced amplitude compared to the contralateral side. This TEP alteration closely resembled that obtained when stimulating the peritumoral and tumoral areas in a case of glioblastoma (Meneghini et al., 2025). Interestingly, both the reabsorption of blood masses in the first case and the surgical resection of the tumor in the other resulted in a marked increase in TEP amplitude associated with the patients’ functional improvement. Both these studies are relevant in two main respects: first, by directly probing the electrophysiological reactivity of the underlying cortical tissue, TMS-EEG can represent a valuable alternative to capture the residual integrity of cortical areas when their assessment by imaging is prevented by hyperdense signals; in addition, these cases corroborate the role of TMS-EEG as a potential readout for predicting and tracking functional recovery after brain injury.

Another focus of TMS-EEG studies after stroke has been the investigation of the impact of stroke on intra- and interhemispheric neurotransmitter systems and their functional role for recovery. Among the proposed mechanisms, modulation of intracortical inhibition within the ipsi- and contralesional hemisphere is thought to constitute a pivotal determinant of post-stroke recovery (Clarkson et al., 2010, Hummel et al., 2009, Joy and Carmichael, 2021, Liuzzi et al., 2014, Paparella et al., 2023). Shifts in the E/I balance can either facilitate or impede rehabilitation, depending on both their direction and timing. In the hyper-acute phase after a stroke event, upregulation of GABAergic activity helps to limit excitotoxic damage; however, persistence of elevated inhibition into later stages can impair reorganization and recovery. By contrast, a transient disinhibitory window appears to foster neuroplasticity and reorganization by means of e.g., dendritic sprouting and synaptogenesis (for review, see e.g. (Joy and Carmichael, 2021). Here, TMS-EEG is also emerging as a potentially powerful tool to investigate intracortical and cortical network dynamics, including paired-pulse and cortical silent period protocols (Bai Z et al., 2023a, Cinnera et al., 2025, Gray et al., 2017, Harquel et al., 2024). TMS-EEG allows to determine changes in intracortical inhibition, interhemispheric communication, and cortical reactivity associated with motor outcomes even in patients, in which common electrophysiological measures such as MEPs cannot be elicited (Tscherpel et al., 2020, Tscherpel et al., 2024).

One study applied TMS-EEG to the ipsilesional M1 of stroke patients and evaluated it from the acute to the late subacute phase three months after the stroke event to track cortical reactivity and GABAergic intracortical inhibition longitudinally (Harquel et al., 2024). The authors found that heightened motor cortical excitability in the acute phase, indexed by low-complexity high-amplitude TEPs, was linked to greater initial impairment, whereas a transient disinhibition, assessed in a paired-pulse TMS-EEG protocol, during the first weeks post-stroke promoted plasticity along with subsequent normalization of intracortical inhibition. This was linked to better motor recovery, especially of distal upper extremity functions. A complementary analysis performed on the same cohort used a data-driven PARAFAC decomposition of TMS-induced oscillations (see Section 5) across recovery stages and observed a beneficial decrease of late TMS-induced alpha waves, linked to the inhibitory system activity (Harquel et al., 2025). Together, these converging results suggest that post-stroke disinhibition appears to unfold in two stages: initially, a localized reduction of inhibition within the ipsilesional motor cortex during the acute-to-early subacute period, followed by a more widespread disinhibitory phase in the late subacute stage.

Changes in the E/I balance are not locally limited to the ipsilesional motor cortex but do impact also longer-range interactions in the motor system, such as interhemispheric interactions between motor cortex homologues. Recent work explored the association between interhemispheric balance of TMS-evoked EEG responses and upper limb impairment in stroke, supporting the view that higher excitability in the contralesional relative to the ipsilesional hemisphere correlated with poorer motor outcomes, suggesting that excessive contralesional activity may impede recovery (Borich et al., 2016, Cinnera et al., 2025).

Together, these studies underscore the value of TMS-EEG in capturing stroke-related changes in cortical excitability and connectivity. They reveal dynamics of changes of the E/I balance that are directly linked to motor impairment and recovery.

### 20.3 Summary and prospectives

In conclusion, the studies discussed above demonstrate the potential of TMS-EEG to assess brain function across different phases after cerebral stroke. From the early detection of the recovery potential to chronic network-level disruptions, TMS-EEG might provide a non-invasive window into post-stroke neurophysiology even allowing to determine intracortical neurotransmitter systems. In the future, these findings may inform precision neurorehabilitation strategies tailored to individual brain responsiveness, ultimately improving outcomes after stroke. Future studies should aim to validate read-outs in larger cohorts, and explore how rehabilitation interventions modulate cortical dynamics in association with recovery.

## 21 Mild traumatic brain injury

### 21.1 Introduction

Mild Traumatic Brain Injury (mTBI) refers to a head injury defined by the temporary disruption of brain activity resulting in confusion, memory loss, and/or loss of consciousness of no more than 30 min (Maas et al., 2022). mTBIs are common, representing up to 90 % of all traumatic brain injuries, and even though they result in less obvious symptoms than severe injuries they do have persistent and disabling sequalae (Gardner and Yaffe, 2015, Maas et al., 2022). Indeed, up to 20 % of people with mTBI experience persistent impairment across physical, emotional, and cognitive functioning (Dery et al., 2021). However, there is significant individual variation in both the presentation and persistence of these symptoms post injury, which represents a major challenge for mTBI diagnosis and prognosis (Coyle et al., 2018, Dery et al., 2021). A key reason for this being incomplete knowledge of the factors contributing to variation in outcomes *and* of exactly how mTBI pathophysiology maps onto mTBI symptomology (Coyle et al., 2018, Dery et al., 2021). The complex pathophysiology of mTBI is beyond the scope of this review (for detailed reviews see (MacFarlane and Glenn, 2015, Naumenko et al., 2023)), however broadly speaking mTBI results in physical shearing forces which cause a cascade of neurometabolic effects resulting in, among other things, E/I imbalance as well as impairments in both structural and functional connectivity (Coyle et al., 2018). TMS-EEG has emerged as a potentially valuable tool with which to investigate these brain changes and their association with persistent symptoms.

### 21.2 TMS-EEG findings

At the time of the 2019 review (Tremblay et al., 2019) there were two studies, which had used TMS-EEG to investigate brain activity following mTBI (Bashir et al., 2012, Tallus et al., 2013), since then there have been another five papers published (Coyle et al., 2023a, Coyle et al., 2023b, Coyle et al., 2023c, Levy-Lamdan et al., 2020, Opie et al., 2019) (Table 21.1). Opie et al. (2019) used TMS-EEG to examine intracortical inhibition and neuroplasticity in 17 adults with mTBI (sustained within the previous 24 months) and 15 healthy controls. Intracortical inhibition was assessed using standard paired-pulse paradigms of short-interval intracortical inhibition (SICI) and long-internal intracortical inhibition (LICI). They also examined neuroplastic responses in mTBI by providing continuous theta-burst stimulation (cTBS) applied to the left primary motor cortex and measuring TEPs pre and 0, 30 mins post stimulation. For TEP measures of SICI and LICI both the mTBI and the healthy control groups showed amplitude reductions in the N100 and the P200, while SICI resulted in modulation of the N45 again in both groups; there were no significant differences between groups. Following cTBS the mTBI group showed significant inhibition of the N45, and a trend towards inhibition of the P30, both at the 30 min post time point. There were no TEP changes following cTBS for the healthy control group, and there were no significant differences between the groups at any timepoint. Finally, N45 modulation following cTBS in the mTBI group was found to be significantly associated with time since injury. The authors concluded that while the TEP measures of cortical inhibition were suggestive of GABA_A_ and GABA_B_ receptor-mediated inhibition being unaffected by mTBI, the effect of cTBS on the N45 in mTBI was suggestive of changes in the plasticity of GABA_A_ networks (Opie et al., 2019).

**Table 21.1:** Synopsis of TMS-EEG studies in mTBI.

**Authors**	**Sample**	**Region(s) of interest**	**EEG recordings**	**TMS parameters**	**Measurements/** **Intervention**	**Key findings**
(Bashir et al., 2012)	1 mTBI12 HS	Left M1	Not reported	Monophasic SP	TEPs Week 2 and 6 post mTBI Pre and 0, 5, 10 min postcTBS	(1) Widespread but less intense TMS-EEG activity 50 ms after TMS pulse at week 2 and 6 (2) cTBS increased this activation at week 2 (compared to a decrease in HS), while no change was observed at week 6
(Tallus et al., 2013)	11 mTBI (symptomatic) 8 mTBI(asymptomatic) 9 HS	Left M1Left DLPFC	60 channels SR: 1,450 Hz	Monophasic SP (90–100–110 % of RMT)Neuro-navigation	TEPs	(1) DLPFC, symptomatic: delayed ipsilateral P30 and contralateral N45, and increased amplitude of N100 (2) DLPFC, asymptomatic: decreased P200 latency (3) M1, both mTBI groups: less P30 amplitude increase to increasing TMS intensities (4) M1, both mTBI groups: P60 interhemispheric latency difference with higher stimulation intensities
(Opie et al., 2018)	17 mTBI15 HS	Left M1	62 channels SR: 2,048 Hz	Paired pulse: CS at 70 %RMT, TS at MEP_1mv_ Single pulse: at MEP_1mv_	ICI TEPs TEPs pre and 0, 30 min post cTBS	(1) No difference in inhibition of TEP for LICI or SICI between groups (2) Increased inhibition of P30 and N45 following cTBS in mTBI. (3) N45 modulation related to time since injury
(Levy-Lamdan et al., 2020)	39 S 14 TBI70 HS	Left M1	32 channels SR: 5,000 Hz	SP 80 % RMT	DELPHI analysis of TEPs using WFA, EPD, LPD, STP)	(1) Widespread decrease in WFA and significant differences in left temporal and parietal EPD in the TBI group (2) DELPHI TEP measures classified TBI from healthy controls at 0.83 sensitivity and 0.81 sensitivity (3) (3) Regression model of DELPHI TEP measures successfully predicted DTI changes in white matter tracts
(Coyle et al., 2023a)	30 mTBI28 HS	Left DLPFC	50 channels SR:10,000 Hz	Biphasic SP 110 % RMT	TEPs	(1) Greater right fronto-central N100 amplitude in mTBI (2) Greater left parietal-occipital N100 amplitude in mTBI (3) Smaller left parietal-occipital P60 amplitude in mTBI (4) No significant correlations between TEPs and clinical or cognitive measures found
(Coyle et al., 2023c)	30 mTBI28 HS	Left DLPFC	50 channels SR:10,000 Hz	Biphasic SP 110 % RMT	P60 and N100 TEPs assessed longitudinally: - Subacute (within 1 month of mTBI) - 3 month follow up - 6 month follow up	(1) Smaller left parietal-occipital P60 amplitude at the subacute and 6- month follow-up timepoints for mTBI (2) Greater right fronto-central N100 amplitude at all timepoints for mTBI
(Coyle et al., 2023b)	30 mTBI 28 HS	Left DLPFC	50 channels SR:10,000 Hz	Biphasic SP 110 % RMT	TEPs assessed pre and post single iTBS session at 3 timepoints: - Subacute (within 1 month of mTBI) - 3 month follow up - 6 month follow up	*Subacute Time point*(1) iTBS induced a reduction of N45 amplitude in mTBI, greatest differences in fronto-central region(2) No TEP changes following iTBS for HS(3) No differences in TEPs following iTBS between mTBI and HS *3 month follow up*(4) No TEP changes following iTBS for mTBI or HS *6 month follow up*(5) iTBS induced an increase in P200 amplitude in mTBI, greatest differences in left frontal region(6) iTBS induced a change in N45 amplitude in HS greatest differences in right parietal region(7) iTBS resulted in a significantly different change in N45 amplitude between mTBI and HS.

Abbreviations: cTBS, continuous theta-burst stimulation; DLPFC, dorsolateral prefrontal cortex; DTI, diffusion tensor imaging; HS, healthy subject; EPD, early phase detection; iTBS, intermittent theta-burst stimulation; LICI, long-interval intracortical inhibition; LPD, late phase detection; M1, primary motor cortex; RMT, resting motor threshold; SICI, short-interval intracortical inhibition; STP, short-term plasticity; WFA, waveform adherence.

Levi-Landman et al. (2020) investigated the utility of TMS-EEG TEPs for evaluating structural and functional connectivity damage in stroke and TBI, including 14 post-acute TBI patients (severity not specified), and 70 healthy controls. Specifically, they compared Diffuse Tensor Imaging data (DTI: measure of integrity of white matter tracts in the brain), to TMS-EEG TEP data analyzed using the automated Direct Electrophysiology Imaging (DELPHI) approach (Zifman et al., 2019). The DEPLHI approach analyses TEPs with a view to quantifying network integrity and plasticity, and in this study the DELPHI TEP measures included the early phase detection (EPD: the slope of the early response), the late phase detection (LPD: the slope of the late response), the waveform adherence (WFA: the TEP response adherence to healthy control reference data) and a measure of short-term plasticity (STP) (Levy-Lamdan et al., 2020). The authors reported significant differences between the healthy control and the TBI group, namely a widespread decrease in WFA and significant differences in left temporal and parietal EPD in the TBI group. The authors also investigated the classification performance of the DELPHI measures, finding the TEP measures were able to classify TBI from healthy controls at 0.83 sensitivity and 0.81 sensitivity (Levy-Lamdan et al., 2020). Finally, a regression model of the DELPHI TEP measures showed successful prediction of DTI measured changes in white matter tracts, with strongest correlations in frontal corpus callosum, anterior internal capsule, and the fronto-occipital fasciculus. The authors suggest their results show that TEPs can be used to measure and track changes in white matter integrity, with implications for the diagnosis and monitoring of TBI-related brain change.

The final three mTBI papers each form part of a single longitudinal study (Coyle et al., 2023a, Coyle et al., 2023b, Coyle et al., 2023c). The study included 58 participants (30 mTBI patients within 4 weeks of injury and 28 demographically matched controls). mTBI participants were assessed at three timepoints post injury (4 weeks, 3 months, and 6 months), with controls assessed across the same timeframe. At each assessment timepoint, left dorsolateral prefrontal cortex (DLPFC) TMS-EEG was provided pre and post a single session of left DLPFC iTBS, with TMS-EEG data analysis again focusing on TEPs (Coyle et al., 2023a, Coyle et al., 2023b, Coyle et al., 2023c). At four weeks post injury mTBI patients exhibited greater right fronto-central and left parietal-occipital N100 amplitude, and smaller left parietal-occipital P60 amplitude compared with controls; with no correlations between TEPs and clinical/cognitive measures (Coyle et al., 2023a). Following iTBS at this time point, mTBI patients exhibited a reduction of the N45 amplitude, most prominently in fronto-central regions; healthy controls showed no TEP changes following iTBS (Coyle et al., 2023b). At the subsequent timepoints, 3 and 6 months, focusing on the between group TEP changes identified at 4 weeks post injury, the authors reported that the greater right-fronto central N100 amplitude seen in mTBI persisted across all time points. The smaller left parietal-occipital P60 amplitude in mTBI was seen at the 4 week and the 6-month time points, while the greater left parietal-occipital N100 amplitude in mTBI was only seen at 4 weeks (Coyle et al., 2023c). With respect to clinical/cognitive measures only for measures of fatigue did group differences persist across all timepoints (Coyle et al., 2023c). Finally, with respect to response to iTBS over time, at the 3-month timepoint there were no changes in TEPs following iTBS for either the mTBI or the healthy control group (Coyle et al., 2023b). While at the 6-month time point iTBS induced several changes in TEPs, namely in the mTBI group increased left frontal P200 amplitude and in the healthy control group modulation of the N45 amplitude most prominent in the right parietal region (Coyle et al., 2023b). Overall, the authors concluded that although post concussive and mood symptoms resolve by 3 and 6-month follow-ups the persistent neurophysiological changes and greater fatigue suggests that mTBI may affect neural communication in a way that requires increased mental exertion to maintain normal cognitive performance.

### 21.3 Summary and prospectives

To date there have been only seven TMS-EEG in mTBI published experimental papers across five studies, with a total of 81 patients. While most of these studies focused on TEPs, the considerable variation in methodologies and specific outcome measures limits the ability to make any firm conclusions from the small body of research to date. Overall, these studies support the utility of TMS-EEG for assessing relevant changes in brain activity following mTBI, including cortical inhibition and excitation, connectivity, and plasticity; as well as in assessing recovery of brain activity over time post injury. Therefore, while TMS-EEG still holds potential in mTBI, particularly with respect diagnostic information as well as individualized prognostic potential, the evidence base remains lacking.

## 22 Multiple sclerosis

### 22.1 Introduction

Multiple sclerosis (MS) is a chronic inflammatory disease of the central nervous system characterized by demyelination and neurodegeneration (Thompson et al., 2018). While high-efficacy disease-modifying therapies can effectively reduce relapse rates and inflammation, they often fail to prevent progression independent of relapse activity (PIRA) (Kappos et al., 2020). Identifying biomarkers that capture early pathogenic changes associated with disease progression, before irreversible damage occurs, is crucial.

Studies have shown that neuroinflammation leads to synaptopathy, disrupting neurotransmission and impairing functional brain networks (Chard et al., 2021). Synaptic dysfunction contributes to altered cortical excitability, maladaptive plasticity, and impaired connectivity between brain regions, all of which are implicated in MS-related disability and progression (Stampanoni Bassi et al., 2022). While MRI has been instrumental in detecting structural damage in MS, it has limitations in identifying functional alterations in brain networks, particularly those driven by synaptic dysfunction. TMS-EEG provides a unique approach to study cortical network dysfunction in MS. TMS-EEG offers real-time assessment of cortical excitability and effective connectivity by directly stimulating brain regions and recording the resulting neural responses. This technique allows for evaluating synaptic function and network dynamics, making it a potentially valuable tool for detecting early changes associated with MS progression.

### 22.2 TMS-EEG findings

Despite the clear potential of TMS-EEG in assessing network alterations, so far only three studies have systematically explored its application in MS.

Zipser et al. (2018) investigated TEPs from M1 stimulation (100 % RMT) in relapsing-remitting MS (RRMS) patients with early-stage disease and minimal disability compared to healthy controls (HCs). Their findings indicated that while TEP latencies and interhemispheric signal propagation were preserved, MS patients exhibited an exaggerated N280 amplitude, suggesting altered long-range cortical excitability and connectivity (Zipser et al., 2018). This study provided early evidence of subtle disruptions in cortical network dynamics preceding significant disability. Furthermore, it reinforced the utility of TMS-EEG in detecting functional impairments that occur before observable structural degeneration, highlighting its potential as an early biomarker for disease progression. However, it is relevant to note that components of the TEP occurring before 45 ms were not analyzed, potentially limiting the ability to detect early cortical excitability changes. In addition, no sham control was implemented. Therefore, activation by auditory or somatosensory inputs generated by the TMS pulse may have contributed to the observed N280 abnormalities.

A study by Leodori et al. (2023) examined motor fatigue mechanisms using a multimodal approach combining neuromuscular assessment and TMS-EEG. The study included RRMS patients and HCs who underwent a fatiguing motor task while TEPs from M1 stimulation (90 % RMT) were assessed before and after exertion. The results revealed that MS patients exhibited an abnormal increase in global mean field power (GMFP) and TMS-evoked source-reconstructed activity in the sensorimotor cortex after fatigue, whereas HCs showed a decrease (Leodori et al., 2023b). Furthermore, the abnormal increase in TEPs after fatigue correlated with supraspinal (i.e., cortical) fatigue indices measured through neuromuscular assessment, reinforcing the notion that cortical dysfunction plays a key role in MS-related fatigue (Leodori et al., 2023b). This suggested that motor fatigue in MS is associated with dysfunctional network regulation rather than impaired corticospinal transmission. The inability to appropriately modulate cortical excitability in response to exertion suggests an underlying alteration in plasticity mechanisms that contribute to persistent fatigue, a common and debilitating symptom in MS. The study further confirms the potential of TMS-EEG in identifying abnormal network dynamics underlying disabling symptoms such as fatigue in MS, thereby providing valuable insights for developing novel therapeutic interventions.

A subsequent study by Leodori et al. (2024) investigated the effects of natalizumab treatment on motor fatigue and motor cortical reactivity using TMS-EEG in MS patients experiencing wearing-off symptoms before their scheduled infusions. By comparing TEPs from M1 stimulation (90 % RMT) pre- and post-infusion, the study revealed that reduced fatigue-induced modulation of sensorimotor TEPs, present during the wearing-off phase, were restored to normal levels following natalizumab administration (Leodori et al., 2024b). This finding suggests that transient alterations in network function are associated with inflammatory activity, even in the absence of overt clinical relapse. Furthermore, the study demonstrated that the normalization of TMS-EEG responses correlated with improvements in fatigue measures, reinforcing the role of inflammation-driven network dysfunction in MS-related fatigue. These results highlight the potential of TMS-EEG as a biomarker for monitoring treatment effects and detecting subtle network abnormalities that may not be captured by conventional imaging techniques.

### 22.3 Summary and prospectives

Collectively, the findings from these studies illustrate the capacity of TMS-EEG to uncover functional alterations in MS that are not readily observable with conventional imaging. By providing real-time measures of cortical excitability and connectivity, TMS-EEG offers a valuable tool for assessing disease trajectory, predicting treatment response, and identifying potential therapeutic targets. Future research should focus on integrating TMS-EEG with other neuroimaging and biomolecular markers to develop a comprehensive framework for monitoring MS progression. Longitudinal studies will be essential in determining whether TMS-EEG metrics can serve as reliable predictors of long-term disability and treatment efficacy.

## 23 Epilepsy

### 23.1 Introduction

Epilepsies reflect uncontrolled neuronal activity and disturbed E/I balance, and TMS-EEG is a promising tool to significantly contribute to the their pathophysiological understanding, diagnosis and treatment (Silvennoinen et al., 2020).

### 23.2 TMS-EEG findings

Over the last 15 years a number of TMS-EEG studies, summarized in Table 23.1 were conducted in the field of epilepsy, and can be broadly classified into diagnostic, pathophysiological and therapeutic categories.

**Table 23.1:** Synopsis of TMS-EEG studies in epilepsy.

**Authors**	**Sample**	**Region(s) of interest**	**EEG recordings**	**TMS parameters**	**Measurements/ Intervention**	**Key findings**
(Valentin et al., 2008)	15 focal epilepsies,15 HC	7 scalp regions	21 channels SR: not reported	Monophasic SP 100 % RMT	TEPs (early vs. late responses)	(1) 11/15 patients showed late EEG responses vs 0/15 HC (sensitivity: 73 %, specificity: 100 %)(2) Late EEG responses predicted lateralization of epileptogenic region in 8/9 patients with lateralized responses(3) Late TMS-EEG responses in combination with interictal scalp EEG achieved diagnostic rate of 100 %
(Del Felice et al., 2011)	10 JME 12 HC	Left M1	32 channels SR: 5,000 Hz	Biphasic SP 110 % RMT	TEPs measured during wake, sleep deprivation and sleep	(1) Sleep deprivation vs. wake, sleep vs wake: significantly increased P100 and N190 amplitude in JME(2) Sleep deprivation vs. wake: small increases in P100 and N190 amplitude in HC(3) JME vs. HC: increased P100 amplitude during drowsiness and sleep; increased N190 during sleep deprivation and deep sleep (a) left M1: increased N100 amplitude (vs. HC); (b) right M1: increased P180 amplitude (vs. HC)
(Kimiskidis et al., 2013)	10 Generalized & 2 Focal temporal lobe epilepsy (Exp1)3 Focal drug-resistant Frontal lobe epilepsy (Exp2)	Epileptogenic Focus	60 channels SR: 1,450 Hz	Trains of active stimuli (frequency: 0.3–15 Hz, number: 1–10)	ED duration	(1) TMS results in a small but significant reduction of ED duration(2) A circular coil more effective compared to a figure of 8 coil3) TMS was effective even was adjusting for TMS onset latency during the ED
(Julkunen et al., 2013)	7 EPM1 6HC	Left M1	60 channels SR: 1,450 Hz	Biphasic SP 90 % RMT Neuronavigation	TEPs, cortical oscillations (ERSP, ITC)	(1) increased P30 and decreased N100/P180 amplitudes(2) decreased alpha-, beta- and gamma-band power(3) decreased ITC in alpha- and beta-bands
(Shafi et al., 2015)	8 PNH8HC	2 ROI (connected and non– connected)	60 channels SR: 1,450 Hz	Biphasic SP 120 % RMT	TEPs (spatial, temporal) Source localization	(1) PNH (vs. HC): increased late TEPs component amplitudes and increased late GMFP (225–700 ms) in connected regions(2) Source localization in 1 PNH patient: convergence with functionally connected region
(Kimiskidis et al., 2015)	25 GGE11 HC (12 non-responders)	Vertex	60 channels SR: 1,450 Hz	Paired-pulse, lower epileptogenic threshold, circular coil	TMS-induced EDs	(1) TMS-induced EDs occurred intermittently, despite constant stimulation parameters(2) Similarity between TMS-induced EDs and spontaneous EDs.(3) The pre-stimulation EEG contained covert, quasi-stable states of high excitability associated with ED generation
(ter Braack et al., 2016)	13 epilepsy (11 generalized, 2 focal) 18 HC	Bilateral M1	64 channels SR: 2,048 Hz	Biphasic SP 110 % RMT Neuronavigation	TEPs	(1) left M1: increased N100 amplitude (vs. HC)(2) right M1: increased P180 amplitude (vs. HC)
(Kimiskidis et al., 2017)	25 GGE (13 responders to ASMs, 12 non-responders) 11 HC	Vertex	60 channels SR: 1,450 Hz	Biphasic Single/Paired-pulse 100 % lower epileptogenic threshold, ISI: 250 ms)F-8 and circular coil	TEPs Signal energy profiles Data mining procedure Measured at rest, during HV and post-HV	(1) TMS evoked EDs in 2 and abnormal TEP morphology in 4 GGE patients(2) GGE: increased N30a, N100a and N100b amplitudes and increased signal energy in the delta-band(3) Diagnostic accuracy of Index test: 0.92 (discriminating HC vs. GGE); 0.80 (discriminating responders vs. non responders)
(Vlachos et al., 2022)	18 GGE11 HC (12 responders to ASMs, 6 non-responders)	Vertex	60 channels SR: 1,450 Hz	Paired pulse, 100 % MSO, circular coil	Coherence as an EEGconnectivity measure	(1) TMS modulated brain connectivity differently in healthy subjects and GGE patients.(2) In healthy subjects, there was a decrease in connectivity, particularly in the high-frequency bands
(She et al., 2025)	19 children (aged 7–13 years) with SeLECTS, 11 children on ASMs	Bilateral M1, inferior frontal and superior temporal regions	64 channels SR:25,000 Hz	Single pulses were applied to the motor cortex before and after rTMS	Connectivity quantified using wPLI, TEP measurements from six ROIs, IED frequency	(1) Active rTMS decreased wPLI connectivity, especially in superior temporal connections(2) IED frequency decreased after active rTMS

Abbreviations: ASMs, anti-seizure medication; ED, epileptic discharge; EPM1, progressive myoclonus epilepsy type 1; ERSP, event-related spectral perturbation; GGE, genetic generalized epilepsy; GMFP, grand mean field potential; HC, healthy control; IED, interictal epileptic discharge; ISI, interstimulus interval; ITC, inter-trial coherence; JME, juvenile myoclonic epilepsy, M1, primary motor cortex; MSO, maximum stimulator output; PNH, periventricular nodular heterotopia; RMT, resting motor threshold; ROIs, regions of interest; rTMS, repetitive transcranial magnetic stimulation; SeLECTS, self-limited epilepsy with centrotemporal spikes; SP: single pulse TMS; SR, sampling rate; wPLI, weighted phase lag index.

The diagnostic studies explored the potential of TMS-EEG as a diagnostic and predictive biomarker in focal and generalized epilepsy as well as a means for localizing the epileptogenic zone. In *focal epilepsy*, early studies aiming to activate the epileptogenic zone with TMS produced negative results, primarily due to technical reasons. However, the interest in TMS-EEG as an activating method was restarted by Valentin et al who investigated a group of patients with focal seizures and healthy controls by applying single-pulse TMS at various scalp positions (Valentin et al., 2008). The authors described early responses, probably representing TEPs that were not significantly different between the two groups, as well as late TMS-EEG responses. The latter were subclassified into delayed responses, resembling interictal epileptiform discharges (IEDs), and repetitive responses, that corresponded to new rhythms activated by TMS. Late responses were not elicited in healthy controls but appeared in 11/15 patients with epilepsy. It is important to note that late responses predicted correctly the lateralization of the epileptogenic zone in 8/9 cases with lateralized responses. Finally, the late TMS-EEG responses were complementary to the interictal scalp EEG findings and achieved, in combination, diagnostic rates of 100 % leading the authors to suggest that TMS-EEG might be added to standard scalp EEG recordings as an activating method, similar to photic stimulation and hyperventilation.

Shafi et al (2015) performed a multi-modal navigated TMS-EEG study guided by connectivity imaging with resting-state fMRI so as to investigate the cortical excitability profile of patients with periventricular nodular heterotopia (PNH), a neurodevelopmental disorder characterized by structural and functional connectivity abnormalities. In patients with active epilepsy, neocortical areas that were part of a cortico-heterotopic circuit, as evidenced by abnormal connectivity to subcortical heterotopic grey matter, exhibited hyperexcitability with augmented late cortical TMS-EEG responses in comparison to healthy controls (Shafi et al., 2015). The abnormal late responses were location-specific, i.e., not elicited by stimulation of areas that did not partake in the aberrant circuit, and emanated from distributed networks often involving the hemisphere contralateral to the stimulation site. The study demonstrated that resting-state fMRI-guided TMS-EEG may be usefully employed to explore cortical alterations in lesional CNS diseases and, specifically, may represent a novel biomarker of epilepsy in grey matter heterotopias.

In *generalized epilepsy*, numerous studies disclosed distinct alterations in cortical excitability and TEP features compared to healthy controls. In patients with progressive myoclonus epilepsy type 1 (EPM1), characterized by action-activated, stimulus-sensitive myoclonus as well as tonic-clonic seizures, Julkunen et al. (2013) reported enhanced amplitude of the P30 peak and reduced amplitude of the N100-P180 waveform with single-pulse TMS of M1 (Julkunen et al., 2013). The former represents a local excitatory phenomenon whereas N100 is a GABA-B mediated inhibitory response (Premoli et al., 2014a) and, therefore, these findings *in toto* indicate cortical hyperexcitability of M1 in EPM1. The authors also reported decreased oscillatory power in the alpha, beta and gamma band, and reduced alpha and beta inter-trial coherence (ITC) post-TMS, most likely reflecting impaired function of cortical-subcortical circuits in patients with EPM1.

Sleep and epilepsy are intricately related, and sleep deprivation is an established activation procedure for provoking epileptiform discharges (EDs) and seizures in order to increase the diagnostic yield of EEG. Patients with genetic generalized epilepsies (GGEs), such as Juvenile Myoclonic Epilepsy (JME), are particularly sensitive to sleep-deprivation and show pronounced circadian fluctuation of cortical excitability, as evidenced by clustering of seizures early in the morning or at awakening. Del Felice et al. (2011) explored the effect of sleep deprivation in JME patients and healthy controls and reported that late TEP waveforms (at 100 and 180 ms post-TMS) were enhanced in both but the amplitude increase was particularly pronounced in the JME subgroup as well as topographically differentiated, i.e., accentuated in anterior cortex vs. centro-posterior areas in controls (Del Felice et al., 2011). These data further indicate that TMS-EEG is a relevant tool to investigate the modulatory effects of sleep and sleep deprivation on cortical excitability in healthy subjects and patients with epilepsy.

Enhanced late TMS-EEG potentials were also noted in a study of patients with generalized epilepsy, including three subjects with JME, and two patients with focal epilepsy as compared with a group of healthy controls (ter Braack et al., 2016). The authors tested M1 bilaterally with single-pulse TMS and reported increased amplitudes of the N100 waveform after TMS on the left M1, and of the P180 waveform after TMS of the right M1. In both cases, significant changes clustered in centro-parietal areas bilaterally with maximal differences observed in the midline. The authors concluded that these changes were not spurious, i.e., due to acoustic artifacts or secondary to motor threshold differences between the two groups, and may potentially be of use in epilepsy diagnostics or in the evaluation of the efficacy of antiseizure medications.

The diagnostic performance of TMS-EEG in generalized epilepsy was further explored by Kimiskidis et al. (2017) in the context of a phase II diagnostic accuracy study with a dual objective: a) to define a brain stimulation protocol for the assessment of cortical excitability in GGE and b) to apply the optimized protocol in GGE patients and healthy controls (Kimiskidis et al., 2017). The GGE cohort was further dichotomized in responders and non-responders to anti-seizure medications (ASMs). On the basis of an optimal feature subset, TMS-EEG classified subjects in the patient or control group with a maximal leave-one-out cross-validation accuracy of 0.92, achieved at the post-hyperventilation state, and discriminated responders to ASMs from non-responders with a maximal accuracy of 0.80 at the resting state. The authors concluded, in line with the above-mentioned studies, that TMS-EEG holds diagnostic potential and might be used for the stratification of the severity of epilepsy.

The modulation of brain connectivity by TMS may also hold diagnostic potential. Vlachos et al. (2022) investigated patients with GGE vs. healthy controls with a paired-pulse TMS-EEG protocol and explored, in a time-resolved manner, the effect of paired-pulse TMS on local and global brain networks constructed using coherence as an EEG measure (Vlachos et al., 2022). Following TMS, connectivity decreases in controls but is comparatively elevated in GGE patients in a frequency and time-specific manner. The differences are particularly prominent in the gamma-band and occur 1–3 s after the administration of TMS pulses. The authors hypothesized that in healthy subjects an underlying mechanism rapidly resets brain connectivity following the perturbational effect of TMS. In contrast, in GGE patients this mechanism is malfunctioning leading to hyperconnectivity or even the induction of EDs. The study concluded that the differential modulation of brain connectivity by TMS in epilepsy patients vs. controls might form the basis of a diagnostic biomarker in GGE.

From a pathophysiological point of view, TMS-EEG emerged as a highly relevant tool for the investigation of brain dynamics in human epilepsy because it is non-invasive, fully parametrizable and perturbs brain function in a spatio-temporally precise manner. It is therefore a well-suited paradigm in order to investigate the mechanisms underlying the generation and termination of EDs. In GGE, for instance, TMS induces EDs that occur stochastically despite constant stimulation parameters (Kimiskidis et al., 2015). The investigation of the pre-TMS period in the “epileptic” (i.e. leading to EDs) vs “non-epileptic” epochs with multiple measures of univariate time series analysis disclosed the existence of multiple, quasi-stable covert states of excitability, a sub-class of which is associated with the generation of TMS-induced EDs. These high excitability states were found to be located in widespread brain regions, including frontal, central and parietal areas. These observations lend experimental support to theoretical models of ictogenesis such as the “multi-attractor concept” (Kalitzin et al., 2010), and reinforce the body of evidence suggesting that GGE is associated with diffuse, rather than focal, cortical hyperexcitability. In addition, the total duration of TMS-induced EDs in GGE was found to be determined, to a certain extent, by the dynamic state of particular brain areas in the early post-TMS period. These areas were part of the default mode network (DMN) and constitute evidence, complementary to EEG-fMRI studies, about the critical involvement of the DMN in the pathogenesis of absence seizures (Carney and Jackson, 2014). These observations provide some insight into the complex spatio-temporal dynamics of TMS-induced EDs and the pathophysiological mechanisms of human generalized epilepsy.

From a therapeutic perspective, it is well-known that the application of rTMS in the interictal state can result in a modest but statistically significant reduction of IEDs (Jin et al., 2022, Walton et al., 2021, Yang et al., 2023). She et al. (2025) applied inhibitory 1-Hz rTMS to M1 of children with self-limited epilepsy with centrotemporal spikes (SeLECTS) and observed that active stimulation, but not sham rTMS, significantly decreased the frequency of IEDs (She et al., 2025) (see also Section 8). In addition, the authors explored the effect of inhibitory rTMS on brain connectivity assessed with the weighted phase lag index (wPLI), a phase-based measure that avoids pseudo-connectivity due to volume conduction. Active stimulation decreased wPLI connectivity between various areas with maximal reductions observed in superior temporal connections in the stimulated hemisphere. Local and remote TEPs were not affected by rTMS, a fact that emphasizes the importance of employing phase-based measures in the assessment of the connectivity effects of neuromodulation. The observation that inhibitory rTMS reduces aberrant hyperconnectivity and IEDs in patients with SeLECTS raises the possibility that neuromodulation with rTMS may be applied as a therapeutic intervention in this syndrome.

Finally, a limited number of TMS-EEG studies explored *the abortive effect of TMS* on ictal or interictal EDs (Kimiskidis et al., 2013, Rotenberg, 2010). In order to explore the brain network changes that underlie this phenomenon, networks were constructed using a direct causality measure of effective connectivity (PMIME) before, during and after EDs (Kugiumtzis and Kimiskidis, 2015). The occurrence of EDs was associated with a transient but prominent reduction of information flow, as shown by significantly reduced PMIME values, whereas the administration of real, but not sham TMS, terminated the ED prematurely and restored network structure.

### 23.3 Summary and prospectives

TMS-EEG may be considered as a clinically useful but underutilized tool in the field of epilepsy. Converging data suggest that it holds significant diagnostic potential in focal and generalized epilepsies, particularly when combined with advanced methods of data analysis. From a pathophysiological perspective, it is a prime example of an active paradigm able to reveal features of the epileptic brain that are not readily apparent using nonprovocative recordings. Accordingly, it is a highly relevant tool for the exploration of human epilepsy. Finally, recent studies (She et al., 2025) rekindled the interest in the therapeutic potential of TMS-EEG in certain epilepsy syndromes. In addition, the abortive effects of TMS raise the possibility that closed-loop systems that will allow the detection and abortion of TMS-induced EDs, both in patients with epilepsy as well as in the broader therapeutic field of TMS.

At this critical point and in order for further progress to occur, an optimization of the employed methodologies with regard to study design and outcomes (Gefferie et al., 2023), a standardization of the procedures and open data-sharing are required (Belardinelli et al., 2019, Hernandez-Pavon et al., 2023).

## 24 Migraine

### 24.1 Introduction

Migraine is a common neurological disorder characterized by recurrent headaches that are typically accompanied by sensory disturbances, nausea, and sensitivity to light and sound (Headache Classification Committee of the International Headache, 2013, Raggi et al., 2024). The pathophysiology of migraine involves complex neuronal and vascular changes, with altered cortical excitability an indicated underlying feature, presumedly most pronounced for migraine with aura (Goadsby et al., 2017, Tolner et al., 2019). In the acute as well as preventive treatment of migraine, neuromodulation techniques including TMS have gained increasing attention (Barker and Shields, 2017, Shen et al., 2023). The use of TMS for investigating changes in brain excitability has been implemented mostly in the absence of EEG recordings, thus providing indirect measures of cortical excitability. TMS-EMG has been most often employed, for investigation of alterations in motor system responsivity in the context of migraine in general (i.e., interictally, thus in between attacks, in comparison to healthy controls)(Afra et al., 1998, Bohotin et al., 2003, Cosentino et al., 2018, Cosentino et al., 2011, Gunaydin et al., 2006, Khedr et al., 2006, Maertens de Noordhout et al., 1992, Neverdahl et al., 2017, van der Kamp et al., 1996, Werhahn et al., 2000), migraine treatment (Artemenko et al., 2008), menstrual cycle (Yuksel and Topalkara, 2021), sleep restriction (Mykland et al., 2022, Mykland et al., 2023) or in relation to an upcoming attack (Cortese et al., 2017, Cosentino et al., 2014b, Mykland et al., 2023). To assess visual system responsivity changes in the context of migraine, TMS has classically been combined with assessing the threshold for perception of visual phosphenes (Artemenko et al., 2008, Bohotin et al., 2003, Brigo et al., 2013, Gerwig et al., 2012, Gunaydin et al., 2006, Khedr et al., 2006, Mulleners et al., 2002, Young et al., 2008). While many − though not all (Bohotin et al., 2003, Cosentino et al., 2014a, Gunaydin et al., 2006) − of the TMS findings can be considered supportive of the concept that cortical hyperexcitability contributes to migraine susceptibility and attack initiation, without TMS-EEG data there is only indirect support for such a claim.

### 24.2 TMS-EEG findings

Only two studies in people with migraine combined TMS with EEG, both in recent years, allowing direct investigation of cortical neurophysiological changes in people with migraine. The first study, by Bauer et al. (2019), compared the potential of EEG phase clustering in response to TMS, as putative disease biomarker in people with migraine with aura and people with juvenile myoclonic epilepsy. On a single electrode, the phase of TMS-evoked responses aligns between trials shortly after the TMS pulse. Phase clustering 20–60 ms post-stimulus in the 8–70-Hz frequency band is a candidate biomarker for measuring cortical excitability (Saari et al., 2018). No difference was found between migraine and control groups, while phase clustering was enhanced in the epilepsy group (Bauer et al., 2019). The other study, by Helling et al. (2023), explored the potential use of TEPs as marker of cortical excitability in migraine with aura, by comparison to healthy controls. Results revealed altered TEP amplitudes in the migraine group, with decreased N100 peak amplitudes in frontal and occipital regions, and increased P60 peak amplitudes. Complementing the earlier work of Bauer et al., no differences were found in phase clustering over stimuli for any of the studied EEG response frequency bands between migraine and controls. This suggests that while the amplitude of cortical responses is altered in migraine with aura, the consistency of these responses across trials remains similar to healthy individuals.

### 24.3 Summary and prospectives

Future clinical applications of TMS-EEG in migraine research could include broader biomarker development not only for migraine in general, but also in the context of attack initiation and treatment response. To assess whether the reported TEP changes in migraine with aura are specific for this group, direct comparison of TEP features between people suffering from migraine with versus without aura is required. Integration of TMS-EEG with neuroimaging techniques will be powerful for a more comprehensive understanding of migraine pathophysiology. In all TMS-EEG studies, solid sham-control experiments are important to circumvent the concern that the EEG response to TMS-related sound and sensory activation may differ between people with migraine and controls (de Tommaso et al., 2014, Helling et al., 2023). By investigation of TEP changes during different phases of the migraine cycle, TMS-EEG has the potential to provide direct understanding of attack-related changes in cortical excitability, as strongly suggested by various TMS-EMG and TMS-phosphene studies. Similar as in the field of epilepsy, where TMS-EEG features can reflect anti-epileptic medication effects (Bauer et al., 2019, Gefferie et al., 2023), TMS-EEG might be helpful to monitor or predict migraine treatment response. This might be particularly the case for preventive treatments that suppress neuronal activity, such as valproate and topiramate. If TMS-EEG could additionally identify individual features of cortical excitability in people with migraine, such features might be correlated with clinical parameters such as attack frequency, and guide personalized treatment approaches.

## 25 Pain

### 25.1 Introduction

Chronic pain affects 20 % of the general population, and is defined as pain lasting more than three months that is present most of the time. Some types of pain—such as primary headaches and low back pain—are among the most common health disorders worldwide (Global Burden of Diseases Collaborators, 2024) and consume more healthcare resources than cancer or cardiovascular diseases. In contrast to its significant impact, up to 40 % of people with chronic pain continue to experience symptoms despite receiving the best available medical therapies (Hansson et al., 2009). Regardless of the initial cause of pain, it is widely accepted that the development and persistence of chronic pain is at least in part, due to an increased sensitivity of nociceptive processing in the CNS (Treede et al., 2022). Over the past twenty years, research has increasingly focused on understanding how the brain integrates nociceptive signals and identifying the neural correlates of pain perception. Studies using techniques such as functional imaging, resting-state EEG, pain-related evoked potentials, and MEP-based assessments of corticospinal excitability have proliferated. These investigations have helped form frameworks for understanding how acute pain is processed in the brain, how dysfunctional changes in brain network excitability may contribute to its chronification, and how this knowledge could inform therapeutic interventions (Vucic et al., 2023).

### 25.2 TMS-EEG findings

TMS-EEG has allowed for the exploration of cortical excitability within motor areas without contamination by changes in spinal or neuromuscular excitability, as well as for assessments of excitability in pain-relevant extra-motor cortical areas. Despite being in its infancy, interesting insights have already begun to emerge from studies exploring TMS-EEG in the pain field. An initial study using TMS-EEG on M1 during heat pain in healthy subjects found an increased N45 amplitude in fronto-central electrodes that directly correlated with pain ratings (Chowdhury et al., 2023). In a related paradigm, De Martino and colleagues tested changes in the P30-N100 (stimulation of M1) and P30-N45 TEP (stimulation of DLPFC) amplitudes and global-mean field power during acute heat pain (De Martino et al., 2023). Acute pain decreased the peak-to-peak amplitude in M1 and DLPFC compared with non-noxious warm, and the global-mean field power selectively in M1. Participants with the largest reduction in local cortical excitability under acute pain showed a negative correlation between DLPFC and M1 local cortical excitability changes. These findings show differential pain-related local and global cortical excitability changes in motor and non-motor areas at a group level, and interindividual differences in pain-related cortical excitability changes. Such insights could influence trial designs and potentially guide target selection in future personalized approaches to improve the efficacy of rTMS for pain control.

TMS-induced local α-band oscillatory power and α-band phase synchronization in remote parietal–occipital regions in healthy subjects decreases during acute heat pain. The decrease in α-band phase synchronization correlated with individual pain sensitivity (De Martino et al., 2024). Concordant changes in weighted phase-lag index under M1 stimulation suggested that these changes are not due to volume conduction (De Martino et al., 2024). In this same study, stimulating the DLPFC resulted in a confined decrease in β1-band power. These results indicate that changes in TMS-induced oscillatory power during acute pain are specific to the natural frequencies of the stimulated regions, ß for the DLPFC and µ (alpha) for M1.

In patients with neuropathic pain, probing M1 by TMS-EEG led to significantly greater current propagation to the anterior insular cortex, which correlated with the affective components of pain. Moreover, increased current flow from M1 to the perigenual anterior cingulate cortex was associated with lower anxiety levels (Huang et al., 2025).

The posterior insula, DLPFC and M1 are among the most studied rTMS targets in pain (Ciampi de Andrade and García-Larrea, 2023). M1 is the most promising target for relieving pain in people with primary headaches (Moisset et al., 2020), fibromyalgia (Silva et al., 2025), and neuropathic pain (Attal et al., 2021), and is currently recommended by international guidelines for the control of the latter. There is, therefore, significant interest in exploring how rTMS changes TMS-EEG responses and how this information could be used to inform therapy in the future. Under experimental tonic pain induced by capsaicin, 10 Hz rTMS applied to M1 decreased the P100 TEP component, an effect similar to that seen after 3 sessions of accelerated intermittent theta burst stimulation with the same total number of pulses. Furthermore, after 10 Hz rTMS, low-gamma power increased, which correlated with both a reduction in pain ratings and changes in the N100 TEP component (Tan et al., 2024).

In a pioneering study exploring deep targets, the analgesic effects of 10 Hz rTMS of the posterior superior insula on high-concentration capsaicin-induced pain were explored during M1 TMS-EEG recordings (Chowdhury et al., 2024). Tonic pain decreased the amplitude of the N45 TEP, and rTMS of the posterior superior insula restored N45 values toward normal levels while significantly reducing pain. The rTMS-induced reduction in pain numerical rating scale scores after active vs. sham rTMS was correlated with and partially mediated by decreases in the N45 TEP. These findings provide evidence of the analgesic effects of rTMS of the posterior superior insula and suggest that the N45 TEP is a potential marker and mediator of both pain and analgesia (Chowdhury et al., 2024).

Finally, in healthy participants undergoing a cold pressor test (hand immersion in cold water to assess pain tolerance), 10 Hz rTMS applied to the DLPFC decreased the N120 TEP amplitude in the contralateral DLPFC and increased it in the ipsilateral insular cortex. Moreover, there was a strong negative correlation between the N120 changes of these two regions whereby the amplitude changes of this dyad were associated with increased pain threshold (Ye et al., 2022). These findings provide novel evidence on local and distributed neuroplastic changes associated with analgesia induced by high-frequency rTMS of DLPFC.

### 25.3 Summary and prospectives

Overall, these studies demonstrate that TMS-EEG may reveal target-specific changes in local excitability and phase- and power-based cortical connectivity metrics during both experimental acute phasic and tonic pain. Changes in TEPs appear to correlate with perceived pain and “trait” pain sensitivity. Data from people with neuropathic pain suggest that probing M1 may increase current propagation to the anterior insula and perigenual cingulate cortex, which in turn, is correlated with affective components of pain and mood symptoms. The effects of rTMS to M1, DLPFC and the posterior superior insula also led to changes in excitability and connectivity measures, further expanding the possibility of using TEPs to predict and monitor responses to rTMS treatment and personalize therapy (De Martino et al., 2025).

## 26 Summary and outlook

TMS-EEG has dynamically developed since its first demonstrations (Cracco et al., 1989, Ilmoniemi et al., 1997). Up to date (October 20, 2025), more than 650 TMS-EEG papers have been published, and their number is exponentially rising. The method provides unprecedented opportunities to probe brain function by perturbing and evoking brain activity with the TMS pulse and record the brain response with EEG. This goes significantly beyond pure recording methods such as resting-state EEG or functional MRI. It allows, for example, to measure brain excitability and effective connectivity. Clinical utility has been consistently demonstrated for several specific applications, such as prognostication of patients with disorders of consciousness or stroke (Table 26.1). On the other hand, for many other clinical entities, TMS-EEG applications are still at their infancy (see 10, 10.1, 10.2, 10.3, 11, 11.1, 11.2, 11.3, 12, 12.1, 12.2, 12.3, 12.4, 13, 13.1, 13.2, 13.3, 14, 14.1, 14.2, 14.3, 15, 15.1, 15.2, 15.3, 16, 16.1, 16.2, 16.3, 17, 17.1, 17.2, 17.3, 18, 18.1, 18.2, 18.3, 18.4, 18.5, 19, 19.1, 19.2, 19.3, 20, 20.1, 20.2, 20.3, 21, 21.1, 21.2, 21.3, 22, 22.1, 22.2, 22.3, 23, 23.1, 23.2, 23.3, 24, 24.1, 24.2, 24.3, 25, 25.1, 25.2, 25.3, 26), and more evidence is clearly needed to judge clinical utility of potential TMS-EEG biomarkers in these indications. It should be kept in mind that TMS-EEG is time-consuming in terms of montage, data registration and data analysis (all its steps) requiring many resources in a lab or clinical setting. Moreover, TMS-EEG is technically demanding, and standards for avoiding or controlling artefacts, or for EEG preprocessing are still not fully developed or universally accepted. Also, the physiological mechanisms underlying the various TMS-EEG metrics are only incompletely revealed. Further progress along all of these lines will be critical for fully exploiting the potential of TMS-EEG in future research and clinical applications. Big team sciences approaches, such as the Team for TMS-EEG (T4TE) initiative (Bortoletto et al., 2023) will be one important strategy forward to clarify how the currently substantial methodological variation in data recording and analysis across laboratories (Beck et al., 2024b) affects TMS-EEG responses. Physiological mechanisms of TMS-EEG may be further specified, e. g., by pharmacological testing (Darmani and Ziemann, 2019), comparison of different brain states such as awake vs. sleep (Massimini et al., 2005), or comparison of EEG responses to TMS with those to intracranial electrical stimulation (Comolatti et al., 2025). TMS-EEG has a still largely unexploited potential for becoming an important tool in neuroscience to better understand brain physiology, and in clinical neurophysiology to better diagnose patients with neurological and psychiatric disorders. Methodological rigor is essential to realize this potential.

**Table 26.1:** Summary of clinical TMS-EEG studies that have shown consistent findings.

**Clinical entity**	**Sample** **sizes**	**Endpoints**	**Effect sizes** **Significances**	**Translational** **stage**	**References**
Major depressiveDisorder (MDD)	59 MDD 58 HC 30 MDD 30 HC 45 MDD20 HC	Increased TEP-N100 amplitudeleft DLPFC or left superior frontal gyrus	p = 0.04 p = 0.001 Cohen d = 0.8	T1	(Li et al., 2025) (Voineskos et al., 2019) (Dhami et al., 2020)
*Comments:* TEP-N100 amplitude correlated with MDD severity (Li et al., 2025); this finding was not replicated in the other two studies (Voineskos et al., 2019, Dhami et al., 2020); all other studies did not find a significant increase of TEP-N100 amplitude in MDD (Bailey et al., 2023c, Dhami et al., 2021, Han et al., 2023, Li et al., 2024, Li X et al., 2023, Niu et al., 2024).					
Schizophrenia (SCZ)	16 EC-SCZ 16 HC 30 EC-SCZ28 HC	Reduced natural frequency in left frontal cortex	p = 0.018 p = 0.0000002	T2	(Donati et al., 2023) (Donati et al., 2025)
Disorders of consciousness(DOC)	6 UWS 6 MCS 6 EMCS2 LIS (control) 43 UWS 38 MCS 102 HC 40 MCS 40 HC 105 UWS 76 MCS 30 HC	Reduced PCI	p = 8 × 10^−7^ p = 0.0001 p = 0.002 p < 0.0001 (UWS vs MCS)p < < 0.0001 (MCS vs HC) p < 0.0001 Cohen d = 0.40 (UWS vs MCS) Cohen d = 0.42(MCS vs HC)	T3-T4	(Casali et al., 2013) (Casarotto et al., 2016) (Casarotto et al., 2024) (Wang Y et al., 2022)
*Comments:* Longitudinal measurements of PCI correlate with and/or predict clinical improvements in DOC (Comanducci et al., 2024, Wang et al., 2023, Xu et al., 2024).					
Alzheimer’s disease (AD)	65 AD 21 HC 20 AD 17 HC 20 AD17 HC	Increased precuneus TEP 90–120Reduced TMS-evoked signal propagation in DMN	p < 0.05 p = 0.01 p < 0.05	T2 T1	(Casula et al., 2023) (Maiella et al., 2024) (Maiella et al., 2024)
Stroke	10 S/SC 10 SC 28 acute stroke 15 HC 40 acute stroke 15 HC 60 acute stroke 15 HC 10 S/SC 10 SC 40 acute stroke15 HC	Sleep-like slow-wave TEP in ipsilesional cortex, increased LMFP ERSP off-period	10/10 0/10 p = 0.002 p = 0.006 p < 0.05 10/10 0/10 p = 0.005	T3 T2	(Sarasso et al., 2020) (Tscherpel et al., 2020) (Tscherpel et al., 2024) (Harquel et al., 2024) (Sarasso et al., 2020) (Tscherpel et al., 2024)
*Comments:* Decrease in sleep-like slow-wave TEP in ipsilesional cortex and/or off-period correlate with clinical recovery (Tscherpel et al., 2020, Harquel et al., 2024, Tscherpel et al., 2024, Sarasso et al., 2025).					

Translational stages (T) are defined as follows (see also Section 7, Fig. 7.1): T1: Biomarker discovery; T2: internal validation; T3: external validation; T4: clinical validation.

Abbreviations: AD, Alzheimer’s disease; DMN, default mode network; EC-SCZ, early-course schizophrenia; EMCS, emergent minimally consciousness syndrome; HC, healthy controls; LIS, locked-in syndrome; MCS, minimally consciousness syndrome; MDD, major depressive disorder; PCI, perturbational complexity index; S, stroke with isolated subcortical lesion; S/SC, stroke with cortical/subcortical lesion; TEP, TMS-evoked potential; UWS, unresponsive wakefulness syndrome.

## 27 Appendix: Protocols how to measure TMS-EEG responses

### 27.1 Introduction

In this consensus review, we have presented the current state-of-knowledge on using TMS-EEG to probe the pathophysiology of brain disorders. Underpinning the field of TMS-EEG research is the ongoing development of improved protocols for performing these challenging experiments. Protocols for measuring TMS-EEG responses involve three key steps: recording the EEG data, preprocessing the data (this procedure includes cleaning the data), and quantifying the data. Careful preparation and execution at each of these steps is required to achieve TMS-evoked potentials (TEPs) with a high signal-to-noise ratio that are both valid (i.e., are recording neural signals reflecting the underlying mechanism of interest) and reliable (i.e., can be reproduced within the same individual and by different research groups). According to the classic principle “garbage in, garbage out”, the first step is to record good-quality data where artifacts are avoided or minimized during data collection. In cases where artifacts cannot be avoided, careful design of specialized preprocessing pipelines is required (Hernandez-Pavon et al., 2023). Finally, choosing appropriate data quantification metrics is essential to capture the underlying neurophysiological mechanism of interest. In this appendix section, we present different protocols for improving the quality of data collection, preprocessing, and quantification in TMS-EEG research.

### 27.2 Online approaches

TMS-EEG is a powerful tool for investigating cortical reactivity and connectivity, as TMS parameters are fully under experimental control, including the target site, stimulation intensity (SI), and orientation of the induced electric field (E-field). However, it is difficult to predict to what extent a certain combination of TMS parameters will actually activate cortical neurons. Nonetheless, a direct cortical perturbation is expected to produce a local activation that progressively spreads towards distant sites according to functional and anatomical connections (Bortoletto et al., 2015, Ilmoniemi et al., 1997). Thus, in principle, looking at EEG signals in real time allows controlling for the actual TMS impact on the cortex (Casarotto et al., 2013, Casarotto et al., 2022, Fecchio et al., 2025, Hernandez-Pavon et al., 2023). This approach is similar to what ultrasound operators routinely apply in clinics when measuring the anatomy of a target organ. Even when applying TMS to M1, stimulation parameters are usually adjusted until a MEP is properly recorded from the targeted peripheral muscle. The application of this approach to TMS-EEG measurements would enable real-time adjustment of TMS parameters, thereby minimizing the contribution of artifacts and confounding factors and maximizing the signal-to-noise ratio of genuine brain responses to direct cortical perturbation.

### 27.3 EEG setup and preparation

EEG recorded during TMS requires specific software and additional experimental steps that differ from those used in traditional EEG. Here we describe some aspects of the EEG setup and preparation for TMS-EEG.

1. Ensure that you use a TMS-compatible EEG system, and that the system can record at high sampling frequencies (for instance, 5,000 Hz or higher) with a wide hardware filtering bandwidth (e.g., DC − 1 kHz or more) and with sufficient dynamic range to avoid saturation during the TMS-pulse artifact.
2. Use an EEG cap with a sufficient number of electrodes to record the spatial properties of the TMS-evoked response. Most studies use at least 60 electrodes, although as few as 32 electrodes have also been used. Depending on your study, you can use passive or active electrodes. For more information on the pros and cons of using active or passive electrodes and theoretical arguments regarding electrode numbers, see Hernandez-Pavon et al. (2023).

There are various online approaches that can be employed to minimize artifacts and enhance data quality. These approaches vary across laboratories, but here we summarize some steps that can be helpful during data recording.

3. Ensure you have all the necessary EEG preparation materials ready. It is important that the abrasive and conductive gels do not contain metallic particles, as they could interact with the strong magnetic field and induce, for instance, decay artifacts.
4. Prior to placing the EEG cap, use an isopropyl alcohol pad to clean the forehead, the skin around the eyes, and the areas where the reference and ground electrodes will be positioned. This will help decrease impedance values.
5. For the reference and ground electrodes, gently abrade the skin with sandpaper or abrasive gel after (or before) the area has been prepped with an alcohol pad.
6. Make sure the EEG cap tightly fits the participant’s head. It is advised to measure the head size before the recording. When using the 10–20 system to report electrode locations, the Cz electrode should be positioned exactly halfway between the nasion and inion, as well as halfway between the left and right preauricular points. The central line should be straight and aligned with the midline.
7. To minimize interference and avoid high-amplitude, TMS-locked artifacts that can affect all channels, it is advisable to position the reference electrode at a significant distance from the TMS coil (Hernandez-Pavon et al., 2023). For example, when the TMS pulse is delivered to the left hemisphere, the reference electrode can be placed on the right mastoid and the ground electrode on the right cheekbone. Similarly, if the EEG system uses the right-driven leg circuit to cancel out the common mode noise from the active EEG channels, also the ground electrode should be placed sufficiently far away from the TMS coil.
8. Prepare EEG electrode contacts as in any EEG experiment. This can be done using abrasive electrode paste and/or conductive gel.
9. For passive electrodes, keep the impedance values ≤ 5 kΩ where possible.

### 27.4 TMS set-up and preparation

1. Jittering of the interstimulus interval is important to prevent habituation or expectation effects. Furthermore, when measuring TEPs, the jitter helps to cancel out line noise and pronounced oscillatory resting-state activity in the average response. Therefore, typically, the TMS systems used allow jittering around 2–2.4 s as a minimum. However, recent evidence suggests that using a fixed interval could help suppress the sensory-evoked response through habituation (Ross et al., 2022).
2. To prevent the possible occurrence of artifacts on the EEG, the recharging of TMS capacitors should be delayed not to overlap either with the EEG response of interest or with a putative baseline period. Therefore, the option to control the TMS recharge delay in the TMS stimulator is important. The recharging timing should be adjustable or set outside the temporal window of interest, as it may introduce an artifact. Specifically, the recharge delay should not overlap with the relevant post-TMS signal; one option is to set it at approximately 900 to 1000 ms. The option to delay the capacitor recharge is available in most commercial systems but can also be controlled remotely using the open-source MAGIC toolbox (Habibollahi Saatlou et al., 2018).
3. The target site should be ideally identified on individual structural MRI, taking into account that the induced E-field is maximum on the gyral crowns.
4. The SI should be initially set based on prior information about the scalp-to-cortex distance and subsequently adjusted based on real-time EEG readouts. A coarse estimation of the induced E-field intensity on the selected target can be helpful, considering that an intensity of 50–70 V/m is usually sufficient to activate cortical neurons in healthy subjects (Numssen et al., 2024).
5. When stimulating M1, the motor threshold should be measured to evaluate whether a sub-threshold intensity is desired to prevent proprioceptive feedback from the activation of peripheral muscles. Muscle responses can be recorded using EMG electrodes. Before placing the EMG electrodes, gently abrade the skin with sandpaper or abrasive gel after the area has been prepped with an alcohol pad. Depending on the muscle of interest, for instance, place the active electrode on the muscle belly, the reference electrode over the muscle tendon, and the ground electrode on the dorsum of the hand.
6. Initially, the operator should be enabled to manually deliver a few single pulses to evaluate the possible occurrence of artifacts in single-trial EEG responses. Then, when stimulation parameters (coil position, angle, orientation, and stimulation intensity) are set, a sequence of pulses with a jittered intertrial interval could be automatically delivered (see step 1).

### 27.5 Neuronavigation

Neuronavigation is strongly recommended to maintain the position, orientation, and angulation of the TMS coil consistently throughout a session and across multiple visits (Lioumis and Rosanova, 2022, Ruohonen and Karhu, 2010). Neuronavigation increases the precision of target selection overall and is especially recommended in the presence of brain lesions to avoid stimulating over necrotic tissue, which does not elicit measurable neural responses (Gosseries et al., 2015).

1. If neuronavigation is available, perform all associated procedures, such as fixing the head tracker and registering landmarks.

### 27.6 Auditory noise masking

1. Ensure that participants are provided with appropriate hearing protection and proceed with the navigation to locate the hot spot.

2. If masking noise is used, the SI should be obtained while the noise masking is being played.

3. When using noise masking to maximally reduce the contribution of unspecific auditory-evoked potentials, the operator should first rely on the participant’s report about residual TMS click perception; then, the absence of auditory components with characteristic late latencies and polarity (N100-P200) and central topography should be verified by looking at average EEG responses to sham TMS in real time.

### 27.7 Assessing the quality of TMS-evoked EEG responses

1 A display of TMS-evoked potentials is crucial for evaluating the quality of EEG responses in real-time. Several open-source software solutions (Casarotto et al., 2022, Parmigiani et al., 2025) as well as commercial solutions are currently available.

2 Visual inspection of single-trial data allows detecting large artifacts of muscular origin that have a characteristic time course (<10 ms) and spatial topography (over lateral electrodes) and mainly occur when stimulating lateral scalp sites (Hernandez-Pavon et al., 2012, Korhonen et al., 2011, Mutanen et al., 2013a).

3 Looking at average responses displayed in average reference after excluding artifact-contaminated channels allows to evaluate the amplitude and time course of TMS-evoked potentials. Brain responses to direct cortical stimulation elicits oscillatory components that are larger than oscillations in the baseline period and that are expected to be (i) larger in the channels close to the stimulation site compared to distant ones; (ii) larger in the first few tens of ms after the pulse compared to late latencies; (iii) asymmetric between homologous channels of the two hemispheres, with larger amplitudes on the stimulated brain side. These characteristics can be evaluated already after averaging a few tens of trials and should inform the decision about whether to keep the stimulation parameters or further adjust them to increase the signal-to-noise ratio.

### 27.8 Offline approaches

While the gold standard in TMS-EEG research is to collect data as free from artifacts as possible (i.e., online approaches for artifact minimization), the reality is that some degree of data contamination by artifacts is inevitable (see Section 2). This is true of all EEG experiments. As a result, it is common practice to use digital signal processing methods to further improve the signal-to-noise ratio by removing or suppressing noise from recorded EEG data (i.e., offline approaches for artifact minimization) (Delorme, 2023). EEG cleaning pipelines often involve multiple steps, which can include removal of data contaminated by artifacts (either removal of entire channels or sections of the data), temporal filters to suppress frequencies in the signal not of interest or contaminated by artifact, and/or spatial filters derived from signal separation methods (e.g., independent component analysis “ICA” and principal component analysis “PCA”) to remove or to suppress signals with certain spatial profiles (Hernandez-Pavon et al., 2022). The artifact profile of TMS-EEG data presents some unique challenges for designing processing pipelines, mainly due to the size (often several orders of magnitude larger than neural data) and frequency of artifacts introduced by TMS. Paradoxically, some TMS-related artifacts can interact with commonly used cleaning methods, resulting in the introduction of new cleaning-related artifacts (Ilmoniemi et al., 2015, Metsomaa et al., 2024, Rogasch et al., 2017). Furthermore, some methods can remove the signal of interest. As a result, common EEG preprocessing pipelines are often inappropriate for TMS-EEG data. Care must be taken to design suitable preprocessing pipelines that minimize noise without introducing new artifacts or compromising the neural signal-of-interest (Hernandez-Pavon et al., 2022, Hernandez-Pavon et al., 2012, Mutanen et al., 2022, Rogasch et al., 2022). The origin of TMS-EEG artifacts and the specific offline methods developed to remove/minimize these artifacts are covered in detail earlier in this review (see also 3, 3.1, 3.2, 3.3, 3.4, 3.5, 4). In this section, we instead focus on the more practical question of how to design preprocessing pipelines for TMS-EEG data, paying particular attention to the steps included in these pipelines and the order in which they are performed. We then compare some commonly used pipelines for TMS-EEG preprocessing and ask the question: which pipeline shall I use?

### 27.9 Design a pipeline appropriate for the artifacts present in the data

First, it is important to recognize that the artifact profile (Section 2) can vary substantially between different TMS-EEG experimental arrangements. This could result from differing aims of studies (e.g., a study requiring a predetermined stimulation site vs. one that can optimize coil position to maximize TEP size and minimize artifacts) or the availability of research equipment between laboratories (e.g., older stimulators where the option to delay recharge artifacts is unavailable, an EEG amplifier with a sample-and-hold circuit). As a result, the requirements of a preprocessing pipeline might differ substantially between different TMS-EEG data sets. For example, if TMS muscle artifacts have been avoided during data collection, then it is not necessary to use aggressive cleaning methods such as ICA or signal space projection with source-informed reconstruction (SSP-SIR) to remove or suppress artifacts in early time periods, which can introduce an added risk of overcorrecting the data. For example, when stimulating midline scalp sites and adjusting TMS parameters based on real-time EEG readout, the contribution of muscle artifacts can be negligible or at least significantly minimized. In this condition, small residual components of muscular origin can be removed by low-pass filtering or isolated as independent components with a limited contribution to the overall data variance, characterized by a specific time–frequency profile and a spatially confined scalp topography. Conversely, stimulation of lateral sites often engenders the unwanted activation of scalp muscles, which can be minimized but not completely avoided by adjusting TMS parameters according to the EEG signal. Therefore, more sophisticated data preprocessing procedures should be applied to disentangle brain responses from confounding muscle responses. The key principle here is to design preprocessing pipelines that are appropriate for the artifact profile of the data.

### 27.10 Removing too much data can be detrimental to the signal-to-noise ratio

A simple approach for increasing the signal-to-noise ratio is to remove data contaminated by noise. This could include removing one or several EEG channels which show persistent noise across the recording or selectively removing trials with excessive noise contamination (e.g., due to increased muscle activity, eye blinks, etc.). On face value, this appears to be a robust choice. However, removing noisy data comes at a cost, as neural signals are also often removed. For example, the TMS coil makes contact with the electrodes near the stimulation site, introducing noise. However, these electrodes also contain the signal-of-interest (e.g., the neural activity from the cortex underneath the coil). Removing too many channels from around the site of TMS, therefore, removes the signal-of-interest. Furthermore, the signal-to-noise ratio of TEPs is also dependent on the number of trials (the ratio is calculated by multiplying the single-trial signal-to-noise ratio by the square root of the number of trials). The more trials that are removed, the lower the theoretical maximum of the signal-to-noise ratio (Hernandez-Pavon et al., 2023). Note that this relationship is not linear. Removing 30 trials from a total of 300 trials would lower the maximum signal-to-noise ratio by about 5 %, whereas removing 30 trials from a total of 100 trials would decrease the maximum ratio by about 16 %. Therefore, removing data represents a careful balance between removing noise while minimizing the removal of signal, and maintaining sufficient data. As a rule of thumb, about 100 clean trials are recommended for TMS-EEG recordings, so where possible, collect enough data that at least this number of trials will be left after cleaning (Hernandez-Pavon et al., 2023).

### 27.11 Avoid using temporal filters over large steps in the data

Perhaps the most important principle in designing TMS-EEG preprocessing pipelines is to be careful with using temporal filters (i.e., filters across time). Temporal filters are commonly used in EEG time series to suppress frequencies in the data that are not of interest, including low frequency signals (high-pass filters), high frequency signals (low-pass filters), both high and low frequency signals at the same time (band-pass filters), or signals at a specific frequency (e.g., line noise; band-stop or notch filters) (de Cheveigné and Nelken, 2019, Hernandez-Pavon et al., 2022). Low-pass filters are also used before downsampling to prevent aliasing artifacts (note that this form of filter is also used during data collection). Temporal filters are applied to individual channels and are often acausal or zero-phase, meaning that they are run both forward and backward across the EEG time series to prevent latency shifts in the signal. Importantly, temporal filters are not perfect and can introduce unwanted distortions to the signal under certain circumstances. For example, filtering over spikes or steps in the data can result in ‘ringing’ artifacts (e.g., the introduction of spurious oscillations) or ‘decay’ artifacts (e.g., a slow return to a baseline level) (de Cheveigné and Nelken, 2019). Due to the acausal design of most filters, these effects can be observed after, but also *before,* the spike/step event. Furthermore, depending on the size of the event and the frequency characteristics of the filter, spurious oscillations can exhibit temporal features similar to those of TMS-evoked potentials.

Large amplitude spikes (e.g., TMS pulse artifact, TMS muscle artifact, TMS recharge artifact) and steps (e.g., TMS discharge artifact) are common in raw TMS-EEG data. Applying temporal filters over these TMS-related artifacts can introduce both ringing and decay artifacts before and after the artifact events (Rogasch et al., 2017). Therefore, it is important to remove or suppress large amplitude TMS artifacts prior to temporal filtering (including antialiasing filters associated with downsampling). A common approach to mitigate TMS pulse and muscle artifacts is to remove data around the pulse (e.g., −2 to 5, 10 or 15 ms). However, if discharge artifacts or other offsets are present after the TMS pulse, this approach can introduce additional steps in the data, which can also lead to filtering artifacts. As a result, it is important to ‘smooth’ the removed data using interpolation, thereby minimizing steps in the data. Successful approaches for preventing filtering artifacts include interpolation using a cubic function (Rogasch et al., 2017), and blending forward and backward time series derived using autoregressive interpolation (Cline et al., 2021). Modified high-pass filters using autoregressive interpolation have also been suggested to minimize distortions resulting from discharge artifacts in the pre- and post-TMS window (Cline et al., 2021). Of note, filtering artifacts can also be introduced at the start and end of trials in epoched data. It is recommended to extract epochs with a large enough time range so that such artifacts won’t interfere with the signal of interest (e.g., usually at least −1000 to 1000 ms around the TMS pulse).

### 27.12 Use spatial filters (e.g., signal separation approaches) with caution

Spatial filters are another type of filtering used to improve the signal-to-noise ratio in EEG data. As opposed to operating on single channels like temporal filters, spatial filters operate on multichannel EEG data. Spatial filtering is based on the fact that signals of different origins are often detectable by multiple channels, each with a distinct topography. For example, neural activity from a given region will be detectable by a large number of channels, usually forming either a radial or tangential dipole due to volume conduction (Delorme et al., 2012). In contrast, eye blinks and eye movements result in signals in channels close to the eyes, whereas craniofacial muscle activity results in signals in lateral channels (Jung et al., 2000). One method of obtaining spatial filters is to use signal separation approaches such as ICA (Makeig et al., 1995). ICA attempts to separate out the data into a series of components (i.e., spatial weightings) based on the underlying temporal statistics of the data. These components often correspond to the topographies of neural signals and various types of artifacts (blinks and eye movement, muscle activity, electrode movement, cardiac activity). Multiplying the spatial weight of a given component by the data reveals the time series explained by that component. Conversely, multiplying the data by all components except for those representing artifacts reconstructs the EEG data without the artifacts. Various other signal separation approaches have been developed for generating spatial filters to identify and minimize artifacts in TMS-EEG data, including SSP, SSP-SIR (Mutanen et al., 2016), source-estimate-utilizing noise-discarding (SOUND) algorithm (Mutanen et al., 2018), PCA, and several variants of ICA (Hernandez-Pavon et al., 2022). However, TMS-EEG data often violates the assumptions underlying signal separation approaches. For example, ICA assumes temporal independence between underlying sources. The TMS pulse weakens this assumption as both artifacts (e.g., TMS-evoked muscle artifacts) and neural signals are time-locked to the pulse (Atti et al., 2024, Metsomaa et al., 2014, Mutanen et al., 2024). As a result, there is a considerable risk that the components will represent a mix of artifact and neural activity due to the inaccurate decomposition of the data. Removing these components will result in overcorrection as the signal of interest is removed alongside the artifact. Conversely, not removing the component leaves artifacts in the data. In contrast, SSP-SIR is not dependent on temporal correlations in the data but does require that the neural signal and artifact signal have differing topographies − an assumption which is weakened when the stimulation site is close to craniofacial muscles (Mutanen et al., 2024). Therefore, using signal separation approaches requires extreme caution in TMS-EEG cleaning, especially when removing artifacts time-locked to the TMS pulse like TMS-evoked muscle artifacts. In contrast, ICA can be used more confidently for suppressing eye blinks/movements and ongoing muscle activity not time-locked to the TMS pulse.

### 27.13 Manual vs. Automated pipelines

Another factor which possibly adds variability to the outcomes of TMS-EEG preprocessing pipelines is the manual selection of which data and components to remove. Even expert EEG analysts show at best moderate agreement when selecting which channels, trials, and components to remove from EEG data (Delorme and Martin, 2021, Pion-Tonachini et al., 2019). Furthermore, manual labeling of EEG data is very time consuming. An alternative approach is to use automated pipelines which use rules determined either through heuristic feature selection or machine learning-based approaches to select channels/trials/components for removal. Automated pipelines for preprocessing resting- and task-based EEG data have gradually improved and now show performance levels rivalling EEG experts (Bailey et al., 2023a, Bailey et al., 2023b, Delorme and Martin, 2021, Pion-Tonachini et al., 2019). Several automated TMS-EEG pipelines have been developed including ARTIST (Wu et al., 2018) and AARATEP (Cline et al., 2021), as well as heuristic rules for classifying TMS-related artifact components in ICA (Korhonen et al., 2011, Metsomaa et al., 2024). While automated pipelines improve consistency and save time, caution is still required. Careful validation against expert analysts is required, and there is always the risk that automated pipelines will perform in unexpected ways under certain circumstances, e.g., if there is an uncommon artifact present in the data. For example, ARTIST showed good performance against expert analysts in initial comparisons (Wu et al., 2018), but showed bigger differences compared to manual pipelines in subsequent comparisons (Bertazzoli et al., 2021) and was slightly less accurate at recovering simulated ground-truth data (Brancaccio et al., 2024). The continued development and validation of automated TMS-EEG pipelines is required and encouraged. However, users are advised to carefully assess the outcomes of any automated pipeline.

### 27.14 A comparison of different TMS-EEG preprocessing pipelines

In Table 27.1, we compare a number of different TMS-EEG preprocessing pipelines used in the literature, including the type of data they were designed to clean and whether they are manual or automated. Note that this is not a complete list of all the pipelines used in the field, nor does it provide a recommendation on which pipeline is most appropriate for certain circumstances. Instead, we wish to highlight the considerable variability in preprocessing pipelines currently used across the TMS-EEG field. For example, the ‘sample-and-hold’ pipeline was designed for a TMS-EEG system where the pulse artifact was not recorded and the TMS muscle artifact was avoided during data collection (Fecchio et al., 2017). The ‘immediate TEP’ pipeline was designed for recording TEPs within 8 ms of the TMS pulse (Beck et al., 2024a). Data were recorded at very high sampling rates (50,000 Hz) to reduce the recovery latency of the pulse artifact and TMS muscle artifacts were avoided online. The ‘two step ICA’ (Rogasch et al., 2014) and ‘SOUND, SSP-SIR’ (Mutanen et al., 2022) pipelines were designed for data containing TMS muscle artifacts, as were the ‘ARTIST’ (Wu et al., 2018) and ‘AARATEP’ (Cline et al., 2021) pipelines. The channel, trial, and component selection were fully automated in the latter two pipelines. Note the variability in the cleaning steps and order of steps between pipelines. Understanding how to best apply preprocessing pipelines to optimize signal-to-noise ratios in TMS-EEG data is an active area of research, without a clear consensus. The continued development and validation of preprocessing pipelines in concert with data collection methods is an important consideration for clinical TMS-EEG studies.

**Table 27.1:** Comparison of different preprocessing pipelines for TMS-EEG.

	**Sample-** **and-hold**	**Immediate TEP**	**Two step ICA**	**SOUND, SSP-SIR**	**ARTIST**	**AARATEP**
References	(Fecchio et al., 2017)	(Beck et al., 2024a)	(Rogasch et al., 2014)	(Mutanen et al., 2024)	(Wu et al., 2018)	(Cline et al., 2021)
Manual vs. automated	Manual	Manual	Manual	Manual	Automated	Automated
Designed for TMS muscle activity?	No	No	Yes	Yes	Yes	Yes
Step 1	Channel rejection	Epoch (−500 to 500 ms)	Channel rejection	Epoch (−1500 to 1500 ms)	Remove DC drift	Epoch (−1000 to 1500 ms)
Step 2	Trial rejection	Baseline correction (−110 to −10 ms)	Epoch (−1000 to 1000 ms)	Baseline correction (−500 to −5 ms)	Interpolate TMS pulse	Interpolate TMS pulse
Step 3	Band-pass filter (1–80 Hz)	Band-pass filter (0.1–2000 Hz)	Demean (−1000 to 1000 ms)	Interpolate TMS pulse	Down- Sample (1000 Hz)	Down- Sample (1000 Hz)
Step 4	Down- Sample (725 Hz)	Trial rejection*	Interpolate TMS pulse	Channel rejection	ICA-1 (TMS muscle, TMS discharge)	Baseline correction (n.s.)
Step 5	Epoch (−600 to 600 ms)		Down- sample (1000 Hz)	Trial rejection	High-pass (1 Hz) low-pass (100 Hz), band-stop (60 Hz) filters	Modified high-pass filter (1 Hz)
Step 6	Interpolate channels		Trial rejection	Robust detrending	Epoch (−500 to 1500 ms)	Channel rejection
Step 7	Re-reference to average		ICA-1^(TMS muscle, TMS discharge)	ICA-1 (blinks, eye movement)	Trial rejection	Interpolate channels
Step 8	Baseline correction (n.s.)		Band-pass (1–100 Hz), band-stop (48–52 Hz) filters	Baseline correction (−500 to −5 ms)	Channel rejection	ICA-1^β^ (blinks, eye movement)
Step 9	ICA-1 (blinks, eye movement, muscle)		ICA-2^(blinks, eye movement, muscle)	SOUND	Interpolate channels	SOUND
Step 10			Interpolate channels	Interpolate channels^#^	ICA-2 (blinks, eye movement, muscle, heart)	Fit and remove decay
Step 11			Re-reference to average	Re-reference to average^#^	Re-reference to average	Interpolate TMS artifact
Step 12				SSP-SIR	Baseline correction (−300 to −100 ms)	Band-stop filter (58–62 Hz)
Step 13				Low-pass filter (80 Hz)		ICA-2(muscle, TMS muscle, TMS discharge)
Step 14						Low-pass filter (200 Hz)
Step 15						Re-reference to average

Abbreviations: n.s. = not specified; * Trial rejection based on trials with prestimulus hand muscle activity.^Interpolated data around the TMS pulse were replaced with zeros prior to ICA, and then re-interpolated post ICA. # Channel interpolation and re-referencing were performed as part of the SOUND algorithm. β An additional ICA step was added in version 2 of the AARATEP pipeline.

### 27.15 Example of a pipeline for analyzing single-pulse TMS-EEG data

In general, recommending a single EEG cleaning pipeline suitable for all kinds of TMS-EEG data is difficult and possibly suboptimal. As discussed, and reviewed in this work, there are pros and cons to using one pipeline over another. In addition, the TMS-EEG recordings are performed with different devices and with different procedures, which in some cases require additional preprocessing steps to be implemented. We have previously described several recommendations for data collection and analysis (Hernandez-Pavon et al., 2023). Moreover, a few pipeline examples have been previously published (Atluri et al., 2016, Bertazzoli et al., 2021, Hernandez-Pavon et al., 2022, Rogasch et al., 2022, Rogasch et al., 2017, Wu et al., 2018).

In Table 27.2, we summarize a few important steps for single-pulse TMS-EEG that are generally valid across a wide range of studies.

**Table 27.2:** Example of a pipeline for analyzing single-pulse TMS-EEG data. Note that this pipeline is only a suggestion, and fewer or additional steps may be performed depending on the TMS-EEG data. In addition, the order of some steps may change depending on the data.

**Step**	**Procedure**	**Note**
1	Make sure to perform an appropriate TMS-EEG preparation, e.g., reduce the impedances (<5 kΩ), positioning of reference and ground electrodes far from the stimulation target, and proper selection of the EEG amplifier settings (hardware filtering bandwidth, sampling rate (≥ 5 kHz), amplitude resolution).	
2	Remove the TMS-pulse artifact.	For a specific hardware configuration (TMS model, amplifier settings, EEG electrodes), the duration of the pulse artifact is fixed. Thus, the approach to remove the pulse artifact should be kept constant across stimulation sites and subjects.
3	Interpolate TMS pulse.	It is important to replace the time window affected by the pulse artifact with a smooth signal that does not affect subsequent filtering.
4	High-pass filtering	Before removing the TMS artifact and any other TMS-evoked artifacts, be cautious of conventional filtering, which may Induce ringing signals. One suggestion is that only after removing the TMS pulse artifact, one applies a high-pass filter to remove DC and very low-frequency oscillations (cutoff >= 0.5 Hz) from continuous data. However, some laboratories do not apply high-pass filtering.
5	Epoch around the TMS pulse.	Ensure the epoch length is sufficient to ensure potential edge artifacts caused by filtering do not impact the TEP. −1000 to 1000 ms is often sufficient but should be checked and may differ if time–frequency analysis is used.
6	Baseline correction, for instance, from −300 to −10 ms.	
7	Remove bad channels and trials.	For example, by visual inspection. A minimum number of good trials (e.g., 80) and channels (>90 %) should be considered for further analysis.
8	Downsample the data (optional)	This step is optional and not really needed for data analysis. However, the data are often recorded at sampling rates higher than required for analysis (e.g., ≥ 5 kHz). Downsampling the data between 500–1000 Hz is often sufficient for analysis and reduces the size of data on disk.
9	Re-reference to the average reference	Although ICA decomposition is mathematically comparable when applied to both common reference and average reference data, visual inspection of components is easier when ICA is applied to data in average reference because topography is more interpretable.
10	Remove muscle artifacts or any other types of artifacts (for instance, ocular artifacts) using your preferred method, for instance, ICA, SSP, PCA, SOUND.	Be aware of the assumptions of the methods when using them. For example, check the data matrix rank before applying ICA, since interpolated channels do not contribute to the max number of independent components that can be estimated (Hernandez-Pavon et al., 2022).
11	Interpolate bad channels	This step can be performed before or after ICA, either approach is not more correct than the other. However, if the number of bad EEG channels to be interpolated is small (< 5 %) results are comparable.
12	Filtering.	Filtering is safest to use at the end, after the short-lasting artifact peaks have been removed.

We wish to emphasize that in the TMS-EEG community, *there is no consensus on an optimal pipeline for preprocessing TMS-EEG data*. Therefore, the above steps are only a suggestion, and there is no consensus on the optimal order steps 2 to 12 are performed when preprocessing TMS-EEG data. Overall, it is good practice to check the intermediate results of each data preprocessing step before running a full pipeline and make sure that the next step in the analysis is not negatively affected by the previous steps (see, for instance, Section 6.2 from (Hernandez-Pavon et al., 2023)). When unexpected effects are observed, it is worth considering whether there is a conflict between the data's properties and the method's assumptions.

### 27.16 Which pipeline shall i use?

Given the above information, which pipeline shall I use? This is a very difficult question to answer. Comparisons of preprocessing pipelines have demonstrated that the choice matters − the resulting TEPs differ when different preprocessing pipelines are run on the same data (Bertazzoli et al., 2021, Rogasch et al., 2022). However, determining which pipeline results in the most accurate representation of the underlying neural signal is challenging to determine, as the ‘ground truth’ is typically unknown. Recent studies have used simulated data to generate a known ground truth to help answer this question (Atti et al., 2024, Brancaccio et al., 2024, Hernandez-Pavon et al., 2012, Mutanen et al., 2024), though this research is in an early stage. One simple approach is to regularly visualize the data during preprocessing. If a preprocessing step, such as temporal filtering, introduces offsets or oscillations to the data (which is often obvious before the TMS pulse), then there is likely a problem. If a preprocessing step like ICA does not remove an obvious artifact, or if there is only a small TEP remaining following preprocessing, then it is likely that the data are under- or over-cleaned. Another alternative is to preprocess the data using multiple pipelines and assess whether the study results are altered. If different pipelines return the same result, then one can be more confident that the pipeline is not the determining factor in a group difference. The TESA toolbox (Mutanen et al., 2020, Rogasch et al., 2017) offers the possibility of designing multiple different preprocessing pipelines, which can be easily implemented on the same dataset. Of note, the choice of pipeline likely matters less if artifacts have been adequately avoided during data collection. For instance, removing TMS-evoked muscle activity offline is very difficult to do accurately without altering the underlying neural activity. Where possible, stick to the gold standard of minimizing artifacts as much as possible during data collection.

### 27.17 Quantification of TMS-EEG responses

Once the data have been collected and preprocessed, the final step is to quantify TMS-EEG responses. The TMS-EEG responses can be analyzed in the temporal and frequency domains as well as at the scalp level or at the source level. There are several ways to do that, and the choice of the measure of interest is strictly linked to the study hypotheses, as each measure reflects a specific neurophysiological process (see Section 5).

#### 27.17.1 Spectral measures or oscillations

It is possible to measure the entire oscillatory response triggered by TMS, which includes both phase-locked and non-phase-locked components. In this case, time–frequency representations (TFR) are computed at the single-trial level and then averaged. This analysis accounts for the non-stationarity of the TMS-related response by dividing the signal into short-time segments and computing the frequency content within each segment. This method enables the measurement of how the frequency content evolves over time.

There are critical parameters that affect the values of the TFR, including the time–frequency method, the window size, and the normalization over baseline. A widely used time–frequency method is the short-time Fourier transform (Gabor, 1946), in which frequencies are calculated over fixed-length time windows. This imposes a rigid trade-off between time and frequency resolution: a good resolution can be achieved in one domain, but typically not both at the same time. Adaptive methods such as wavelet convolution (Grossmann and Morlet, 1984) have been developed to overcome this limitation. These methods allow the time and frequency resolution to vary across frequencies, offering better frequency resolution at lower frequencies and better temporal precision at higher frequencies. Overall, depending on the particular method, different aspects may be emphasized in the signal, and currently there is no established objective criterion to decide which one is best (Barzan et al., 2022, Moca et al., 2021).

Several indices can be measured from the TFR. First, it is possible to obtain the power of the TFR, also called TMS-related spectral perturbation (TRSP). TRSP is the extension of event-related spectral perturbation (Makeig, 1993) to TMS, and it measures the average dynamic change in amplitude across all the bands of the EEG frequency spectrum after TMS. Studies measuring TRSP have shown that cortical areas resonate to TMS perturbation with a specific dominant frequency, named the natural frequency (D'Ambrosio et al., 2022, Rosanova et al., 2009), and that there is a hierarchical gradient directed from slower frequencies caudally to faster frequencies rostrally (Ferrarelli et al., 2012, Rosanova et al., 2009). Moreover, TFR can be exploited to obtain several other indices, for instance, inter-trial coherence, phase locking values, and phase-lag index, which measure phase across trials at single channels (Pellicciari et al., 2017, Tremblay et al., 2019).

#### 27.17.2 Peak amplitude, peak latency, and mean amplitude (ROI, cluster-based, peak)

In addition to spectral measures or oscillations, it is possible to isolate phase-locked responses and obtain TEPs. Conceptually, TEPs are equivalent to event-related potentials (ERPs) following TMS. To study TEPs, the signal can be averaged across multiple trials. The resulting waveform emphasizes brain activity that is time-locked and phase-locked to the TMS. This occurs because components that are consistently aligned in time and phase across trials sum up, while non-phase-locked activity, such as spontaneous oscillations and non-phase-locked responses, tends to cancel out due to its variability. As a result, the averaging process effectively enhances the phase-locked response by reducing uncorrelated activity, thereby isolating the evoked components of the brain’s reaction to TMS. Like for TFR, several approaches can be used to measure TEPs.

The most common way to quantify the magnitude and latency of a given TEP is to define a time window and identify the maximum point within that time window (either the most positive or negative peak, depending on the research question) in average potentials. The voltage at this point is referred to as the peak amplitude, while the time at which this peak occurs is known as the peak latency. One approach to quantifying TEPs is to measure the amplitude and latency of the TEP peaks across a subset of electrodes or a specific region of interest (ROI). The selection of electrodes for ROI analyses is determined in advance, based on scalp locations where responses are expected (e.g., the site of stimulation), or it can be derived using data-driven techniques, such as cluster-based permutation statistics.

An alternative method for measuring TEP peak amplitudes and latencies is to determine the mean amplitude. The mean amplitude represents the average voltage over a specific time window. In simpler terms, it calculates the voltage at each sample point within that time window and then computes the average of these voltages. All these measures have been widely used to evaluate TMS brain responses in M1. In healthy participants, TMS of M1 evokes several peaks, described at approximately 15 (N15), 30 (P30), 45 (N45), 60 (P60), 100 (N100), and 180 (P180) milliseconds (Casarotto et al., 2010, Kerwin et al., 2018, Lioumis et al., 2009, Rostami et al., 2022).

#### 27.17.3 Global and local mean field power

The global mean field power (GMFP) or global mean field amplitude (GMFA) is another measure that can be used to measure TEPs across all electrodes. The GMFP/GMFA computes the standard deviation (root mean square) across electrodes at a given point in time (Lehmann and Skrandies, 1980).

When only a ROI is used (i.e., a small number of electrodes from a specific brain region), this measure is called local mean field power (LMFP) or local mean field amplitude (LMFA). The LMFP/LMFA is the standard deviation (root mean square) across a specific ROI at a given point in time corresponding to TEP peaks (Casarotto et al., 2013, Casarotto et al., 2019).

#### 27.17.4 Perturbational complexity index

A synthetic measure of brain activity, which gauges at the same time the differentiation and integration of neuronal responses to direct perturbation, is the Perturbational Complexity Index (PCI). This measure is obtained from TMS-evoked potentials after estimation of cortical currents and statistical analysis to detect the deterministic component of the brain’s response to stimulation. PCI has been validated in a large benchmark population and has been shown to reliably discriminate between conditions of preserved consciousness and reduced consciousness in severely brain-injured patients (Casali et al., 2013, Casarotto et al., 2016, Sinitsyn et al., 2020). The sensitivity of PCI in detecting consciousness is not affected by the background EEG pattern, supporting the notion that EEG responses to perturbation convey complementary information to the observation of spontaneous EEG activity (Casarotto et al., 2024). Recently, the occurrence of sleep-like brain dynamics has been proposed as a more general mechanism behind loss of function after brain lesions (Massimini et al., 2024). An index of complexity of brain responses to perturbation, which can also be computed locally, has been implemented (Comolatti et al., 2019) and tested on patients with focal lesions, e.g., stroke patients (Sarasso et al., 2020).

#### 27.17.5 Other measures of signal propagation

The interhemispheric signal propagation (ISP) index is another measure that can be used to assess the spread of TMS-EEG responses. Specifically, the ISP measures how quickly the signals are transmitted between the two brain hemispheres. The ISP is also used to assess brain connectivity and function between two hemispheres (Casula et al., 2020). ISP is calculated by comparing the strength of TMS-evoked activity in one hemisphere with the corresponding TMS-evoked activity in the opposite hemisphere.

### 27.18 TMS-EEG reporting

Finally, it is critically important that researchers carefully and comprehensively report their TMS-EEG methods. While a consensus document on TMS-EEG reporting does not exist as of yet, we provide in Table 27.3, based on a proposal in a recent publication (Beck et al., 2024b) a list of items that should be reported. Moreover, authors are encouraged to adhere to principles of open science and provide, whenever possible, access to raw and/or preprocessed TMS-EEG data, and the code used for EEG preprocessing.

**Table 27.3:** Proposal for TMS-EEG reporting.

Sample demographics	Sample size, biological sex, age (mean ± SD, range),other specific clinical sample characteristics
Stimulation parameters	Hemisphere(s) stimulated, stimulation site(s), determination of stimulation site,target muscle(s) , method for coil positioning, number of trials, stimulation intensity/intensities, determination of stimulation intensity, inter-trial interval, jitter, coil geometry, coil diameter, coil name, coil orientation, stimulator, stimulator manufacturer, pulse waveform, pulse duration,orientation of induced current in the brain, recharge delay
Experimental setup	Type of auditory masking, type of ear protection, measures of PEP reduction,type of sham control, type of psychophysical assessment of auditory/somatosensory inputs, type of task, type of state control, type of participant instruction, type of blinding
EEG acquisition	EEG amplifier, EEG manufacturer, number of EEG electrodes, EEG electrode type, position of electrodes, sampling rate, electrode impedance, EOG,position of reference electrode, position of ground electrode, highpass filter (hardware filter), lowpass filter (hardware filter), recording software
EEG preprocessing	Highpass filter (software filter), lowpass filter (software filter), type of filter, notch filter, software used for preprocessing, epoching window, number of discarded epochs, number of discarded channels, criteria used to discard epochs, methods used to interpolate discarded channels, baseline correction interval, type of baseline correction, downsampling, re-referencing, window for removing the TMS artifact, type of interpolation, specific analysis tools for TMS-EEG data, ICA, type of ICA, number of ICAs performed, number of ICs removed, methods used to discard ICs
Data reporting	Time window of reporting, electrodes for reporting, readouts (TMS-EEG metrics) reported, statistical tests used

Abbreviations: ICA, independent component analysis; ICs, independent components; EOG, electrooculogram; PEP, peripheral-evoked potential.

## Declaration of competing interest

The authors declare the following financial interests/personal relationships which may be considered as potential competing interests: SC: Advisor and shareholder of Intrinsic Powers, a spin-off of the University of Milan. DCdA: Investigator-initiated research supported by Pfizer, Allergan, Mundipharma, Abbot, Medtronic. ZJD: Brainsway: Scientific Advisory Board; Brainsway and MagVenture: research support. PBF: Reimbursement for educational activities from Otsuka Australia Pharmaceutical Pty Ltd and equipment for research from Brainsway Ltd. Founder of TMS Clinics Australia. PJ: Consulting for Nexstim Plc (Helsinki, Finland). EK: Consulting fees from Nexstim Inc. CJK: Equity holder in Alto Neuroscience, Inc. and Flow Neuroscience, Inc. GK: Scientific co-founder and advisor of Sinaptica Therapeutics Inc. received payment or honoraria for lectures, presentations, speakers bureaus, manuscript writing, or educational events from: Epitech, Roche, Novo Nordisk. PL: Consultant for TMS-EEG to Sinaptica Ltd. and Nexstim Ltd. MM: Cofounder and shareholder of the spin-off Intrinsic Powers. TPM: Employed by Nexstim Plc. NCR: Received grant research funding from the Australian Research Council (ARC), and the Medical Research Future Fund (MRFF); contract research funding from the Commonwealth Scientific and Industrial Research Organisation (CSIRO), and CMAX Clinical Research PTY LTD; consultancy fees from OVID Therapeutics Inc. MR: Shareholder and advisor of Intrinsic Powers Inc., Spin-off of the University of Milan. SS: Advisor of Intrinsic Powers, Inc., a spin-off of the University of Milan. HRS: Received honoraria as speaker and consultant from Lundbeck AS, Denmark, and as editor (Neuroimage Clinical) from Elsevier Publishers, Amsterdam, The Netherlands. Received royalties as book editor from Springer Publishers, Stuttgart, Germany, Oxford University Press, Oxford, UK, and from Gyldendal Publishers, Copenhagen, Denmark. AZ: Has invented TMS coils and systems, which are produced by BrainsWay LTD, and has financial interest in BrainsWay LTD. CZ: Minority equity ownership in sync2brain GmbH (Germany), a manufacturer of a real-time EEG analysis device for personalized TMS applications. RJI: Co-founder and minority owner of the company Cortisys. All other authors have declared to have no conflicts of interest.
